# Supplementary material for: A gut-brain-gut axis orchestrates host responses counteracting microbiome-induced iron insufficiency
Source: EMBO J. 2025 Nov 3;44(24):7590–619. doi: 10.1038/s44318-025-00619-6 (PMC12705764; doi:10.1038/s44318-025-00619-6)
Supplement: Supplementary file 2 — Appendix [file 44318_2025_619_MOESM2_ESM.pdf]

A gut-brain-gut axis orchestrates host responses counteracting microbiome-induced iron insufficiency

Guanqun Li[1,2,3,4], Yangyang Wu[1,2,3,4], Xiaowen Huang[1,2,3,4], Minghui Du[1,2,3], Hongyun Tang[1,2,3,5,6 *]

Table of Contents:

Appendix Figure S1. pp. 2

Appendix Figure S2. pp. 3

Appendix Figure S3. pp. 4-5

Appendix Figure S4. pp. 5-6

Appendix Figure S5. pp. 6-7

Appendix Fig.S1

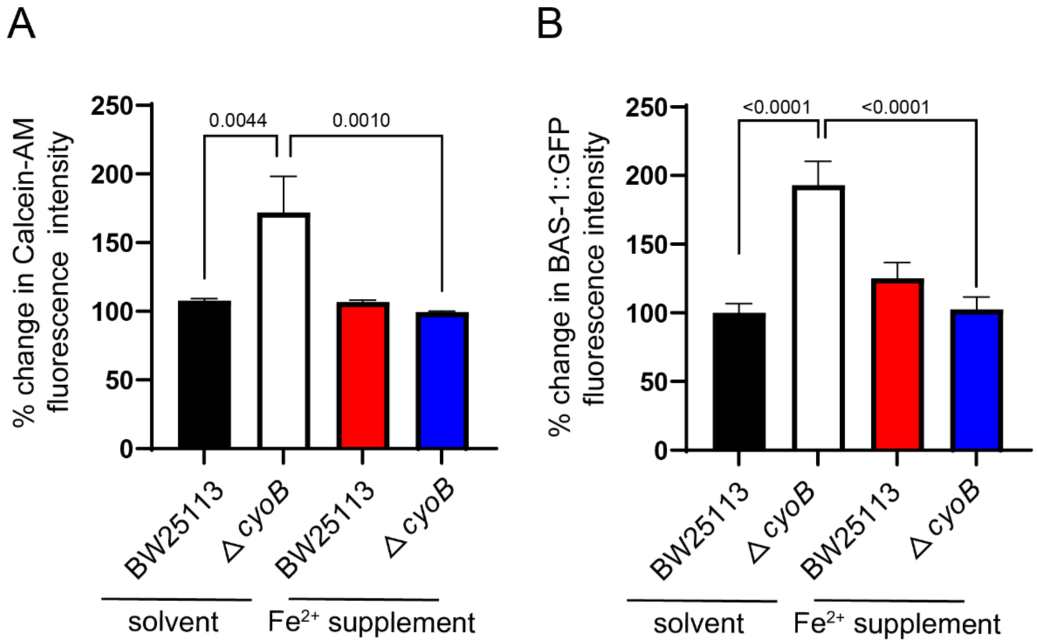

Appendix Fig. S1 $FeSO_4$ (Fe^{2+} iron) supplementation suppresses *ΔcyoB*-induced reduction in labile iron and the increase in BAS-1::GFP.

(A) Bar graphs indicate that the increase in Calcein fluorescence induced by the *ΔcyoB E. coli* mutant was reversed by supplementation with 4 mM $FeSO_4$. Experiments were conducted in N2 wildtype worms. Percent changes in Calcein fluorescence intensity were normalized to levels in animals treated with both BW25113 and solvent. P values were determined by one-way ANOVA; n > 30 for each group.

(B) Bar graphs illustrate that $FeSO_4$ supplementation reverses the induction of BAS-1::GFP expression caused by *ΔcyoB* bacteria treatment. Experiments were performed in *Is[bas-1p::BAS-1::GFP]* worms. Percent change in BAS-1::GFP fluorescence intensity was calculated by normalizing to levels in animals treated with both BW25113 and solvent. Data are presented as mean ± SEM. P values were determined by one-way ANOVA; n > 20 for each group.

Appendix Fig.S2

A

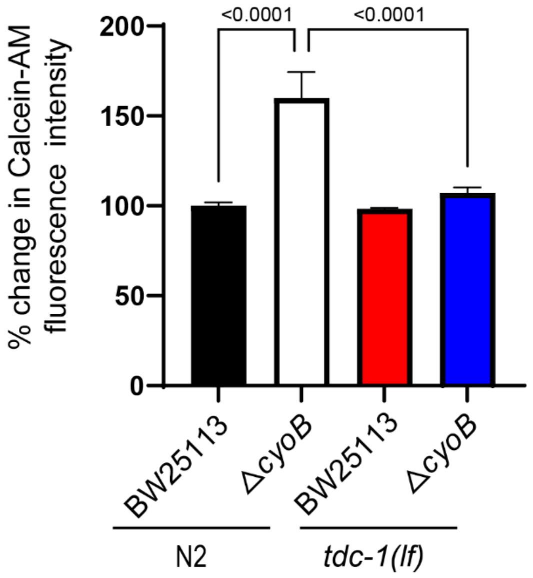

Appendix Fig. S2 Octopamine and tyramine signaling suppress the decrease in labile iron induced by *ΔcyoB E. coli*

(A) A bar graph indicates that the *tdc-1(n3420)* mutation alleviates the iron decrease caused by *ΔcyoB E. coli*. The percentage change in Calcein fluorescence was calculated by normalizing to the levels in wild-type N2 animals treated with BW25113 bacteria. Data represent the mean ± SEM. P values were determined by one-way ANOVA; n > 25 in each group. Eliminating octopamine and tyramine by knocking out TDC-1, a tyrosine decarboxylase(Alkema, Hunter-Ensor et al., 2005), suppresses the reduction in iron availability caused by *ΔcyoB E. coli*. This reveals that neurons use tyramine and octopamine to suppress labile iron levels, opposing the iron-elevating effects of serotonin and dopamine. This may explain why *cat-1(lf)* mutants, defective in transporting monoamines including dopamine, serotonin, tyramine, and octopamine, exhibit a less severe labile iron reduction than *bas-1(lf)* mutants, which are impaired in serotonin/dopamine synthesis, under *ΔcyoB E. coli* challenge. The presence of both positive and negative neuronal regulation of iron levels aligns with the evolutionary need for precise iron control in the organism.

Appendix Fig.S3

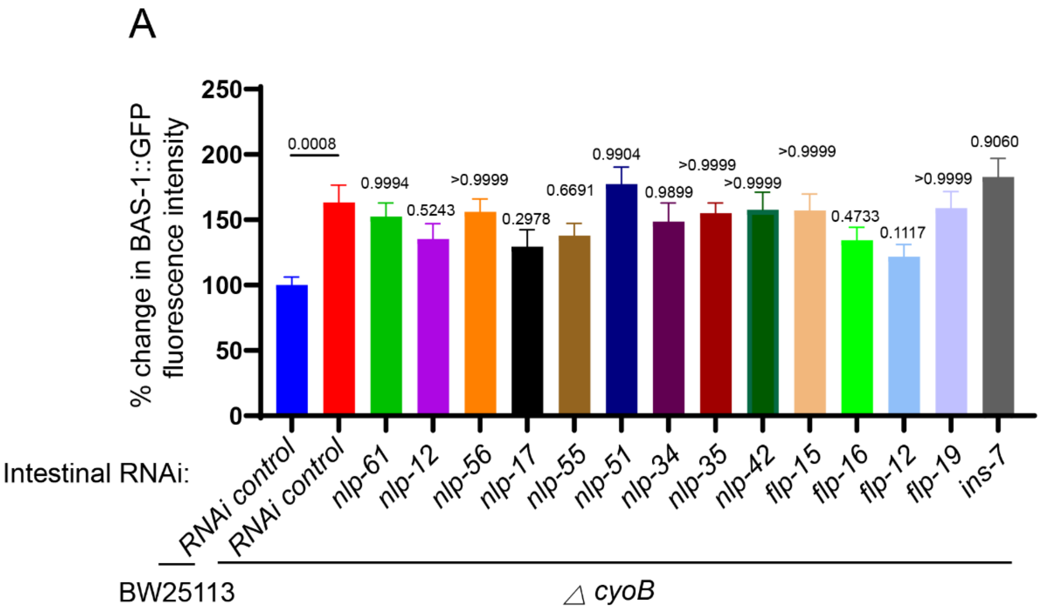

Appendix Fig. S3 Intestine-specific RNAi analysis of peptides for their potential roles in gut-to-brain cell-nonautonomous signaling

(A) Bar graphs showing that intestine-specific RNAi against the indicated peptide-encoding genes does not suppress the *ΔcyoB E. coli*-induced increase in neuronal BAS-1 expression. We identified 14 neuropeptides (NLPs, FLPs, INS-family), with at least a 2-fold upregulation in response to bacterial-induced iron deficiency and expression restored upon iron repletion, through RNA-seq profiling of *C. elegans* exposed to four treatments, including BW25113, *ΔcyoB E. coli*, BW25113 supplemented with iron, and *ΔcyoB E. coli* supplemented with iron. However, intestine-specific RNAi targeting these peptides did not attenuate *ΔcyoB E. coli*-induced neuronal BAS-1::GFP activation, indicating that none of these single neuropeptides are required for gut-to-brain signaling in the presence of *ΔcyoB E. coli*. These findings suggest functional redundancy among neuropeptide families or the existence of alternative mechanisms in gut-to-brain communication, underscoring the complexity of this regulatory axis. The *rde-1(lf); kbIs7 [nhx-2p::RDE-1 + rol-*

6(su1006)] worm strain expressing BAS-1::GFP, was utilized for intestine-specific knockdown. The percentage change in BAS-1::GFP fluorescence was calculated by normalizing to the levels in animals treated with control RNAi and BW25113. Data represent the mean ± SEM. Statistical analysis was performed using one-way ANOVA; n > 30 per group.

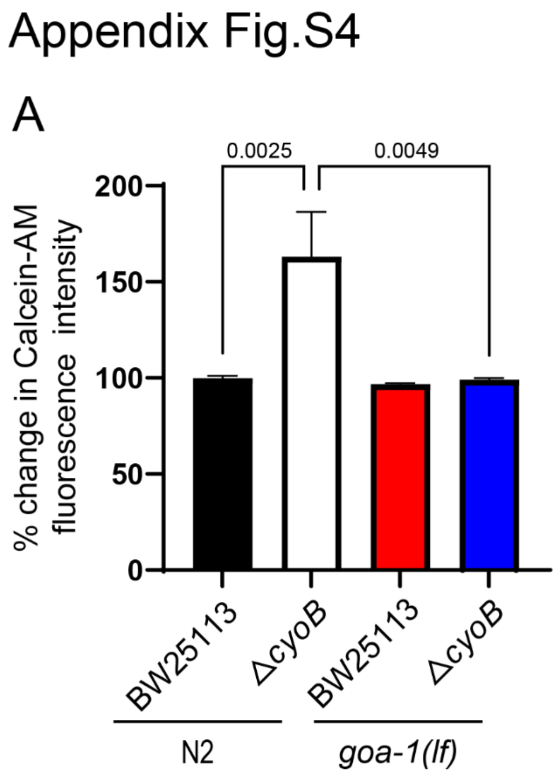

Appendix Fig. S4 Enhancing neuronal excitability suppresses the *ΔcyoB E. coli*-induced decrease in labile iron.

(A) Bar graphs indicate that the *goa-1(sa734)* mutation alleviated the reduction in labile iron caused by the *ΔcyoB E. coli*. The percentage change in Calcein fluorescence was calculated by normalizing to the levels in wild-type N2 animals treated with BW25113 bacteria. Data represent the mean ± SEM. P values were determined by one-way ANOVA; n > 25 per group. Inhibiting $G\alpha_0$ signaling, which typically suppresses the electrical activity of a set of neurons, including serotonergic

neurons(Ravi, Zhao et al., 2021), using the *goa-1(sa734)* mutant, which lacks the Gα_0 subunit, potentiates serotonergic neuronal activity and serotonin release. Therefore, we hypothesized that this mutation would suppress the iron deficiency phenotype like exogenous serotonin supplementation. Indeed, compared to the increase in Calcein staining in N2 worms treated with *ΔcyoB* bacteria, the *goa-1(sa734)* mutant worms exhibited no such increase in the Calcein signal, reflecting the suppressive effect of the *goa-1(sa734)* mutation on the *ΔcyoB*-induced reduction in free iron levels. Therefore, neuronal electrical activity may promote labile iron levels by modulating neurotransmitter secretion, adding another layer of neuronal regulation to iron metabolism.

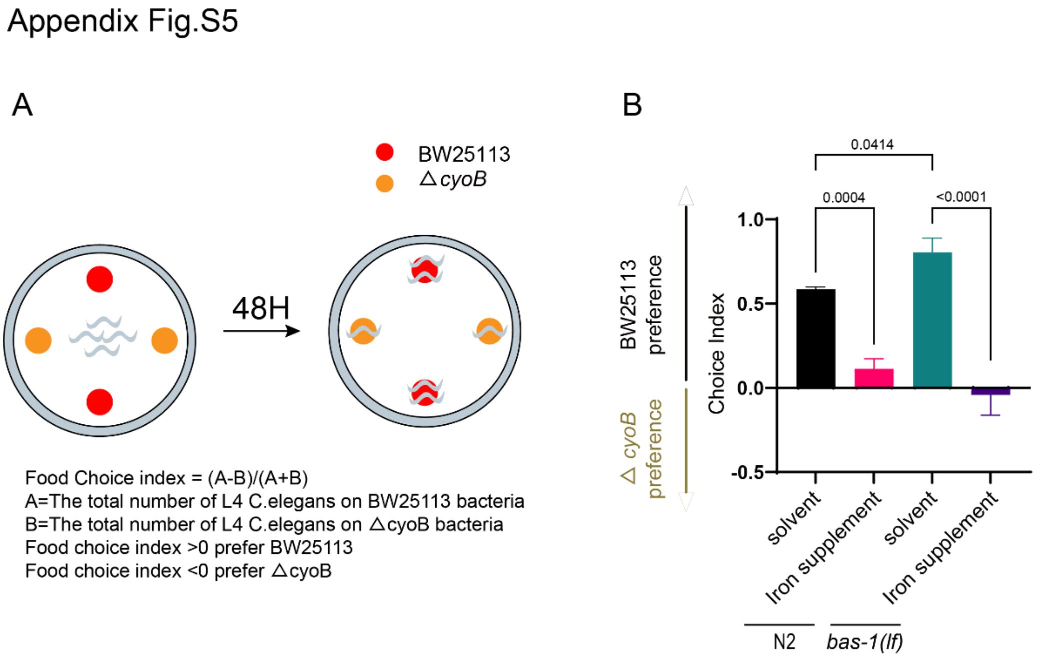

Appendix Fig. S5 Decreased iron availability in *C. elegans* modifies its behavior to choose food with high iron content.

(A) A schematic diagram illustrating the food choice behavior assay, as previously performed(Margie, Palmer et al., 2013), and the method for calculating the choice index. (B) Bar graphs depicting the food choice index between BW25113 and the *ΔcyoB E. coli*, with and without iron supplementation, in wild-type and *bas-1(lf) C. elegans*. Error bars represent SEM. The p-value is derived from one-way ANOVA.

n > 100. Wild-type *C. elegans* exhibit a strong preference for BW25113 wildtype *E. coli* over *ΔcyoB E. coli*, likely due to the iron-depleting effects of the latter. This avoidance is abolished by iron supplementation, confirming that reduced iron availability, rather than other *ΔcyoB* bacteria-related effects, drives the preference for the wildtype *E. coli*. Consistent with this, *bas-1(lf)* mutants, which display exacerbated iron deficiency, avoid *ΔcyoB* bacteria more robustly than wild-type worms, a behavior also reversed by iron repletion. Together, these findings support that iron deficiency in the animal drives foraging behavior to prioritize iron-rich food sources, revealing a link between iron metabolic state and animal's behavior.

References

Alkema MJ, Hunter-Ensor M, Ringstad N, Horvitz HR (2005) Tyramine Functions independently of octopamine in the Caenorhabditis elegans nervous system. *Neuron* 46: 247-60

Margie O, Palmer C, Chin-Sang I (2013) C. elegans chemotaxis assay. *J Vis Exp*: e50069

Ravi B, Zhao J, Chaudhry SI, Signorelli R, Bartole M, Kopchock RJ, Guijarro C, Kaplan JM, Kang L, Collins KM (2021) Presynaptic Galphao (GOA-1) signals to depress command neuron excitability and allow stretch-dependent modulation of egg laying in Caenorhabditis elegans. *Genetics* 218
