# Supplementary figures and images for: A gut-brain-gut axis orchestrates host responses counteracting microbiome-induced iron insufficiency (part 1 of 2)
Source: EMBO J. 2025 Nov 3;44(24):7590–619. doi: 10.1038/s44318-025-00619-6 (PMC12705764; doi:10.1038/s44318-025-00619-6)

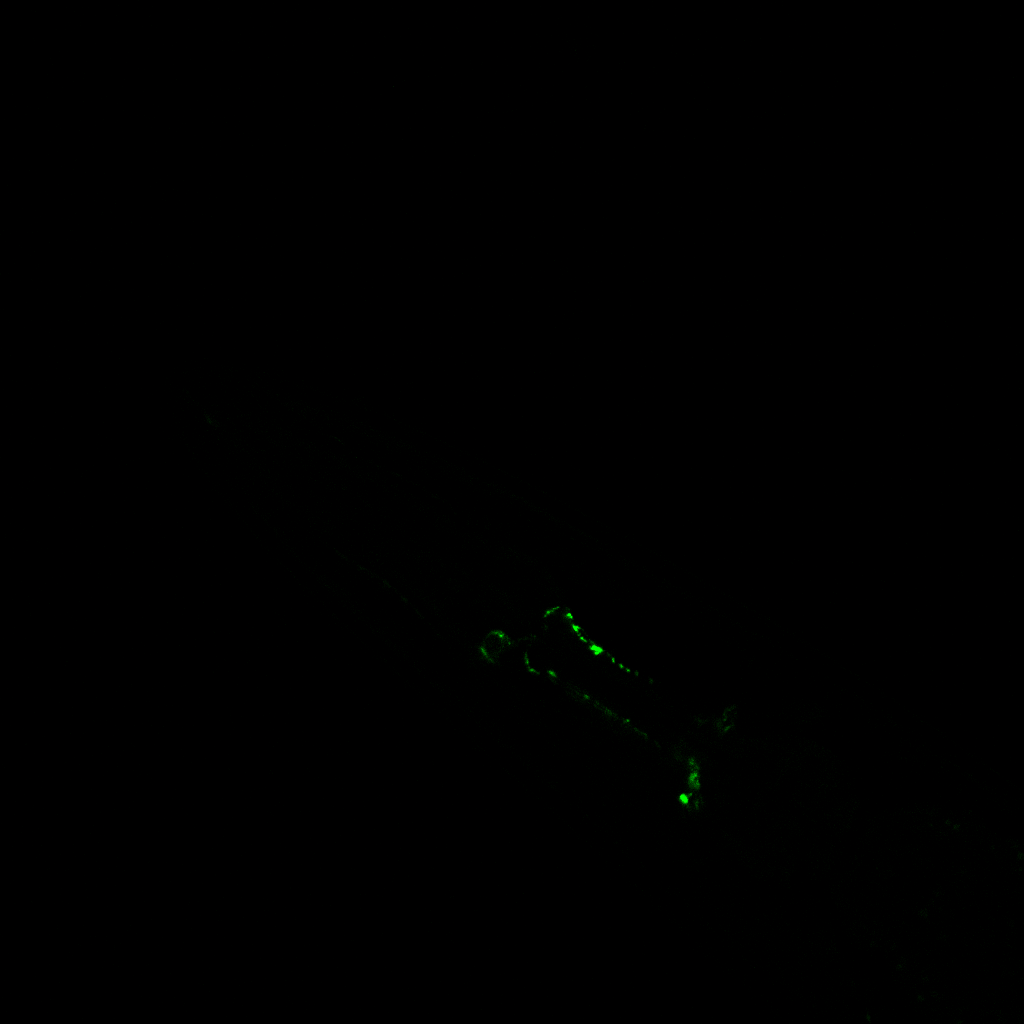

Supplement: Supplementary file 4 — Source data Fig. 1 [file 44318_2025_619_MOESM4_ESM.zip › Figure 1/1D/BW/BAS-1GFP.tif]

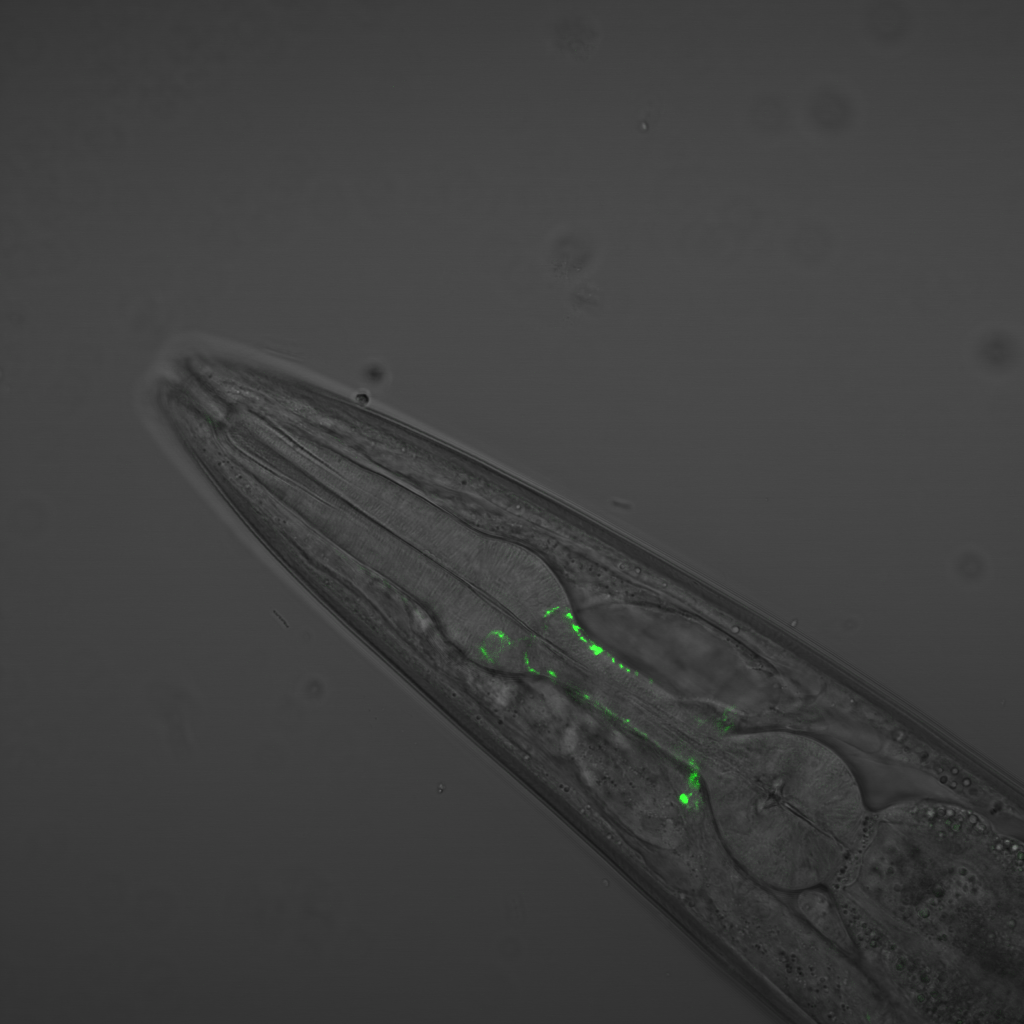

Supplement: Supplementary file 4 — Source data Fig. 1 [file 44318_2025_619_MOESM4_ESM.zip › Figure 1/1D/BW/Merge.tif]

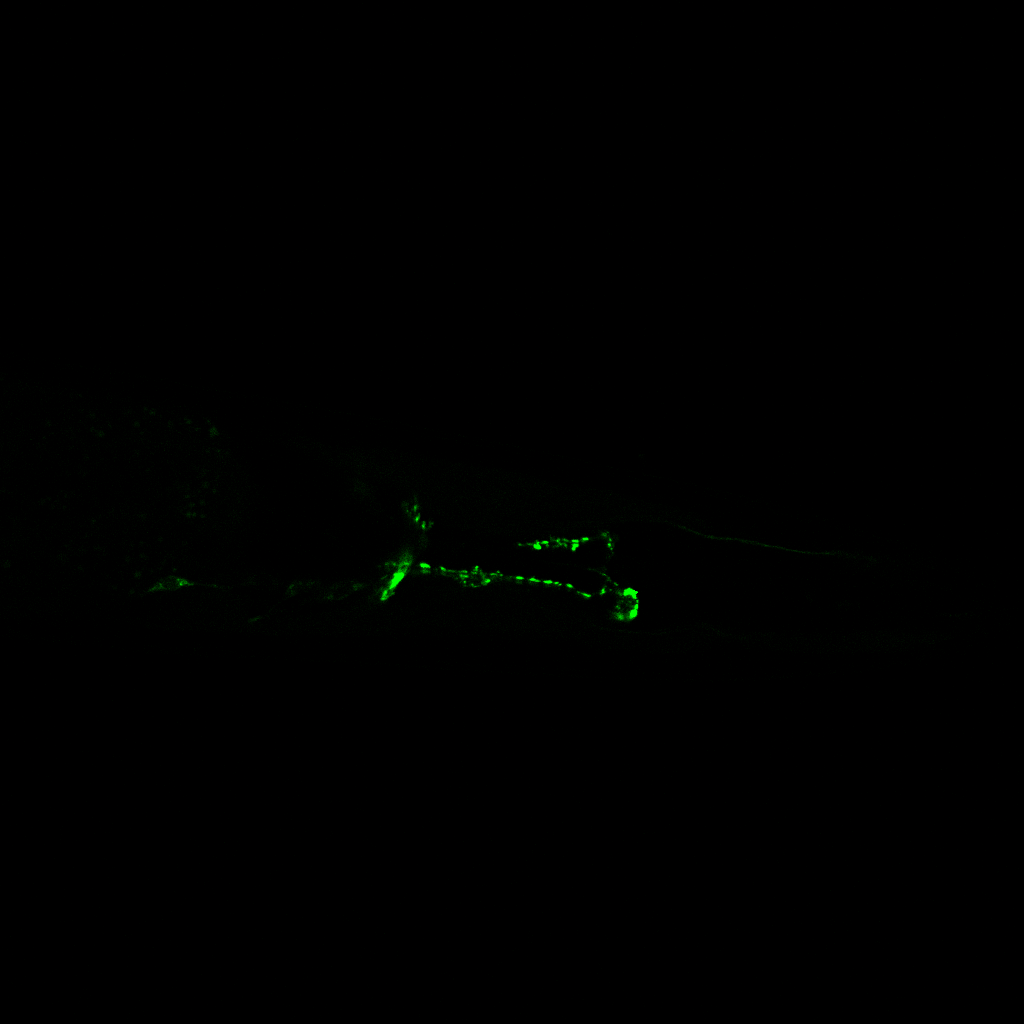

Supplement: Supplementary file 4 — Source data Fig. 1 [file 44318_2025_619_MOESM4_ESM.zip › Figure 1/1D/CYOB/BAS-1GFP.tif]

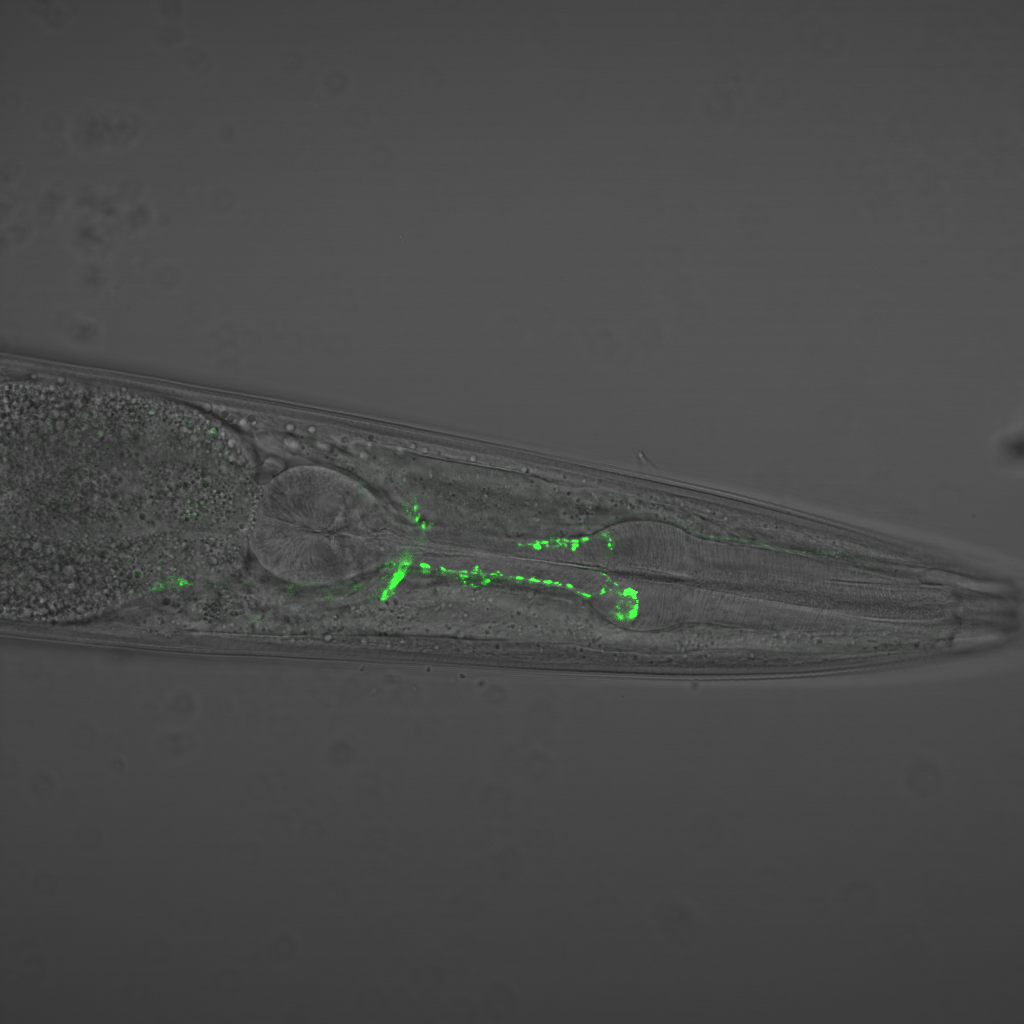

Supplement: Supplementary file 4 — Source data Fig. 1 [file 44318_2025_619_MOESM4_ESM.zip › Figure 1/1D/CYOB/Merge.tif]

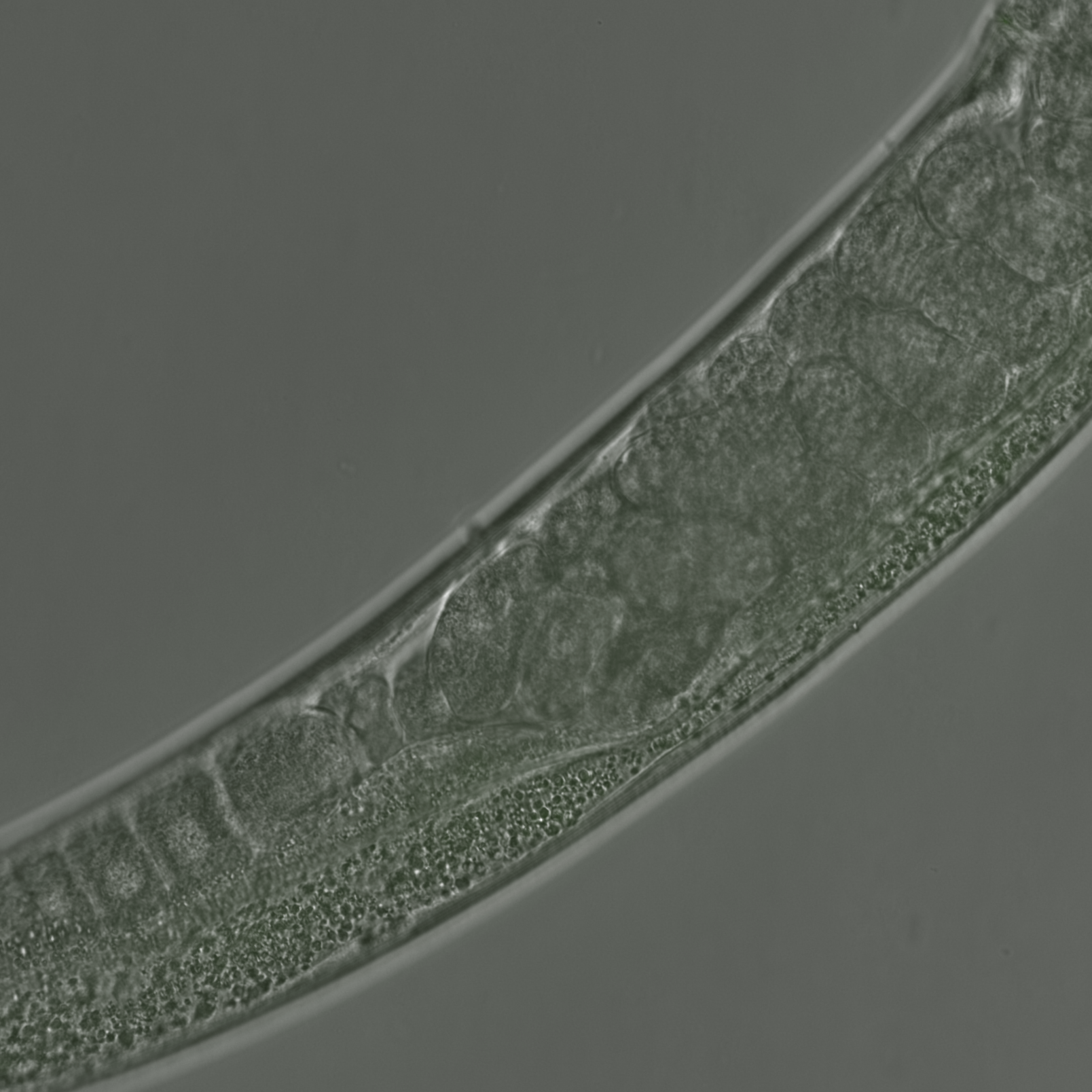

Supplement: Supplementary file 5 — Source data Fig. 2 [file 44318_2025_619_MOESM5_ESM.zip › Figure 2/2A/a.tif]

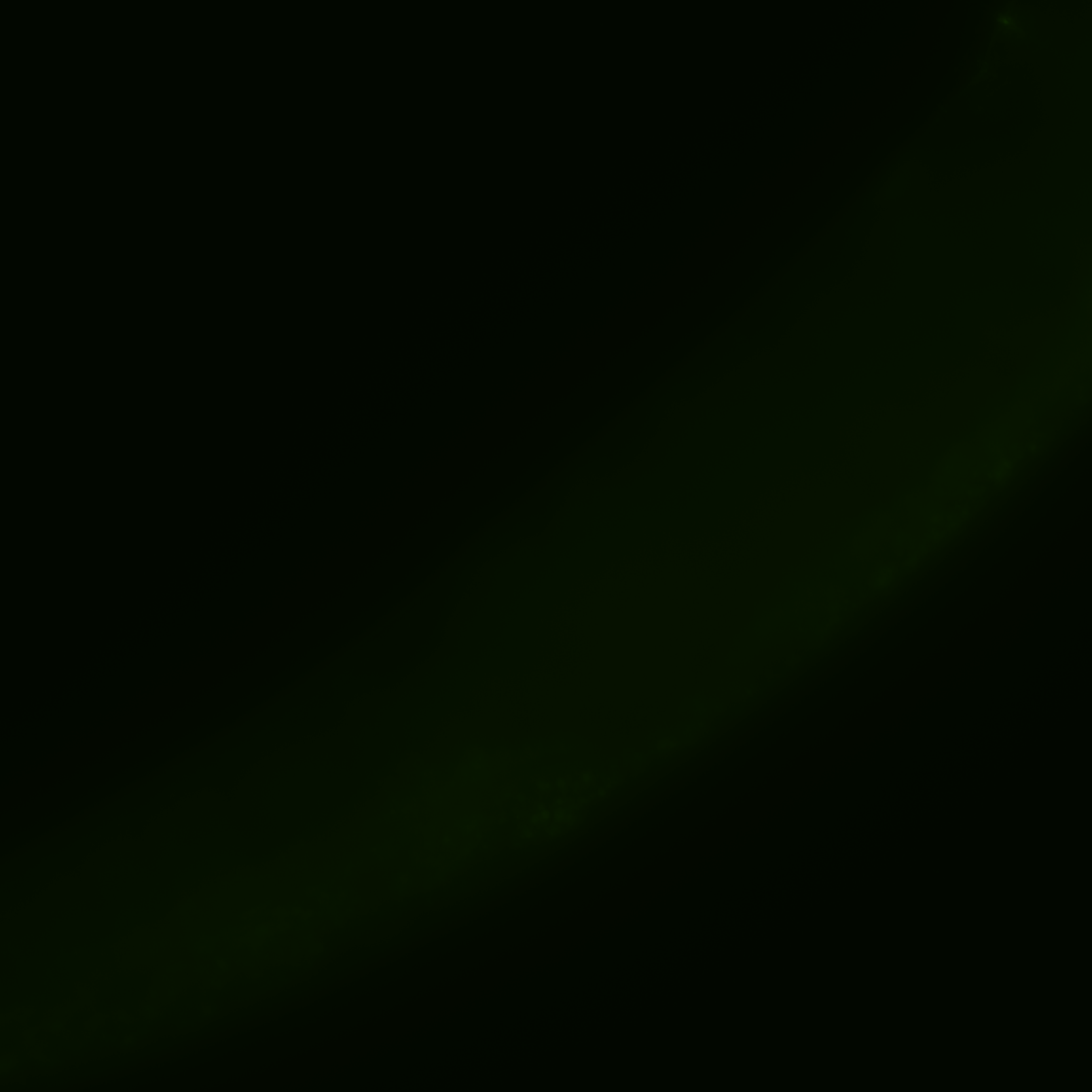

Supplement: Supplementary file 5 — Source data Fig. 2 [file 44318_2025_619_MOESM5_ESM.zip › Figure 2/2A/b.tif]

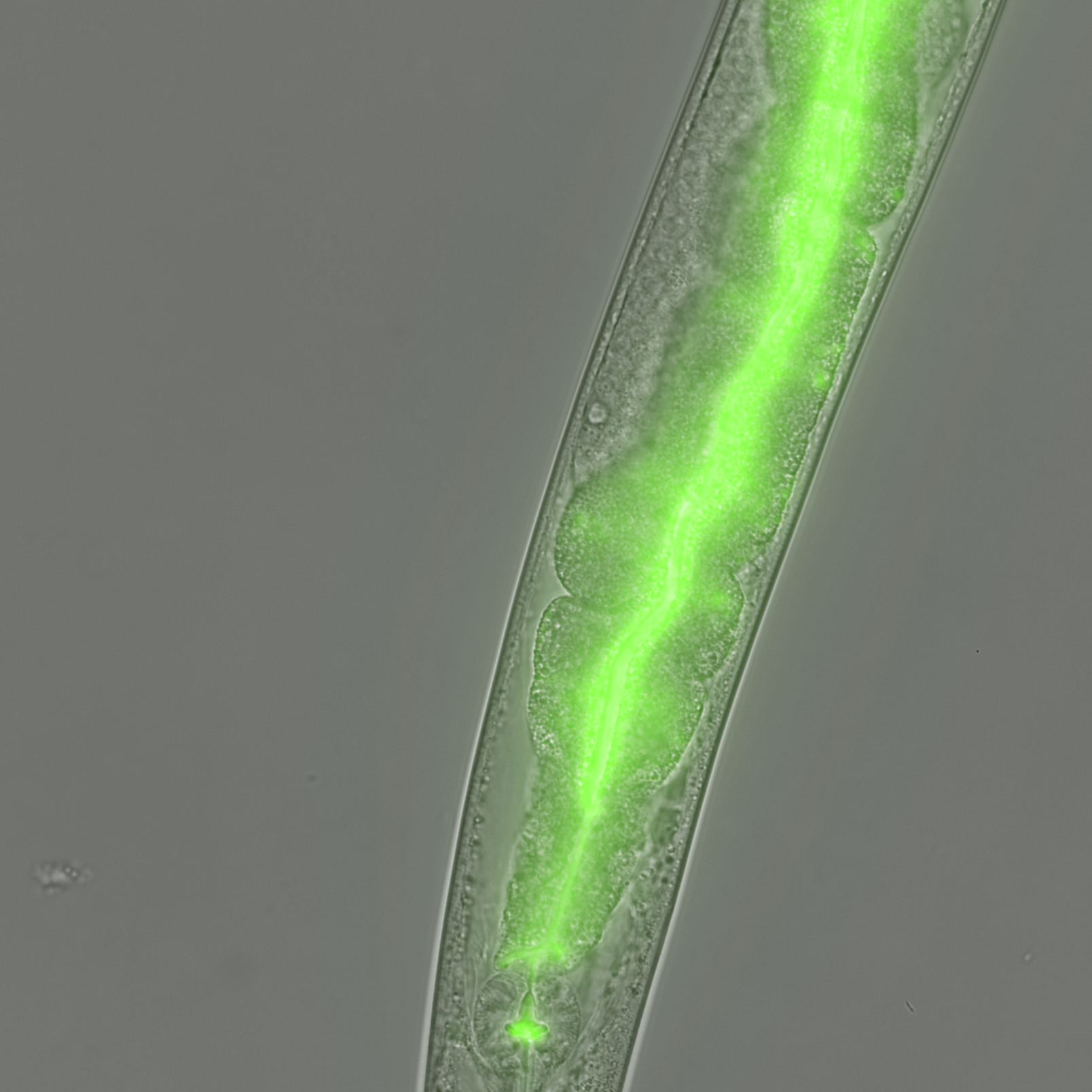

Supplement: Supplementary file 5 — Source data Fig. 2 [file 44318_2025_619_MOESM5_ESM.zip › Figure 2/2A/c.tif]

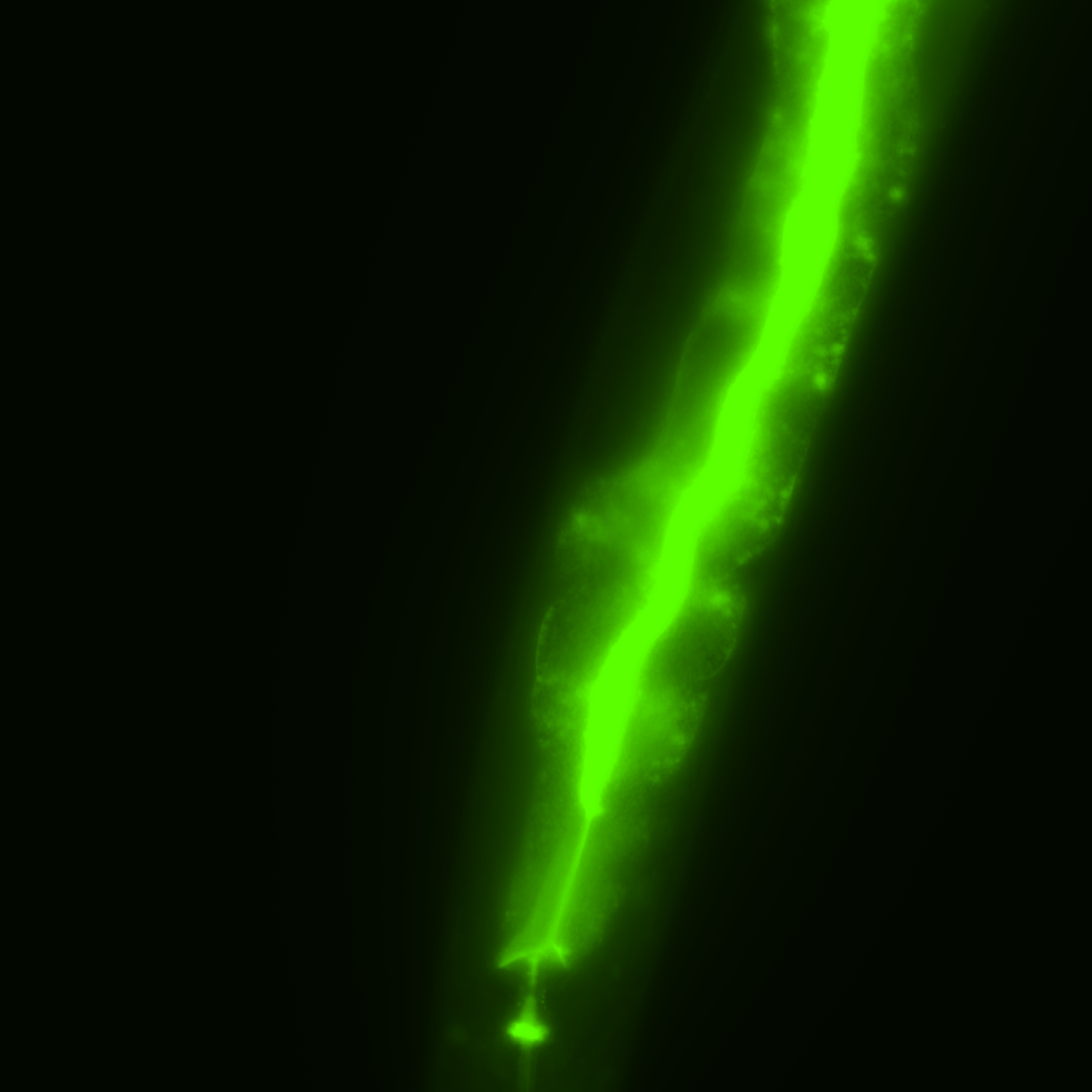

Supplement: Supplementary file 5 — Source data Fig. 2 [file 44318_2025_619_MOESM5_ESM.zip › Figure 2/2A/d.tif]

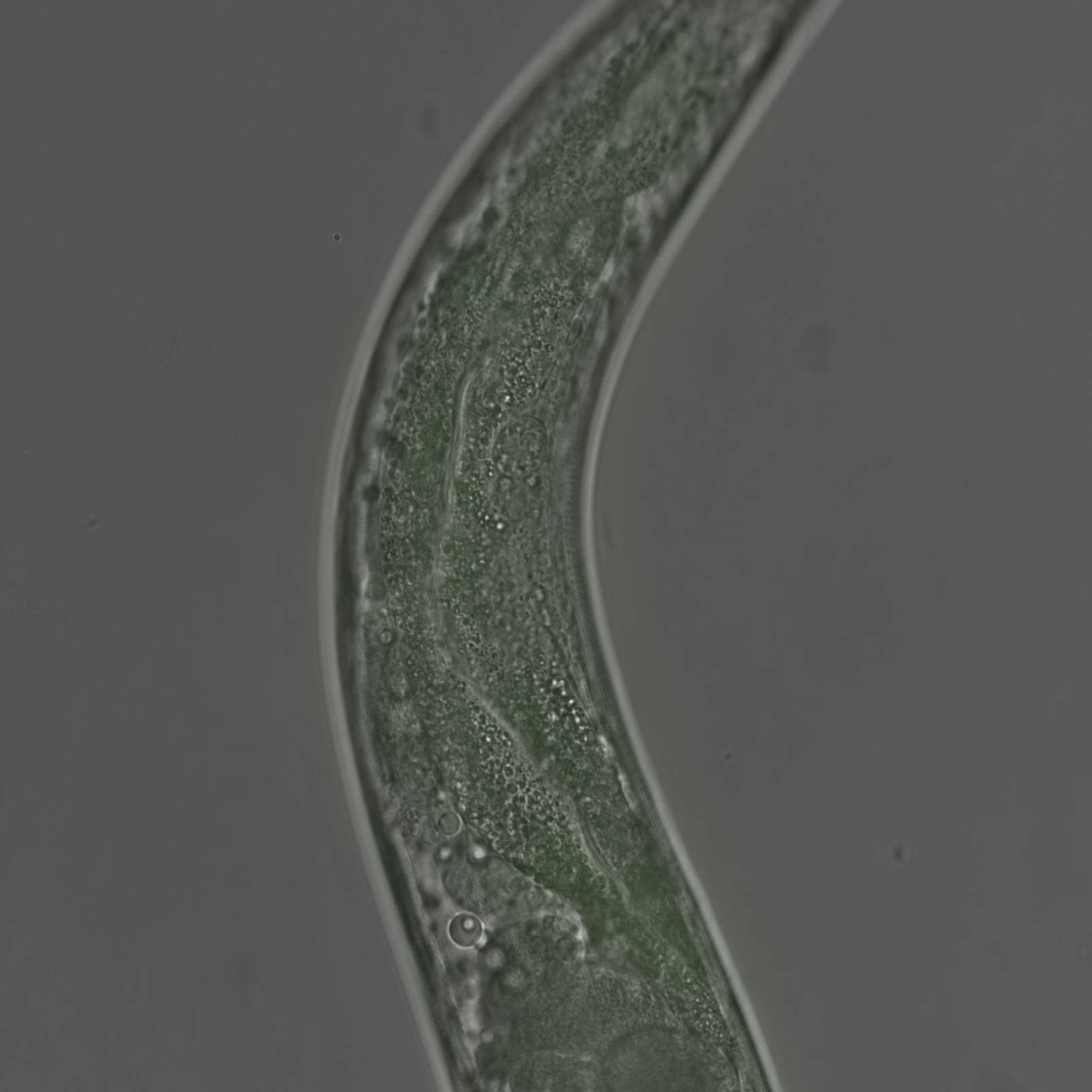

Supplement: Supplementary file 5 — Source data Fig. 2 [file 44318_2025_619_MOESM5_ESM.zip › Figure 2/2A/e.tif]

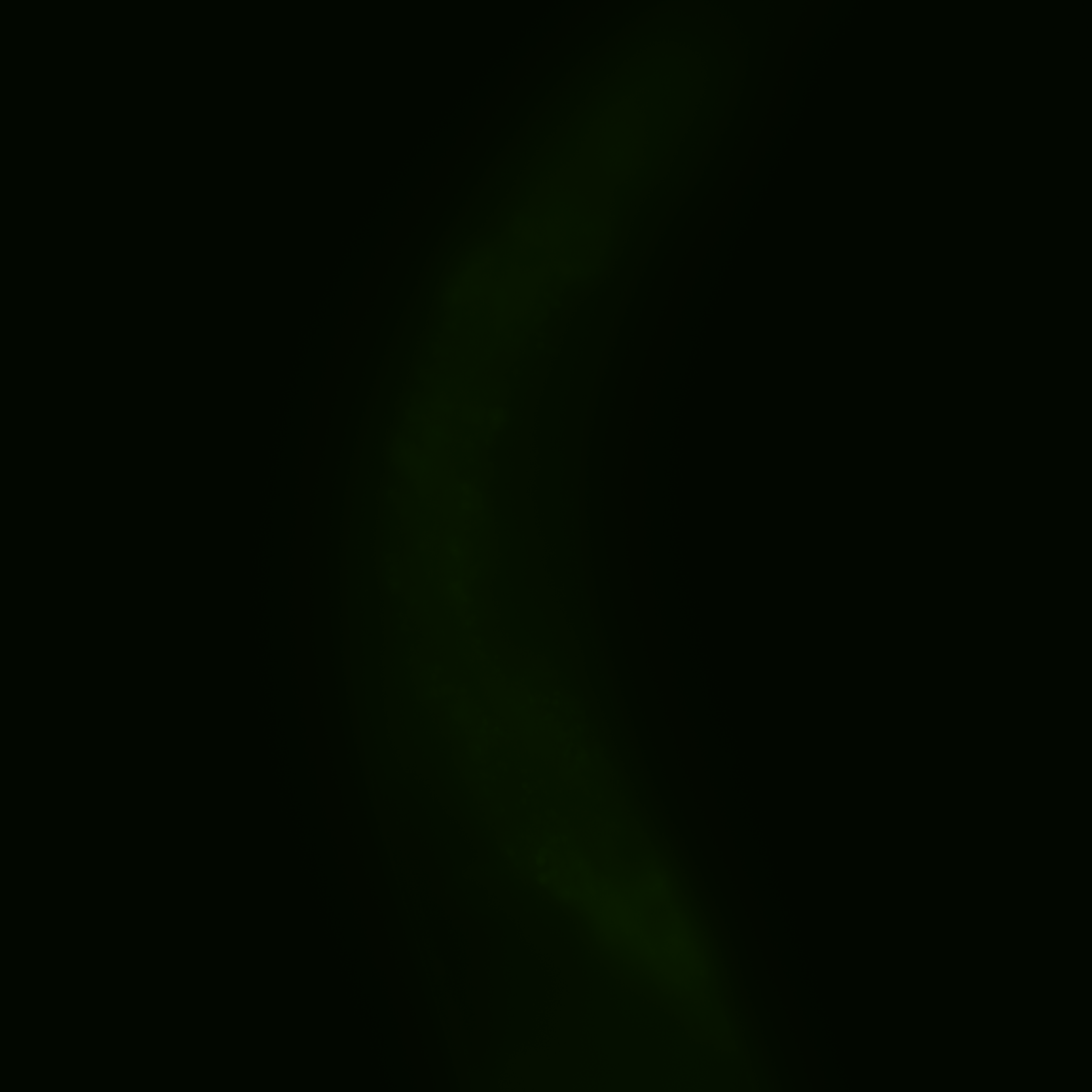

Supplement: Supplementary file 5 — Source data Fig. 2 [file 44318_2025_619_MOESM5_ESM.zip › Figure 2/2A/f.tif]

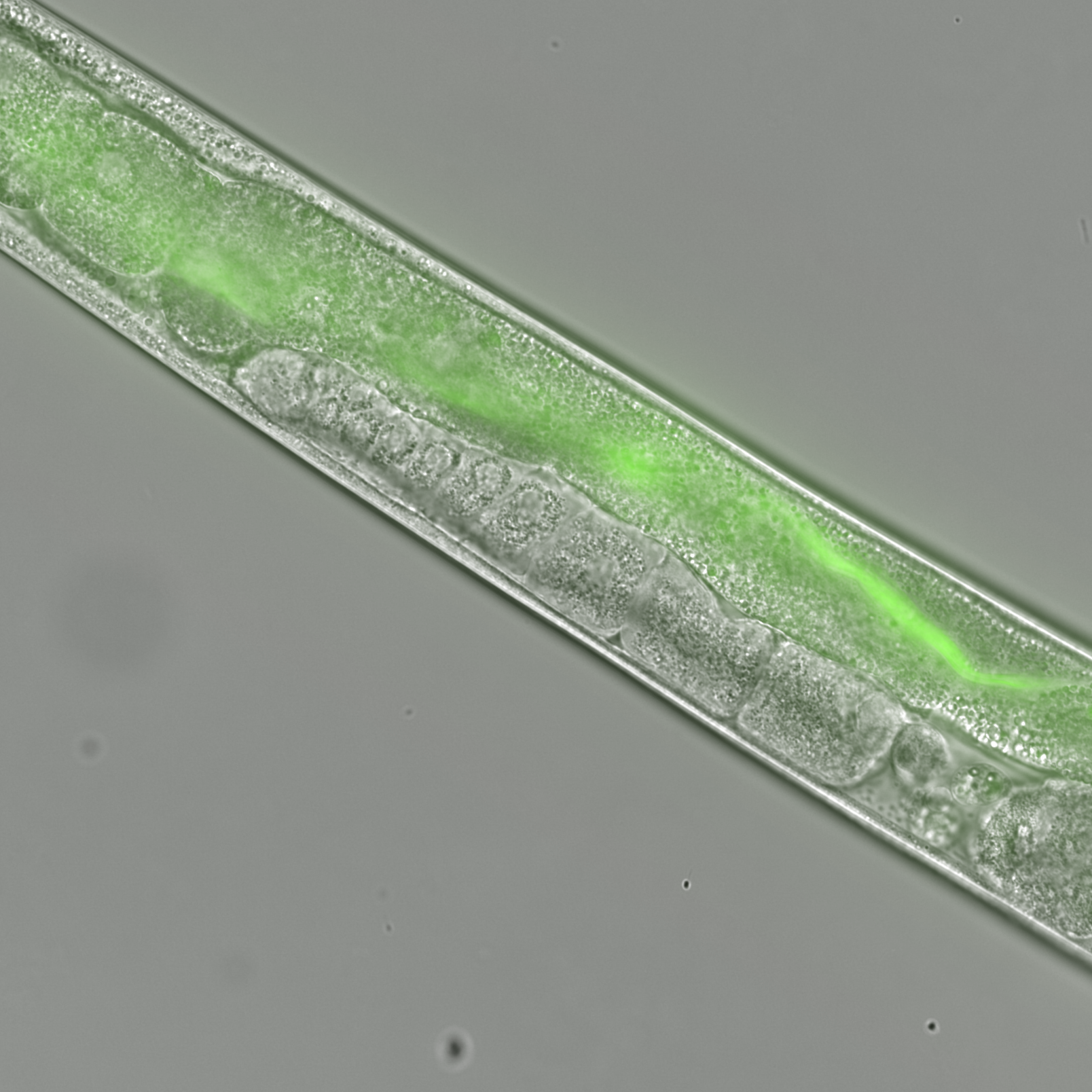

Supplement: Supplementary file 5 — Source data Fig. 2 [file 44318_2025_619_MOESM5_ESM.zip › Figure 2/2A/g.tif]

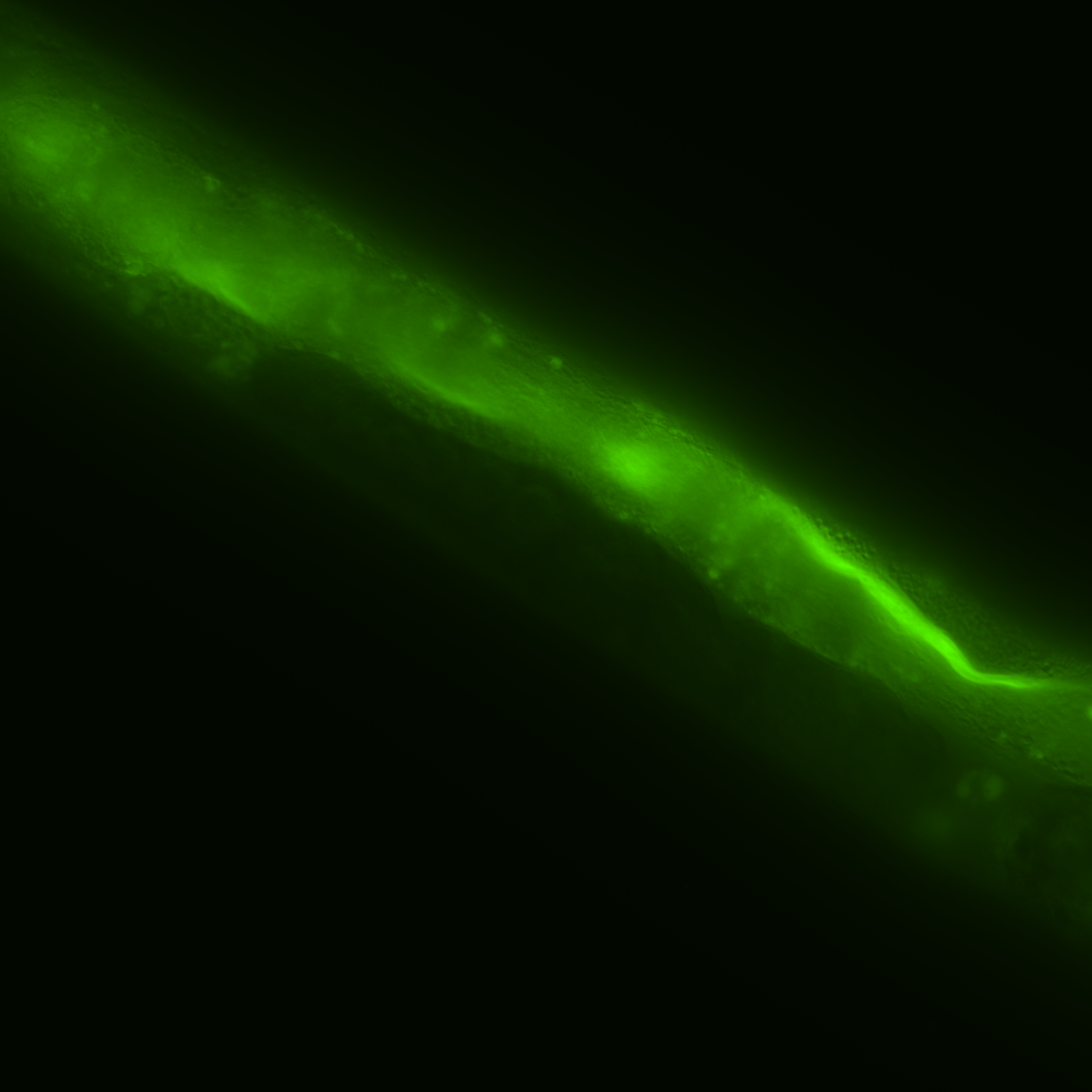

Supplement: Supplementary file 5 — Source data Fig. 2 [file 44318_2025_619_MOESM5_ESM.zip › Figure 2/2A/h.tif]

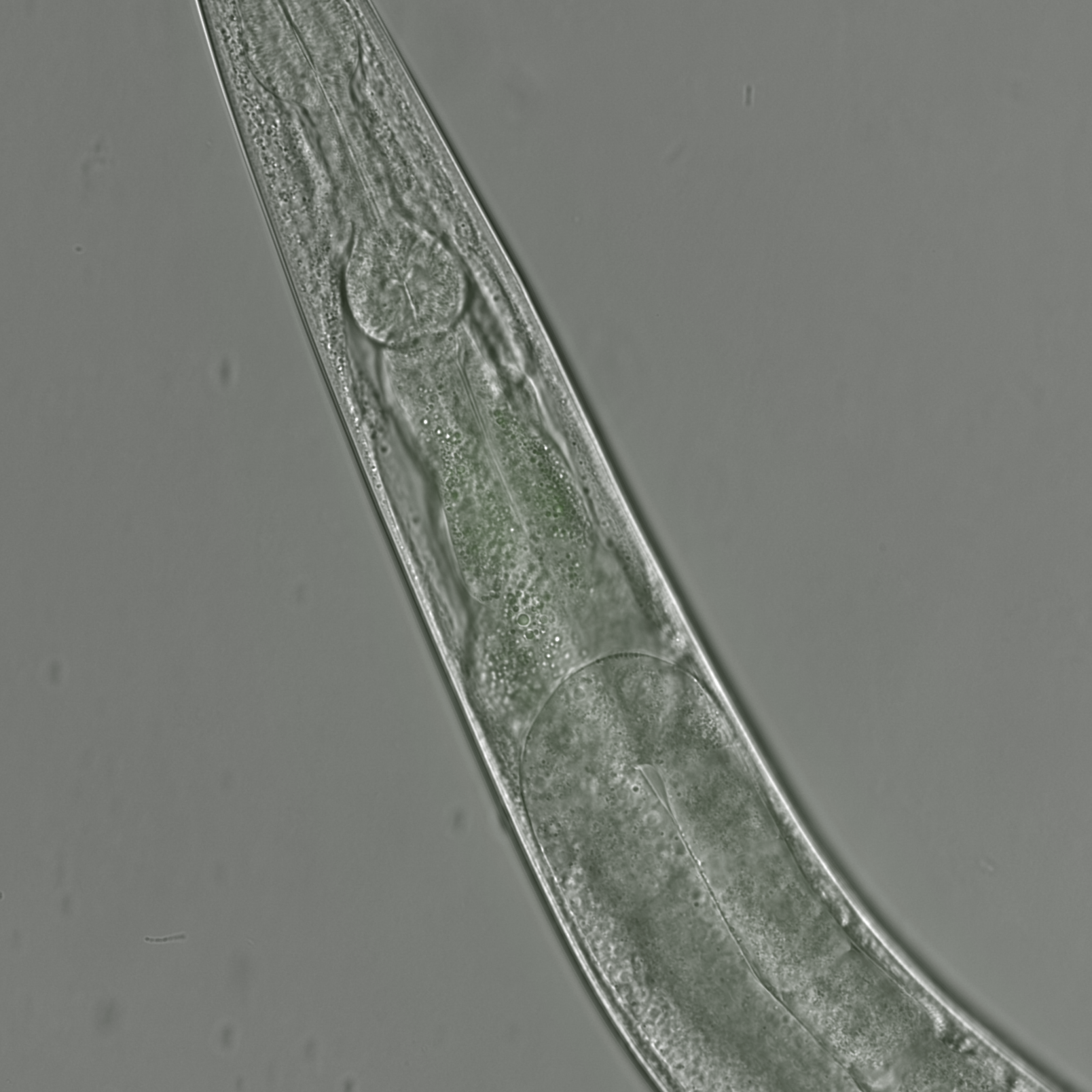

Supplement: Supplementary file 5 — Source data Fig. 2 [file 44318_2025_619_MOESM5_ESM.zip › Figure 2/2A/i.tif]

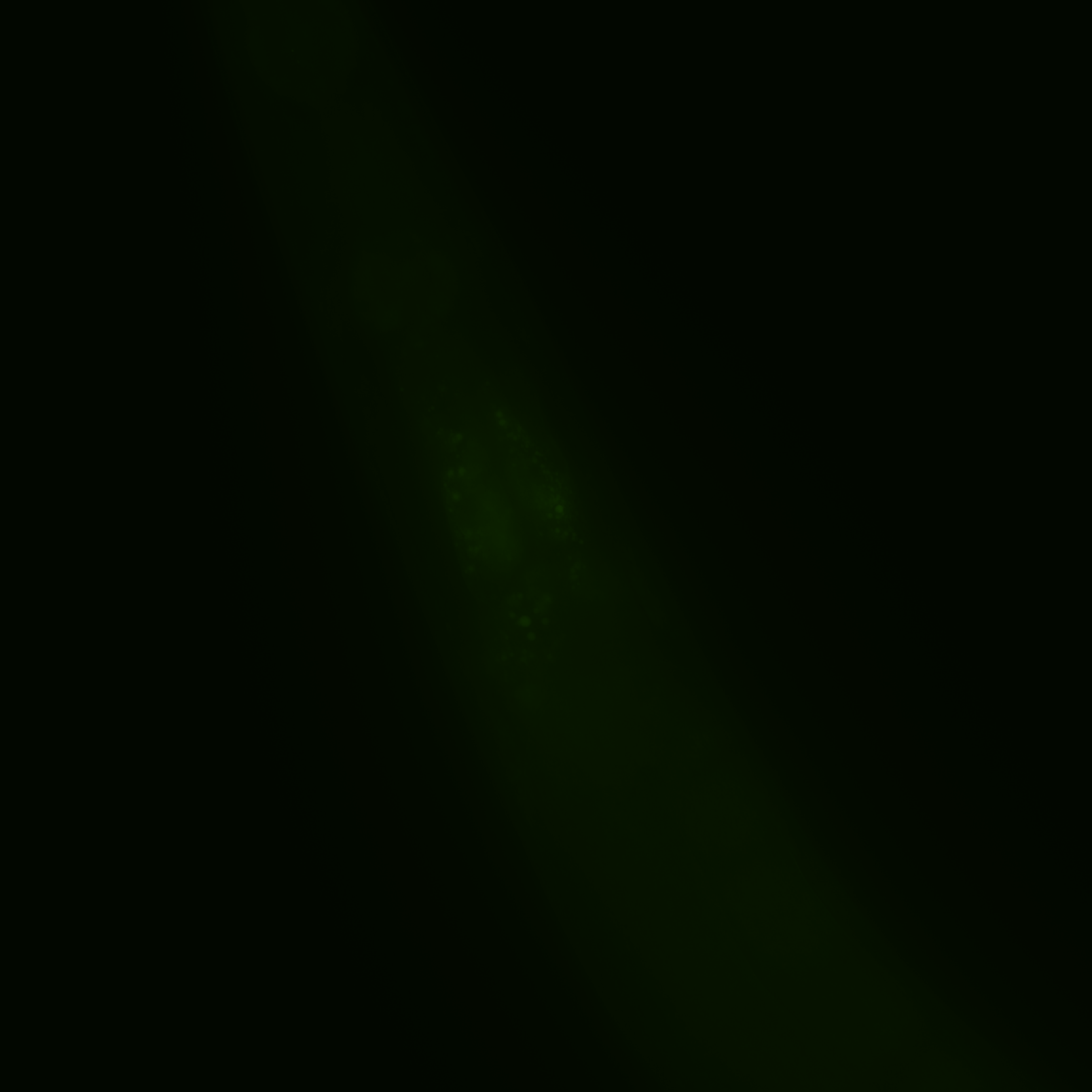

Supplement: Supplementary file 5 — Source data Fig. 2 [file 44318_2025_619_MOESM5_ESM.zip › Figure 2/2A/j.tif]

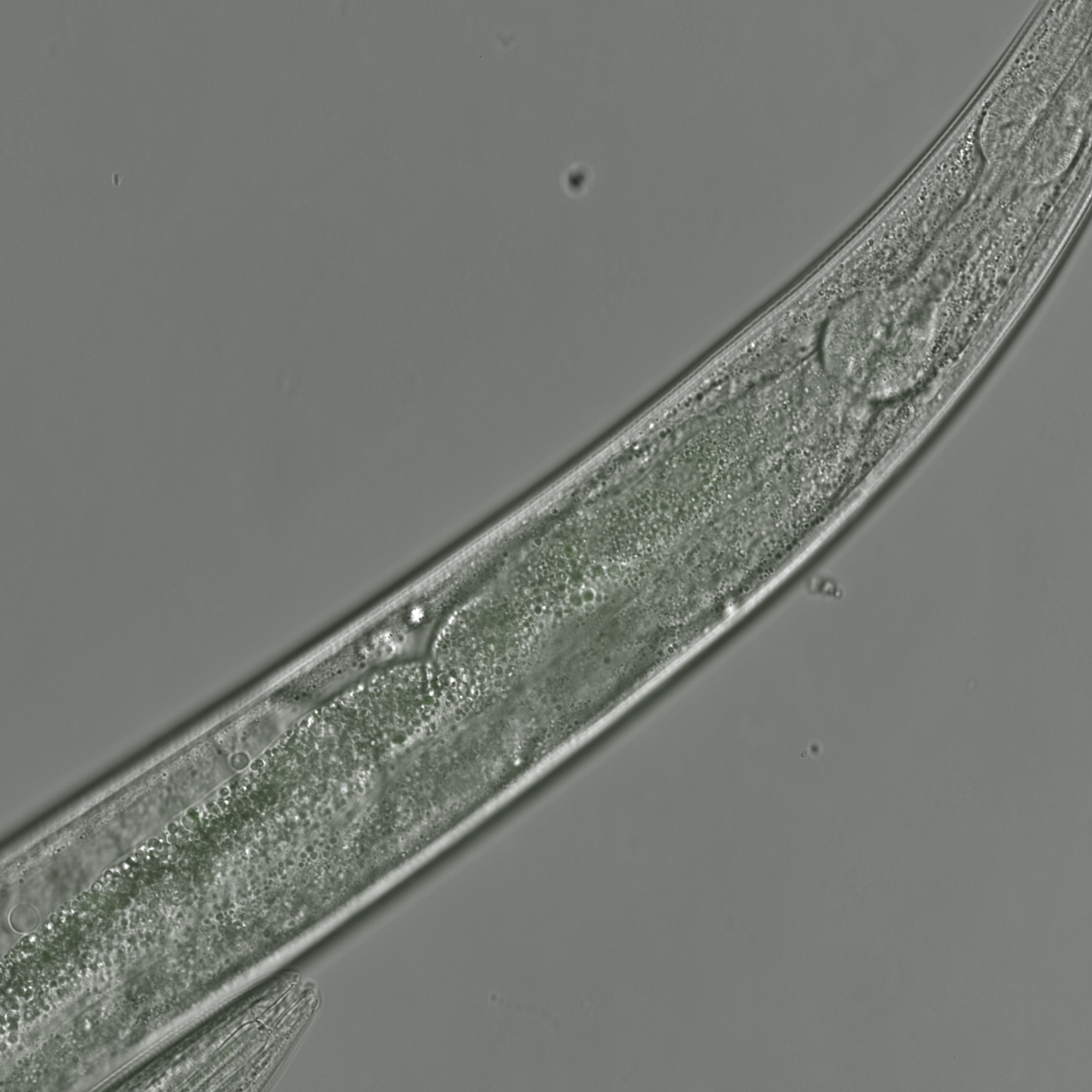

Supplement: Supplementary file 5 — Source data Fig. 2 [file 44318_2025_619_MOESM5_ESM.zip › Figure 2/2A/k.tif]

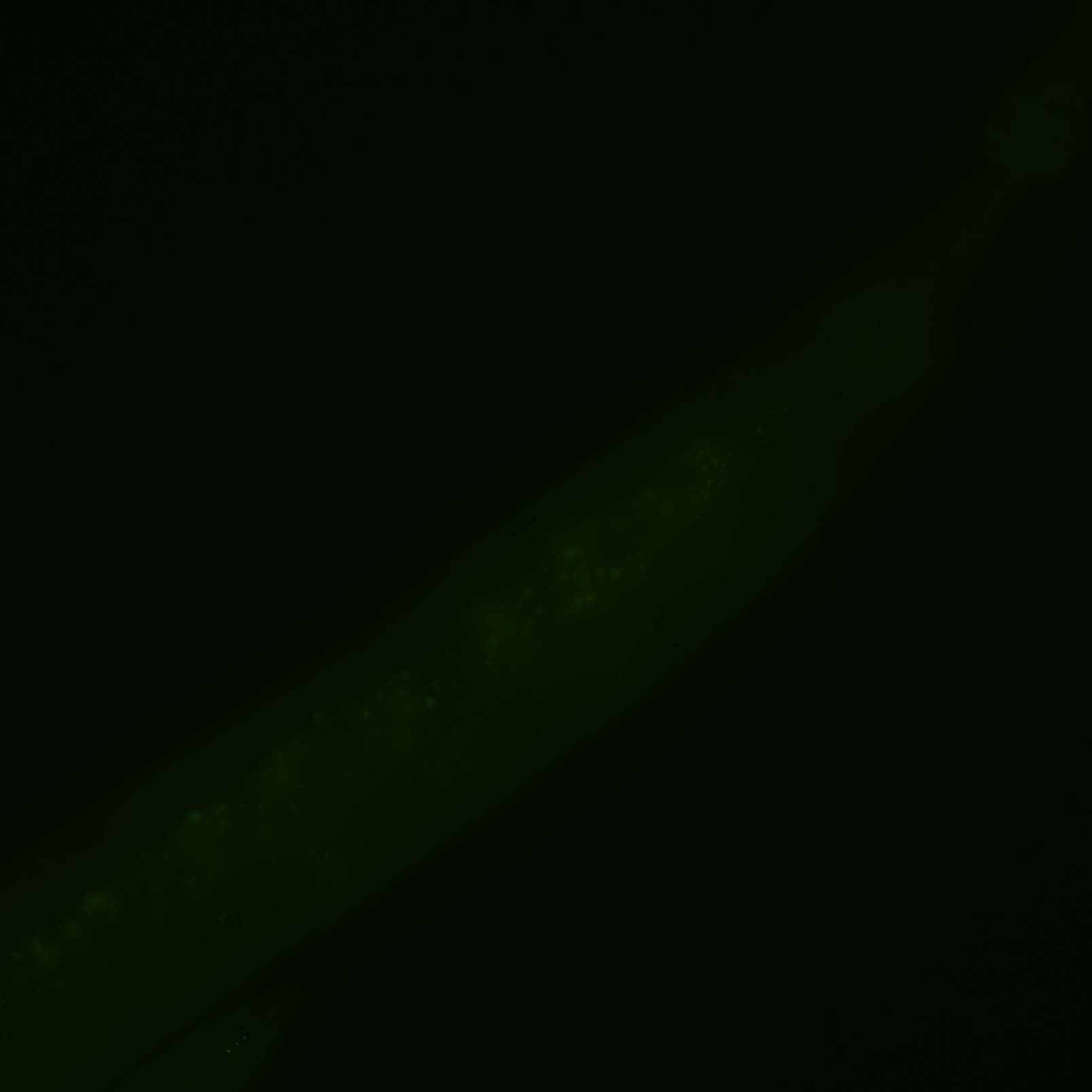

Supplement: Supplementary file 5 — Source data Fig. 2 [file 44318_2025_619_MOESM5_ESM.zip › Figure 2/2A/l.tif]

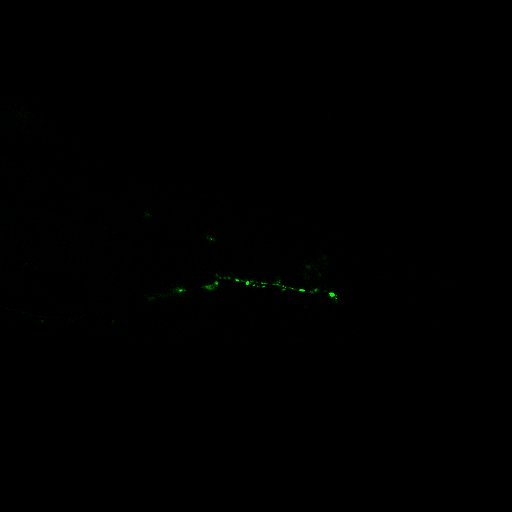

Supplement: Supplementary file 5 — Source data Fig. 2 [file 44318_2025_619_MOESM5_ESM.zip › Figure 2/2C/a.tif]

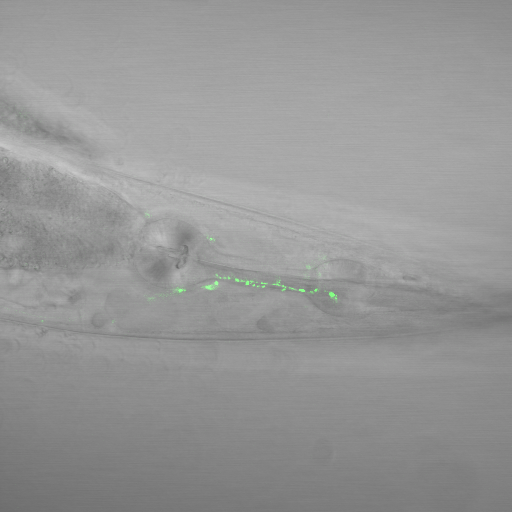

Supplement: Supplementary file 5 — Source data Fig. 2 [file 44318_2025_619_MOESM5_ESM.zip › Figure 2/2C/b.tif]

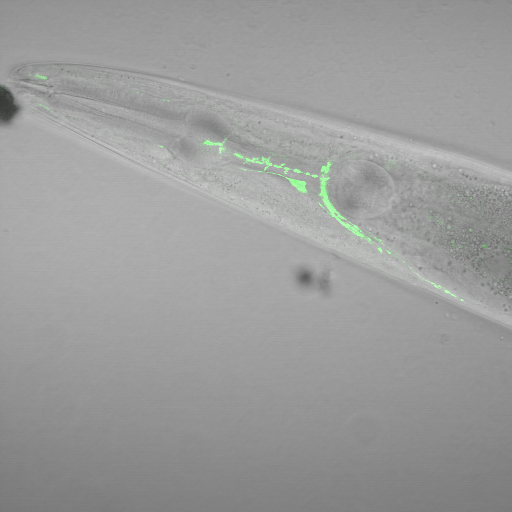

Supplement: Supplementary file 5 — Source data Fig. 2 [file 44318_2025_619_MOESM5_ESM.zip › Figure 2/2C/c.tif]

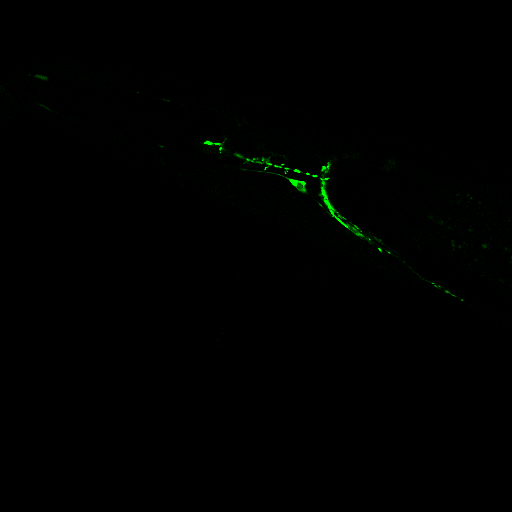

Supplement: Supplementary file 5 — Source data Fig. 2 [file 44318_2025_619_MOESM5_ESM.zip › Figure 2/2C/d.tif]

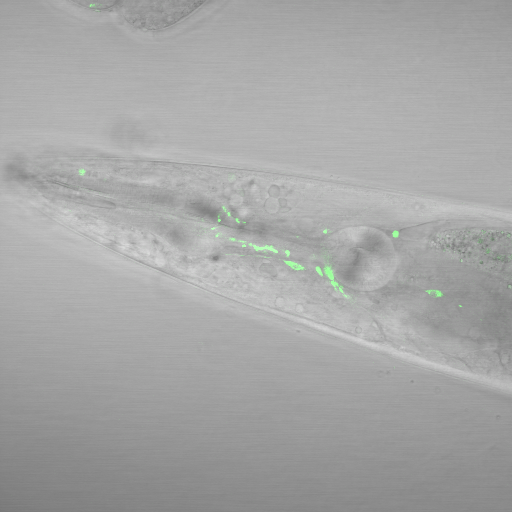

Supplement: Supplementary file 5 — Source data Fig. 2 [file 44318_2025_619_MOESM5_ESM.zip › Figure 2/2C/e.tif]

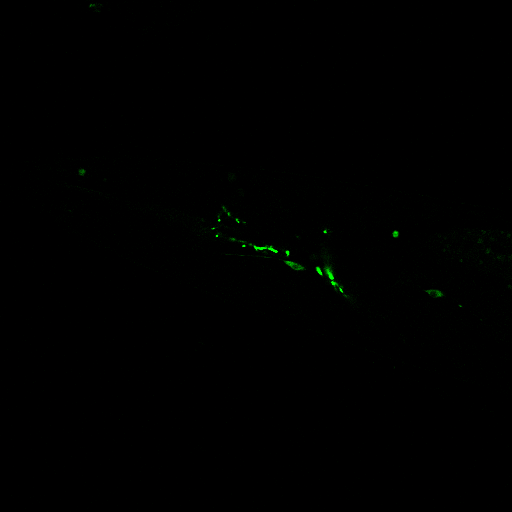

Supplement: Supplementary file 5 — Source data Fig. 2 [file 44318_2025_619_MOESM5_ESM.zip › Figure 2/2C/f.tif]

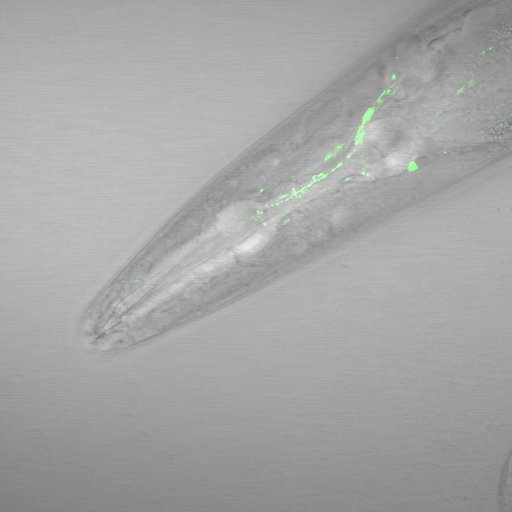

Supplement: Supplementary file 5 — Source data Fig. 2 [file 44318_2025_619_MOESM5_ESM.zip › Figure 2/2C/g.tif]

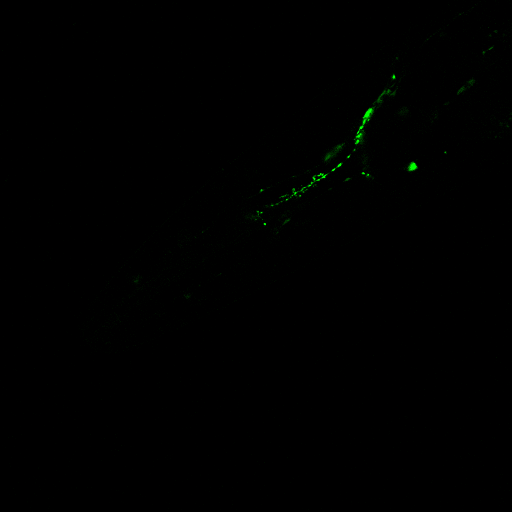

Supplement: Supplementary file 5 — Source data Fig. 2 [file 44318_2025_619_MOESM5_ESM.zip › Figure 2/2C/h.tif]

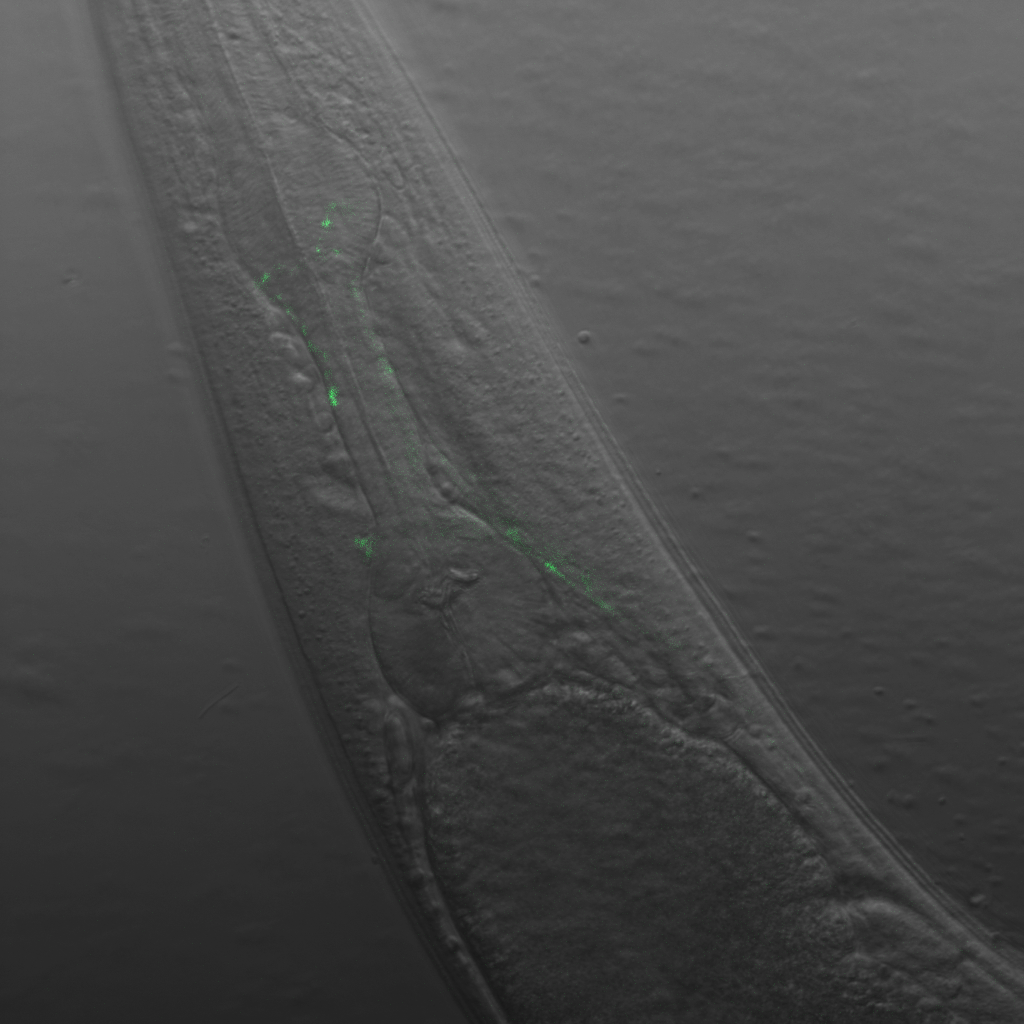

Supplement: Supplementary file 5 — Source data Fig. 2 [file 44318_2025_619_MOESM5_ESM.zip › Figure 2/2E/a.tif]

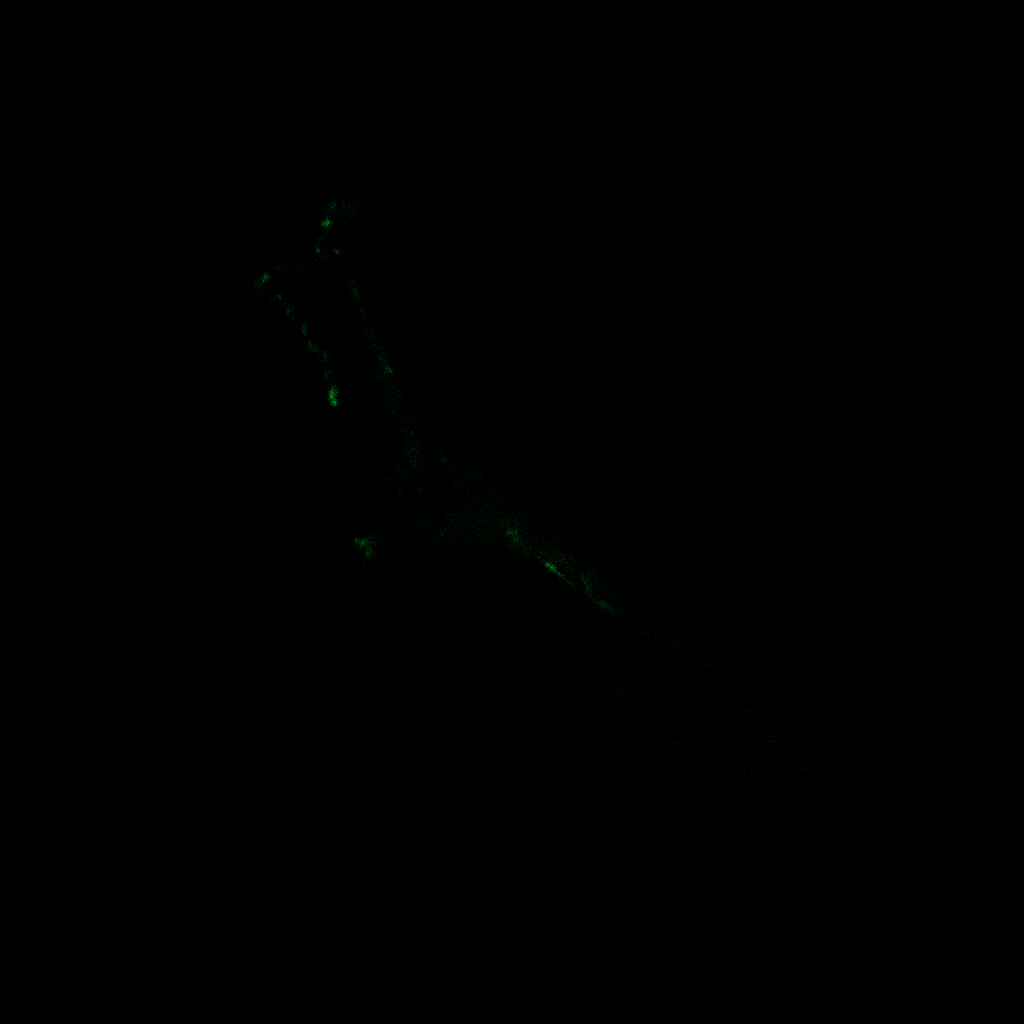

Supplement: Supplementary file 5 — Source data Fig. 2 [file 44318_2025_619_MOESM5_ESM.zip › Figure 2/2E/b.tif]

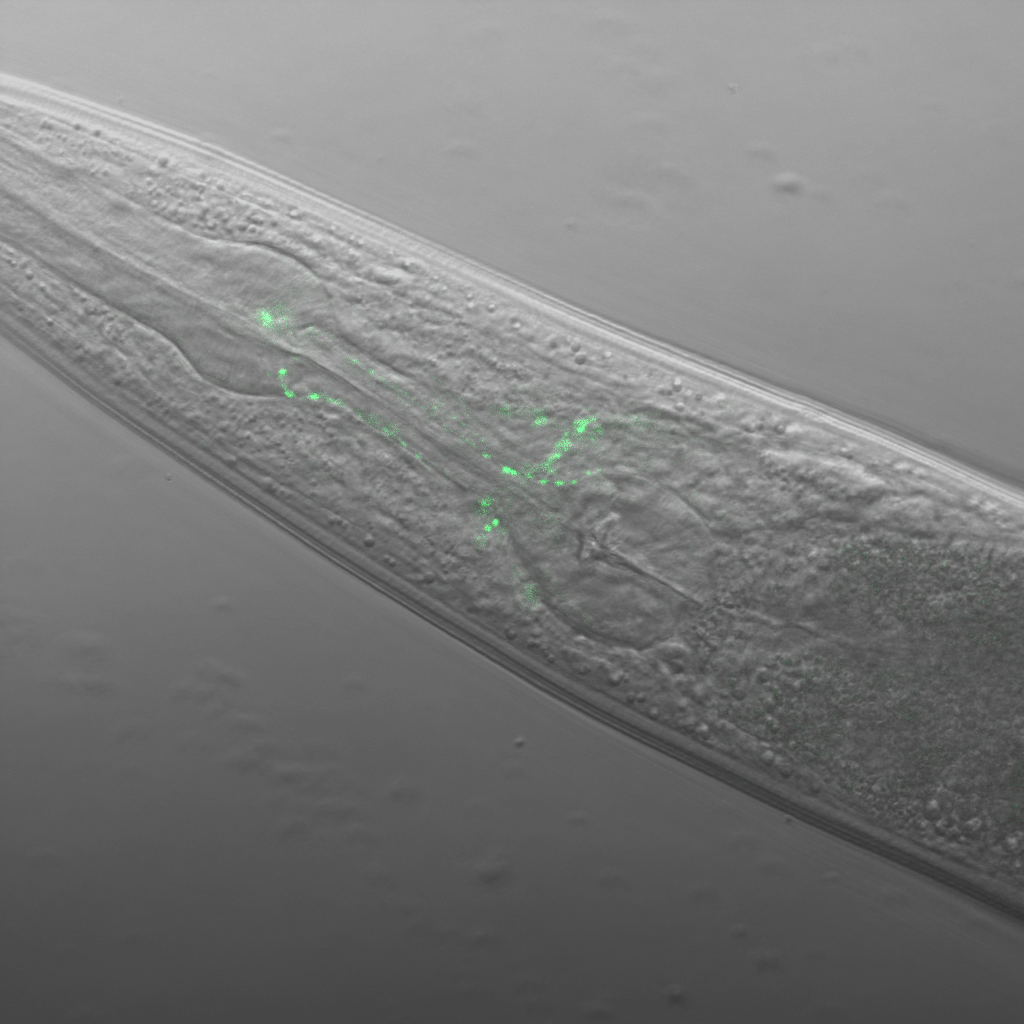

Supplement: Supplementary file 5 — Source data Fig. 2 [file 44318_2025_619_MOESM5_ESM.zip › Figure 2/2E/c.tif]

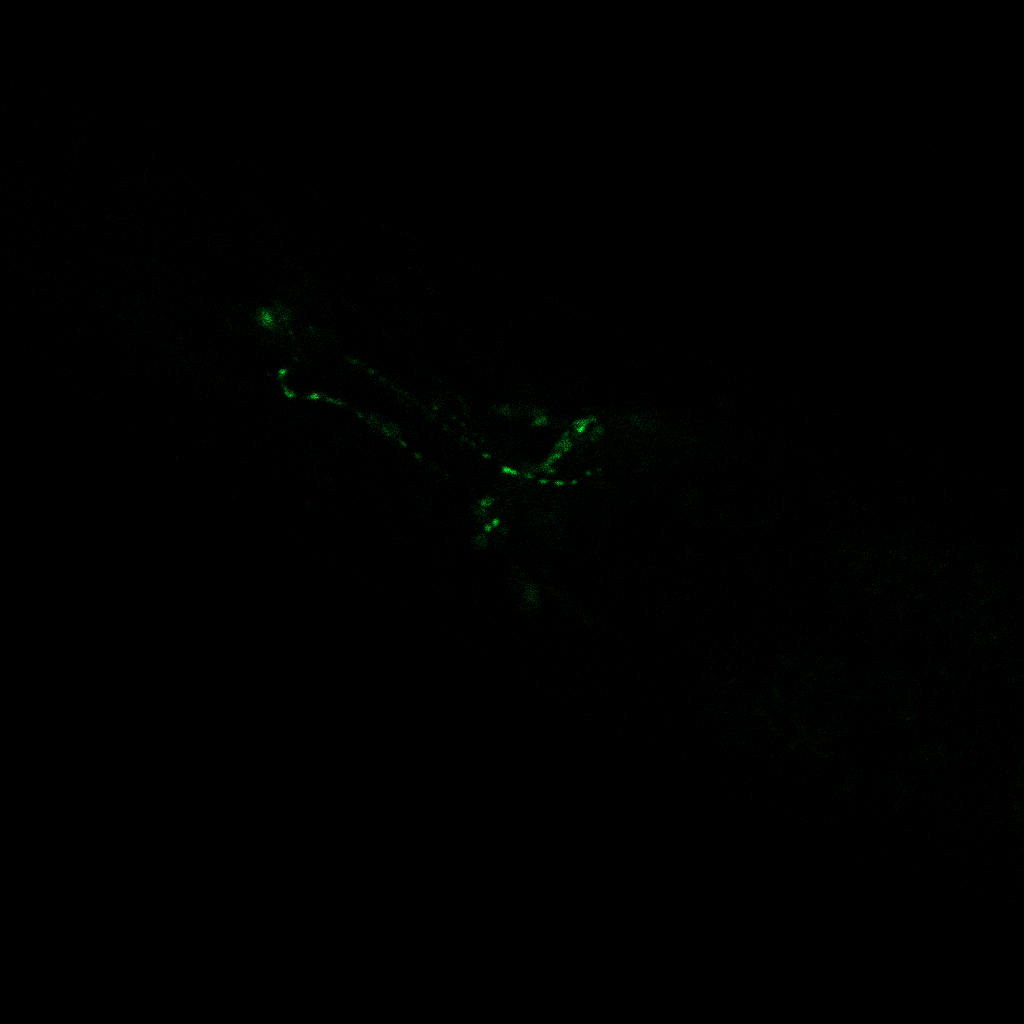

Supplement: Supplementary file 5 — Source data Fig. 2 [file 44318_2025_619_MOESM5_ESM.zip › Figure 2/2E/d.tif]

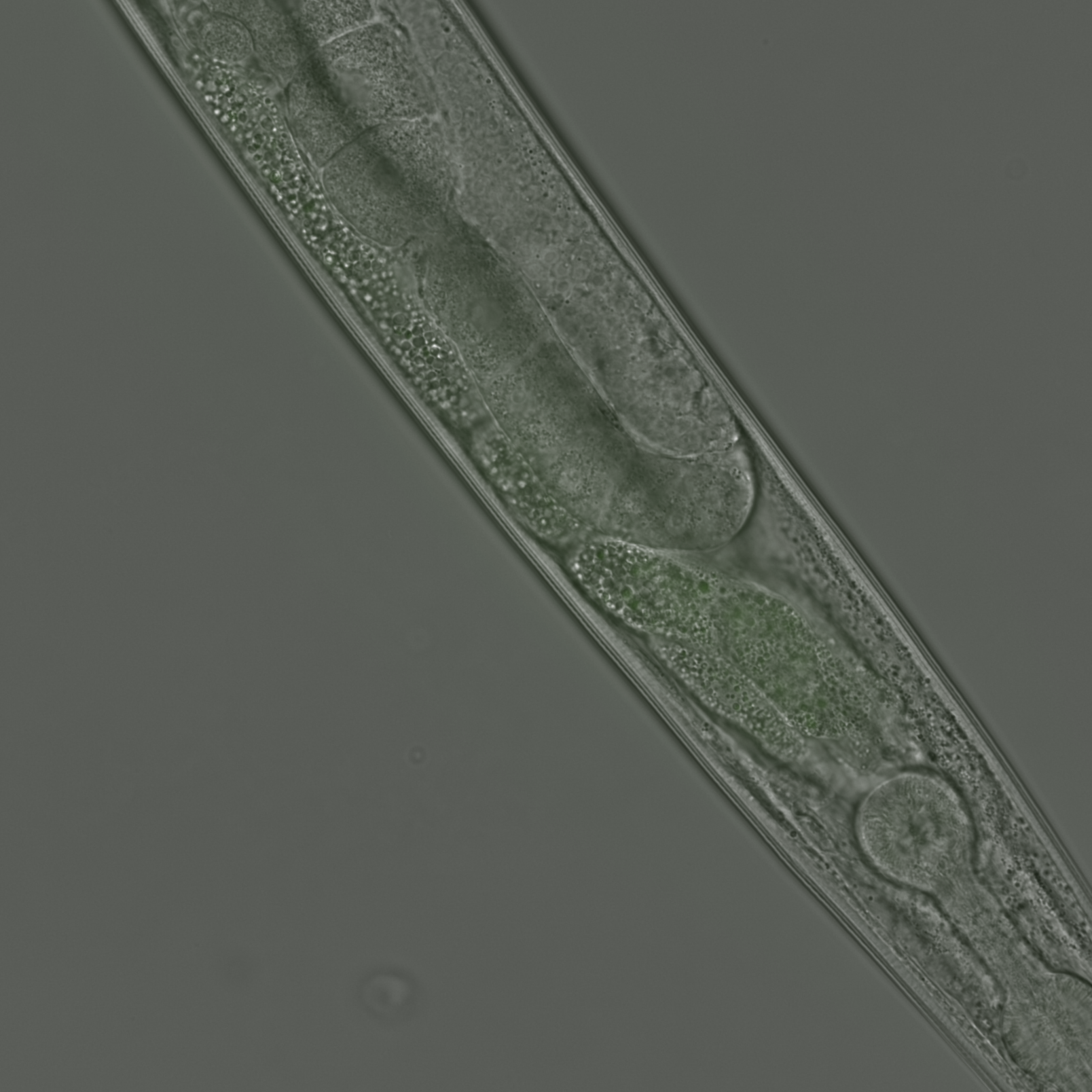

Supplement: Supplementary file 6 — Source data Fig. 3 [file 44318_2025_619_MOESM6_ESM.zip › Figure 3/3A/a.tif]

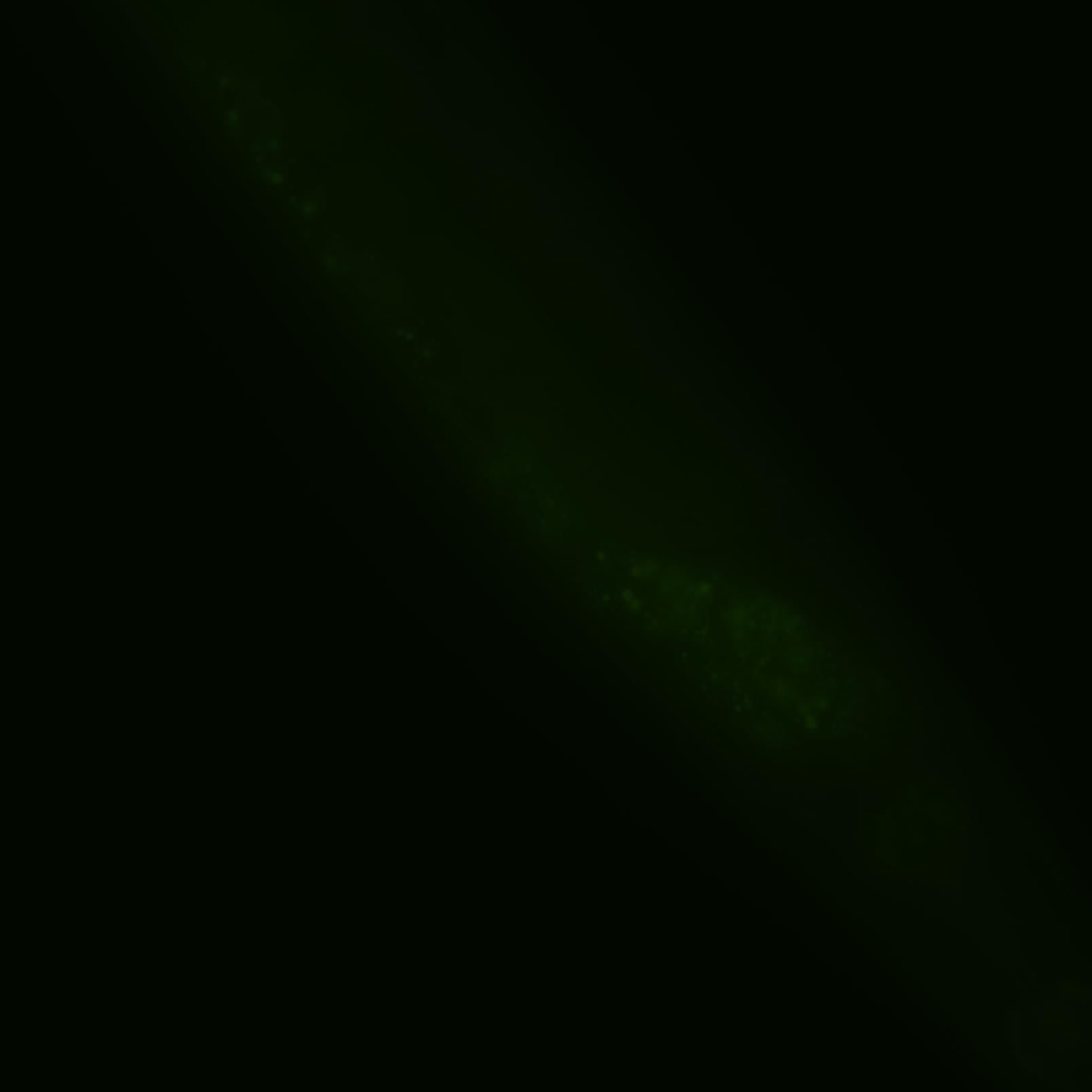

Supplement: Supplementary file 6 — Source data Fig. 3 [file 44318_2025_619_MOESM6_ESM.zip › Figure 3/3A/b.tif]

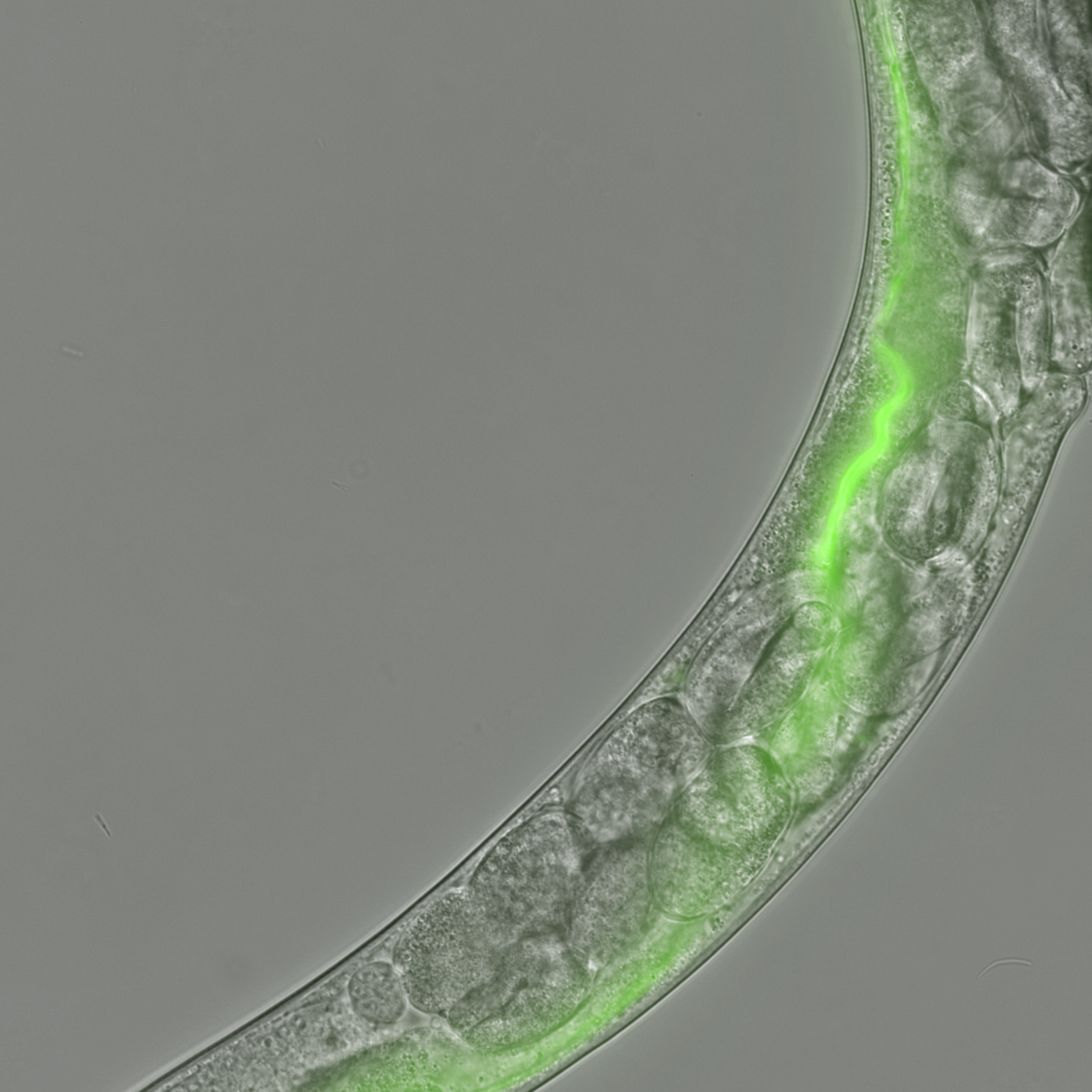

Supplement: Supplementary file 6 — Source data Fig. 3 [file 44318_2025_619_MOESM6_ESM.zip › Figure 3/3A/c.tif]

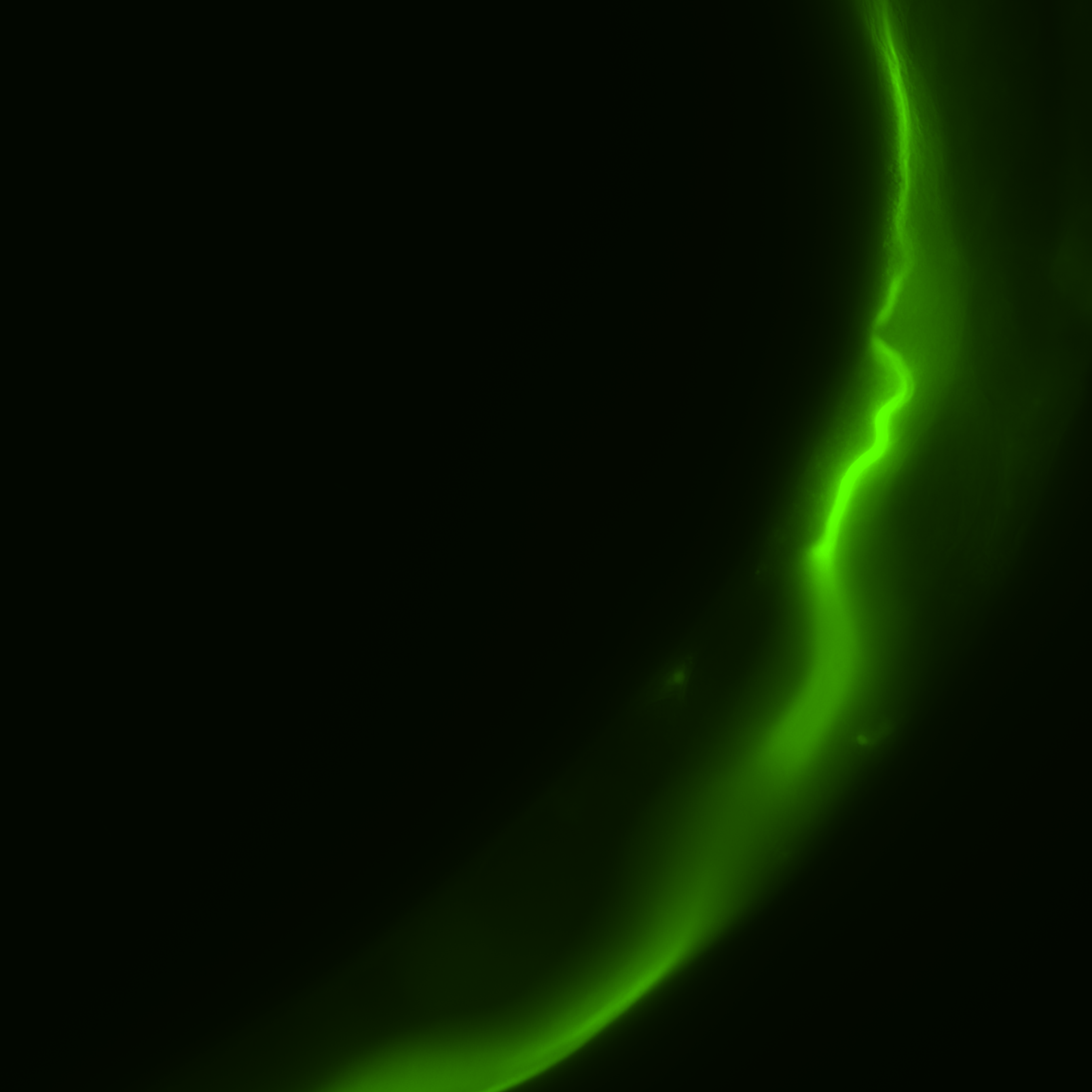

Supplement: Supplementary file 6 — Source data Fig. 3 [file 44318_2025_619_MOESM6_ESM.zip › Figure 3/3A/d.tif]

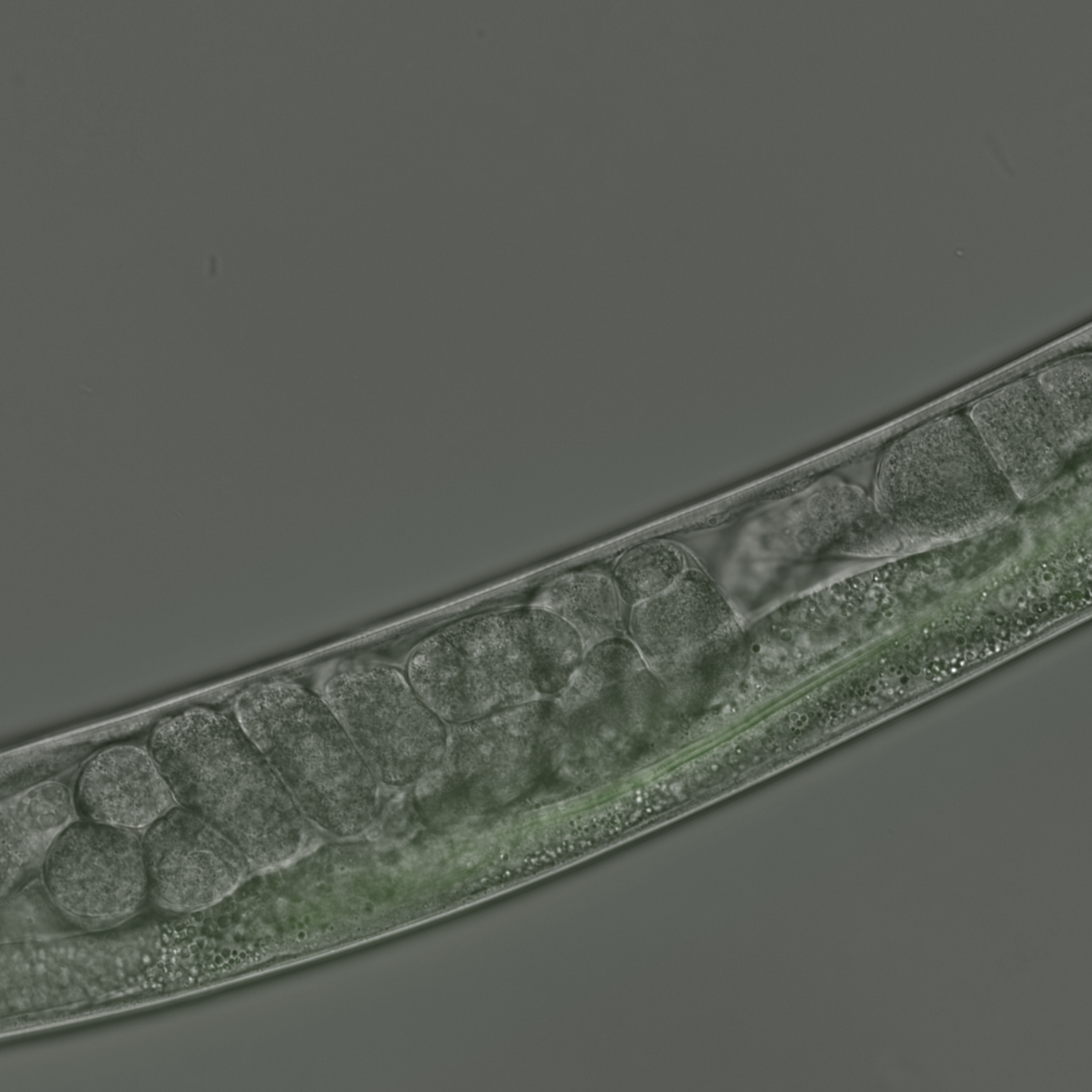

Supplement: Supplementary file 6 — Source data Fig. 3 [file 44318_2025_619_MOESM6_ESM.zip › Figure 3/3A/e.tif]

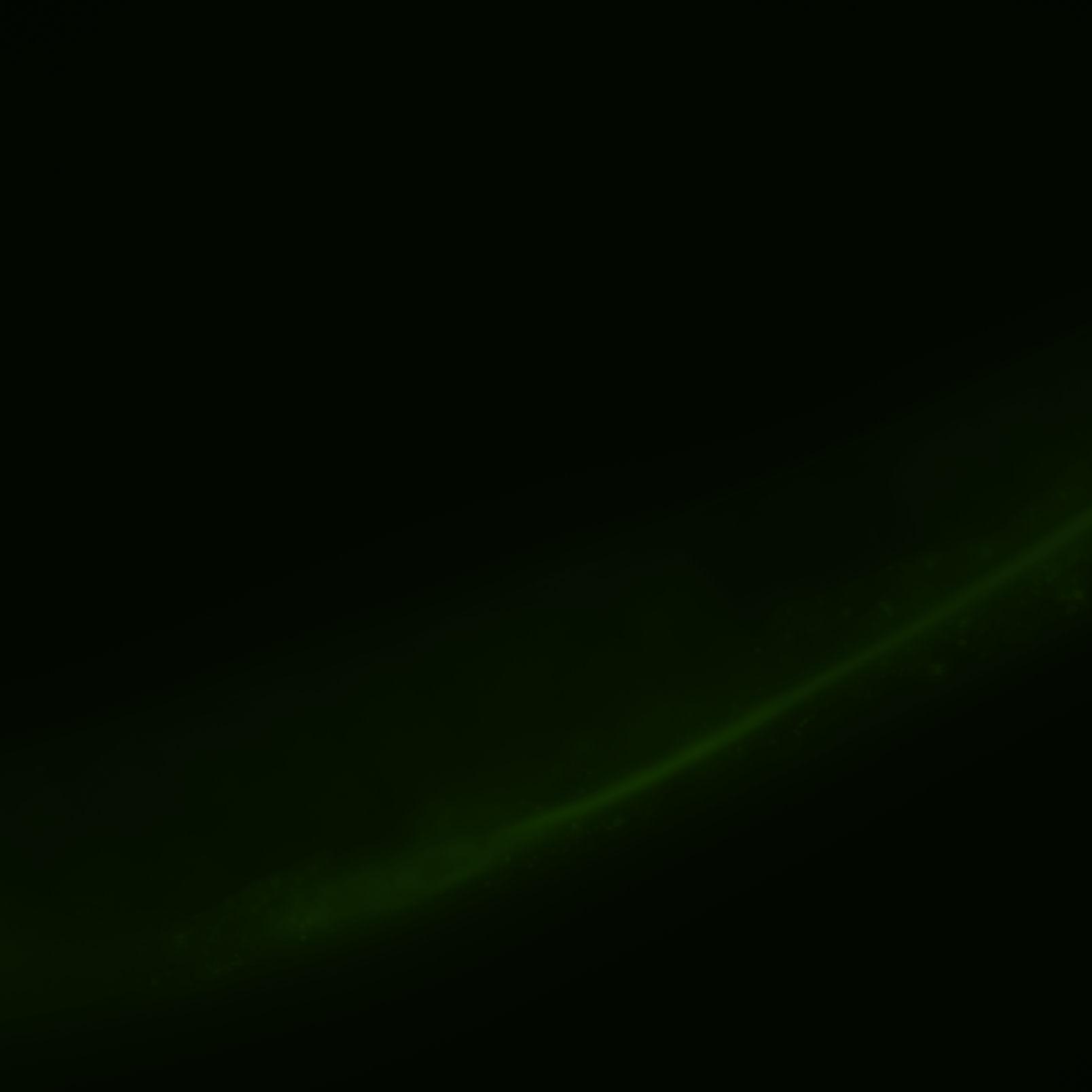

Supplement: Supplementary file 6 — Source data Fig. 3 [file 44318_2025_619_MOESM6_ESM.zip › Figure 3/3A/f.tif]

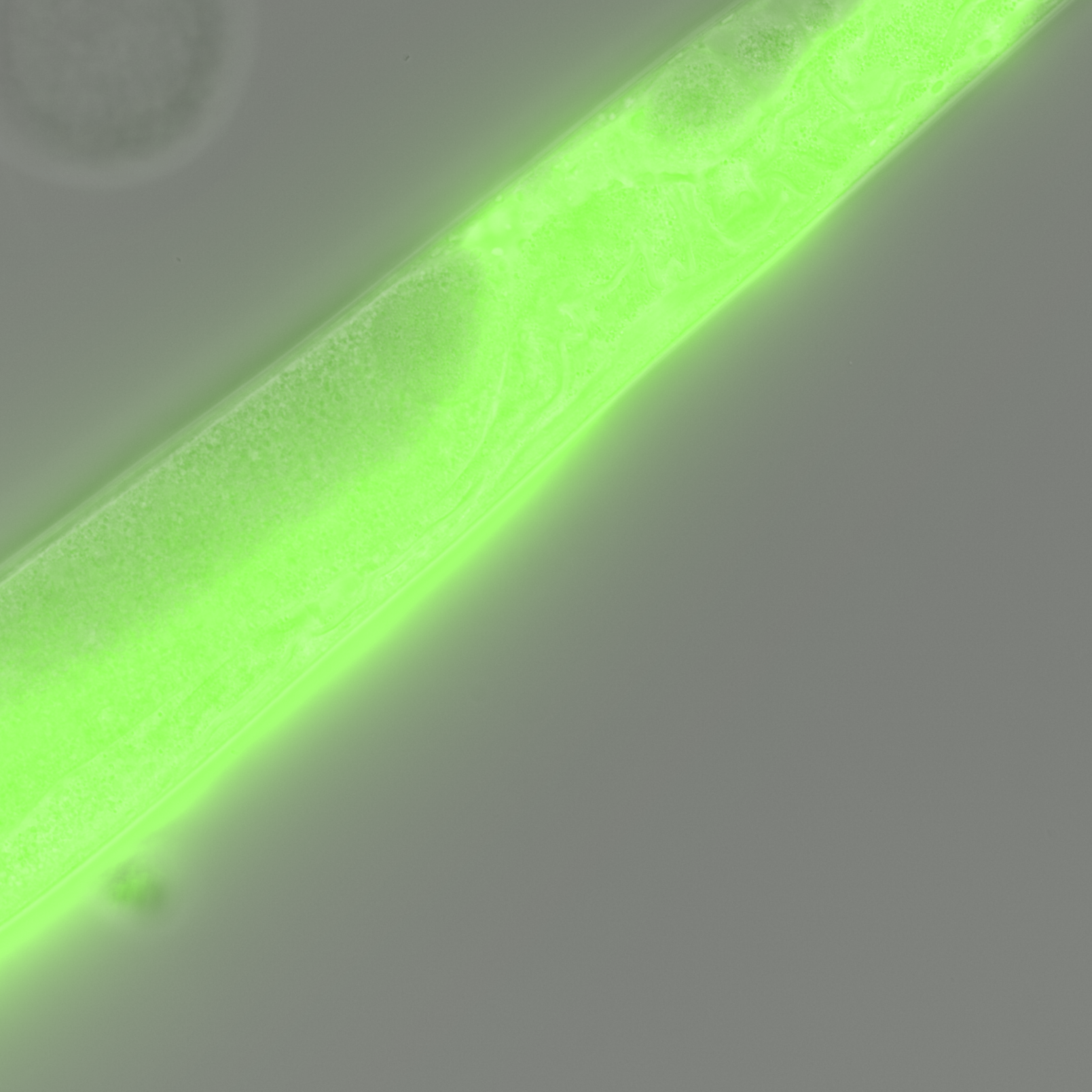

Supplement: Supplementary file 6 — Source data Fig. 3 [file 44318_2025_619_MOESM6_ESM.zip › Figure 3/3A/g.tif]

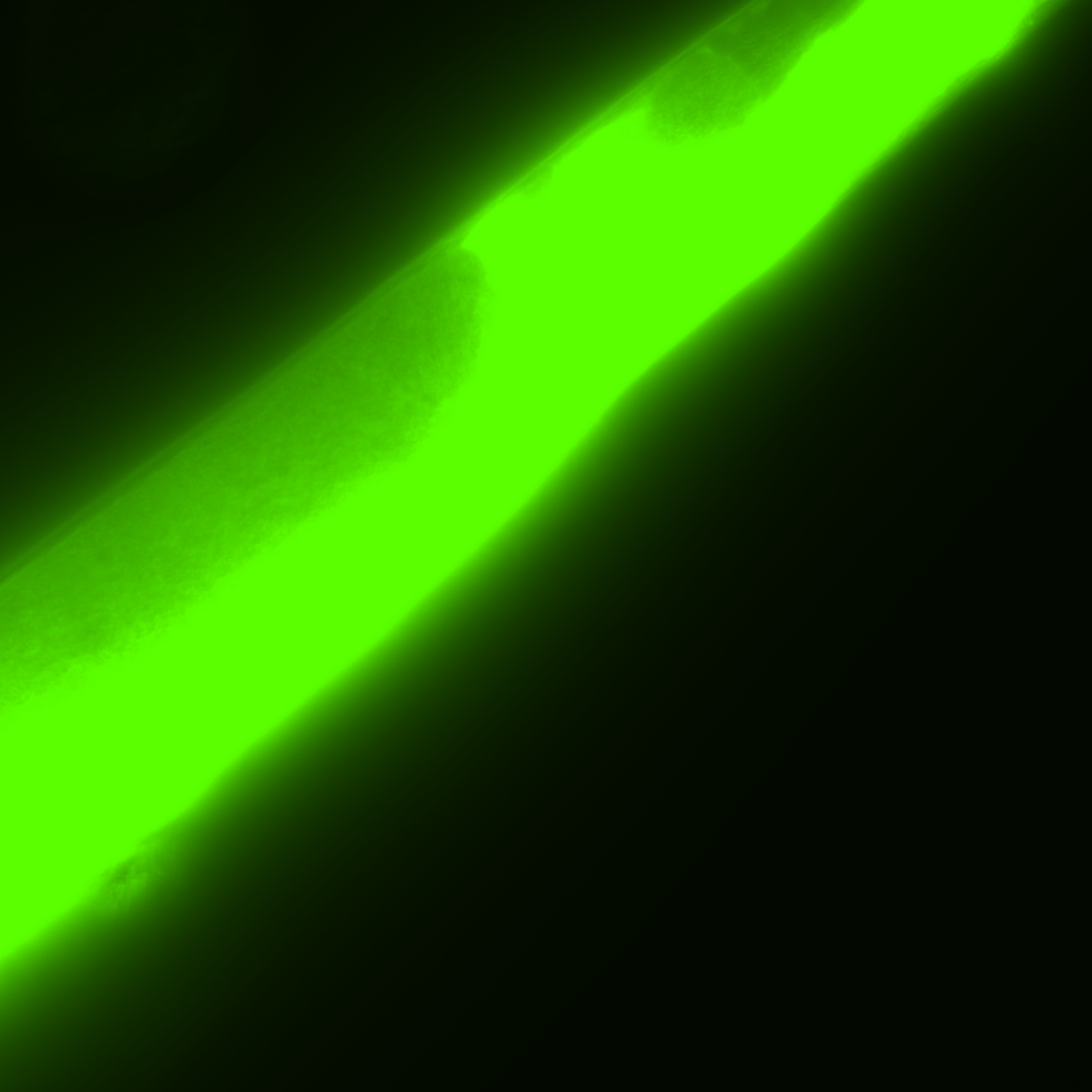

Supplement: Supplementary file 6 — Source data Fig. 3 [file 44318_2025_619_MOESM6_ESM.zip › Figure 3/3A/h.tif]

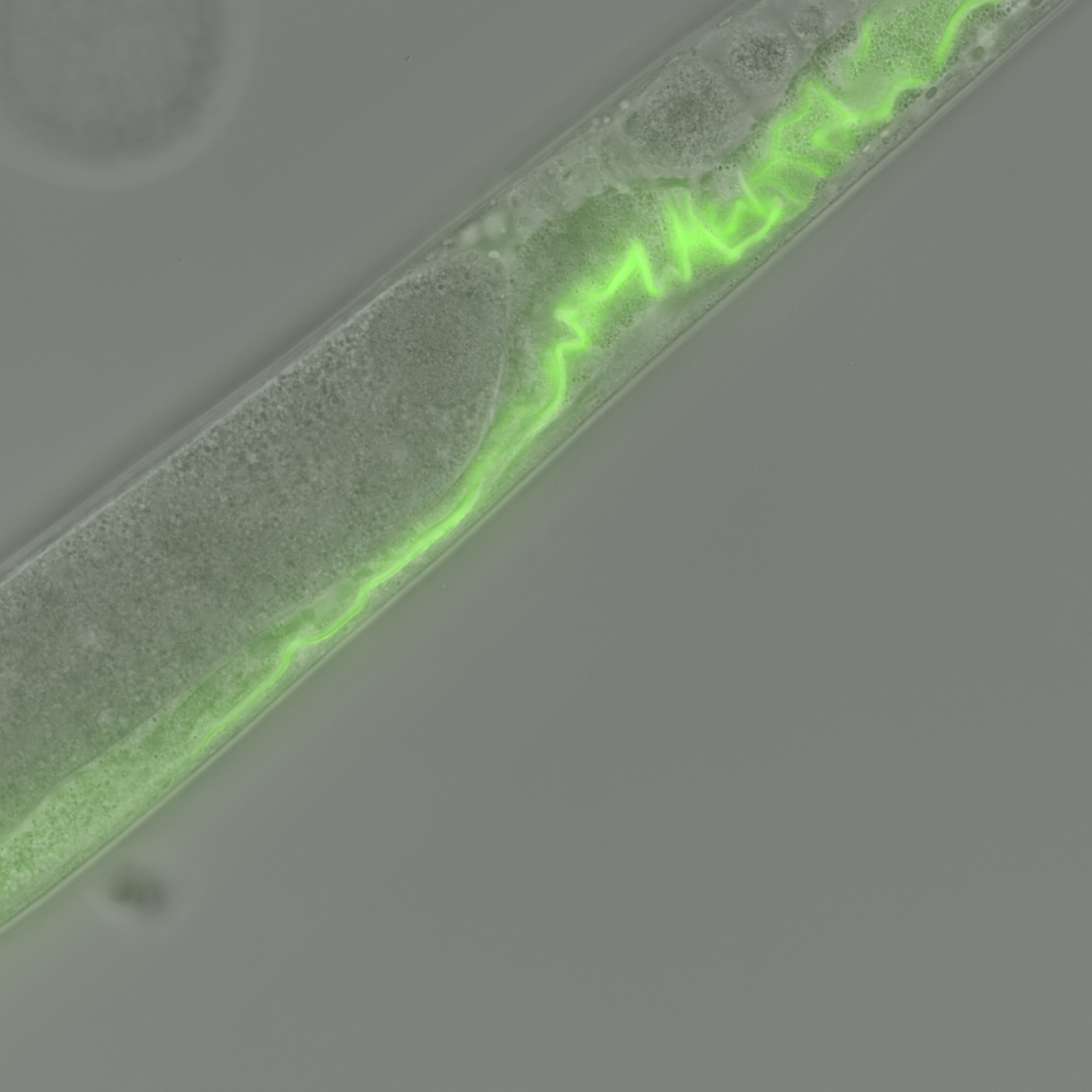

Supplement: Supplementary file 6 — Source data Fig. 3 [file 44318_2025_619_MOESM6_ESM.zip › Figure 3/3A/i.tif]

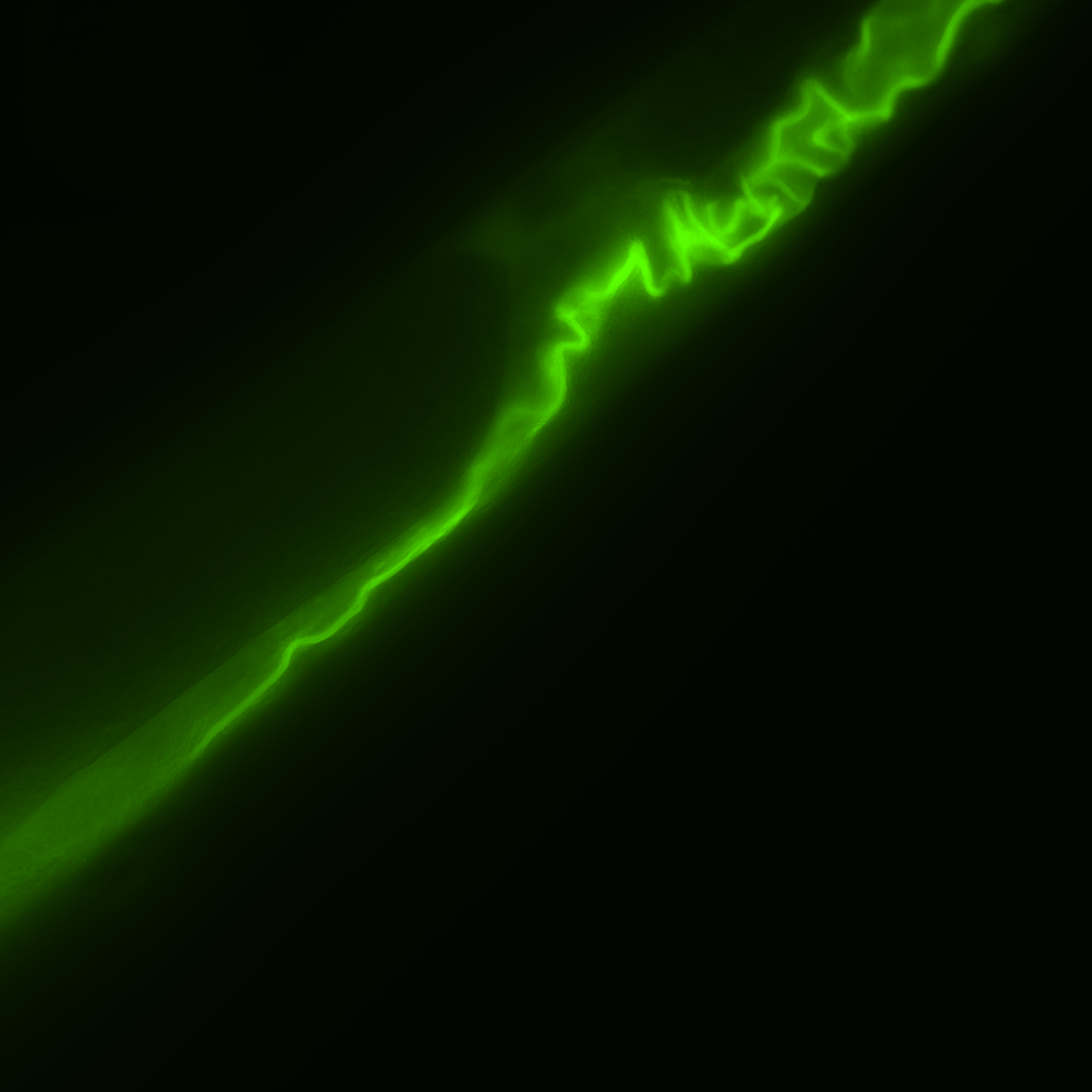

Supplement: Supplementary file 6 — Source data Fig. 3 [file 44318_2025_619_MOESM6_ESM.zip › Figure 3/3A/j.tif]

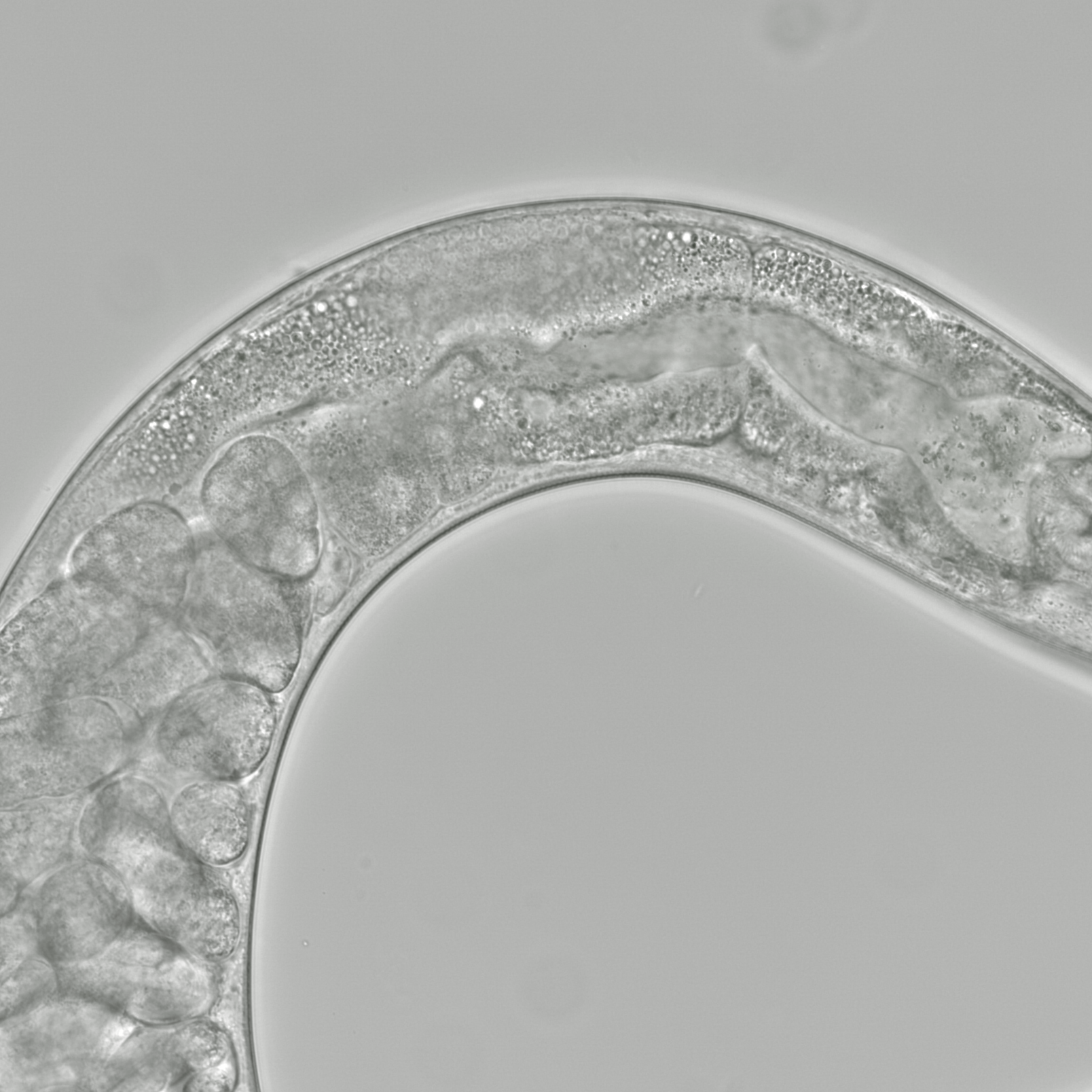

Supplement: Supplementary file 6 — Source data Fig. 3 [file 44318_2025_619_MOESM6_ESM.zip › Figure 3/3C/a.tif]

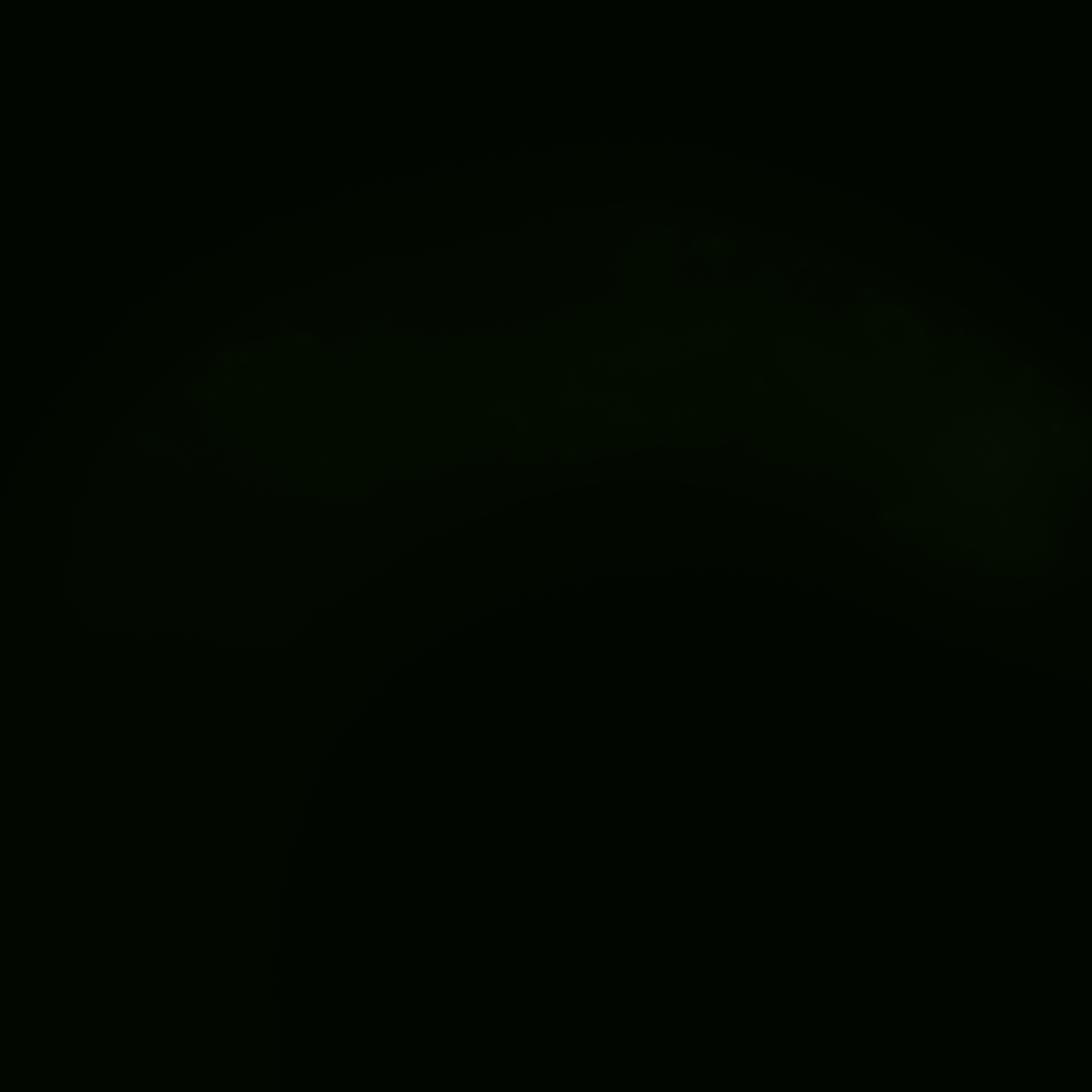

Supplement: Supplementary file 6 — Source data Fig. 3 [file 44318_2025_619_MOESM6_ESM.zip › Figure 3/3C/b.tif]

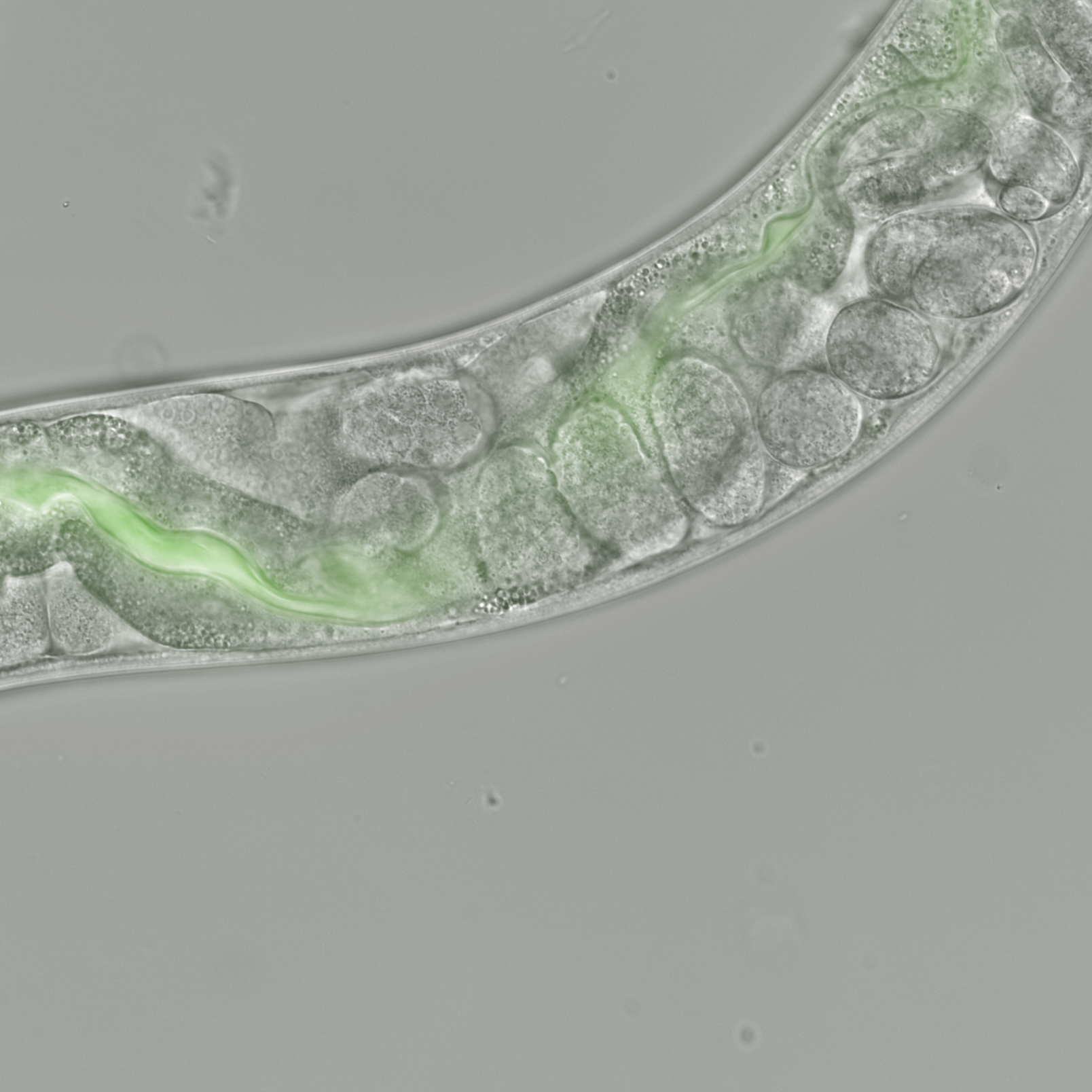

Supplement: Supplementary file 6 — Source data Fig. 3 [file 44318_2025_619_MOESM6_ESM.zip › Figure 3/3C/c.tif]

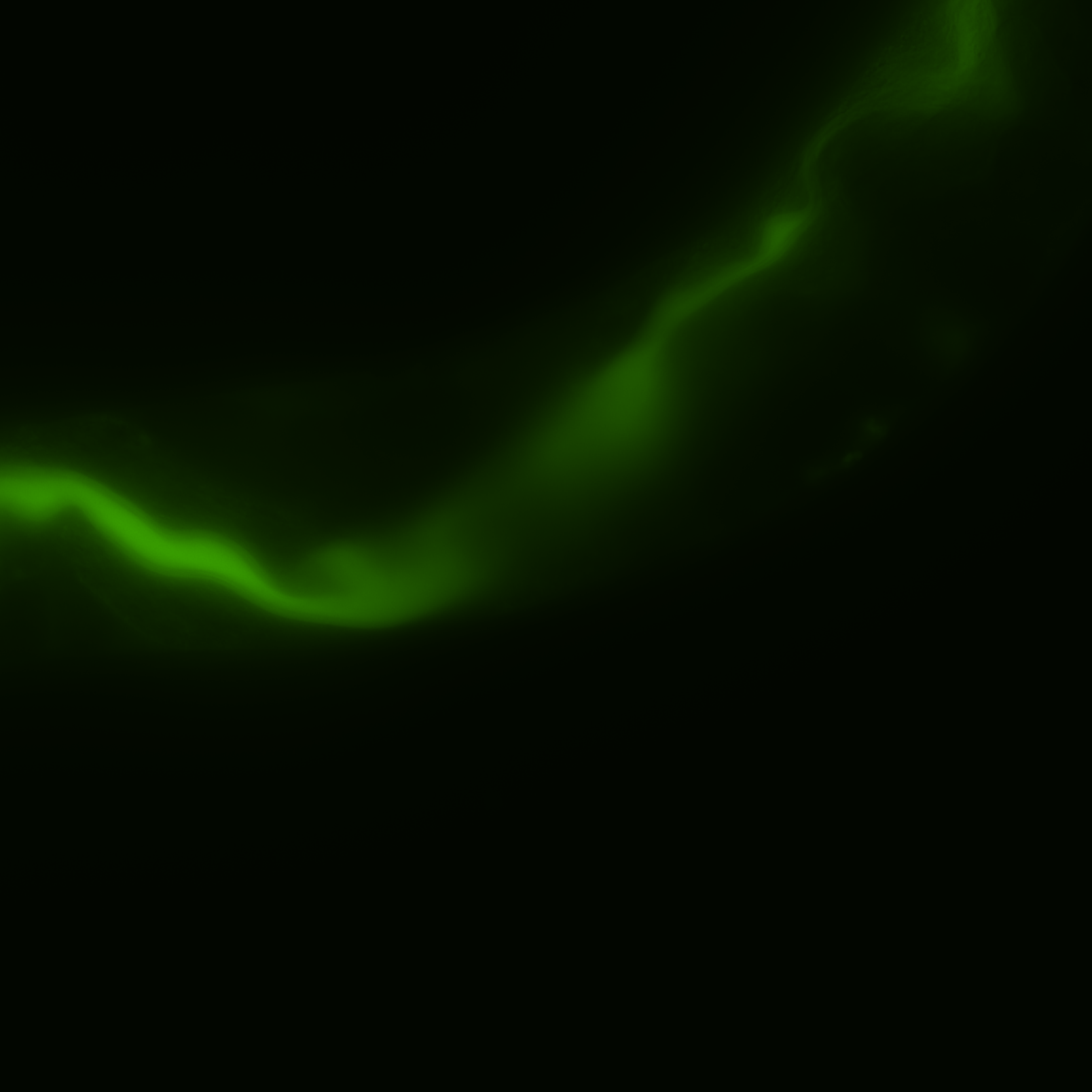

Supplement: Supplementary file 6 — Source data Fig. 3 [file 44318_2025_619_MOESM6_ESM.zip › Figure 3/3C/d.tif]

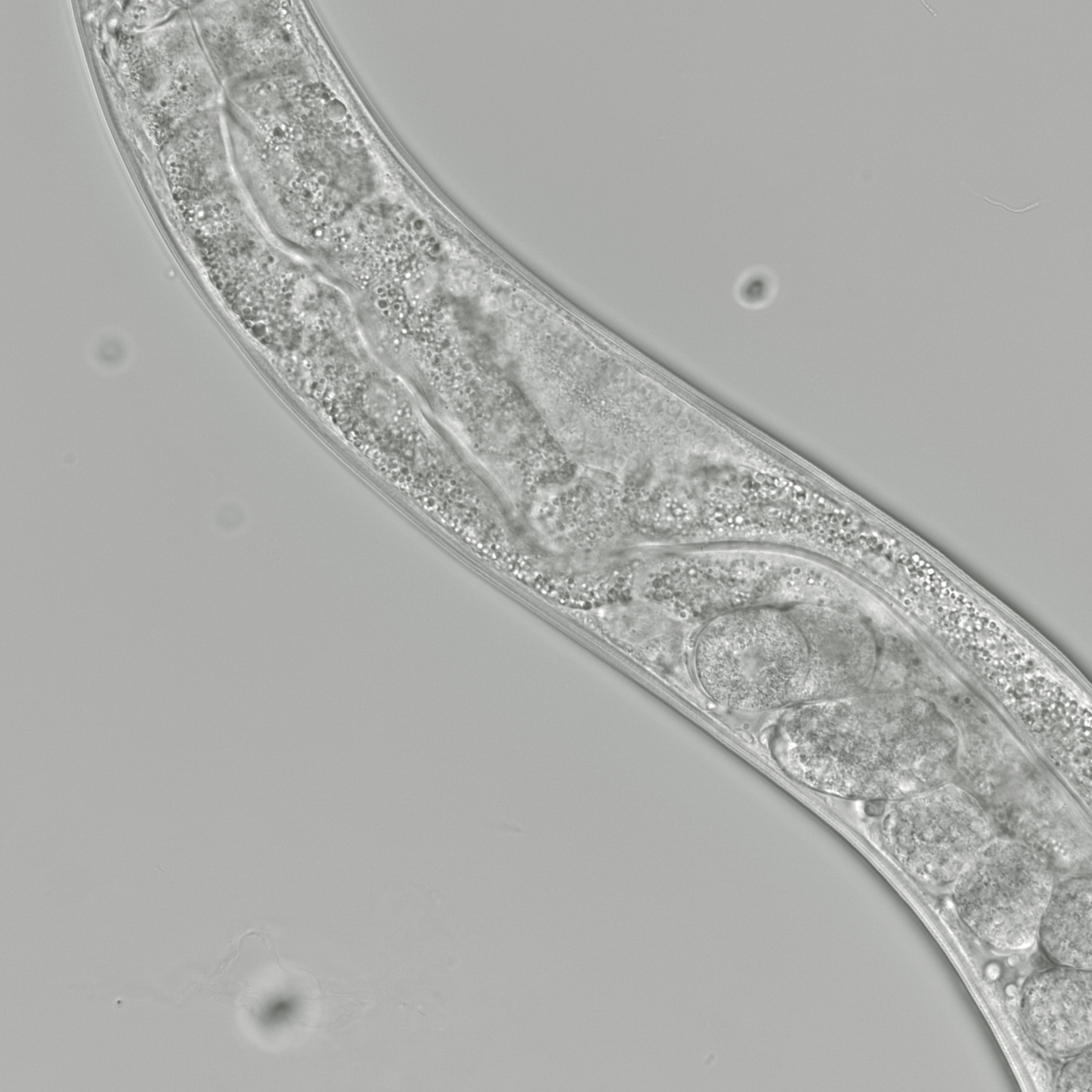

Supplement: Supplementary file 6 — Source data Fig. 3 [file 44318_2025_619_MOESM6_ESM.zip › Figure 3/3C/e.tif]

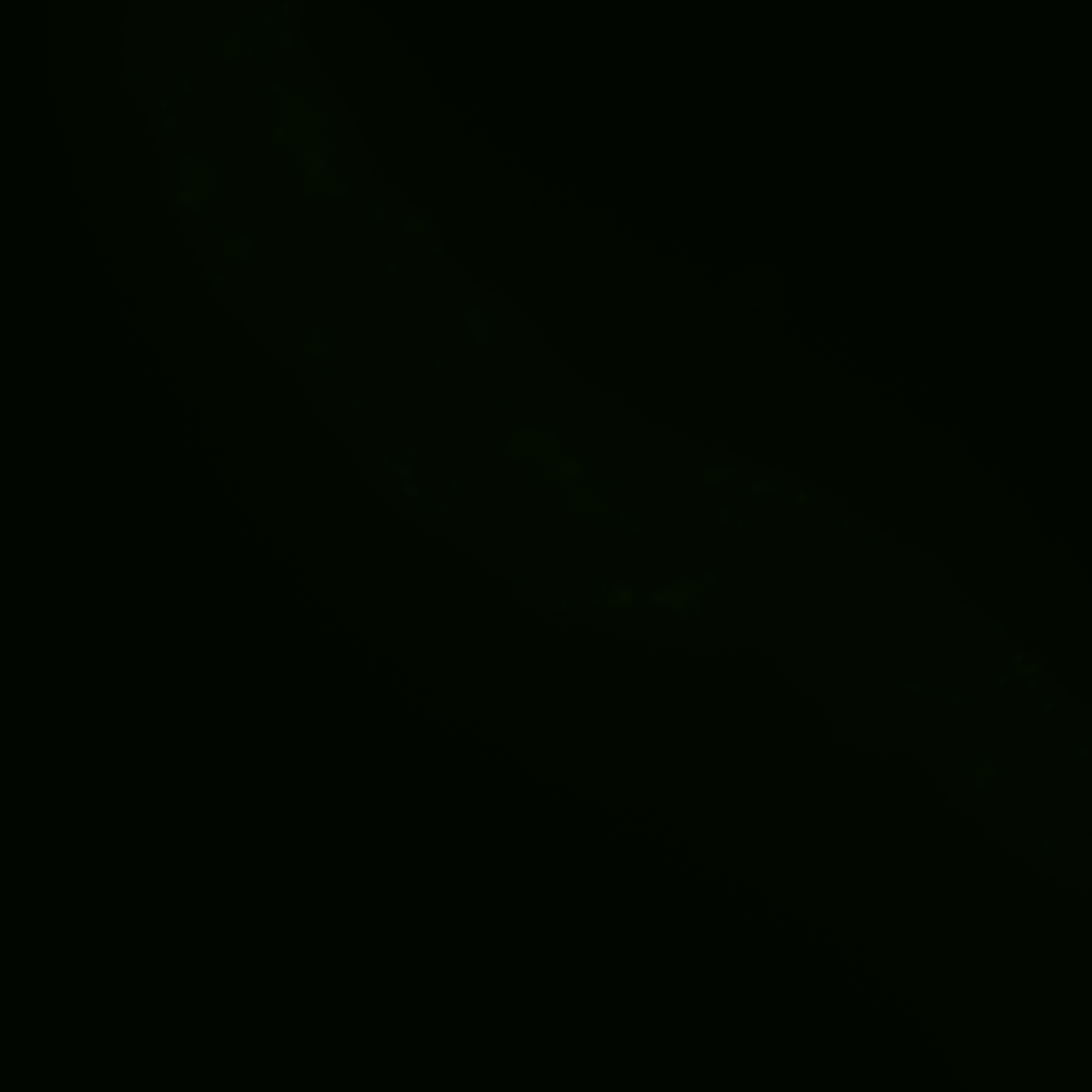

Supplement: Supplementary file 6 — Source data Fig. 3 [file 44318_2025_619_MOESM6_ESM.zip › Figure 3/3C/f.tif]

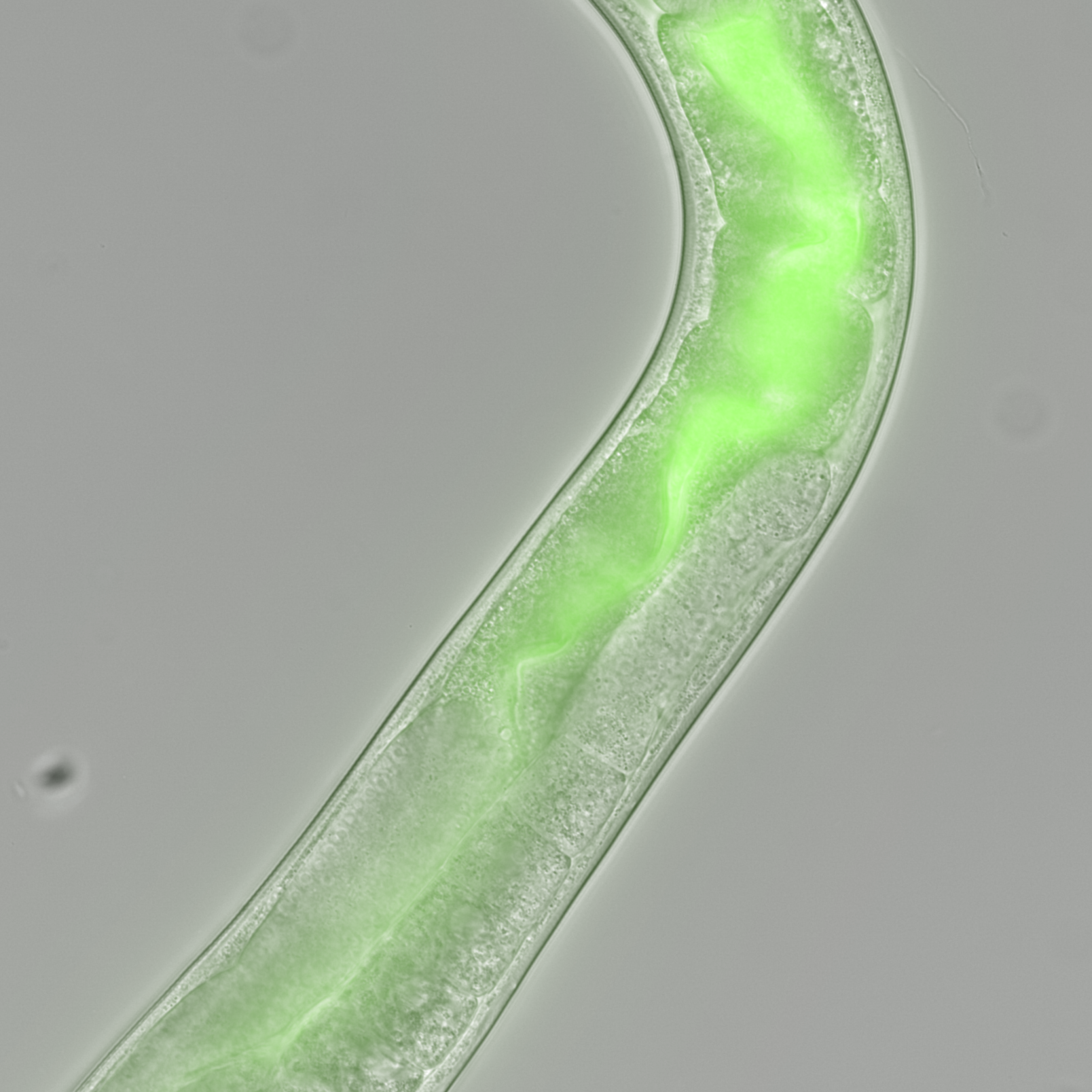

Supplement: Supplementary file 6 — Source data Fig. 3 [file 44318_2025_619_MOESM6_ESM.zip › Figure 3/3C/g.tif]

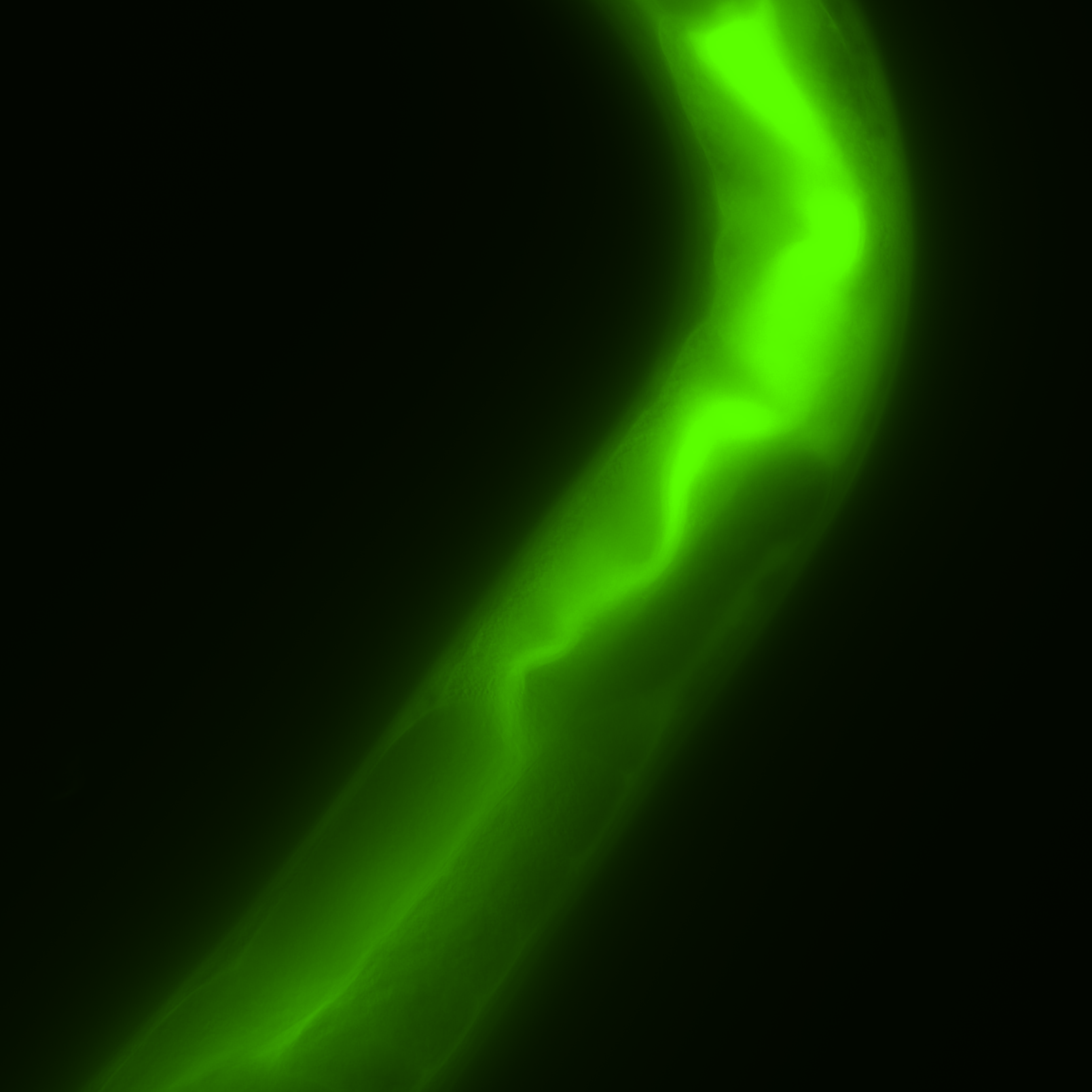

Supplement: Supplementary file 6 — Source data Fig. 3 [file 44318_2025_619_MOESM6_ESM.zip › Figure 3/3C/h.tif]

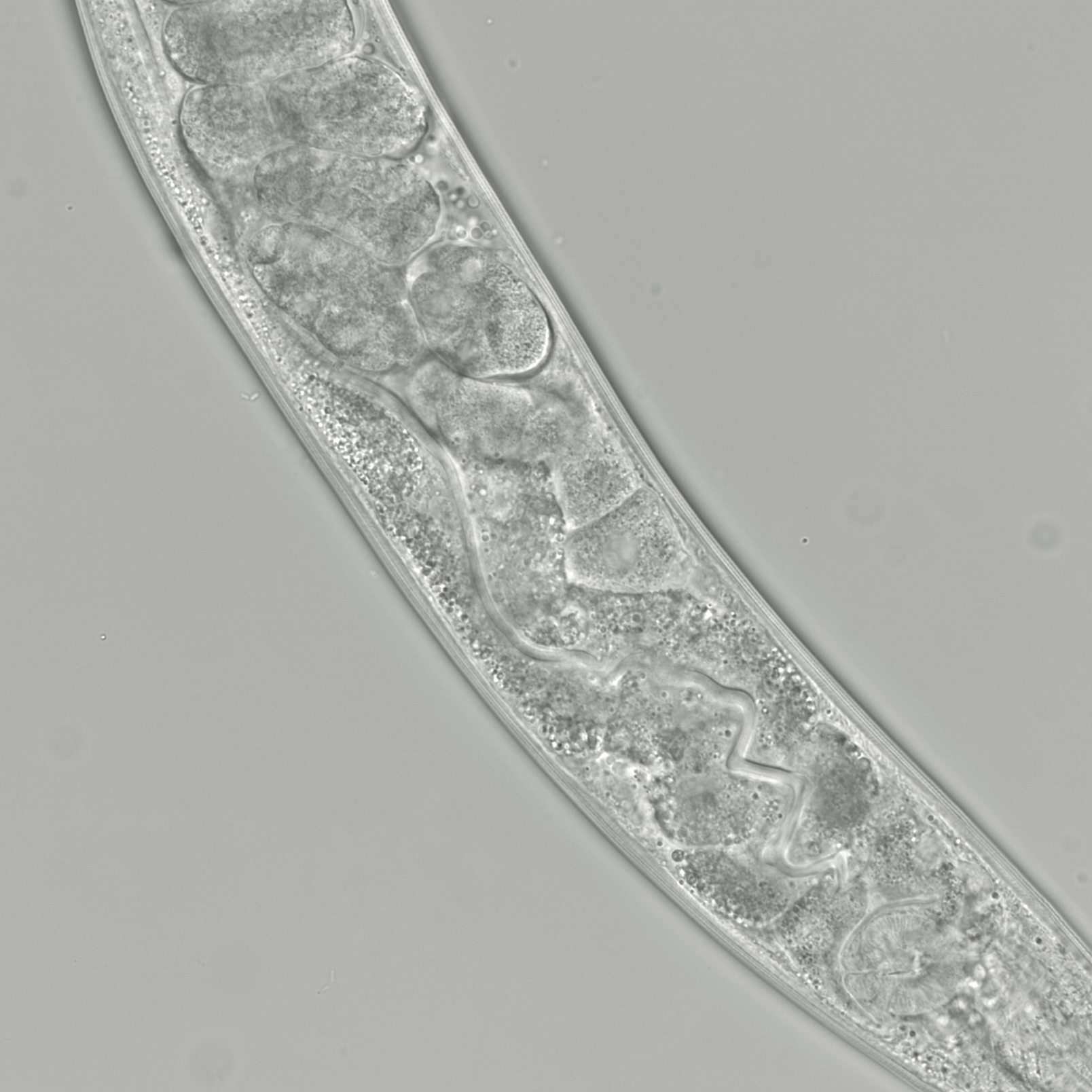

Supplement: Supplementary file 6 — Source data Fig. 3 [file 44318_2025_619_MOESM6_ESM.zip › Figure 3/3E/a.tif]

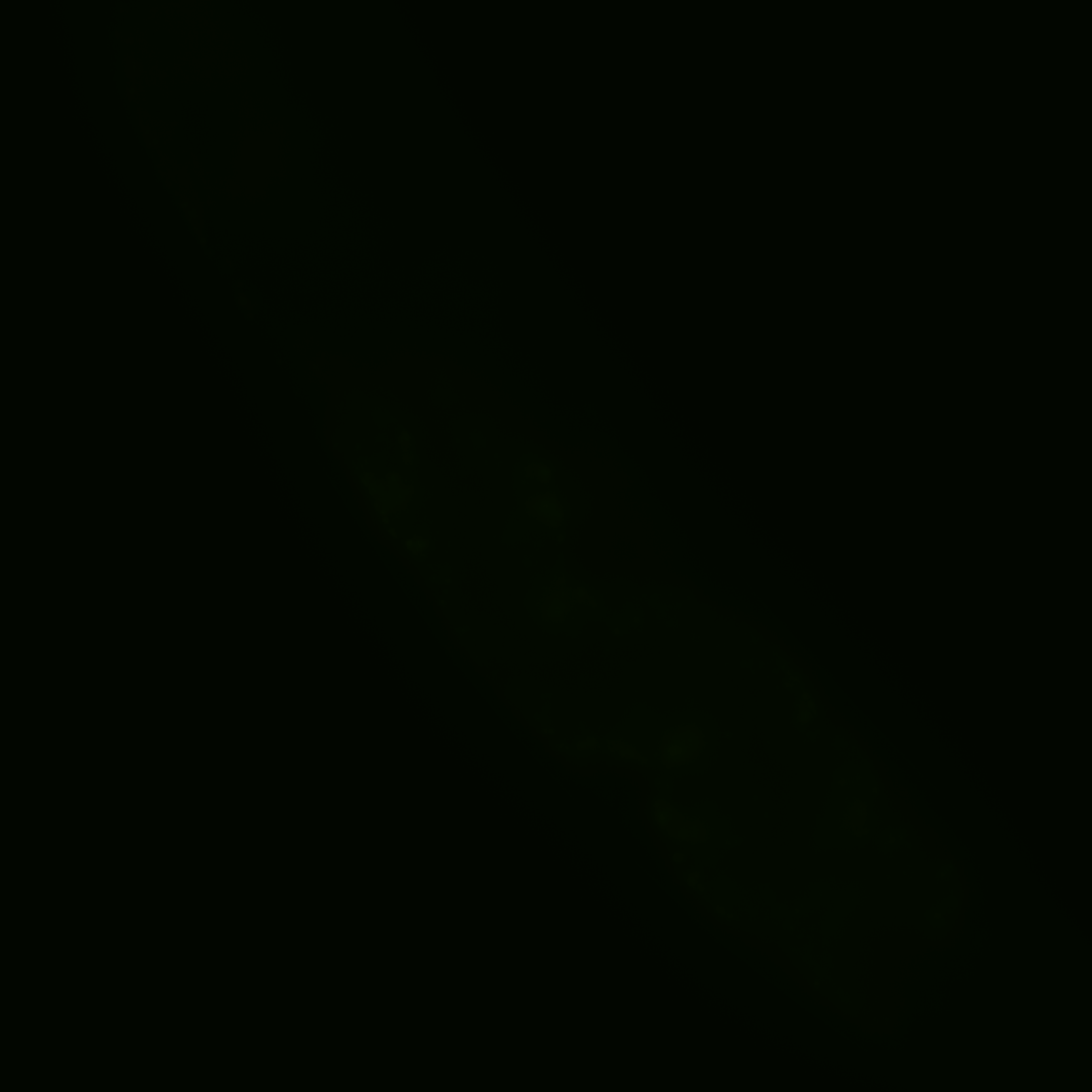

Supplement: Supplementary file 6 — Source data Fig. 3 [file 44318_2025_619_MOESM6_ESM.zip › Figure 3/3E/b.tif]

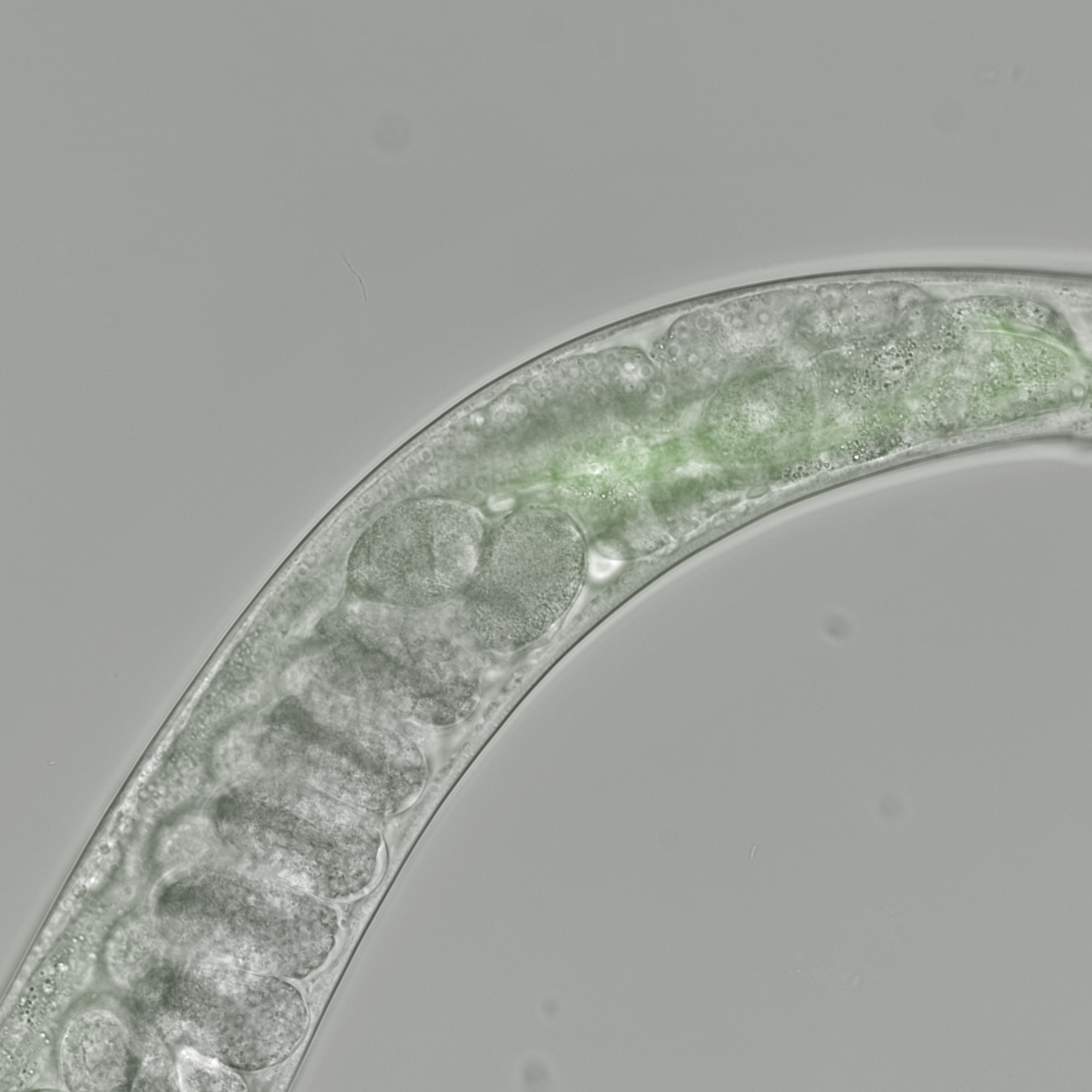

Supplement: Supplementary file 6 — Source data Fig. 3 [file 44318_2025_619_MOESM6_ESM.zip › Figure 3/3E/c.tif]

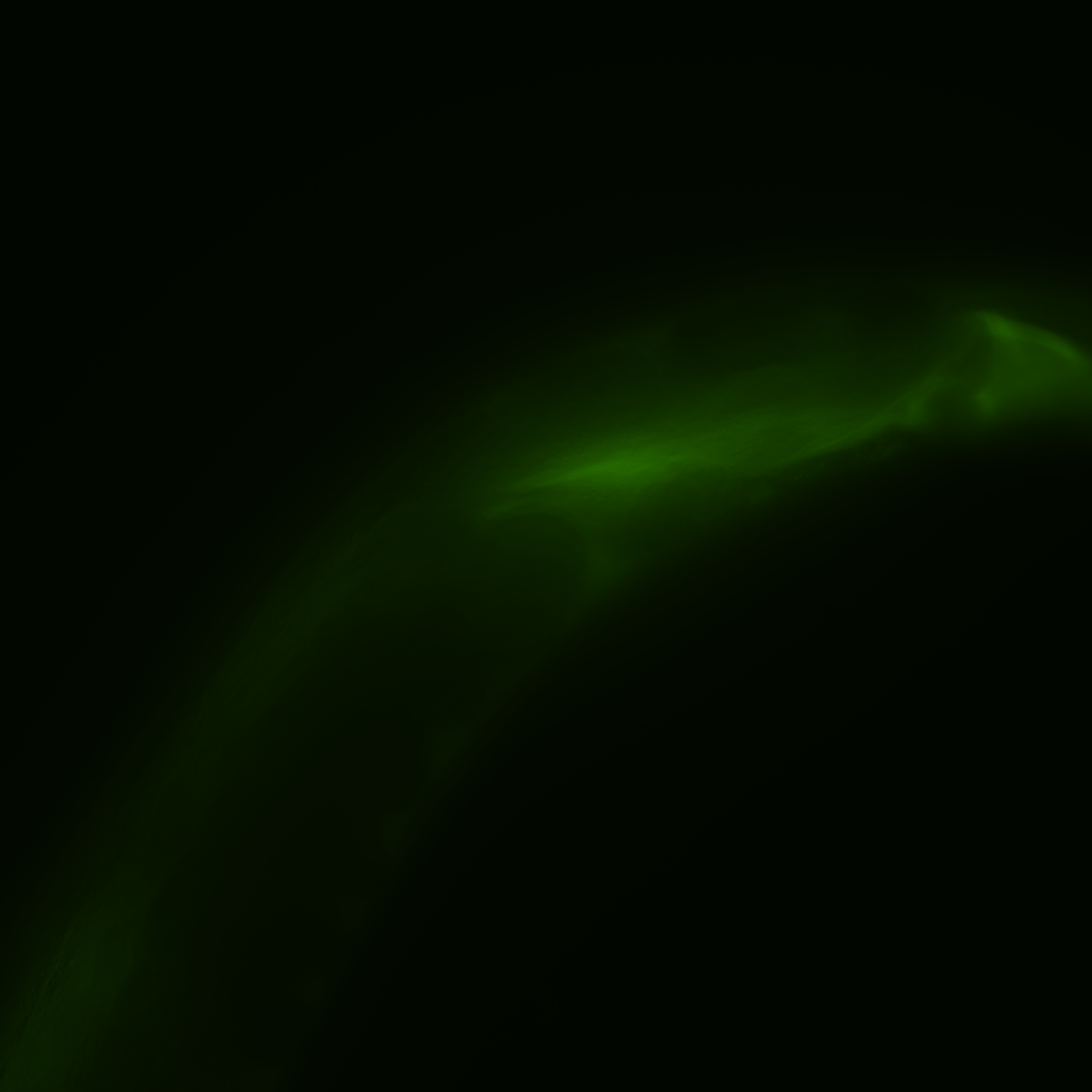

Supplement: Supplementary file 6 — Source data Fig. 3 [file 44318_2025_619_MOESM6_ESM.zip › Figure 3/3E/d.tif]

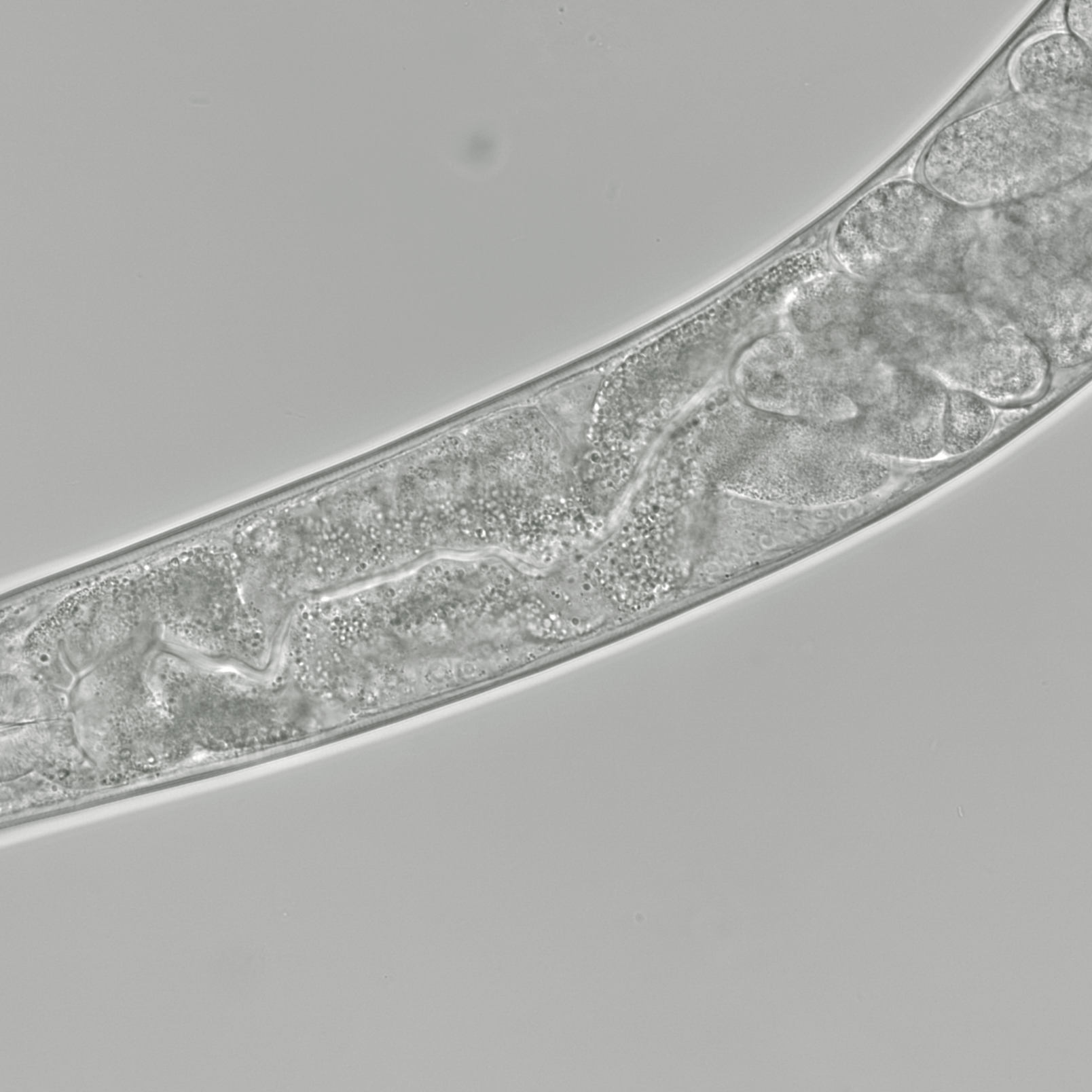

Supplement: Supplementary file 6 — Source data Fig. 3 [file 44318_2025_619_MOESM6_ESM.zip › Figure 3/3E/e.tif]

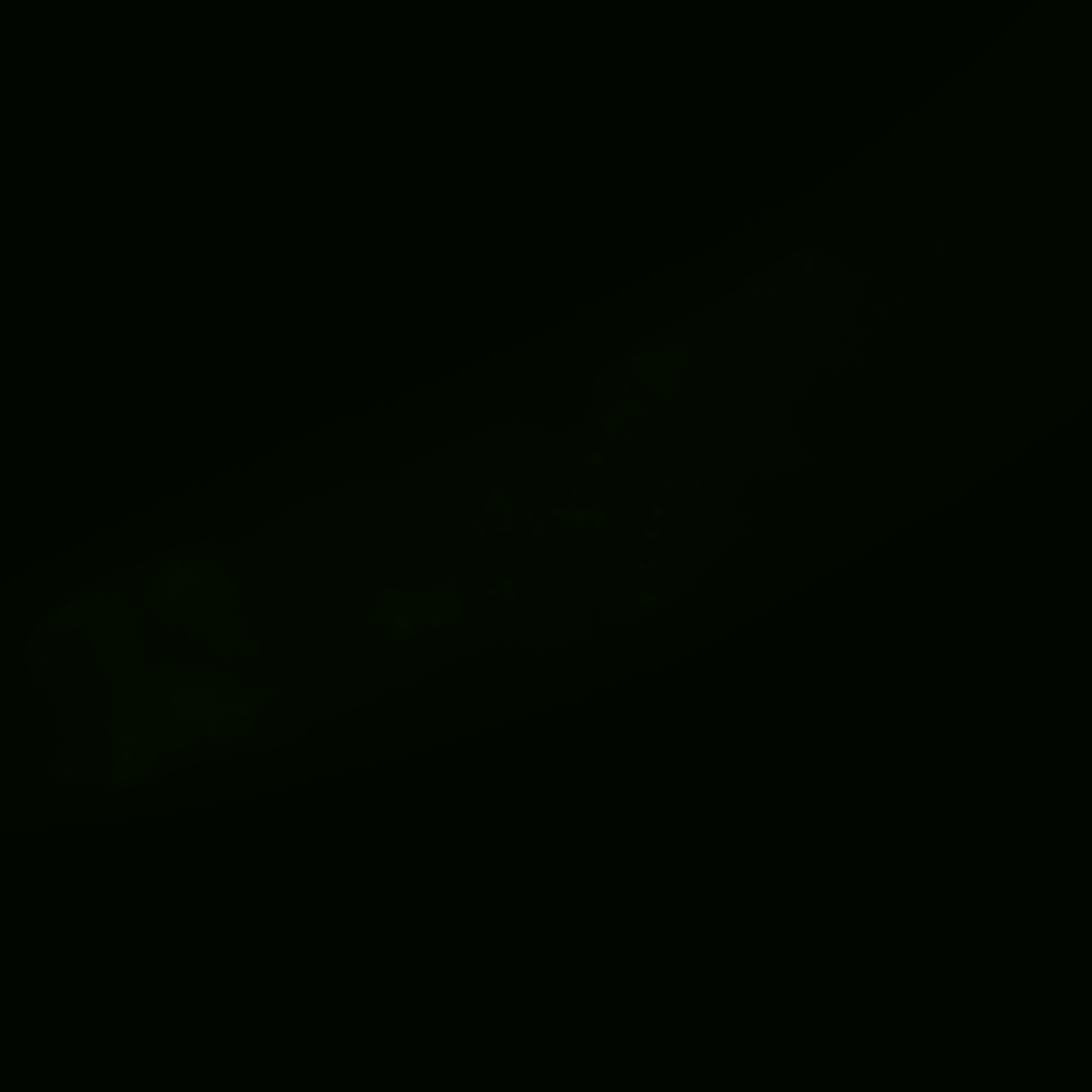

Supplement: Supplementary file 6 — Source data Fig. 3 [file 44318_2025_619_MOESM6_ESM.zip › Figure 3/3E/f.tif]

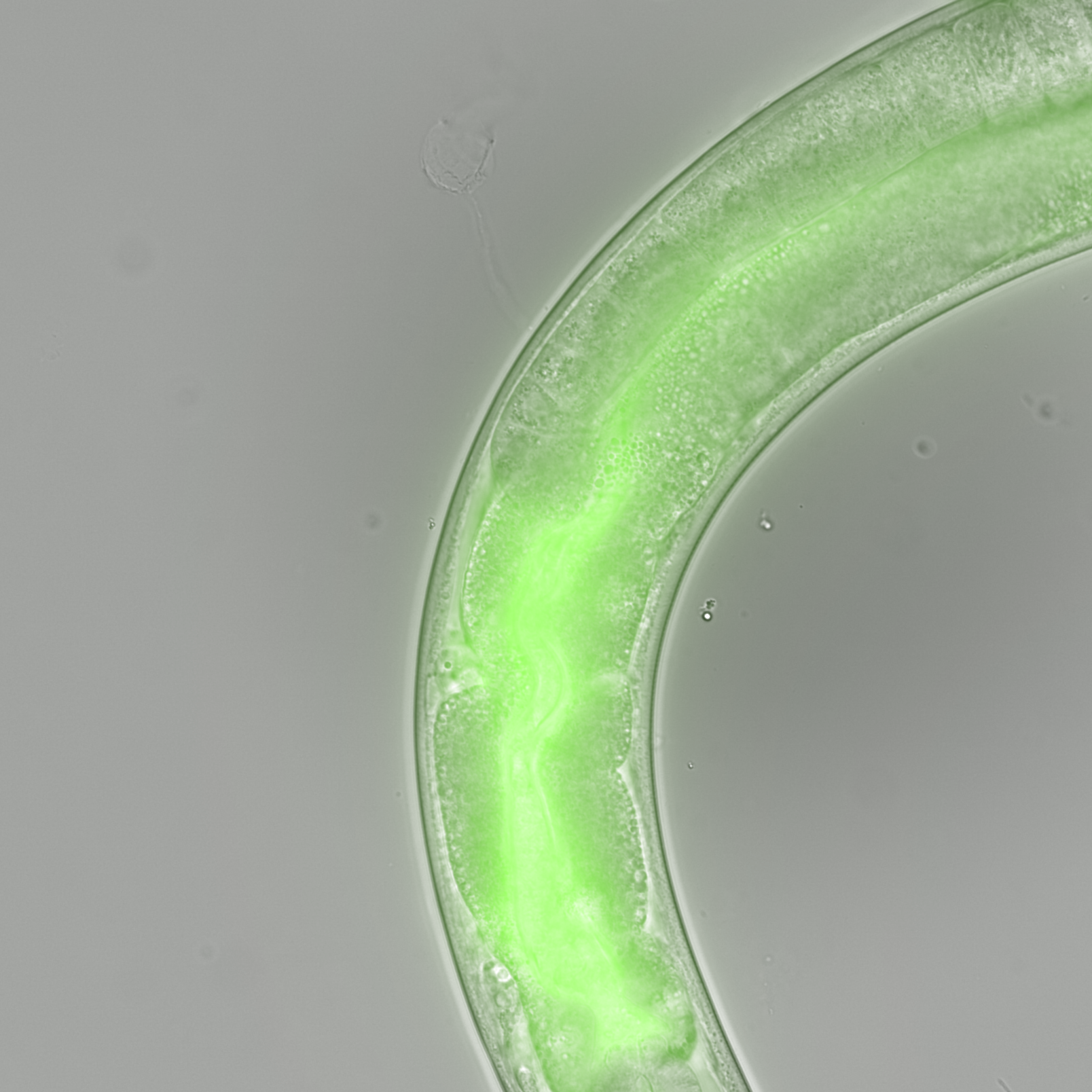

Supplement: Supplementary file 6 — Source data Fig. 3 [file 44318_2025_619_MOESM6_ESM.zip › Figure 3/3E/g.tif]

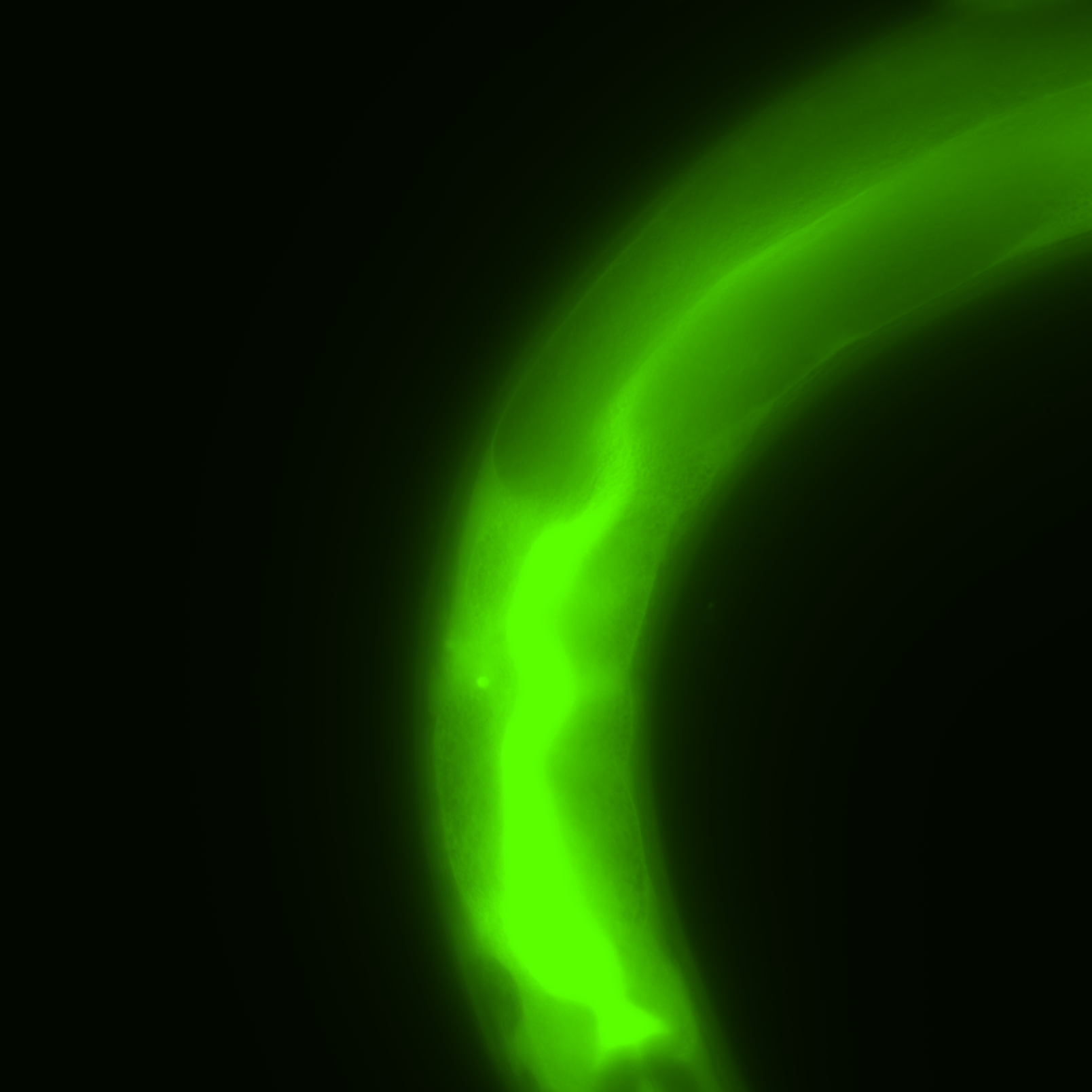

Supplement: Supplementary file 6 — Source data Fig. 3 [file 44318_2025_619_MOESM6_ESM.zip › Figure 3/3E/h.tif]

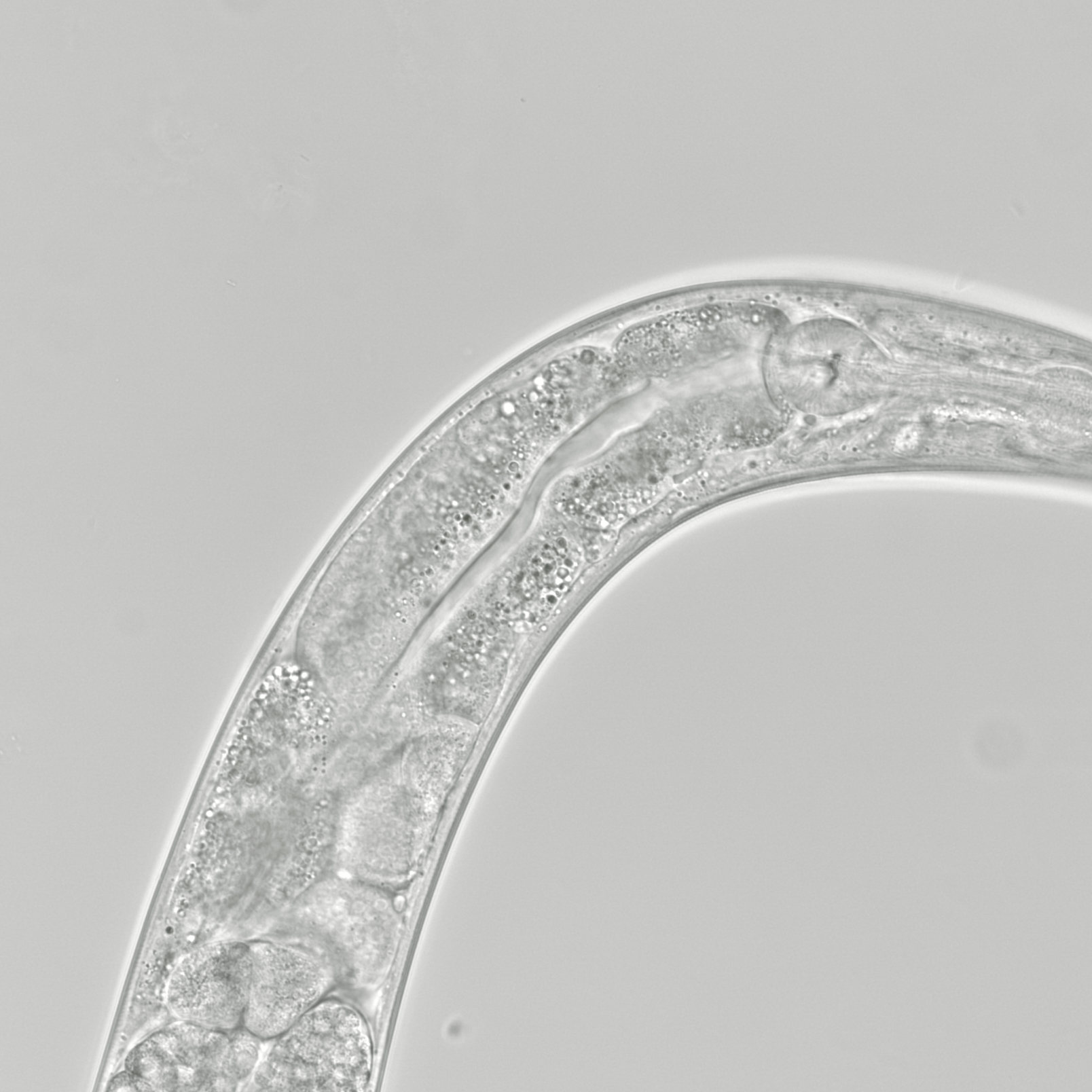

Supplement: Supplementary file 6 — Source data Fig. 3 [file 44318_2025_619_MOESM6_ESM.zip › Figure 3/3G/a.tif]

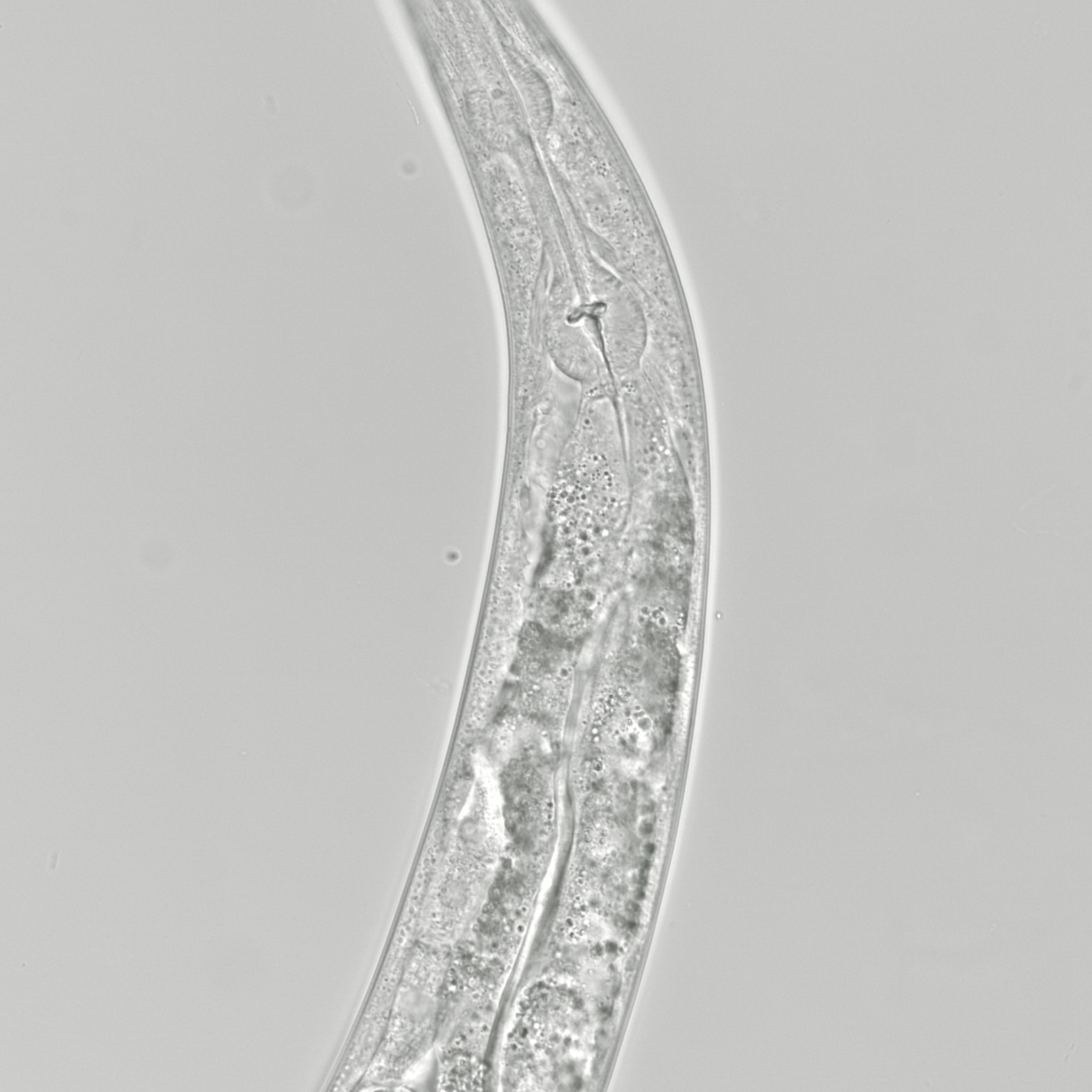

Supplement: Supplementary file 6 — Source data Fig. 3 [file 44318_2025_619_MOESM6_ESM.zip › Figure 3/3G/b.tif]

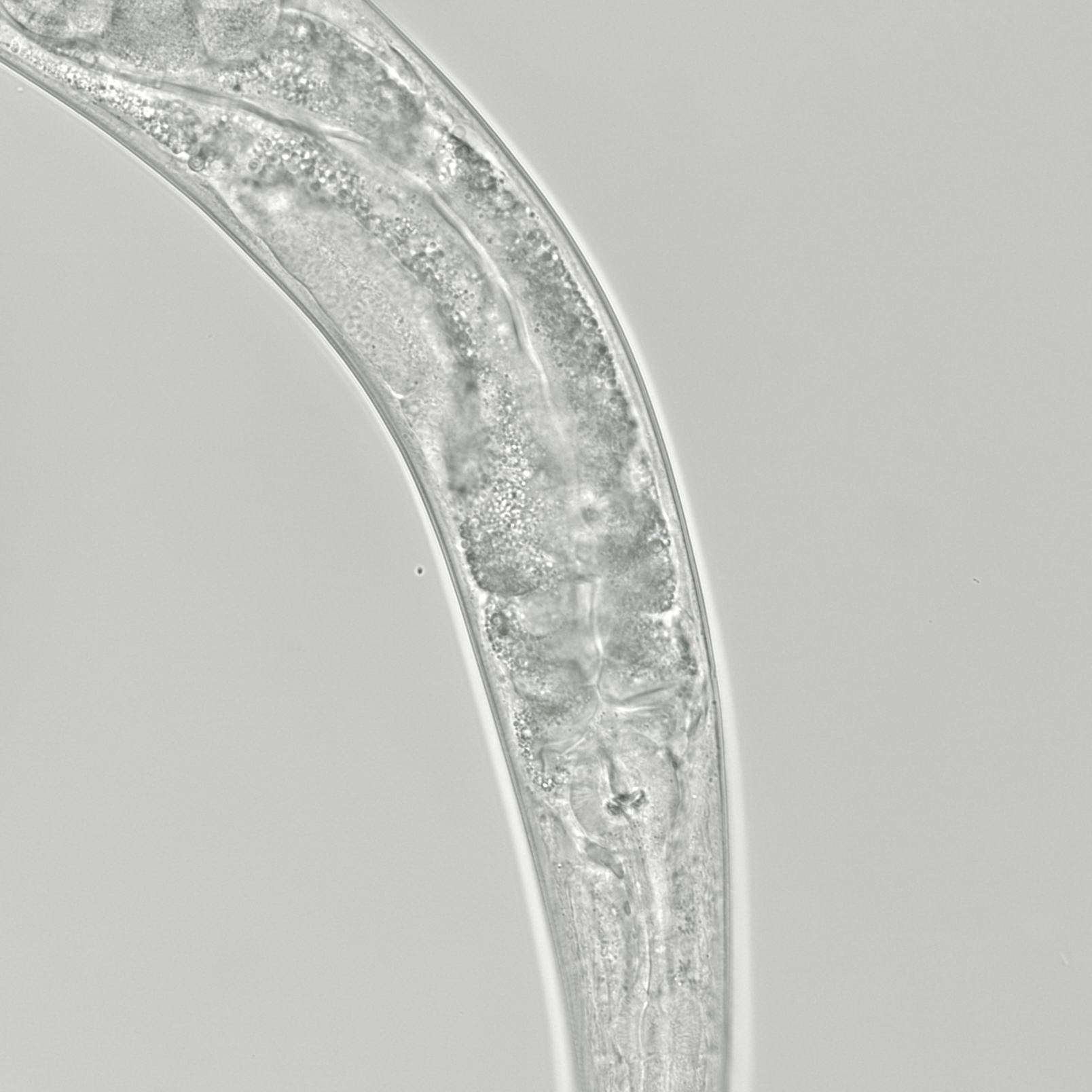

Supplement: Supplementary file 6 — Source data Fig. 3 [file 44318_2025_619_MOESM6_ESM.zip › Figure 3/3G/c.tif]

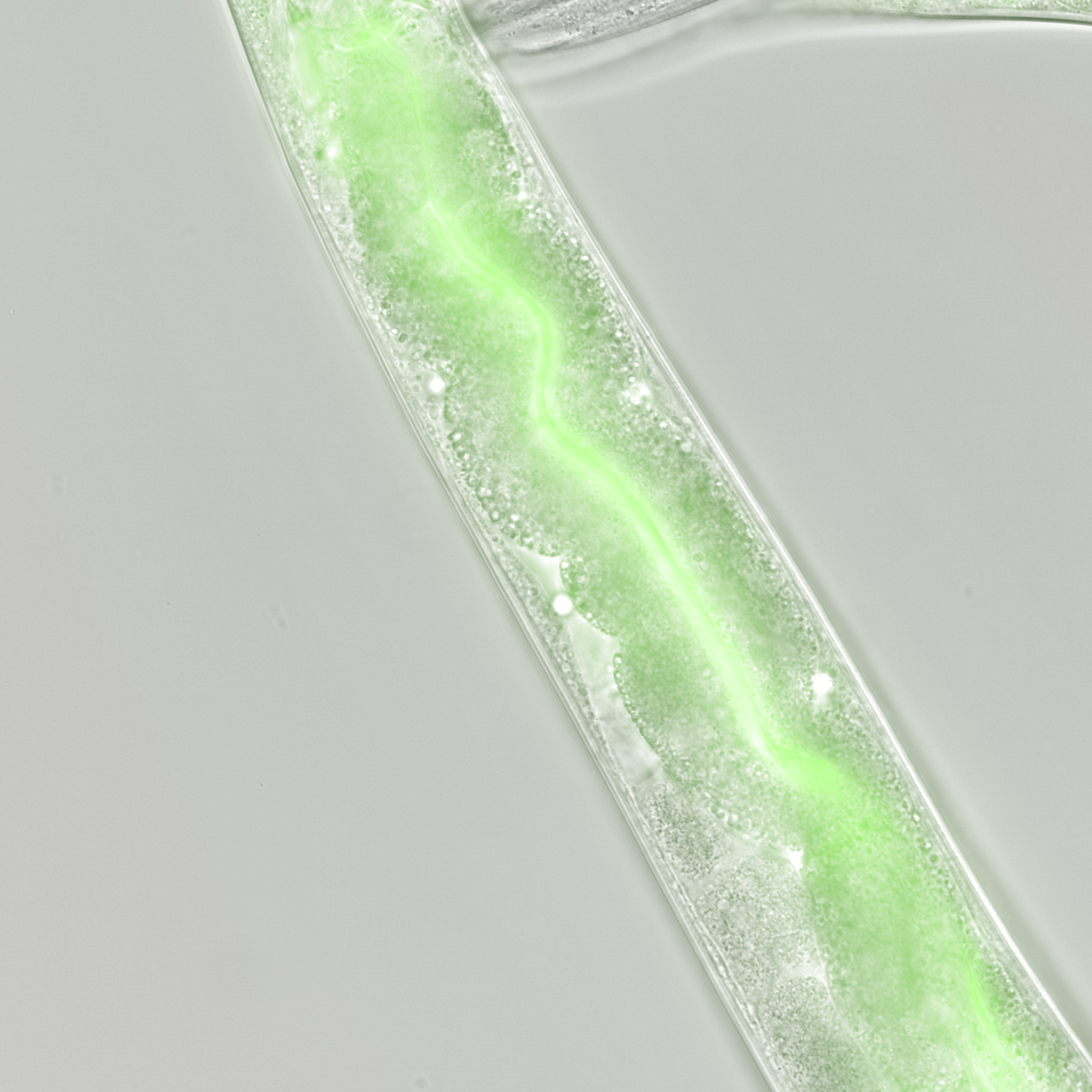

Supplement: Supplementary file 6 — Source data Fig. 3 [file 44318_2025_619_MOESM6_ESM.zip › Figure 3/3G/d.tif]

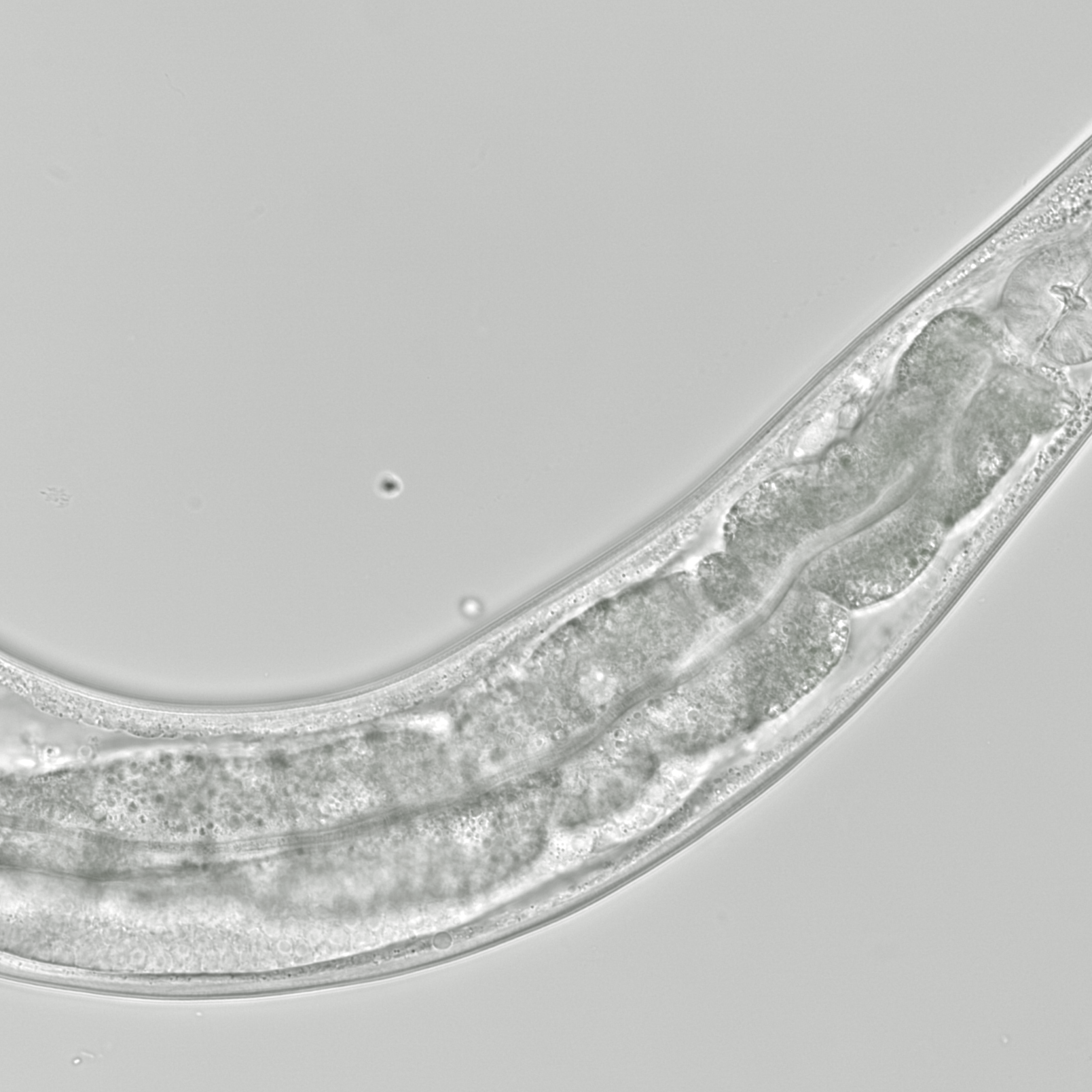

Supplement: Supplementary file 6 — Source data Fig. 3 [file 44318_2025_619_MOESM6_ESM.zip › Figure 3/3G/e.tif]

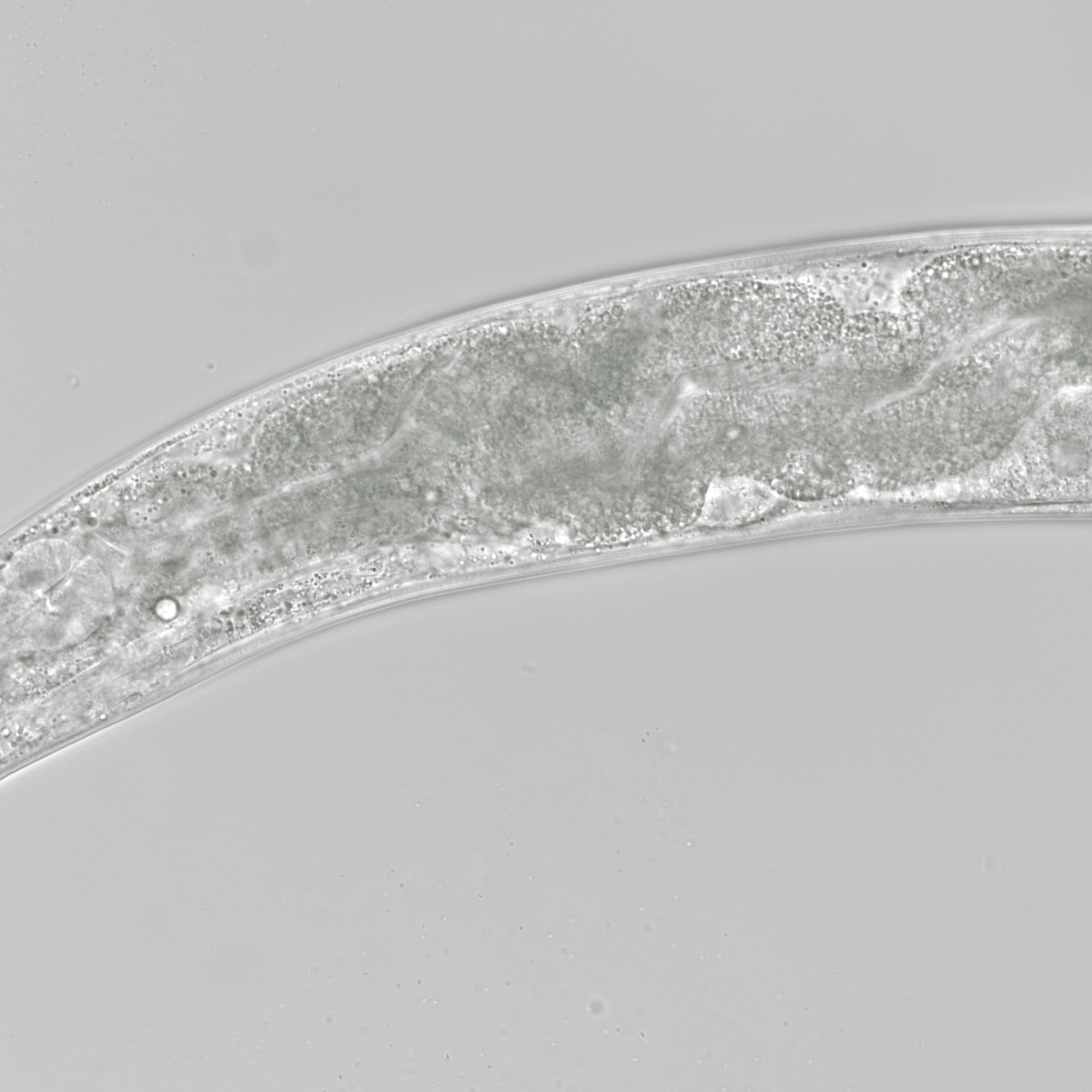

Supplement: Supplementary file 6 — Source data Fig. 3 [file 44318_2025_619_MOESM6_ESM.zip › Figure 3/3G/f.tif]

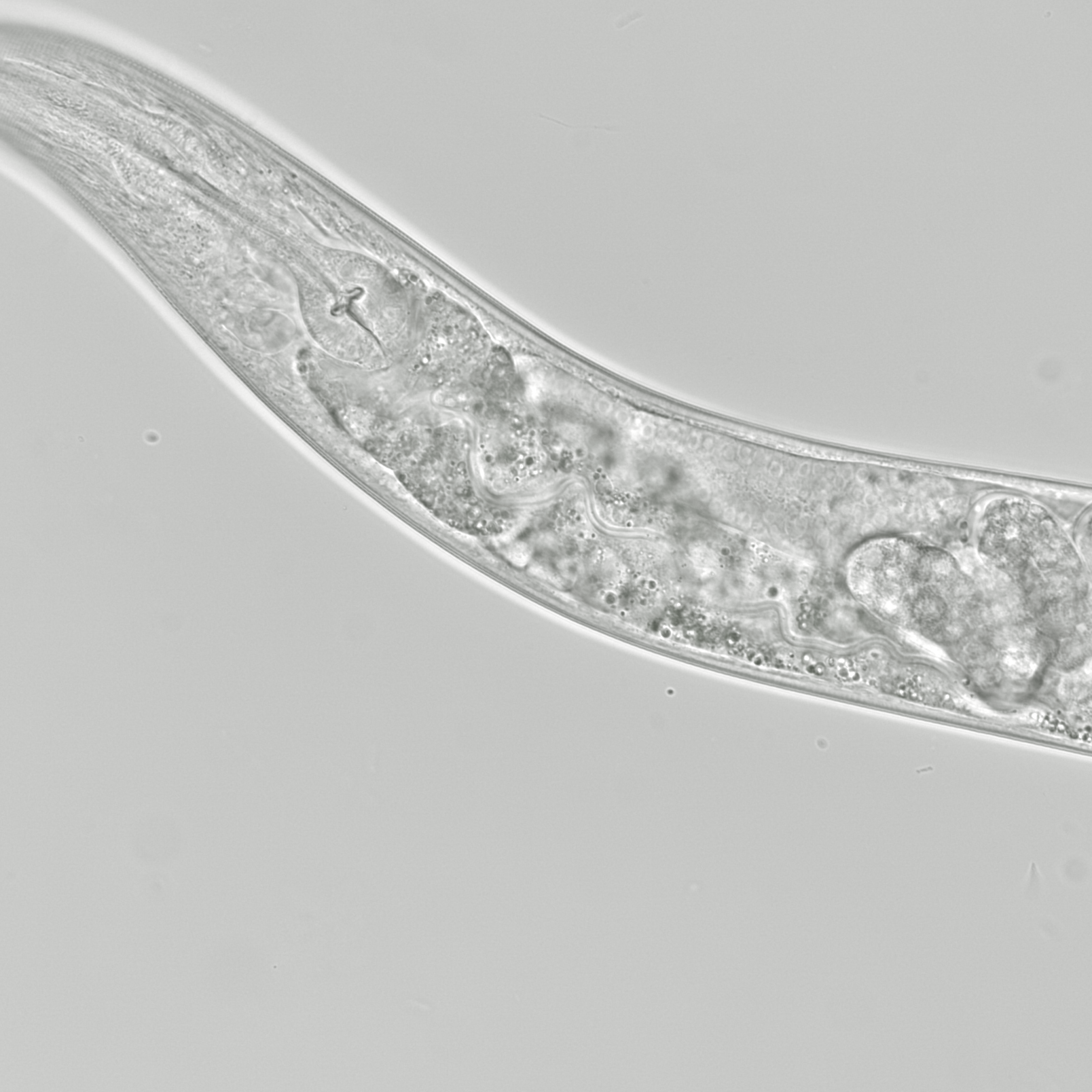

Supplement: Supplementary file 6 — Source data Fig. 3 [file 44318_2025_619_MOESM6_ESM.zip › Figure 3/3G/G.tif]

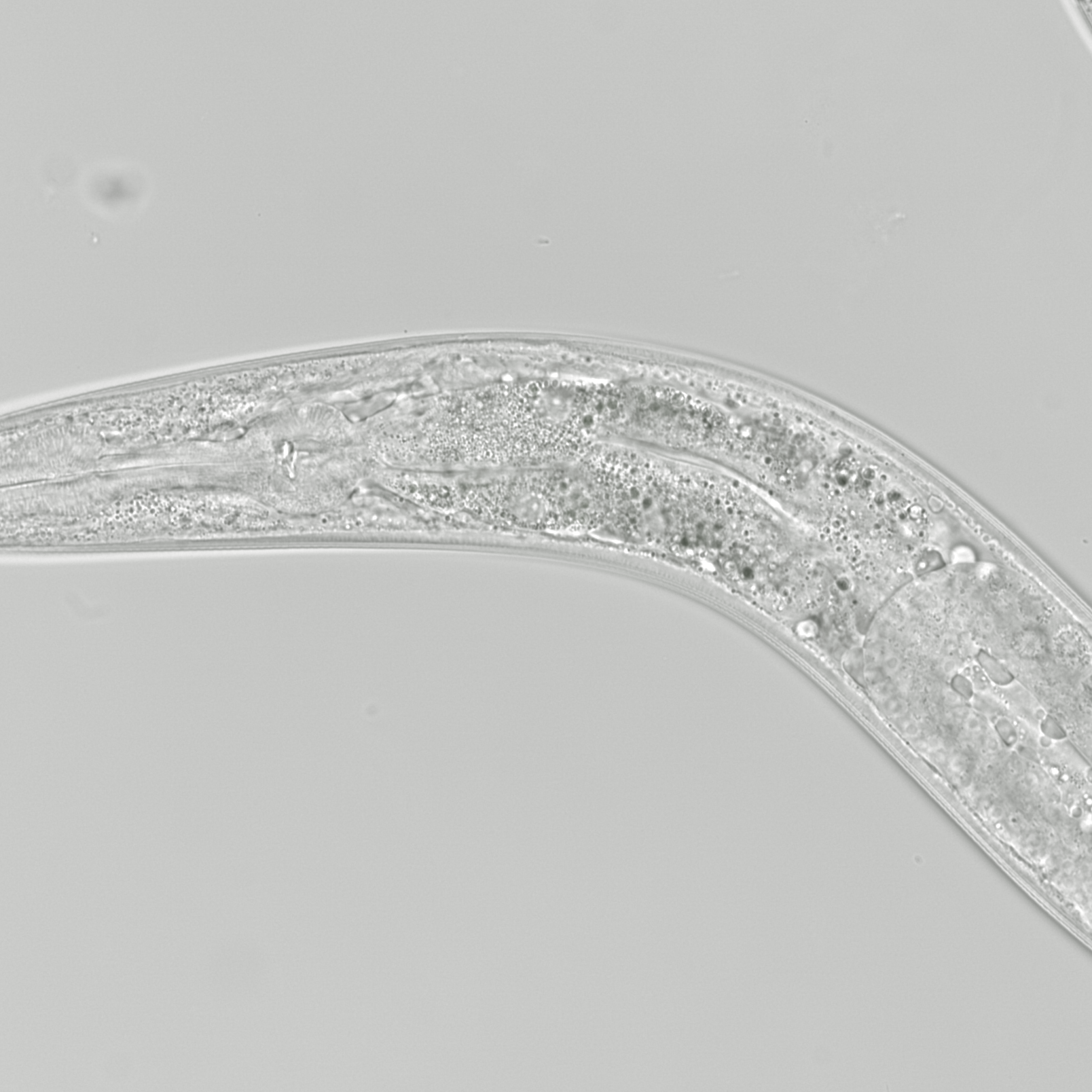

Supplement: Supplementary file 6 — Source data Fig. 3 [file 44318_2025_619_MOESM6_ESM.zip › Figure 3/3G/h.tif]

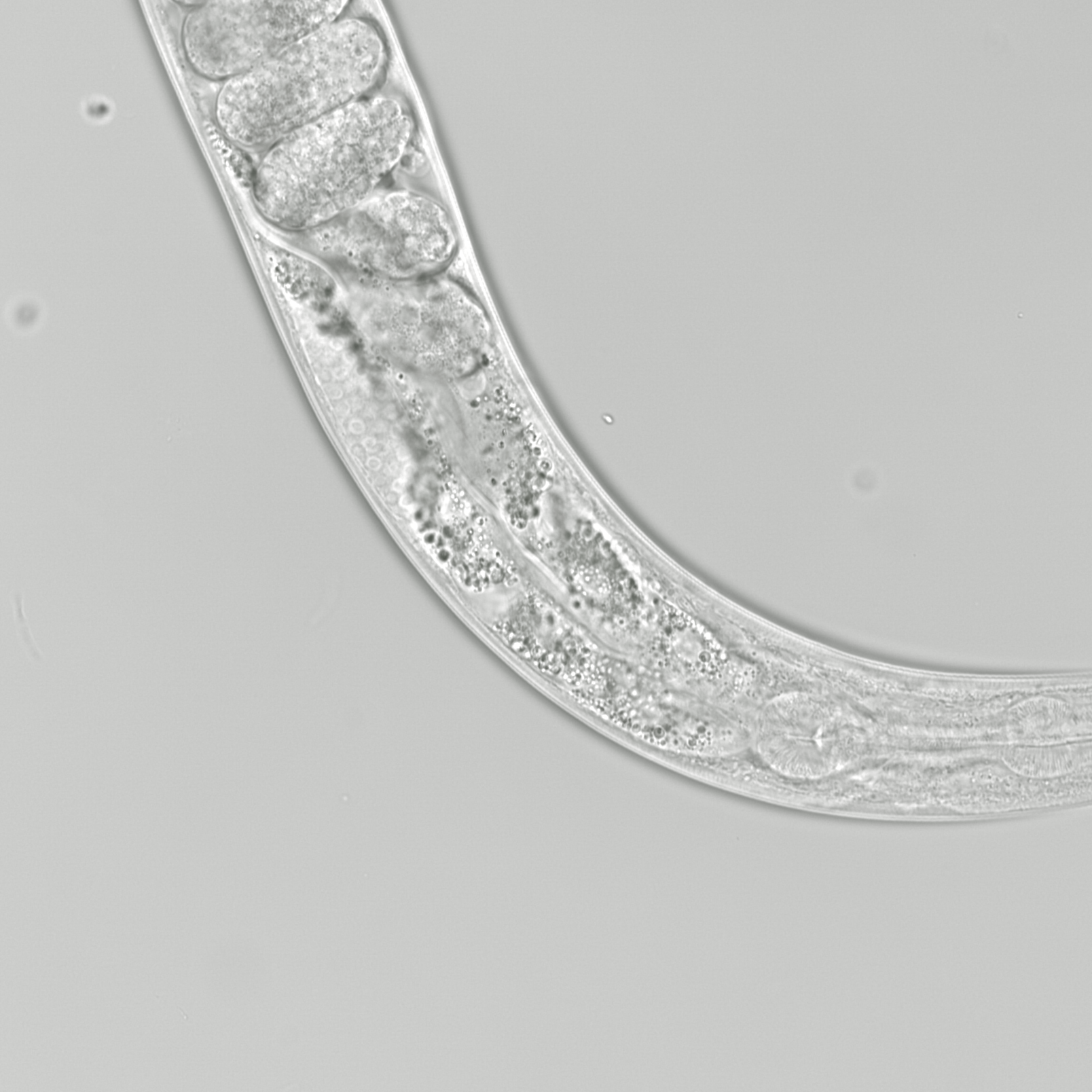

Supplement: Supplementary file 6 — Source data Fig. 3 [file 44318_2025_619_MOESM6_ESM.zip › Figure 3/3G/i.tif]

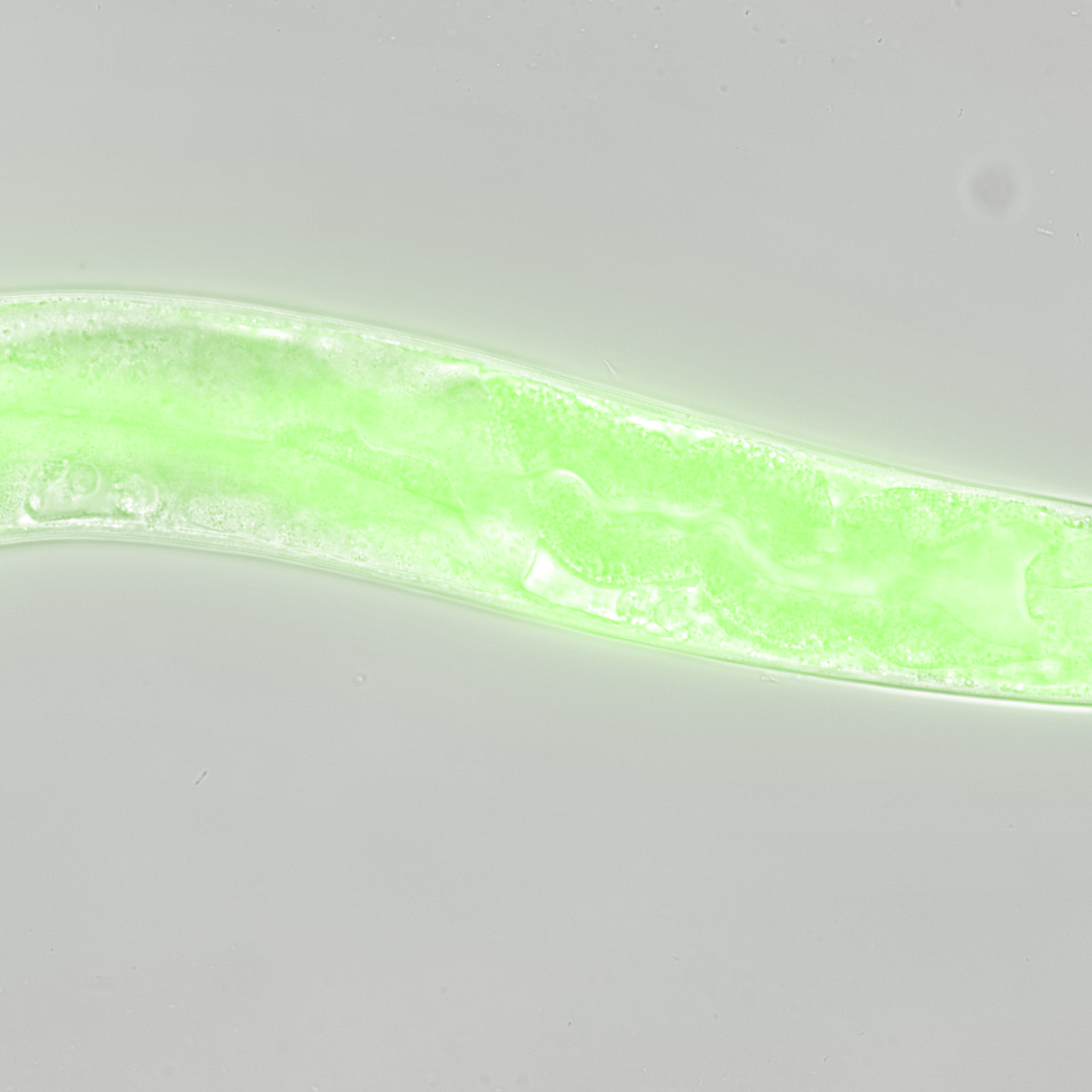

Supplement: Supplementary file 6 — Source data Fig. 3 [file 44318_2025_619_MOESM6_ESM.zip › Figure 3/3G/j.tif]

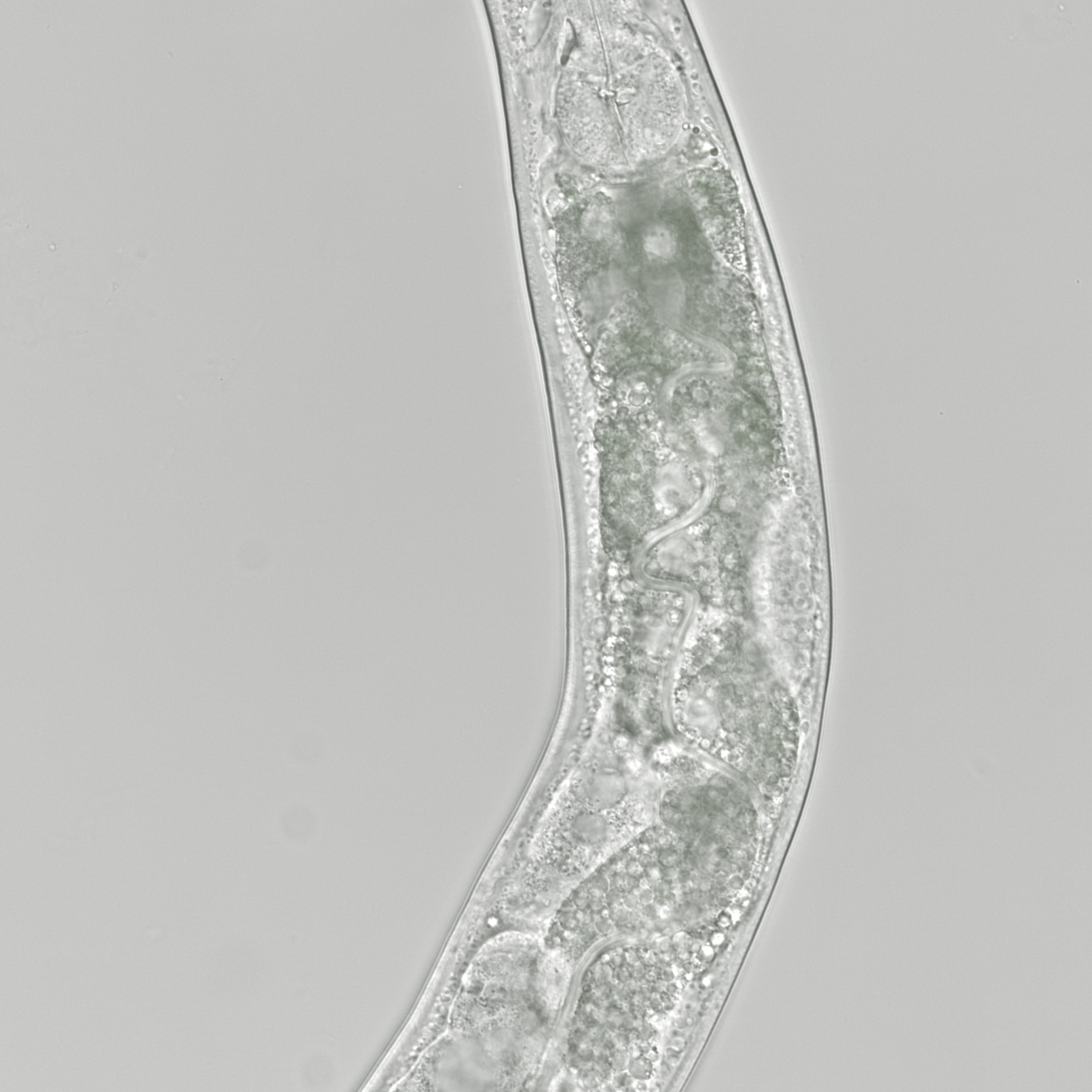

Supplement: Supplementary file 6 — Source data Fig. 3 [file 44318_2025_619_MOESM6_ESM.zip › Figure 3/3G/k.tif]

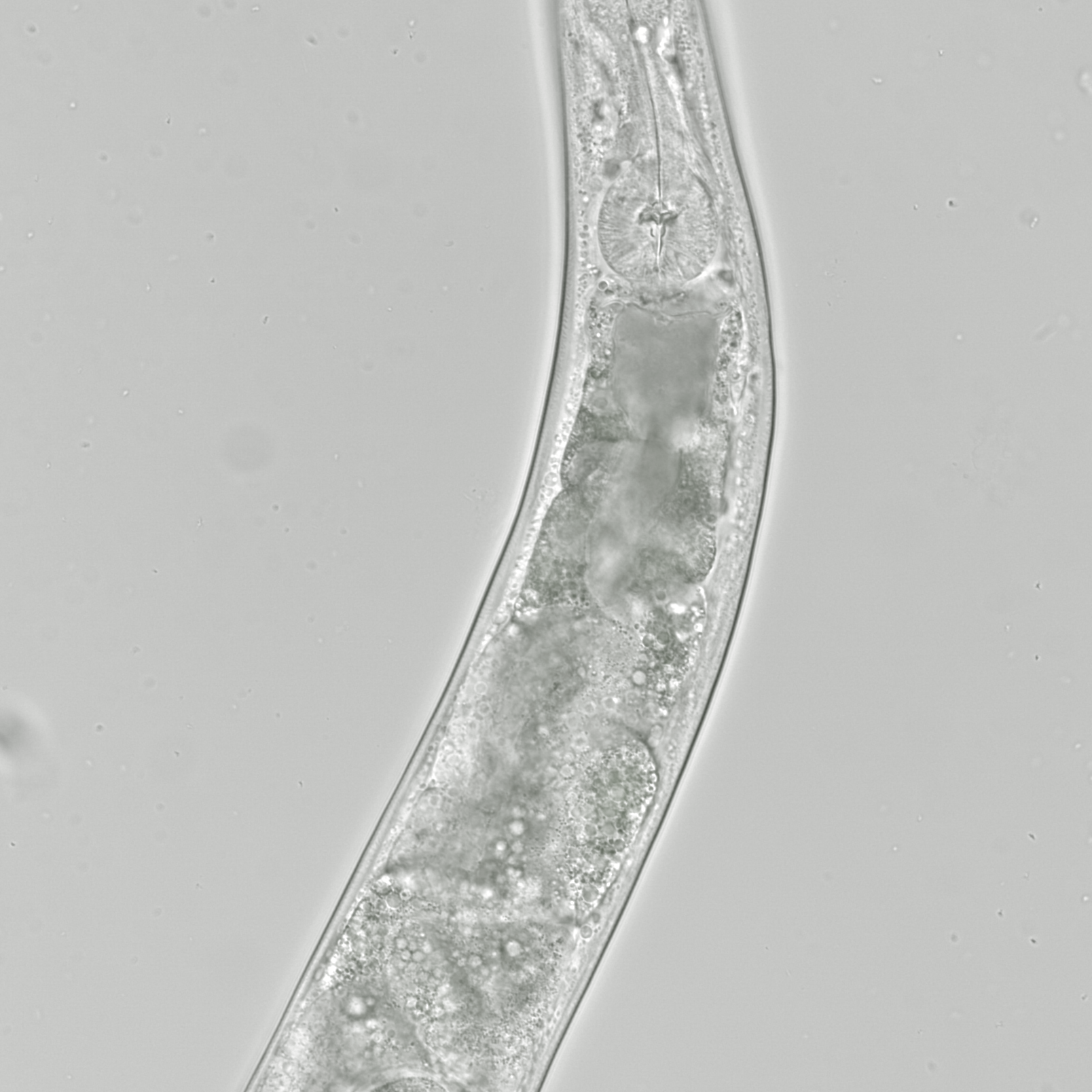

Supplement: Supplementary file 6 — Source data Fig. 3 [file 44318_2025_619_MOESM6_ESM.zip › Figure 3/3G/l.tif]

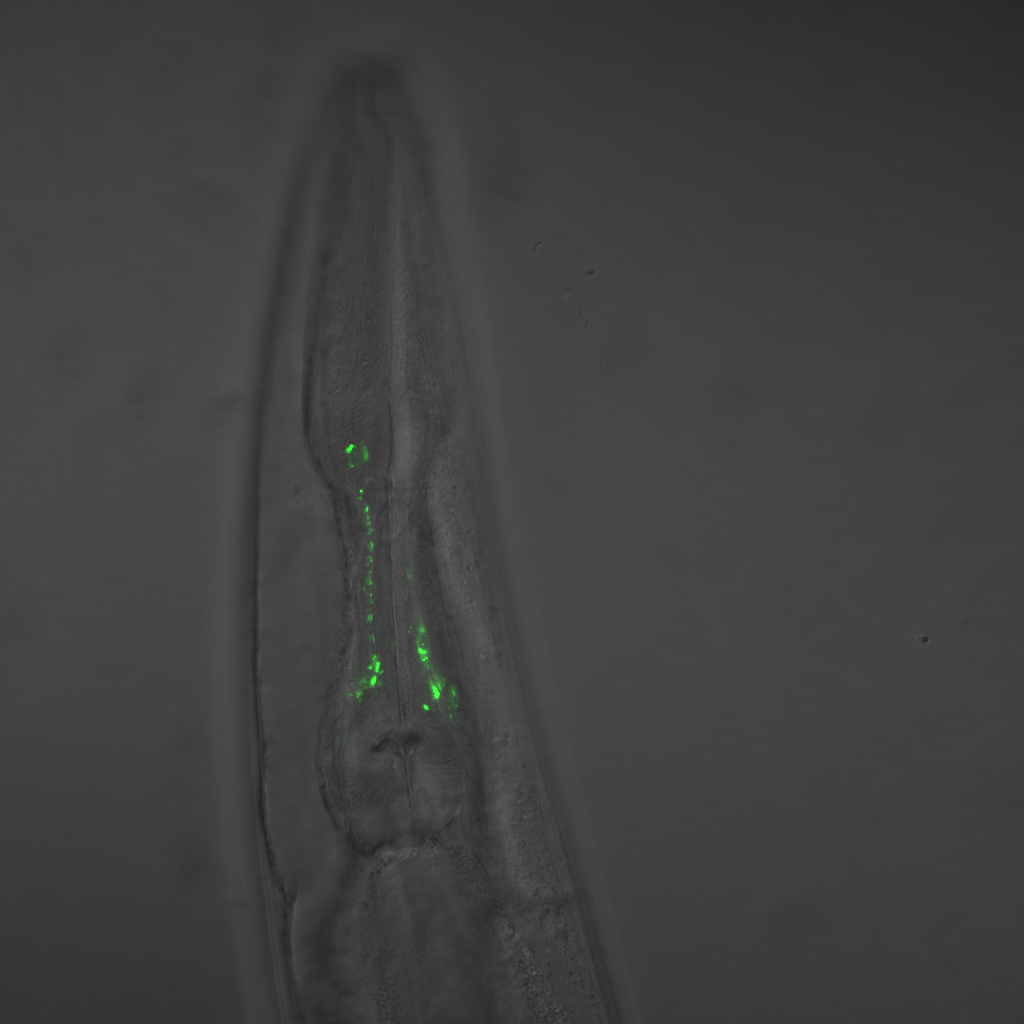

Supplement: Supplementary file 7 — Source data Fig. 4 [file 44318_2025_619_MOESM7_ESM.zip › Figure 4/4B/a.tif]

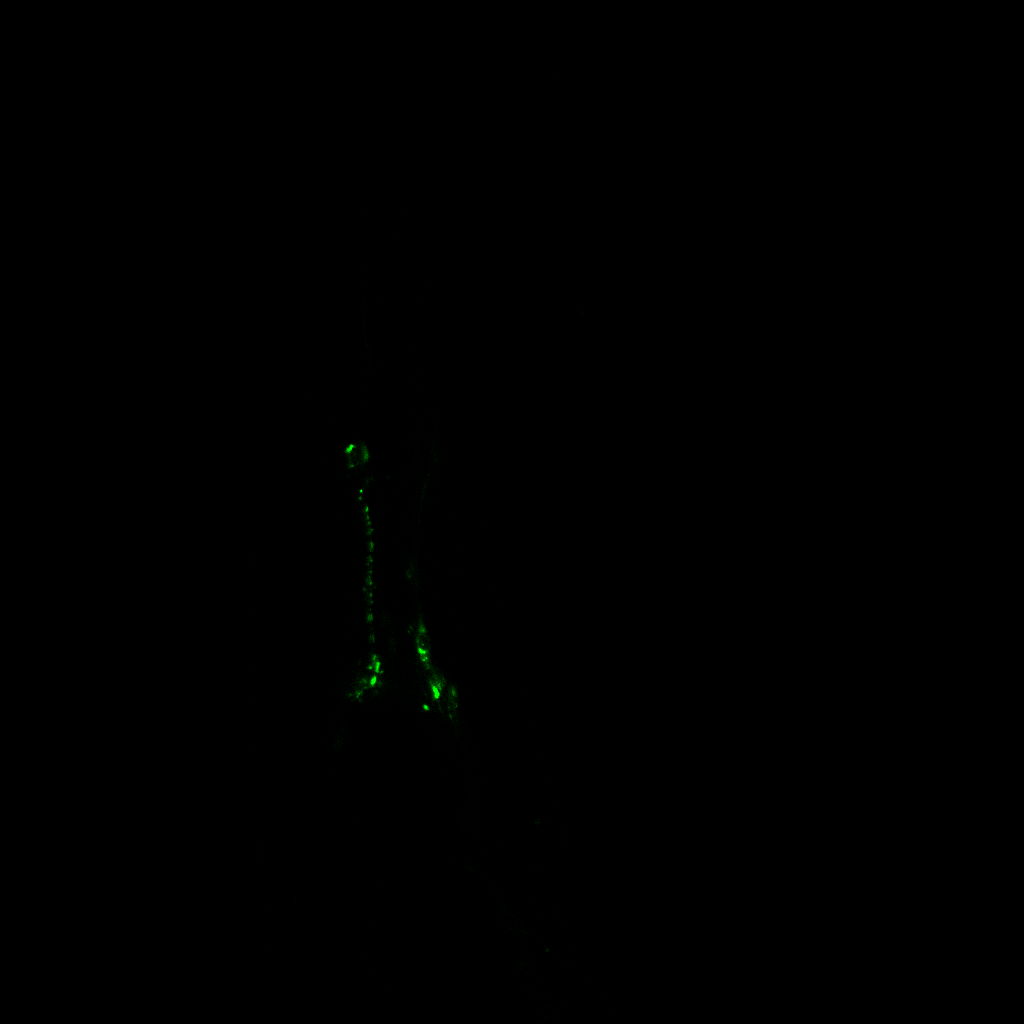

Supplement: Supplementary file 7 — Source data Fig. 4 [file 44318_2025_619_MOESM7_ESM.zip › Figure 4/4B/b.tif]

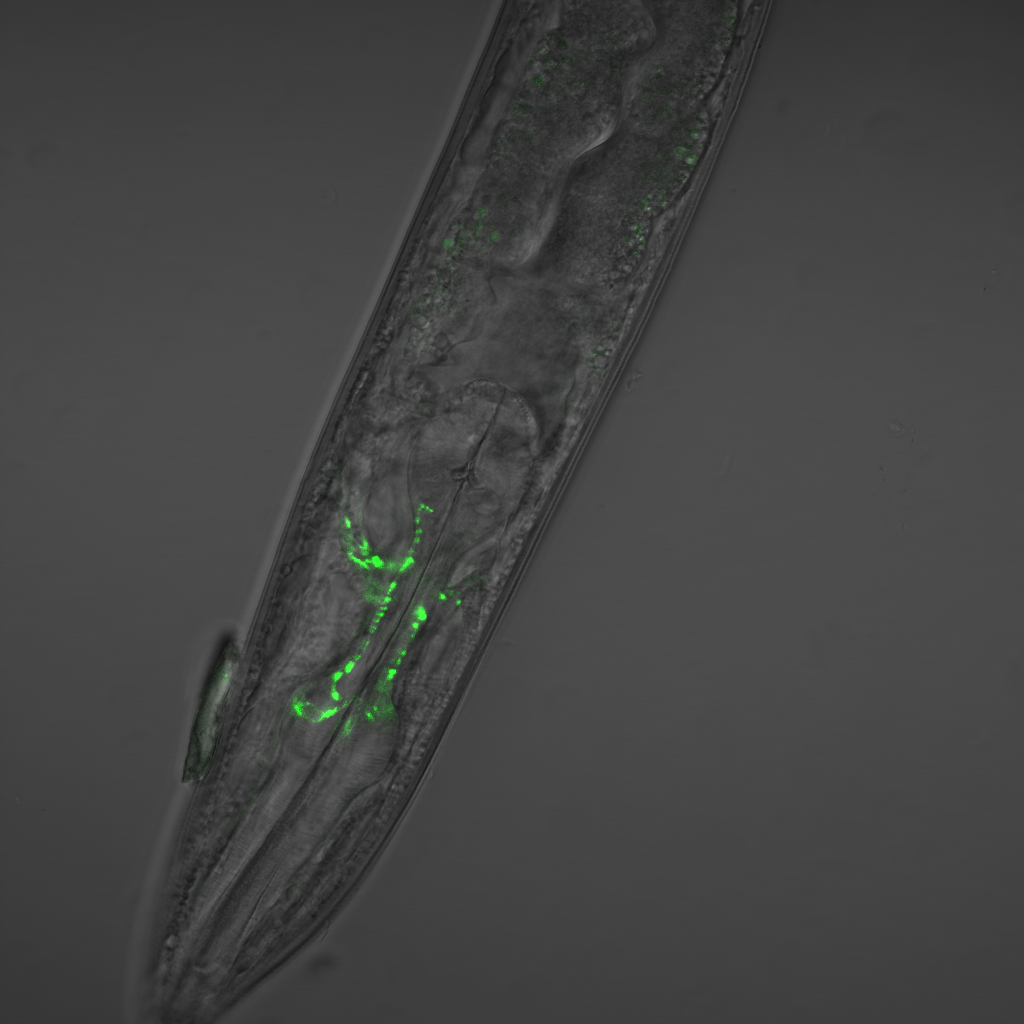

Supplement: Supplementary file 7 — Source data Fig. 4 [file 44318_2025_619_MOESM7_ESM.zip › Figure 4/4B/c.tif]

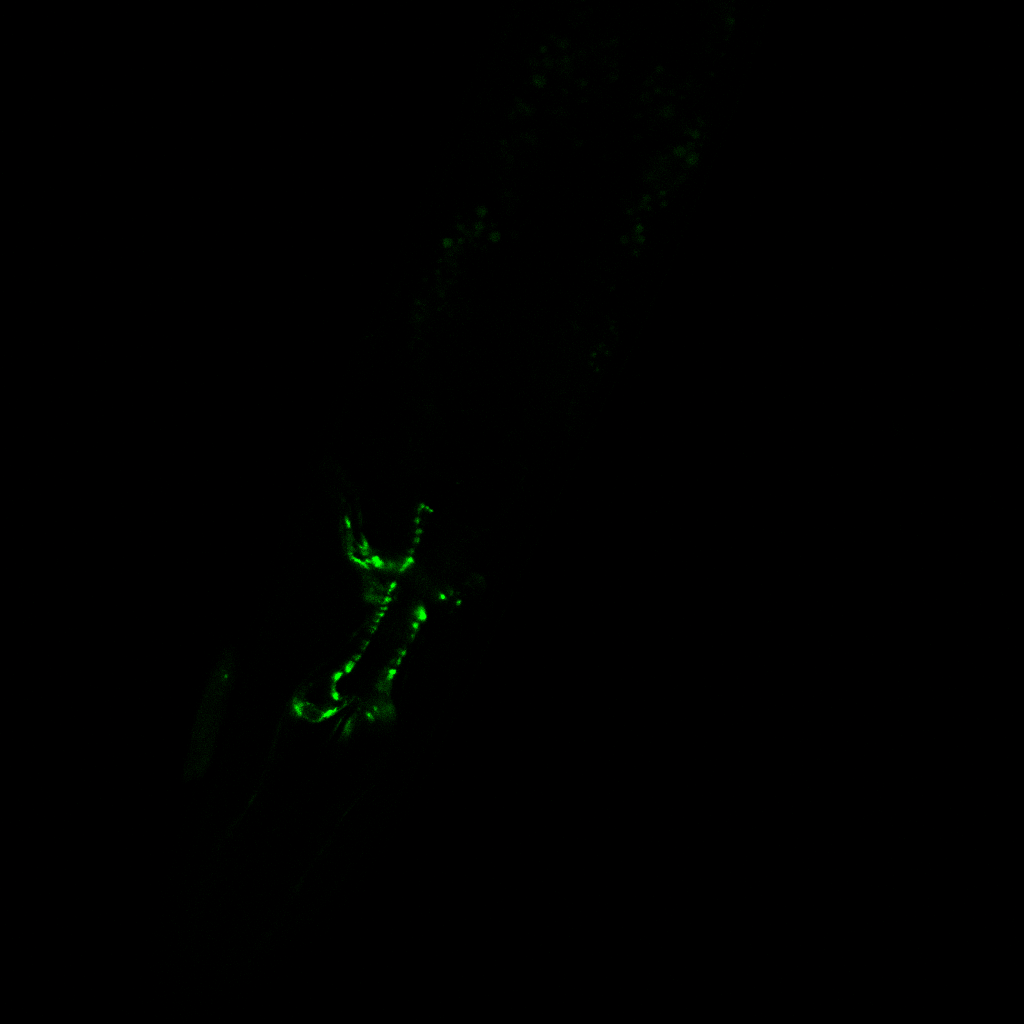

Supplement: Supplementary file 7 — Source data Fig. 4 [file 44318_2025_619_MOESM7_ESM.zip › Figure 4/4B/d.tif]

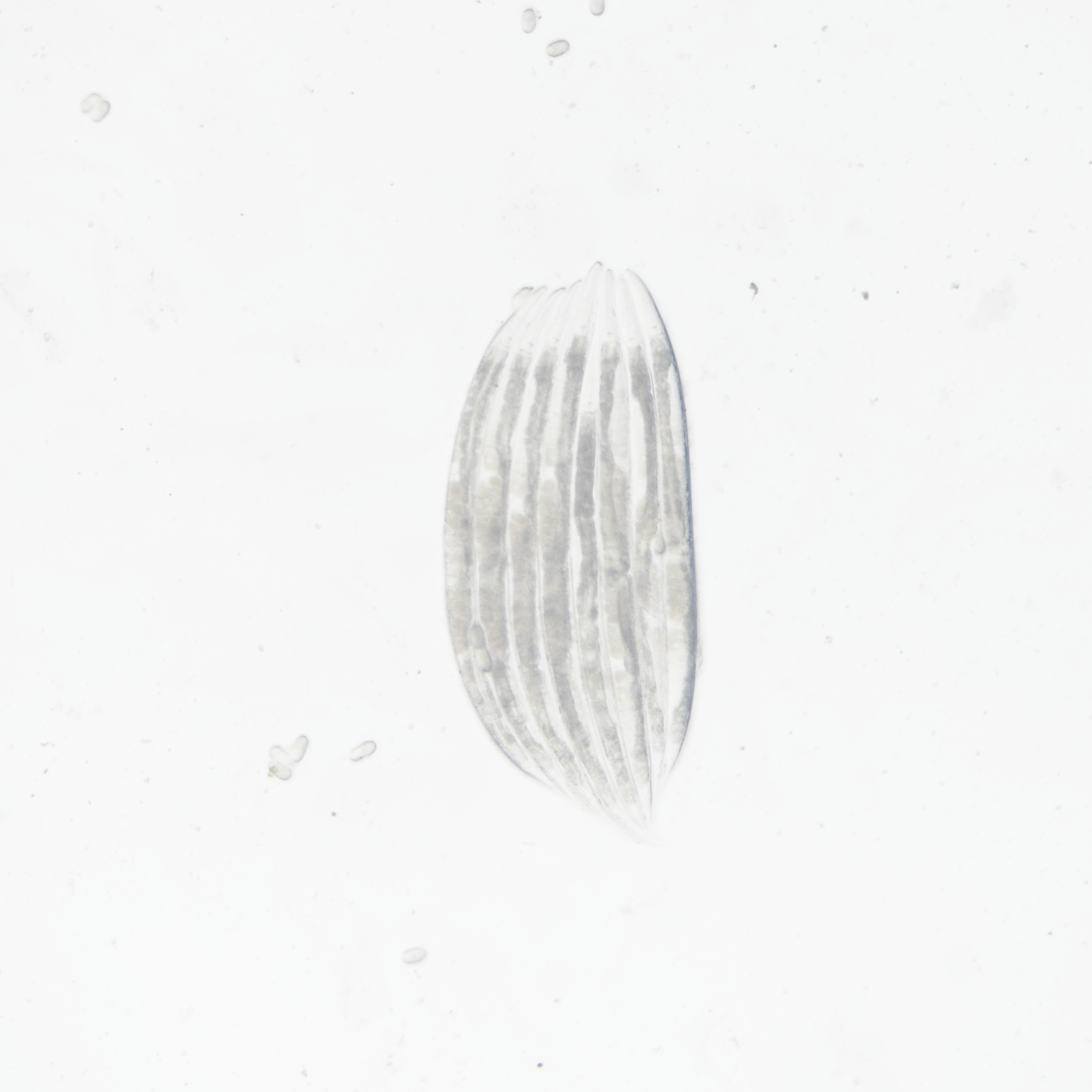

Supplement: Supplementary file 7 — Source data Fig. 4 [file 44318_2025_619_MOESM7_ESM.zip › Figure 4/4D/a.tif]

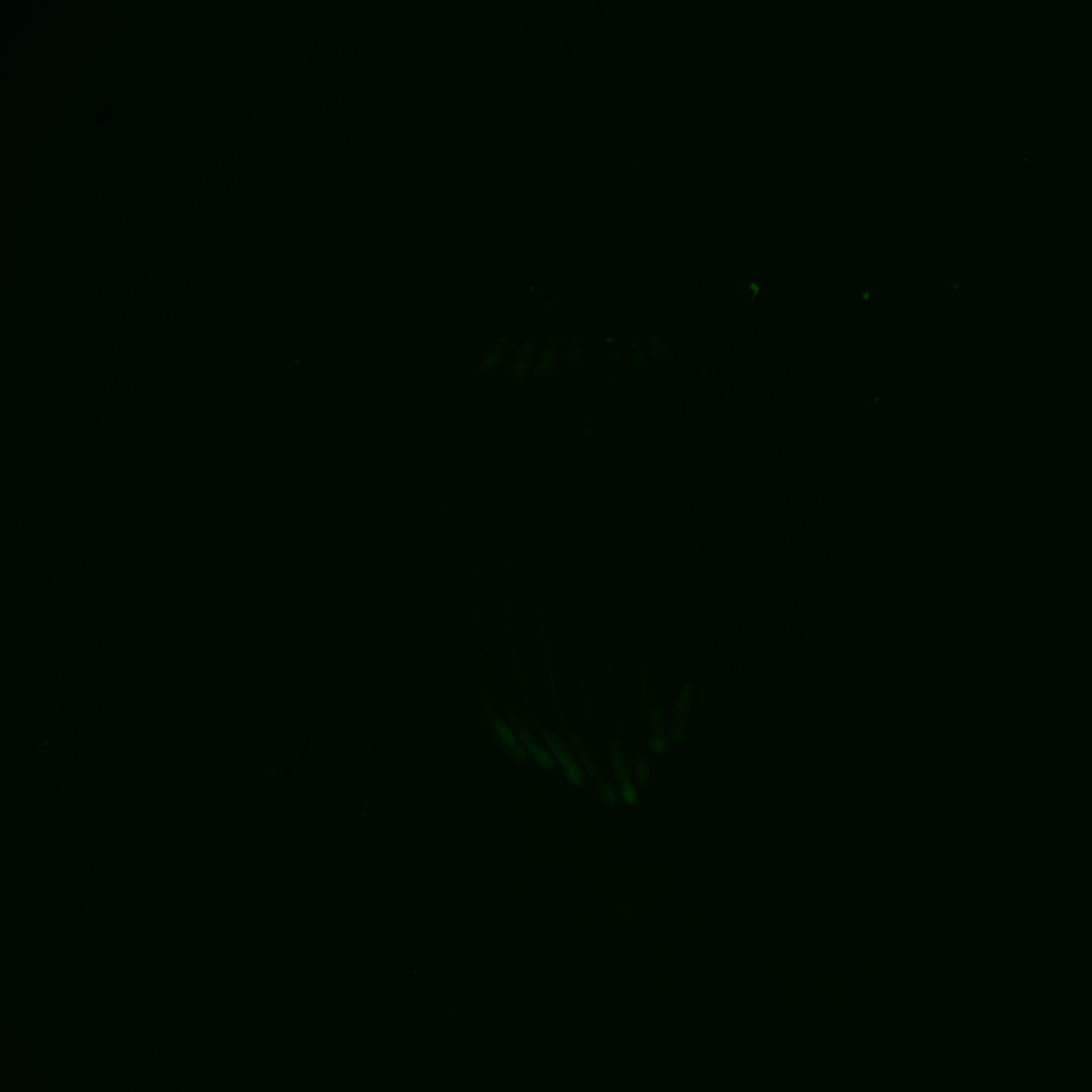

Supplement: Supplementary file 7 — Source data Fig. 4 [file 44318_2025_619_MOESM7_ESM.zip › Figure 4/4D/b.tif]

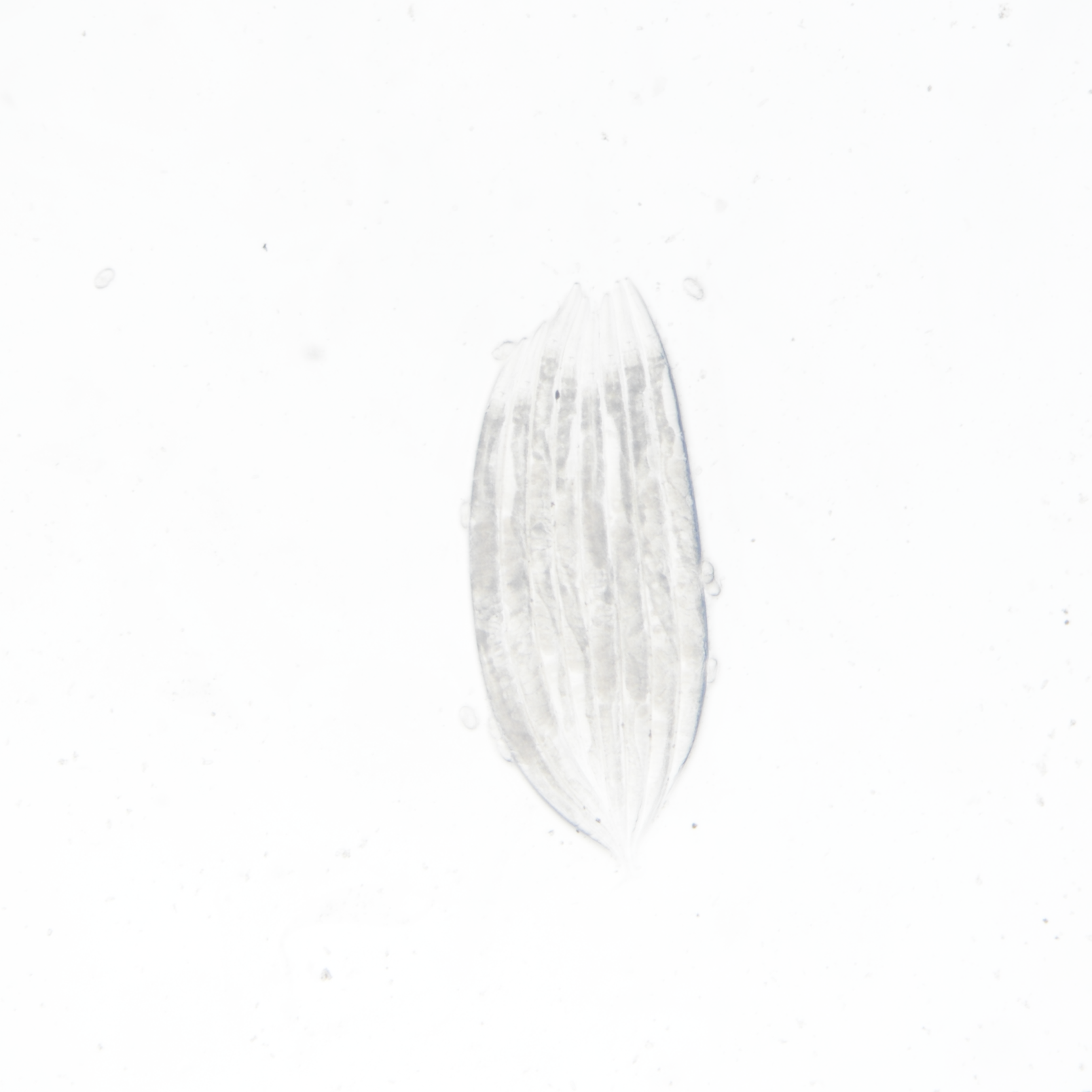

Supplement: Supplementary file 7 — Source data Fig. 4 [file 44318_2025_619_MOESM7_ESM.zip › Figure 4/4D/c.tif]

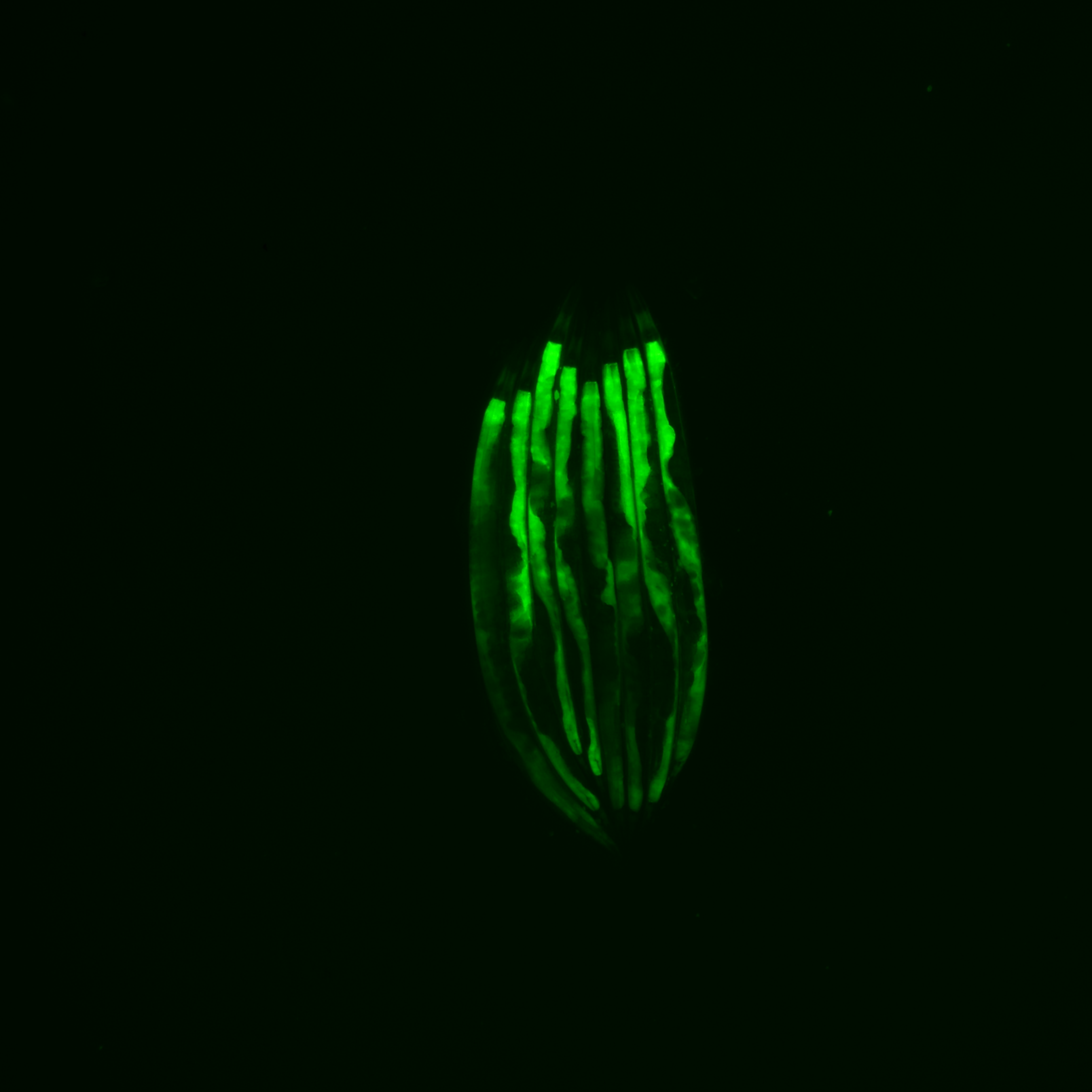

Supplement: Supplementary file 7 — Source data Fig. 4 [file 44318_2025_619_MOESM7_ESM.zip › Figure 4/4D/d.tif]

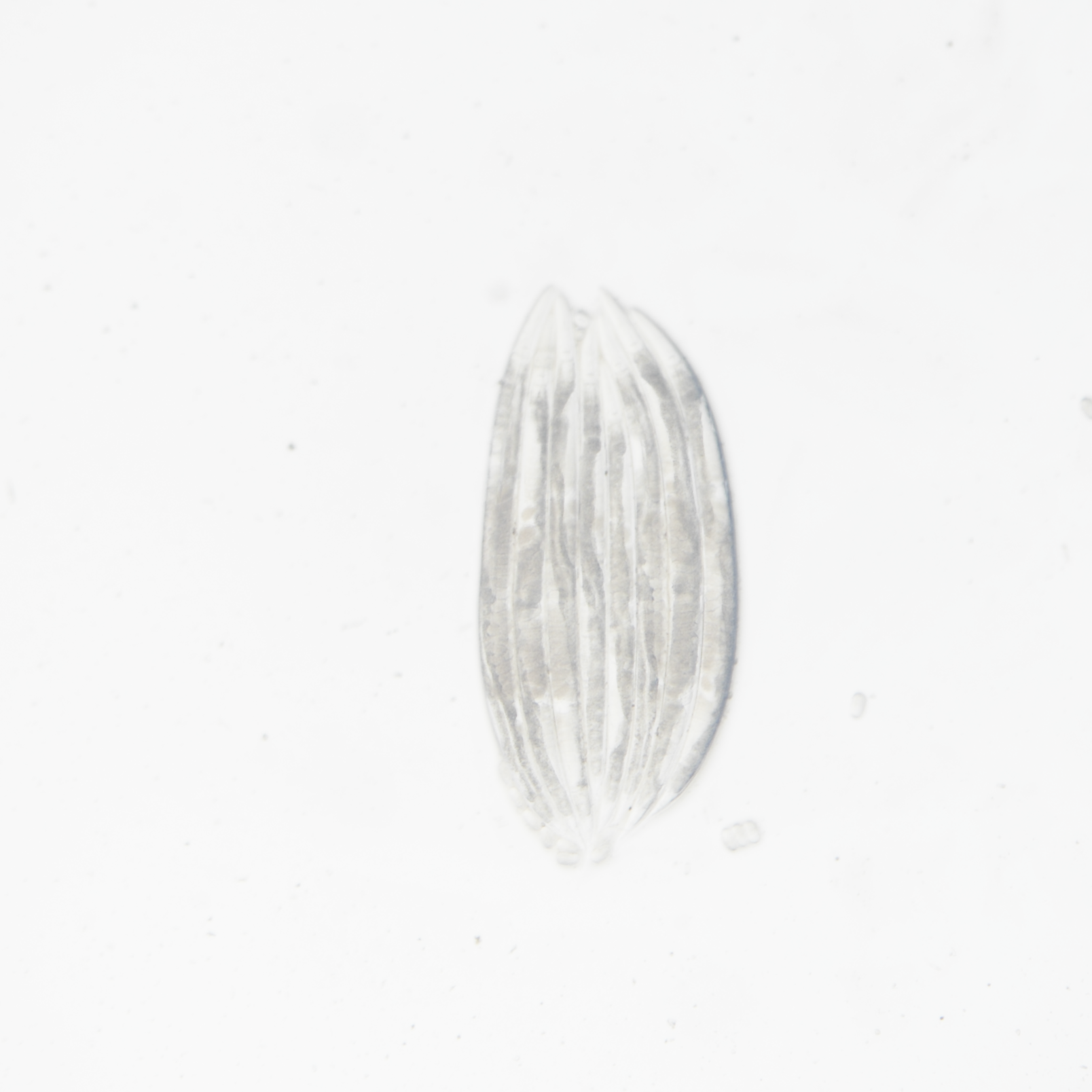

Supplement: Supplementary file 7 — Source data Fig. 4 [file 44318_2025_619_MOESM7_ESM.zip › Figure 4/4D/e.tif]

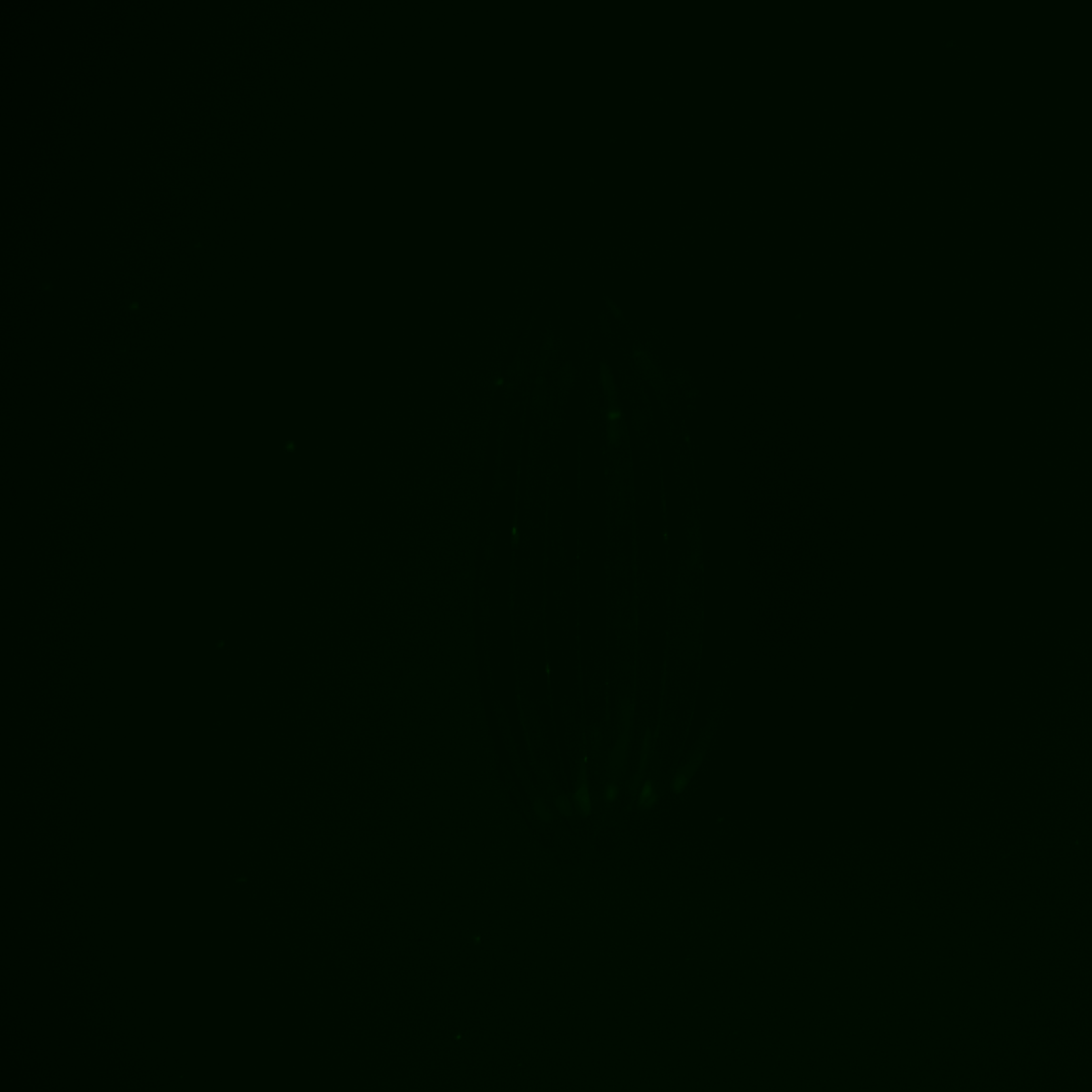

Supplement: Supplementary file 7 — Source data Fig. 4 [file 44318_2025_619_MOESM7_ESM.zip › Figure 4/4D/f.tif]

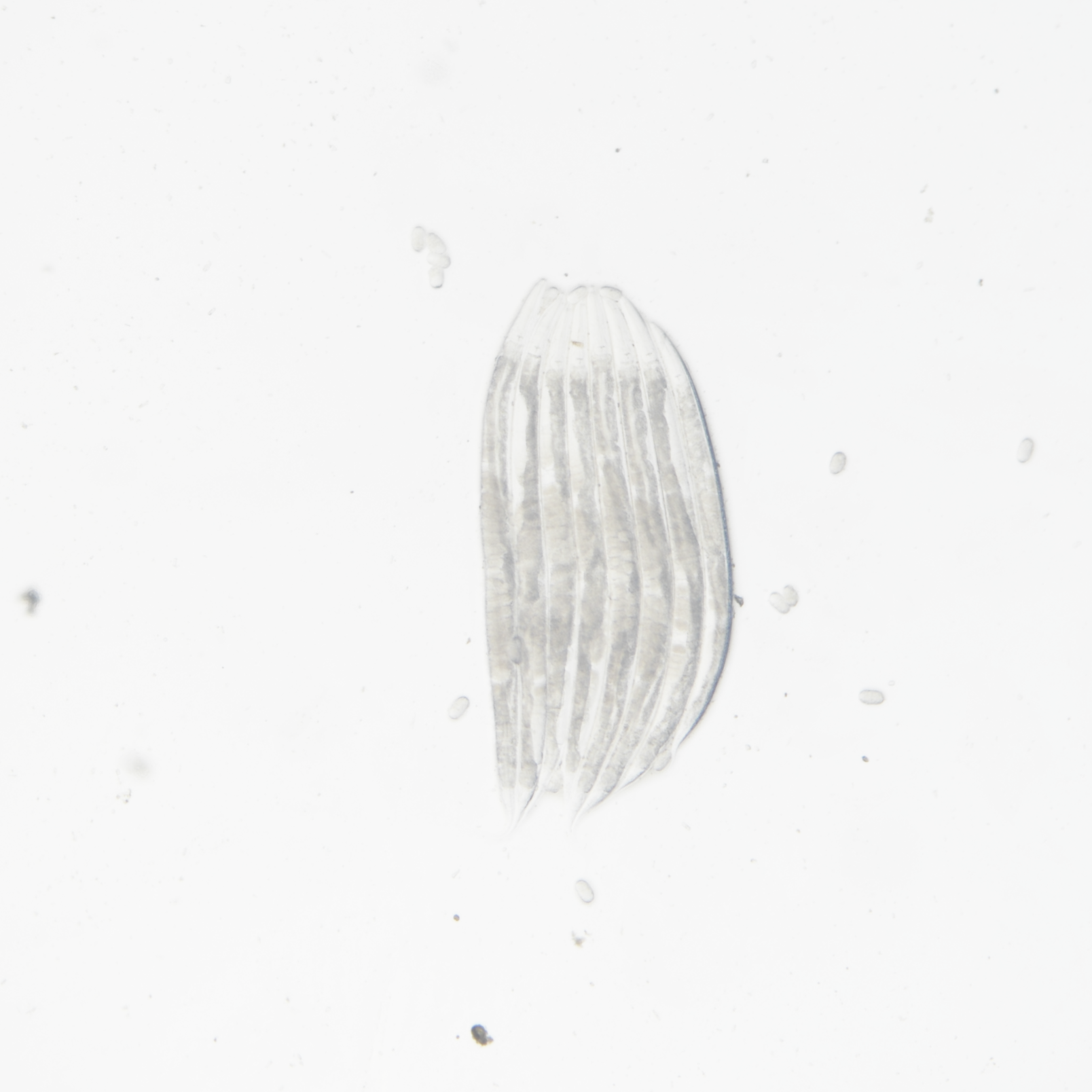

Supplement: Supplementary file 7 — Source data Fig. 4 [file 44318_2025_619_MOESM7_ESM.zip › Figure 4/4D/g.tif]

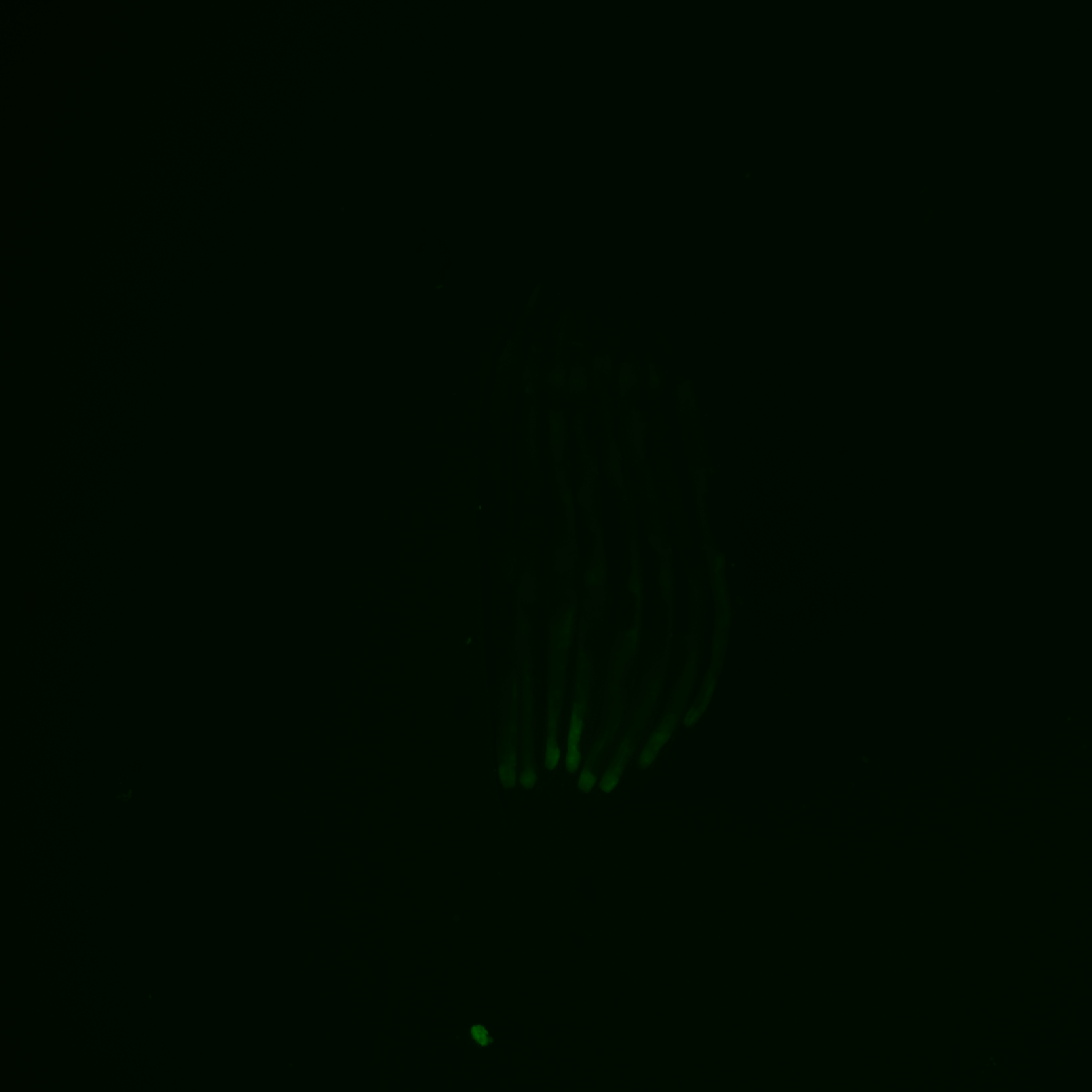

Supplement: Supplementary file 7 — Source data Fig. 4 [file 44318_2025_619_MOESM7_ESM.zip › Figure 4/4D/h.tif]

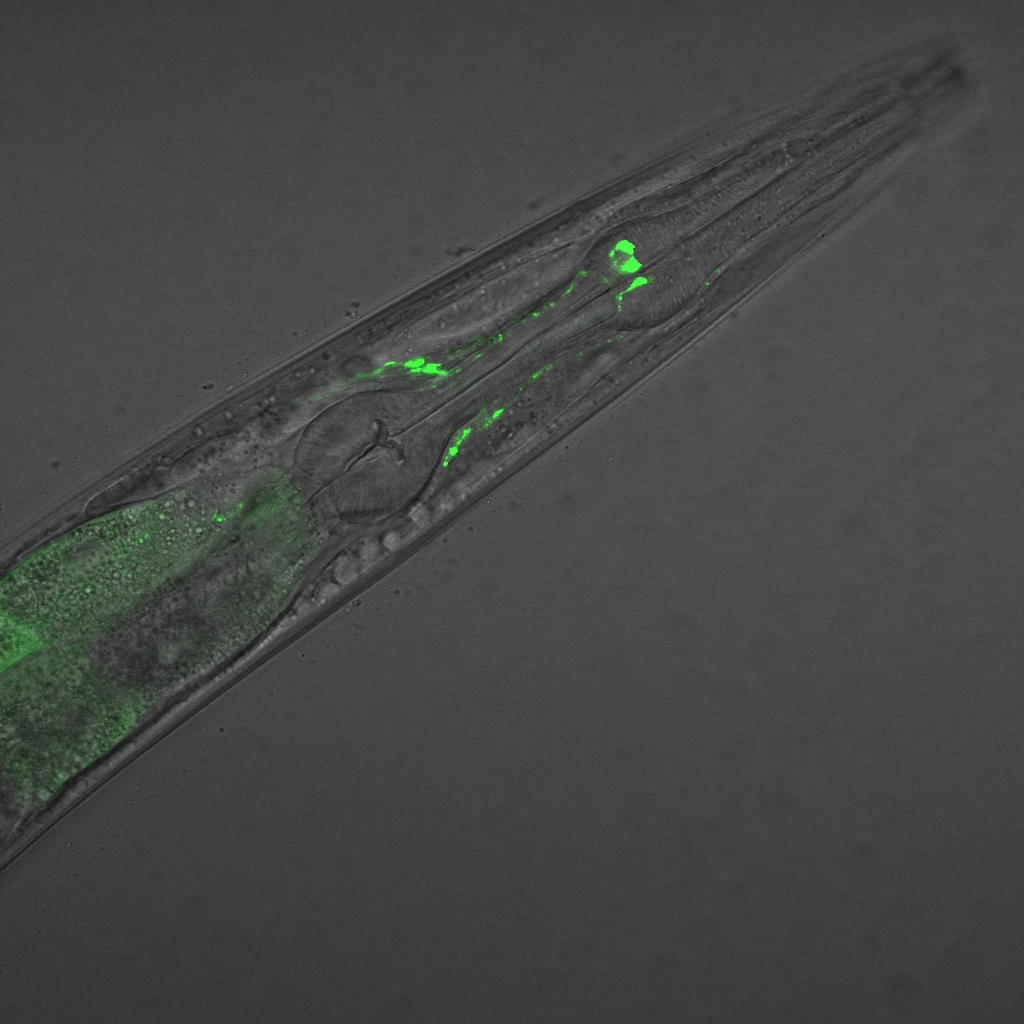

Supplement: Supplementary file 7 — Source data Fig. 4 [file 44318_2025_619_MOESM7_ESM.zip › Figure 4/4F/a.tif]

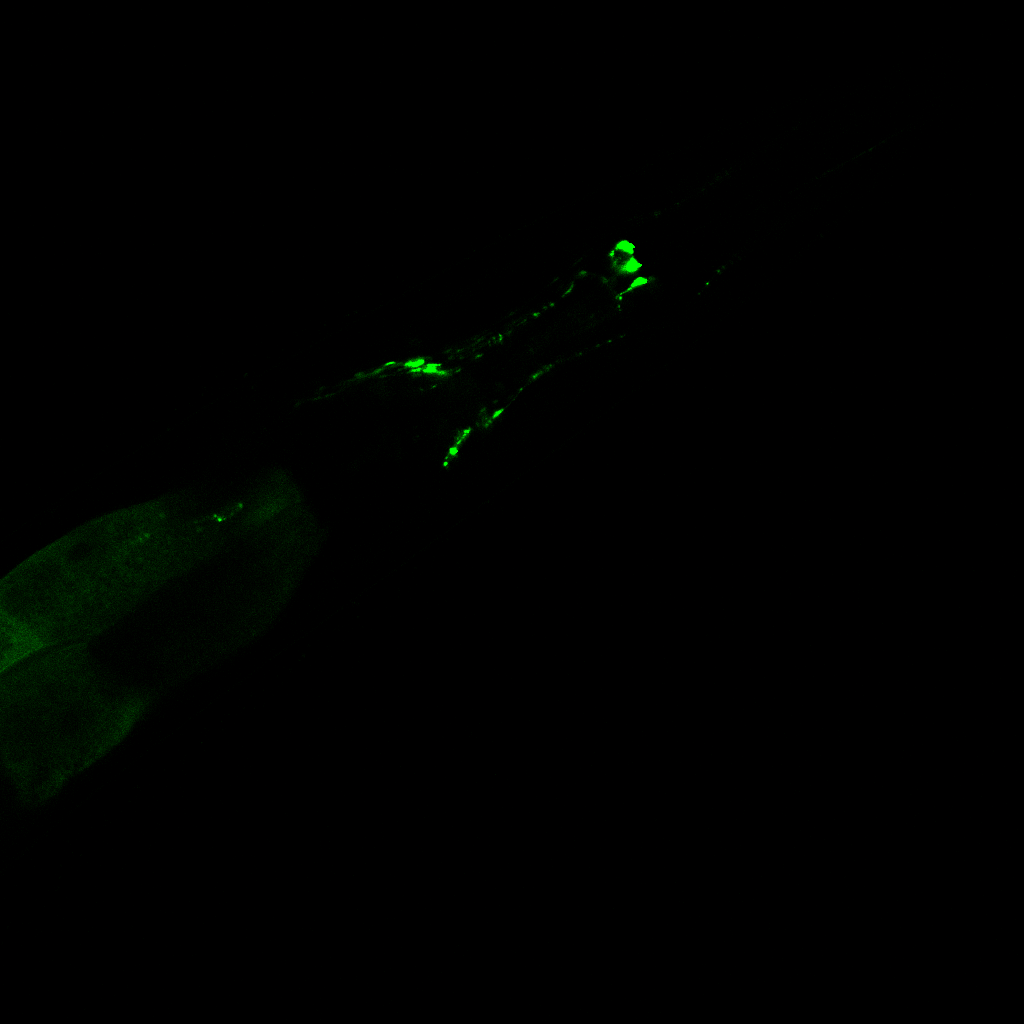

Supplement: Supplementary file 7 — Source data Fig. 4 [file 44318_2025_619_MOESM7_ESM.zip › Figure 4/4F/b.tif]

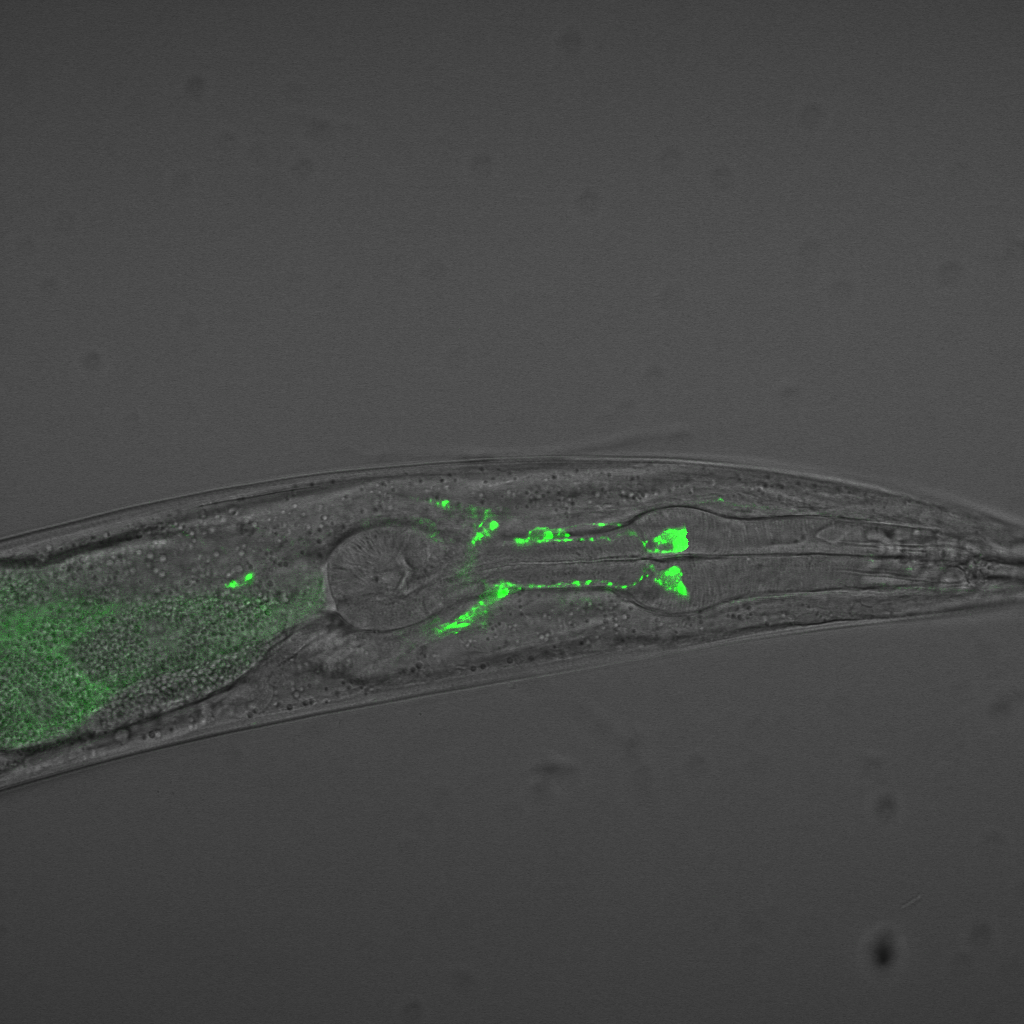

Supplement: Supplementary file 7 — Source data Fig. 4 [file 44318_2025_619_MOESM7_ESM.zip › Figure 4/4F/c.tif]

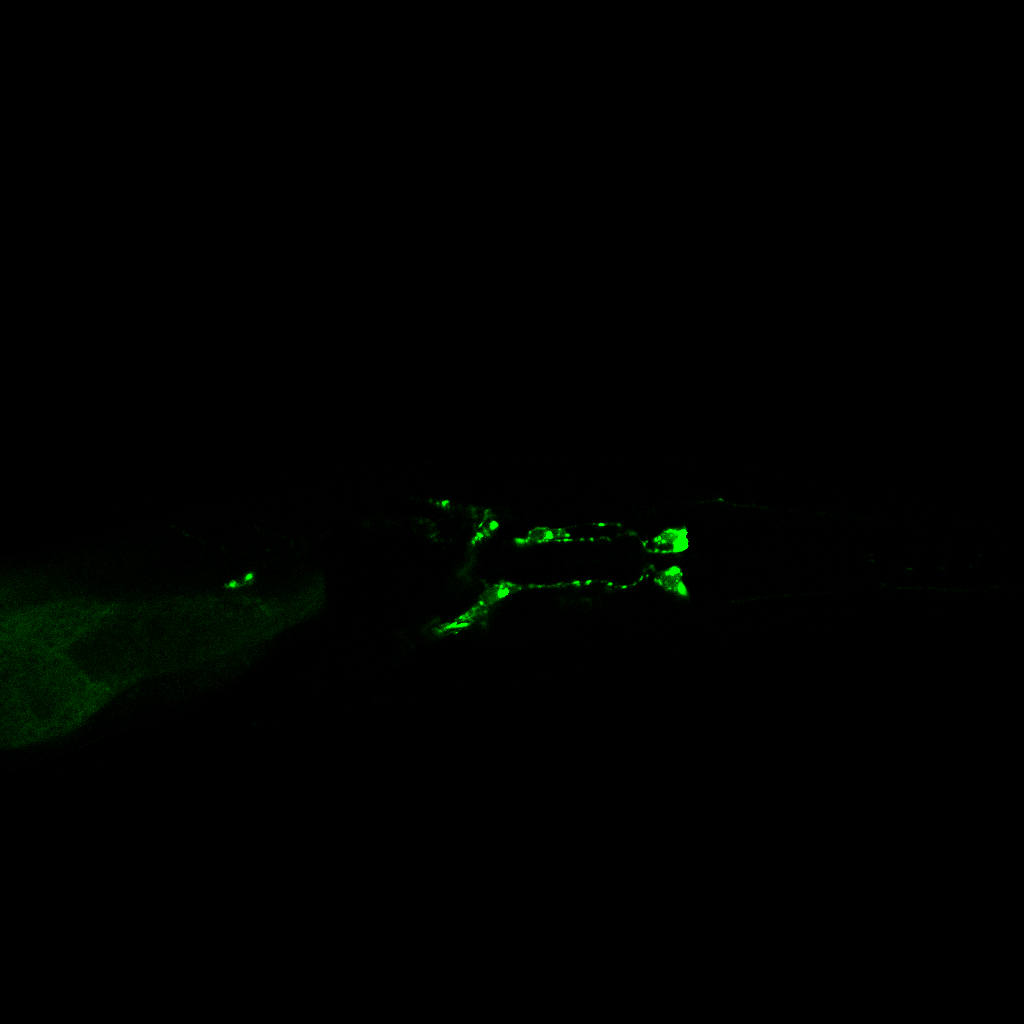

Supplement: Supplementary file 7 — Source data Fig. 4 [file 44318_2025_619_MOESM7_ESM.zip › Figure 4/4F/d.tif]

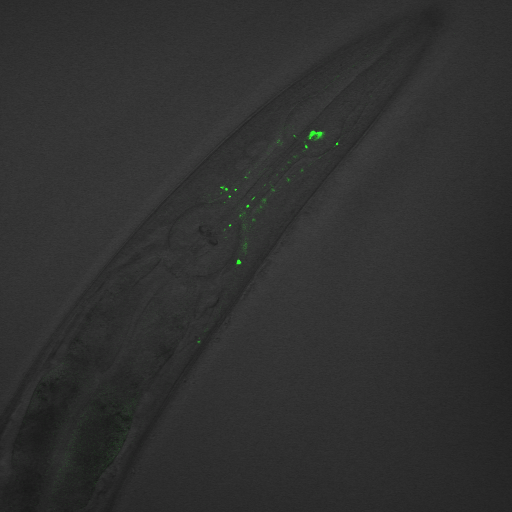

Supplement: Supplementary file 8 — Source data Fig. 5 [file 44318_2025_619_MOESM8_ESM.zip › Figure 5/5B/a.tif]

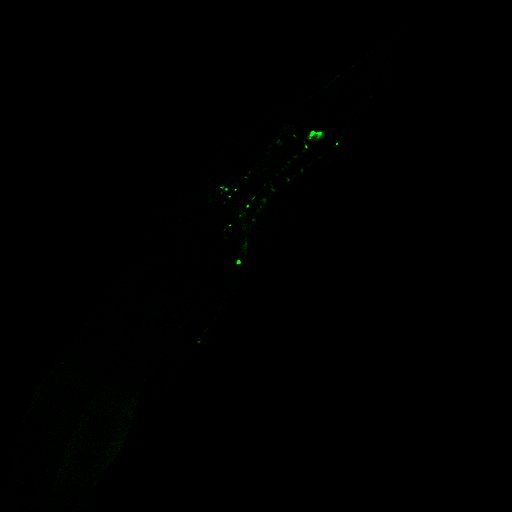

Supplement: Supplementary file 8 — Source data Fig. 5 [file 44318_2025_619_MOESM8_ESM.zip › Figure 5/5B/b.tif]

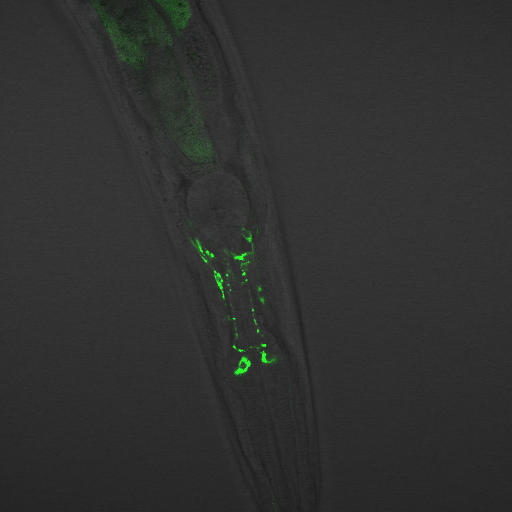

Supplement: Supplementary file 8 — Source data Fig. 5 [file 44318_2025_619_MOESM8_ESM.zip › Figure 5/5B/c.tif]

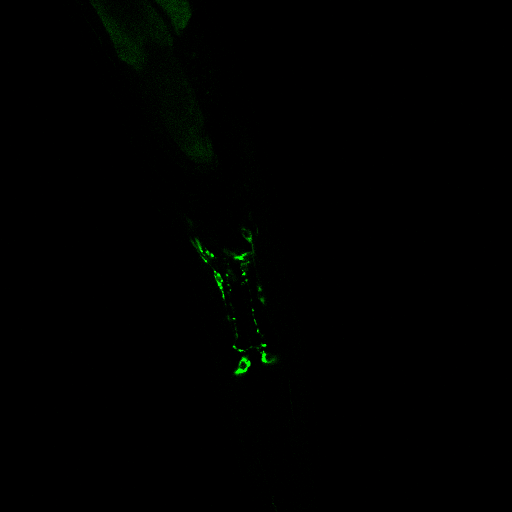

Supplement: Supplementary file 8 — Source data Fig. 5 [file 44318_2025_619_MOESM8_ESM.zip › Figure 5/5B/d.tif]

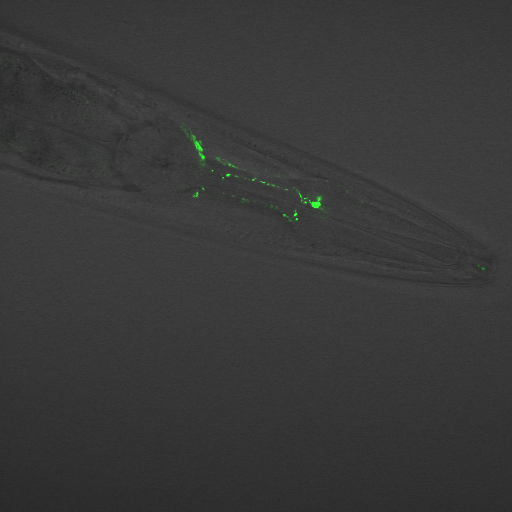

Supplement: Supplementary file 8 — Source data Fig. 5 [file 44318_2025_619_MOESM8_ESM.zip › Figure 5/5B/e.tif]

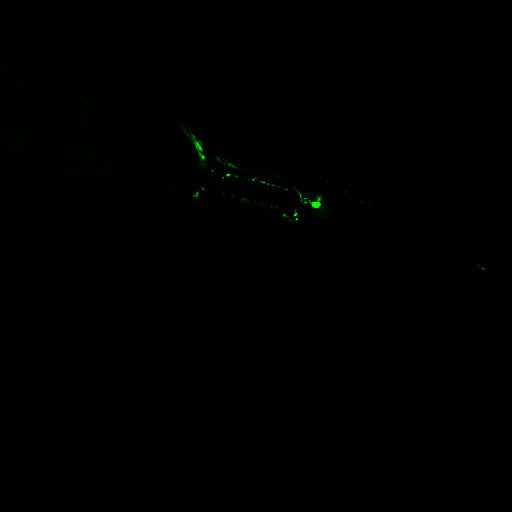

Supplement: Supplementary file 8 — Source data Fig. 5 [file 44318_2025_619_MOESM8_ESM.zip › Figure 5/5B/f.tif]

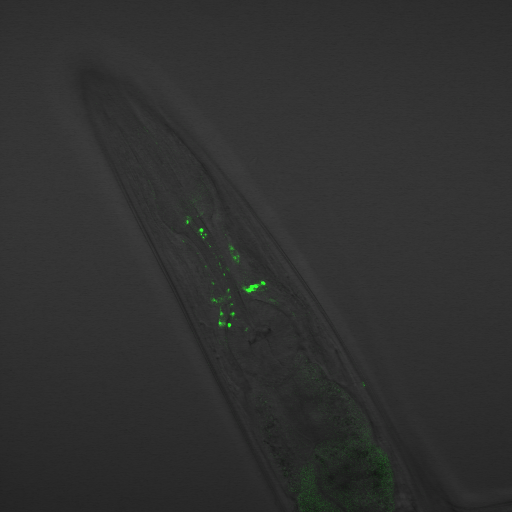

Supplement: Supplementary file 8 — Source data Fig. 5 [file 44318_2025_619_MOESM8_ESM.zip › Figure 5/5B/g.tif]

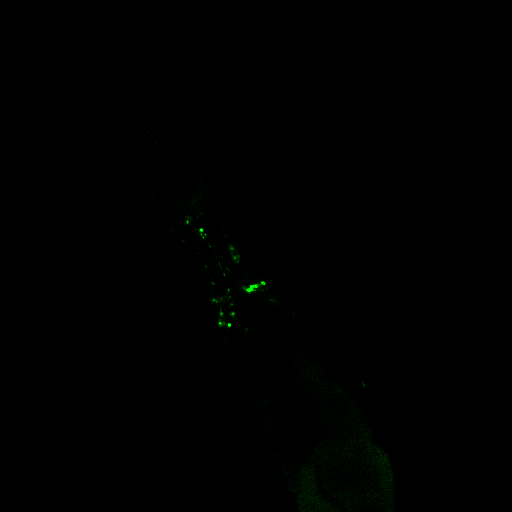

Supplement: Supplementary file 8 — Source data Fig. 5 [file 44318_2025_619_MOESM8_ESM.zip › Figure 5/5B/h.tif]

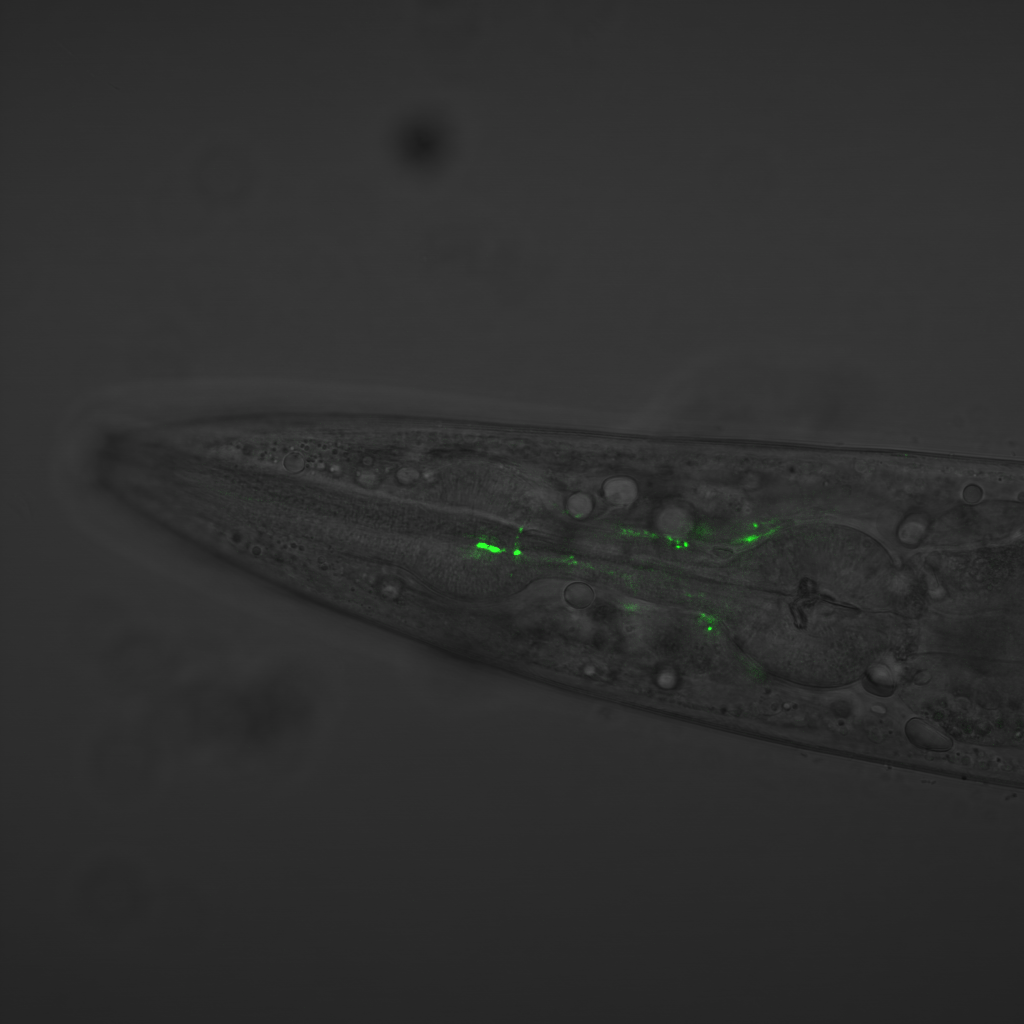

Supplement: Supplementary file 8 — Source data Fig. 5 [file 44318_2025_619_MOESM8_ESM.zip › Figure 5/5D/a.tif]

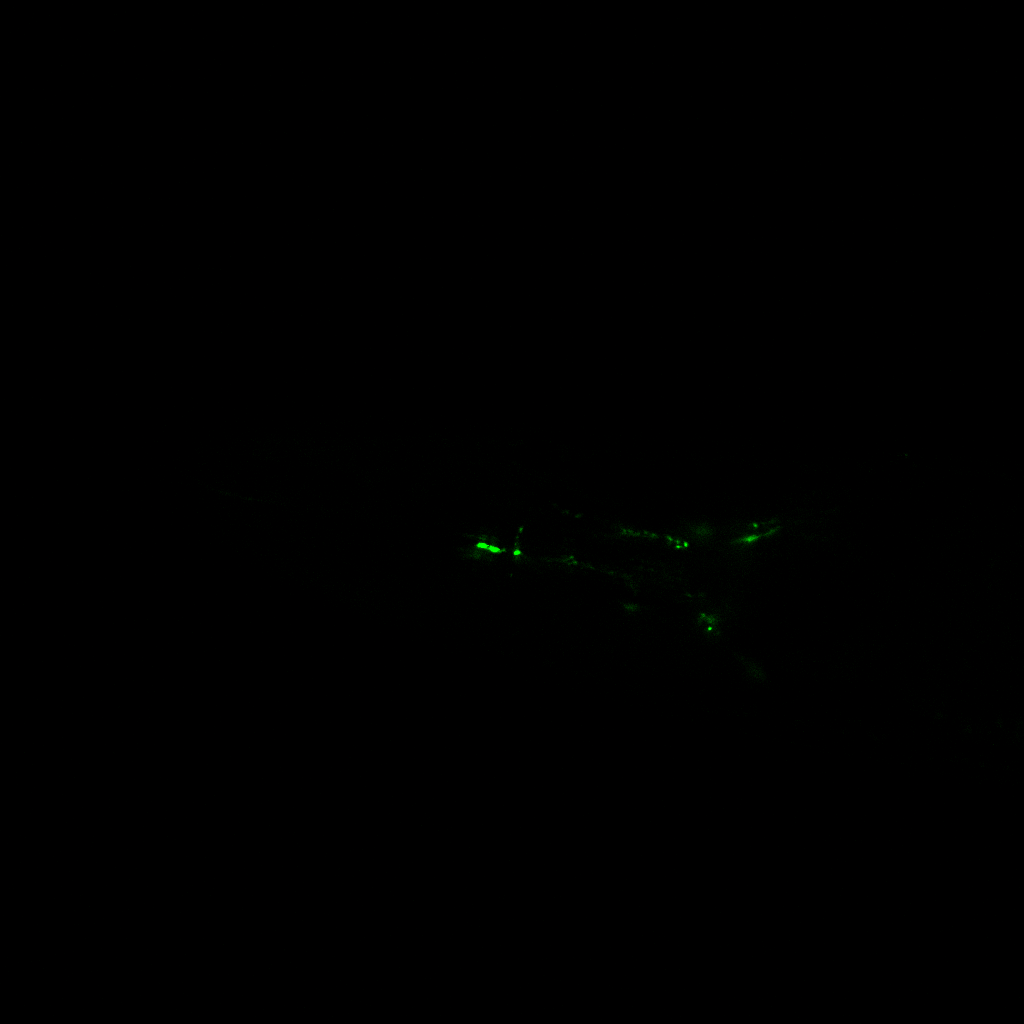

Supplement: Supplementary file 8 — Source data Fig. 5 [file 44318_2025_619_MOESM8_ESM.zip › Figure 5/5D/b.tif]

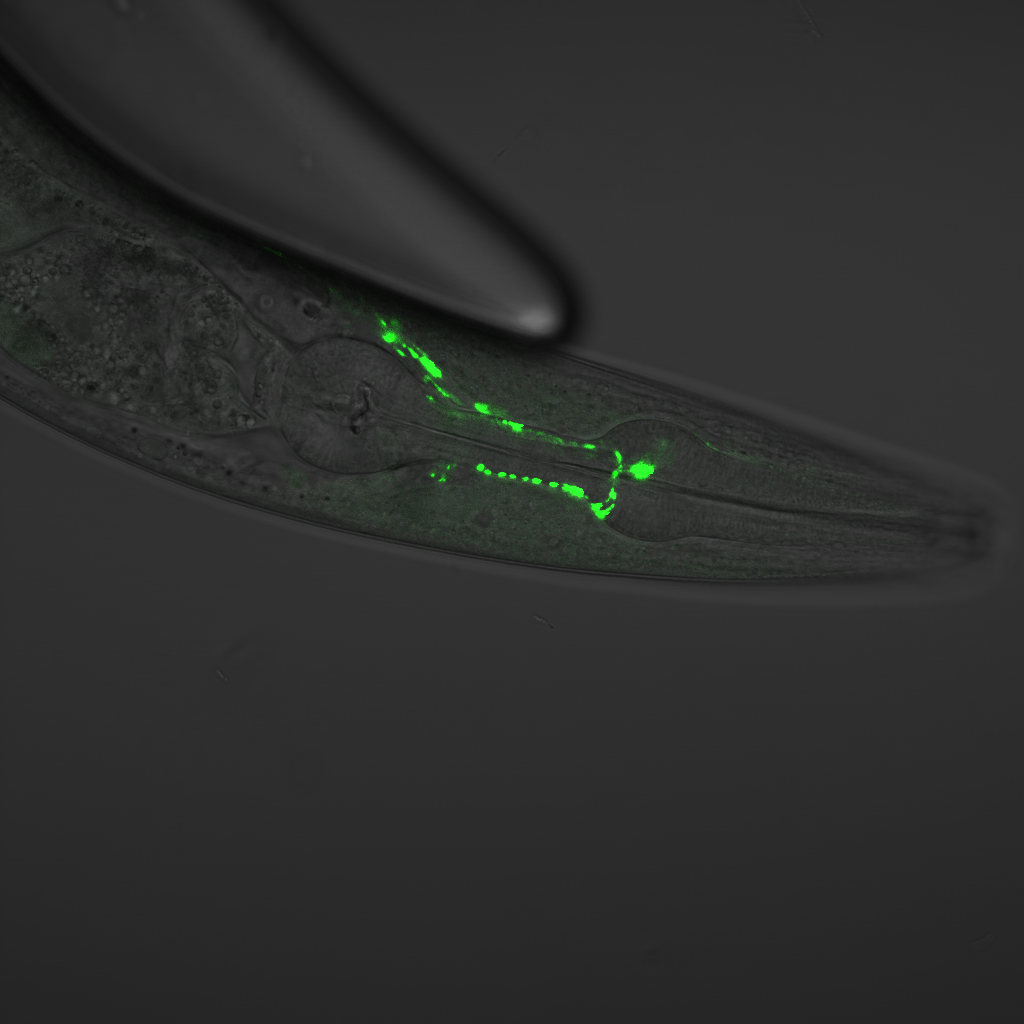

Supplement: Supplementary file 8 — Source data Fig. 5 [file 44318_2025_619_MOESM8_ESM.zip › Figure 5/5D/c.tif]

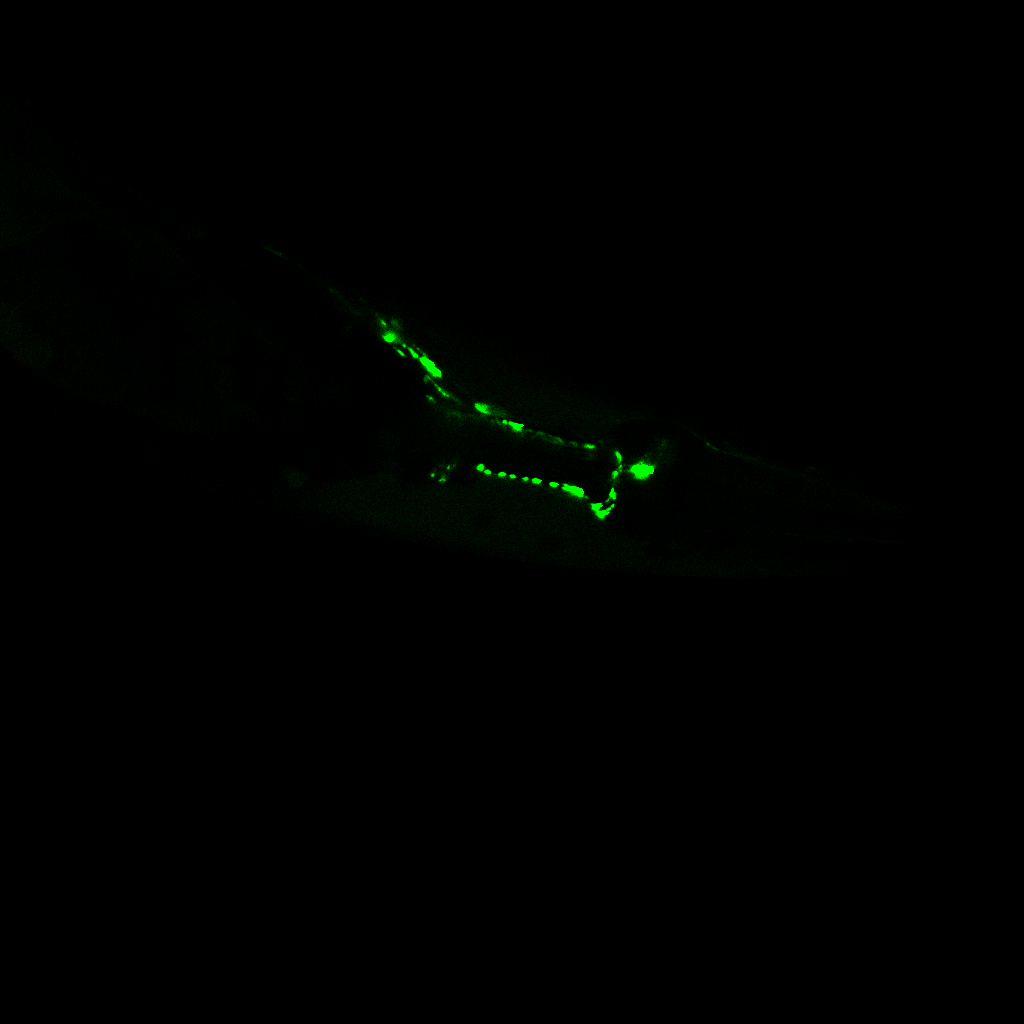

Supplement: Supplementary file 8 — Source data Fig. 5 [file 44318_2025_619_MOESM8_ESM.zip › Figure 5/5D/d.tif]

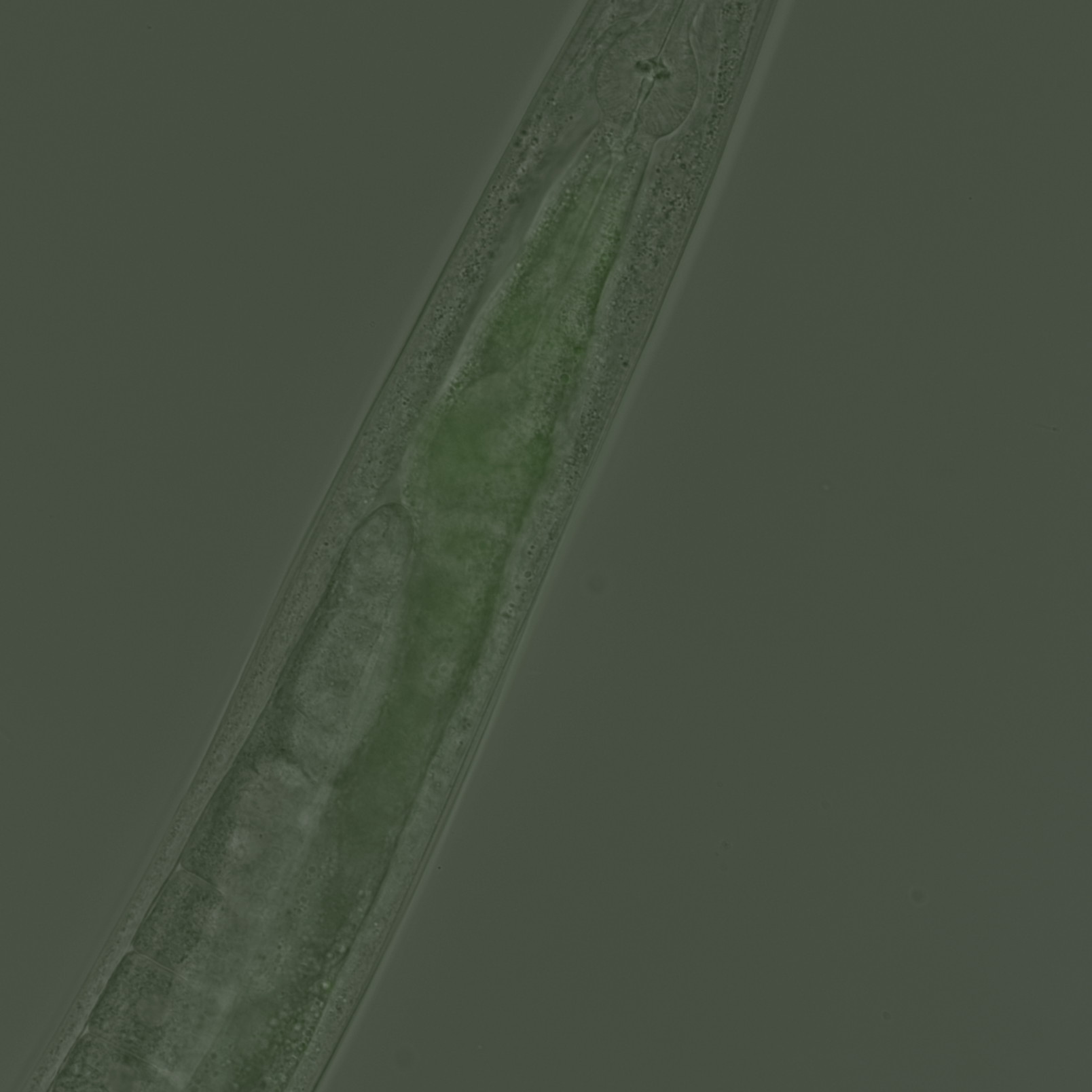

Supplement: Supplementary file 8 — Source data Fig. 5 [file 44318_2025_619_MOESM8_ESM.zip › Figure 5/5F/a.tif]

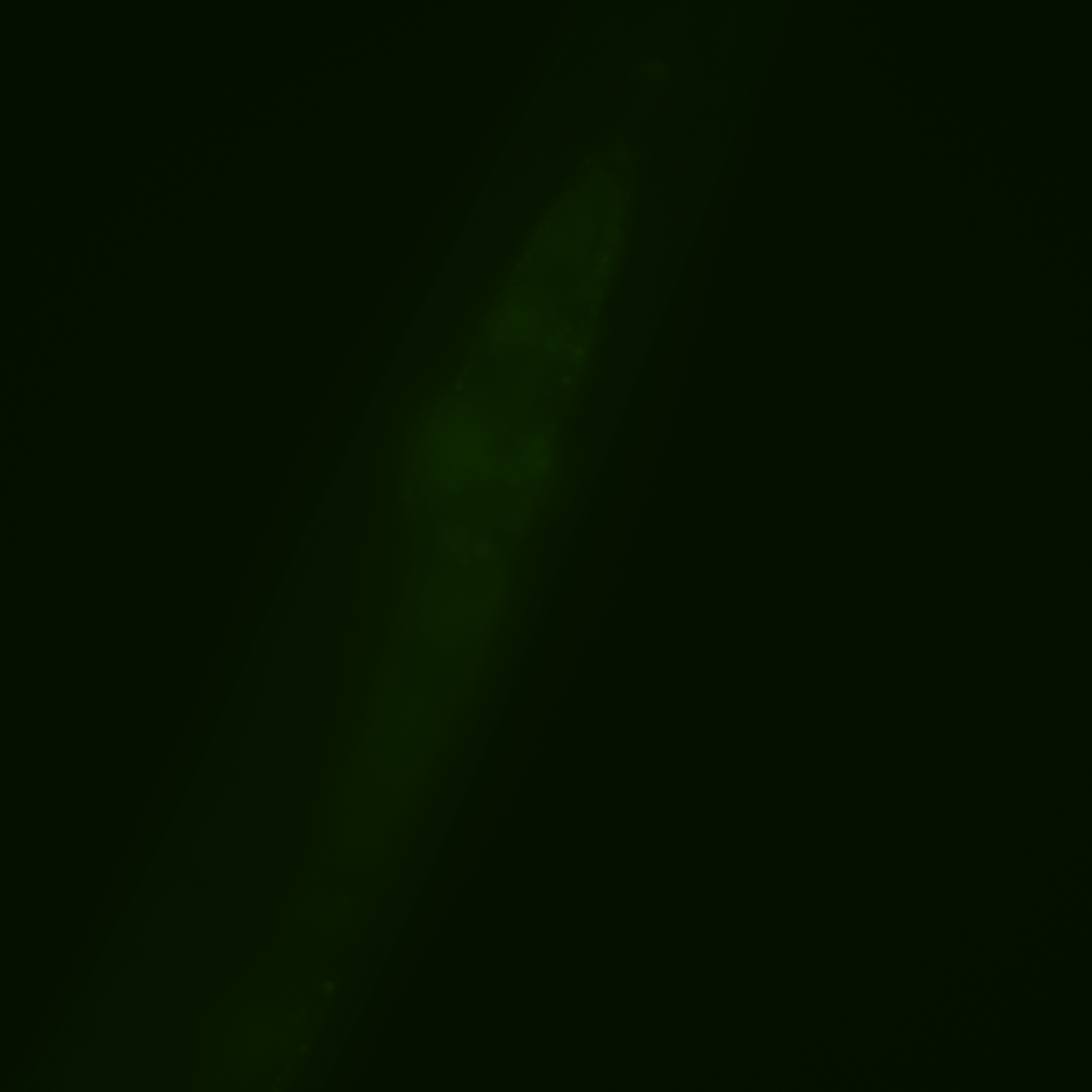

Supplement: Supplementary file 8 — Source data Fig. 5 [file 44318_2025_619_MOESM8_ESM.zip › Figure 5/5F/b.tif]

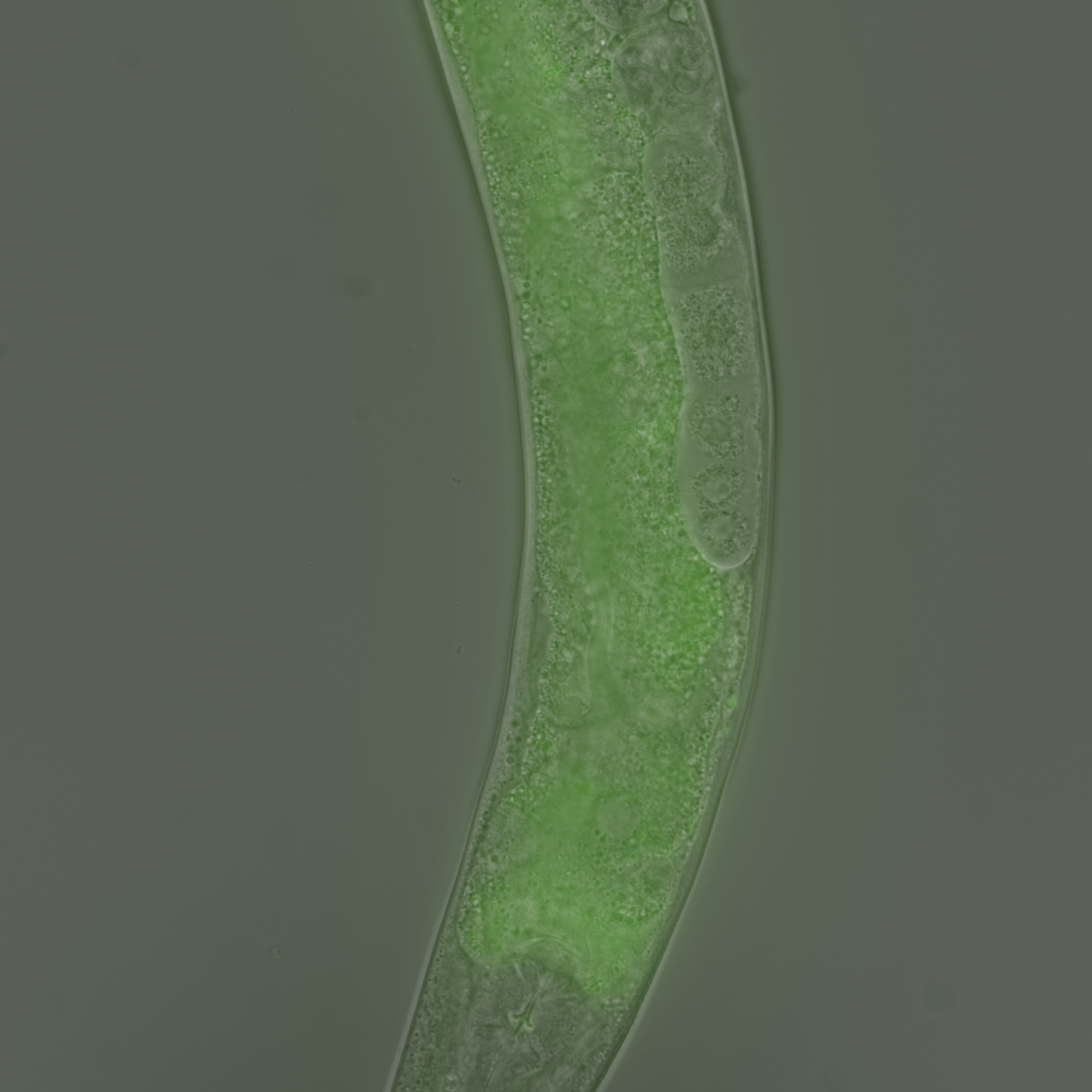

Supplement: Supplementary file 8 — Source data Fig. 5 [file 44318_2025_619_MOESM8_ESM.zip › Figure 5/5F/c.tif]

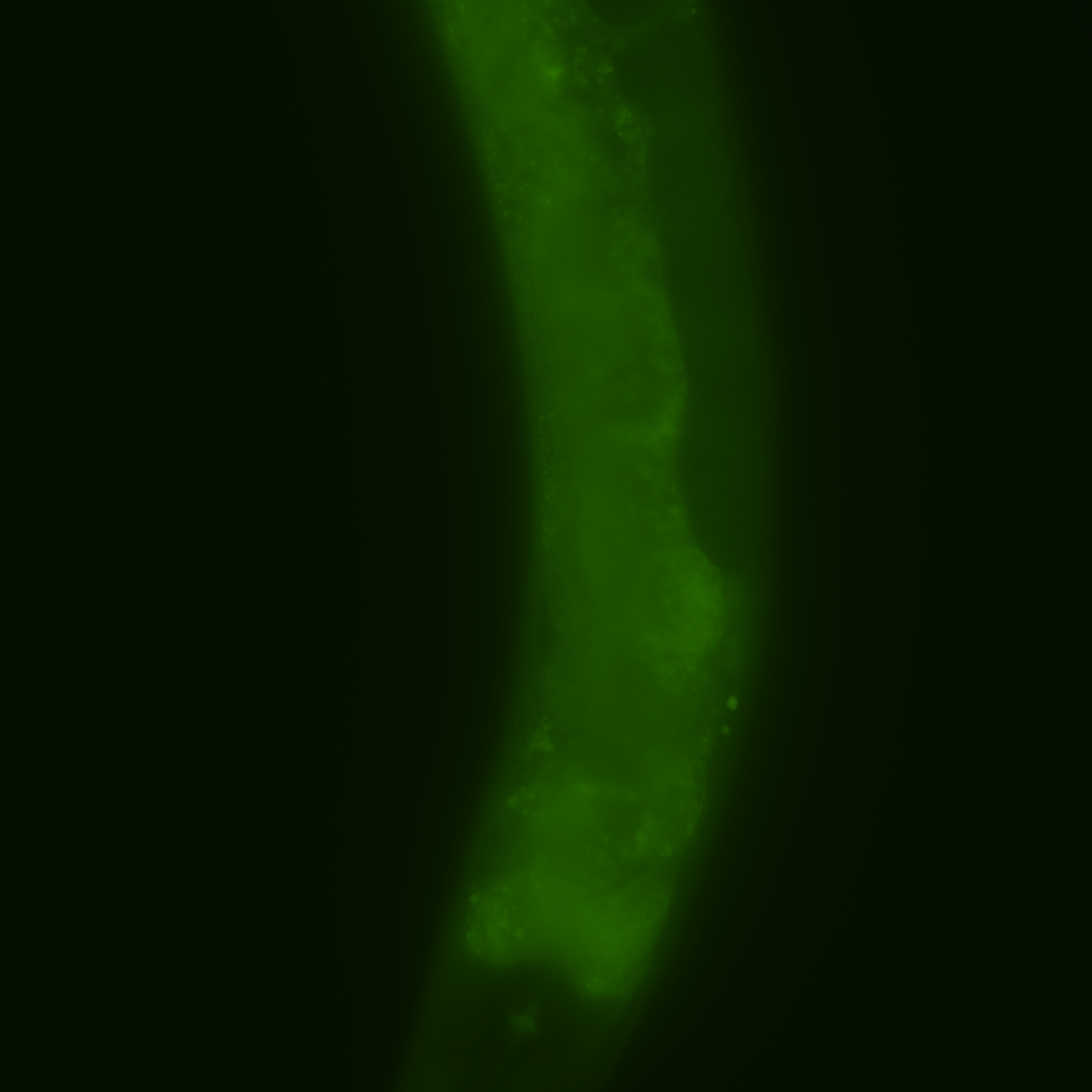

Supplement: Supplementary file 8 — Source data Fig. 5 [file 44318_2025_619_MOESM8_ESM.zip › Figure 5/5F/d.tif]

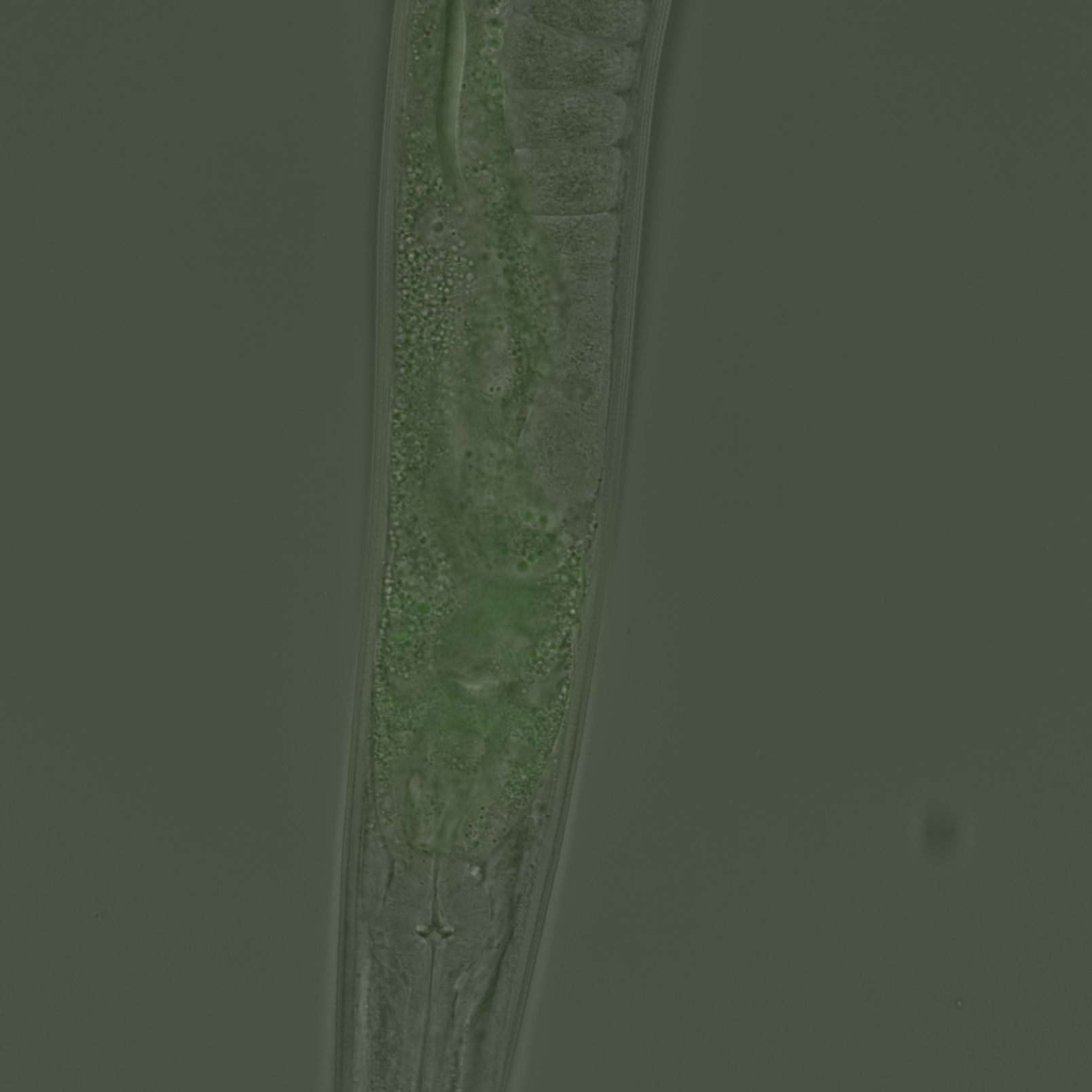

Supplement: Supplementary file 8 — Source data Fig. 5 [file 44318_2025_619_MOESM8_ESM.zip › Figure 5/5F/e.tif]

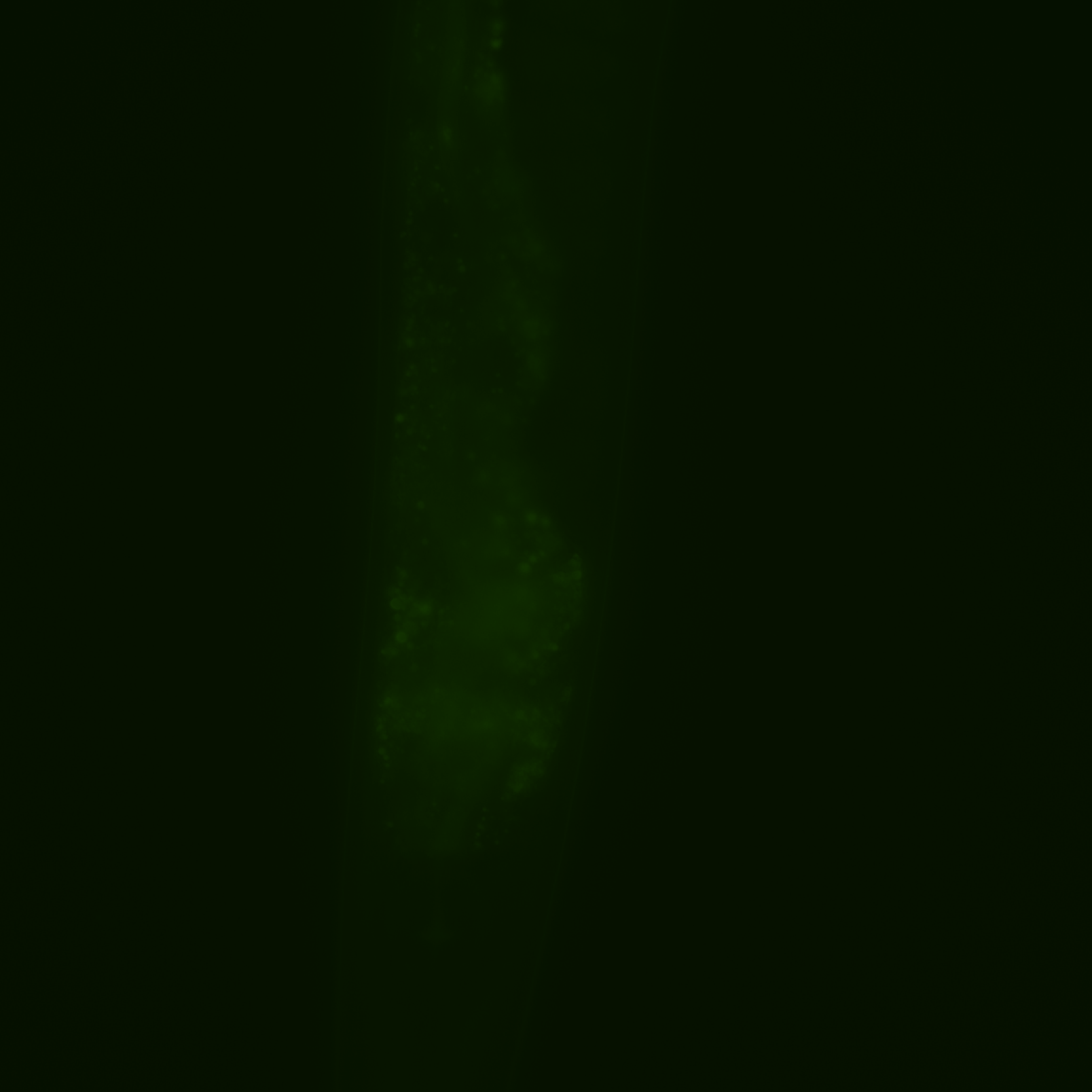

Supplement: Supplementary file 8 — Source data Fig. 5 [file 44318_2025_619_MOESM8_ESM.zip › Figure 5/5F/f.tif]
