# Supplementary material for: A gut-brain-gut axis orchestrates host responses counteracting microbiome-induced iron insufficiency
Source: EMBO J. 2025 Nov 3;44(24):7590–619. doi: 10.1038/s44318-025-00619-6 (PMC12705764; doi:10.1038/s44318-025-00619-6)
Supplement: Supplementary file 5 — Source data Fig. 2 [file 44318_2025_619_MOESM5_ESM.zip › Figure 2/Instructions for reading the original data.docx]

**Methodology of analyzing fluorescence intensity of images by ImageJ:**

Step 1: Open the FITC image with ImageJ

Step 2: Convert the image to 8-bit

Step 3: Establish the threshold for detecting fluorescent intensity in the images by defining lower and upper limits, with the lower threshold set at a minimum value (e.g., 10) and the upper threshold fixed at 255.

Step 4: Measure the fluorescence intensity.

**Normalization Procedure:**

Step 1: Calculate the average fluorescence intensity of the control group

Step 2: Normalize the data by dividing each sample's raw ImageJ fluorescence value by the mean fluorescence value of the corresponding control group, yielding a ratio relative to the control.

Step 3: Multiply by one hundred percent to convert the ratios into percentages.

Note: When setting the threshold, it is essential to ensure accurate signal detection while minimizing both over-detection and under-detection. Images with low fluorescence intensity may produce mean values that reach the lower threshold (e.g., 10), which yields identical values in the dataset.
