# Supplementary figures and images for: A gut-brain-gut axis orchestrates host responses counteracting microbiome-induced iron insufficiency (part 2 of 2)
Source: EMBO J. 2025 Nov 3;44(24):7590–619. doi: 10.1038/s44318-025-00619-6 (PMC12705764; doi:10.1038/s44318-025-00619-6)

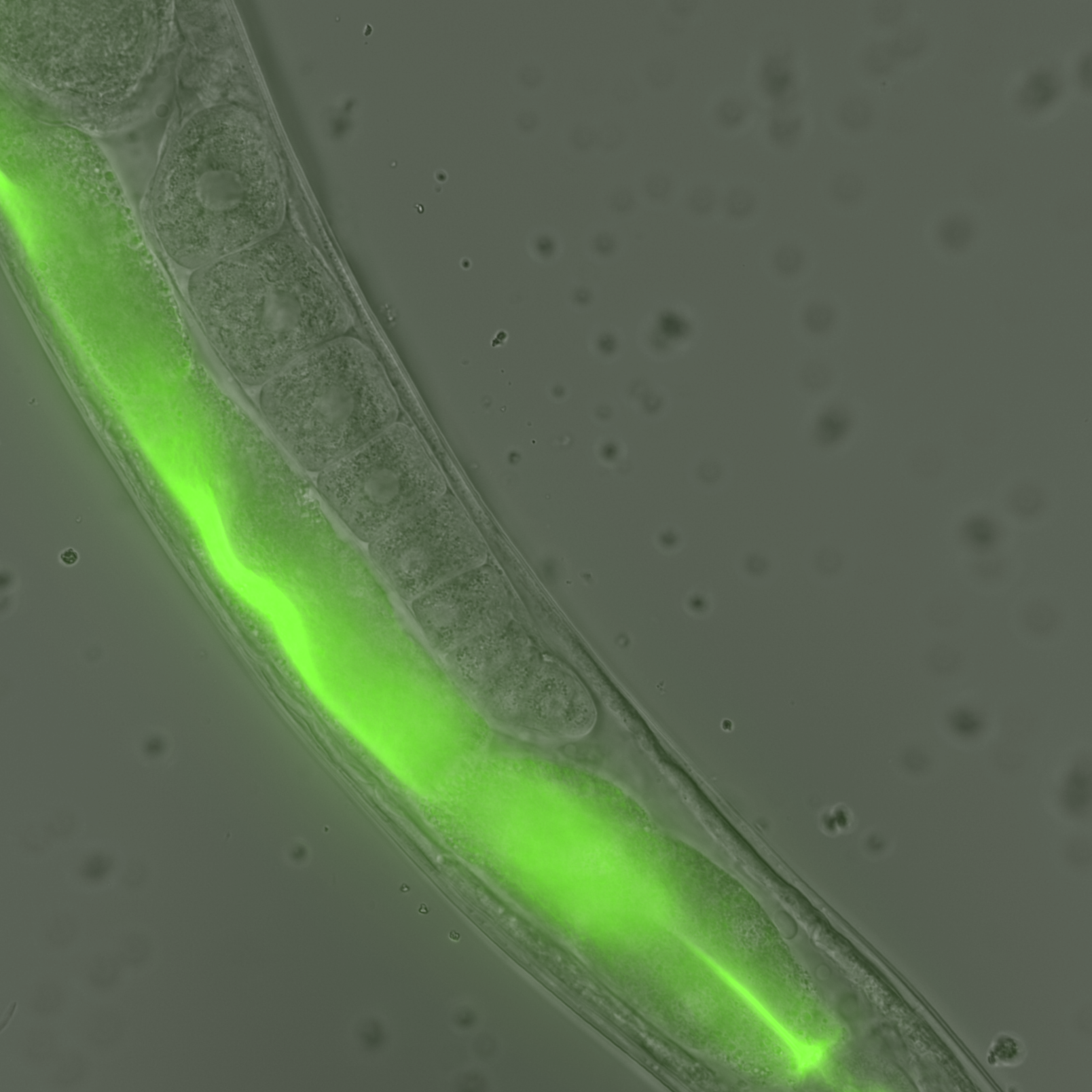

Supplement: Supplementary file 8 — Source data Fig. 5 [file 44318_2025_619_MOESM8_ESM.zip › Figure 5/5F/g.tif]

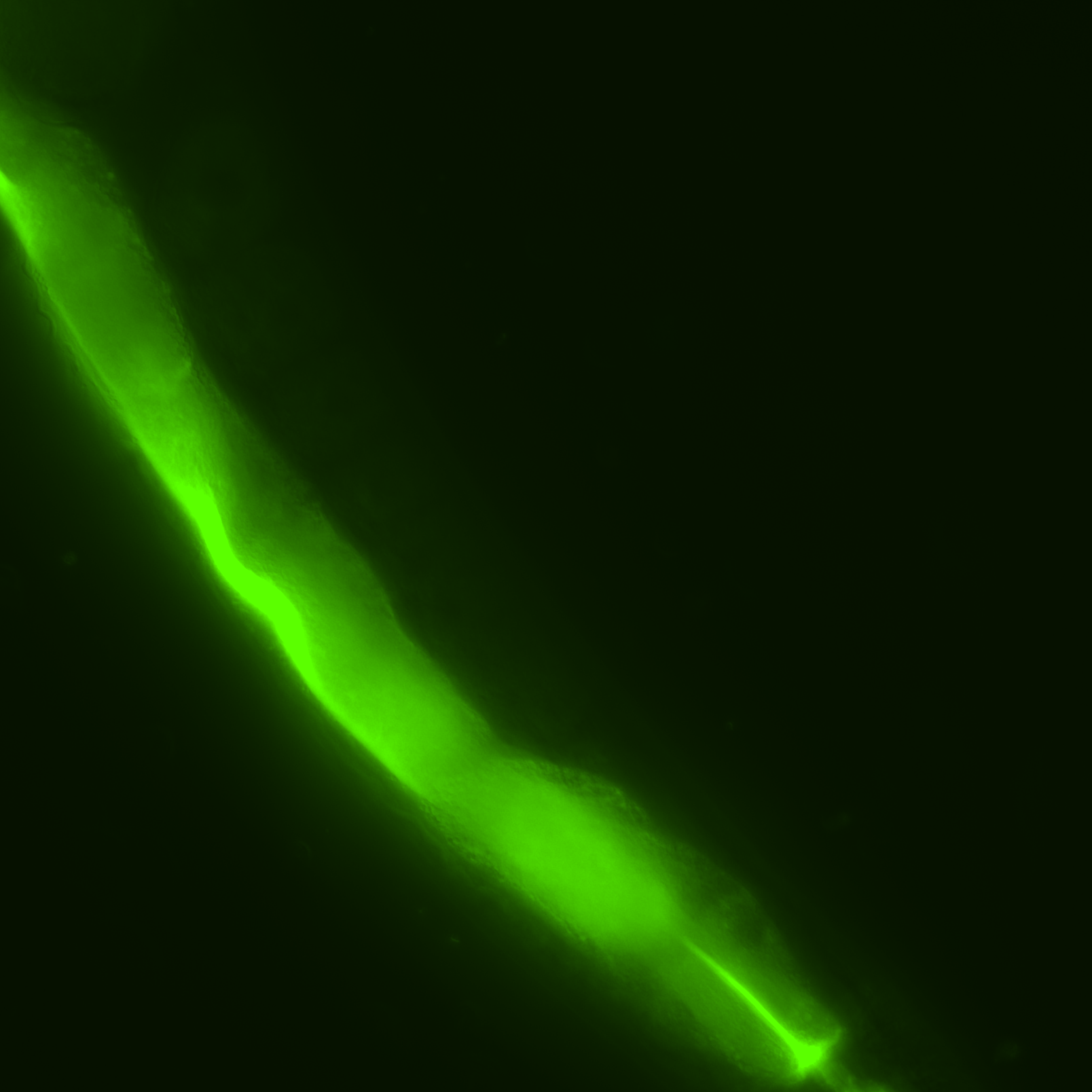

Supplement: Supplementary file 8 — Source data Fig. 5 [file 44318_2025_619_MOESM8_ESM.zip › Figure 5/5F/h.tif]

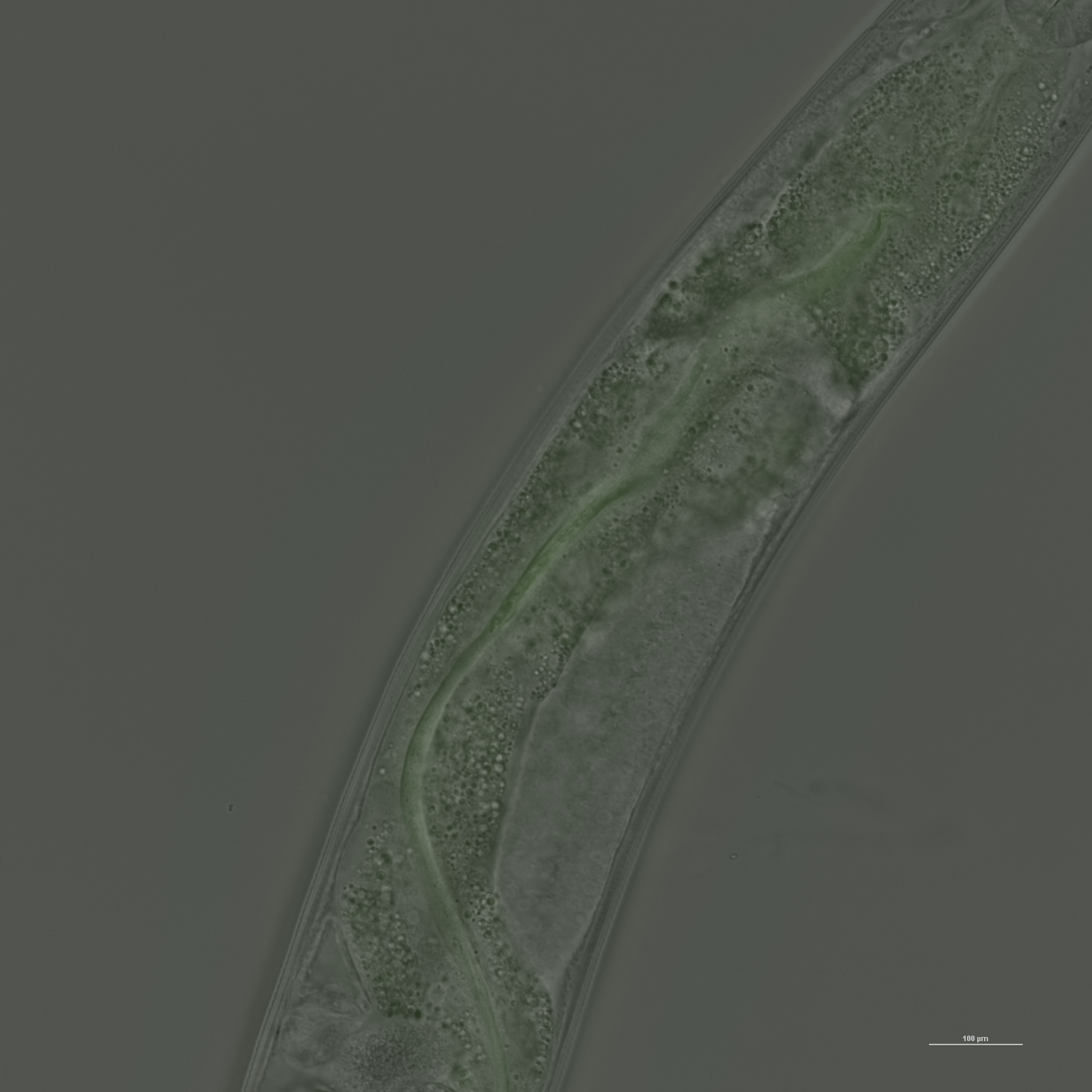

Supplement: Supplementary file 8 — Source data Fig. 5 [file 44318_2025_619_MOESM8_ESM.zip › Figure 5/5H/a.tif]

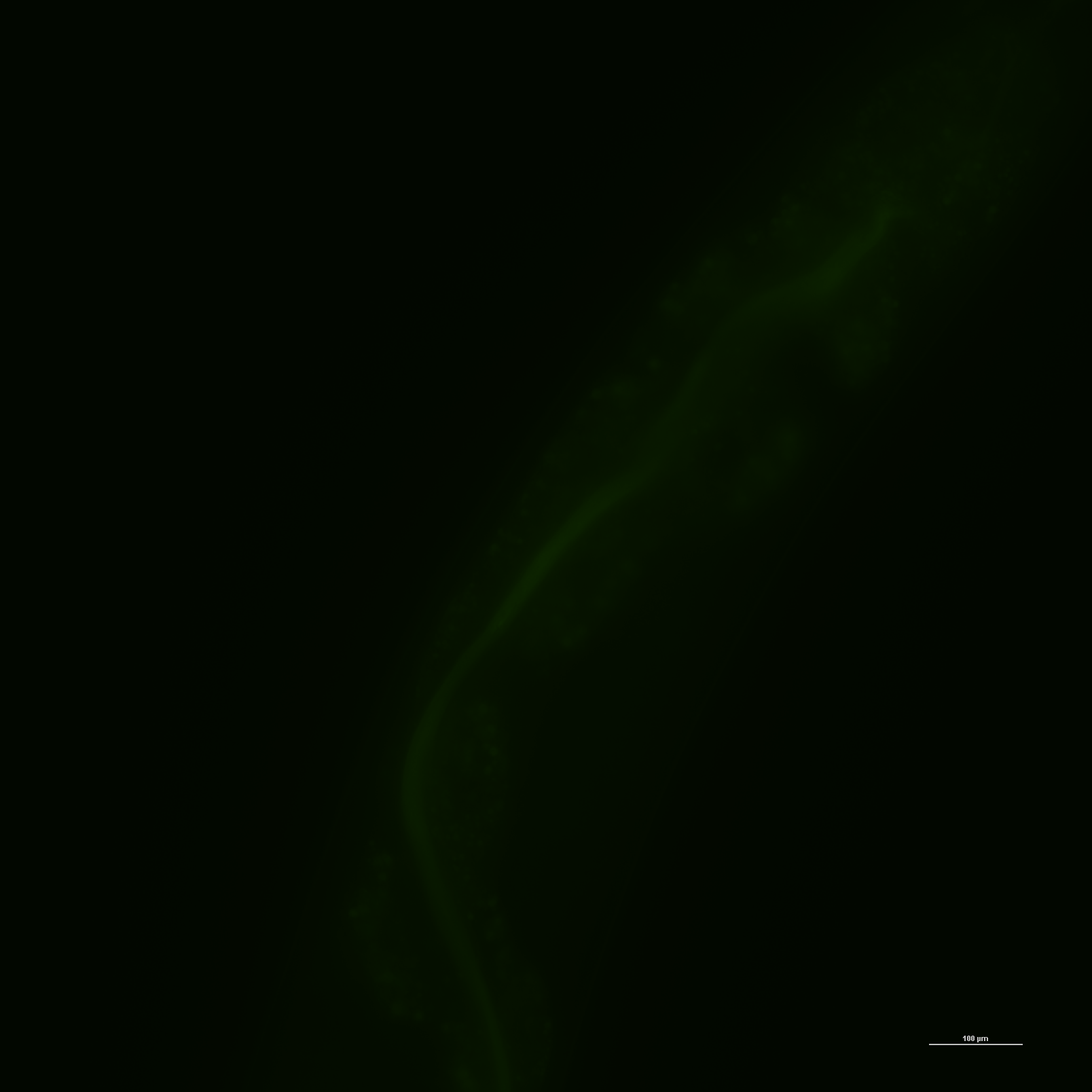

Supplement: Supplementary file 8 — Source data Fig. 5 [file 44318_2025_619_MOESM8_ESM.zip › Figure 5/5H/b.tif]

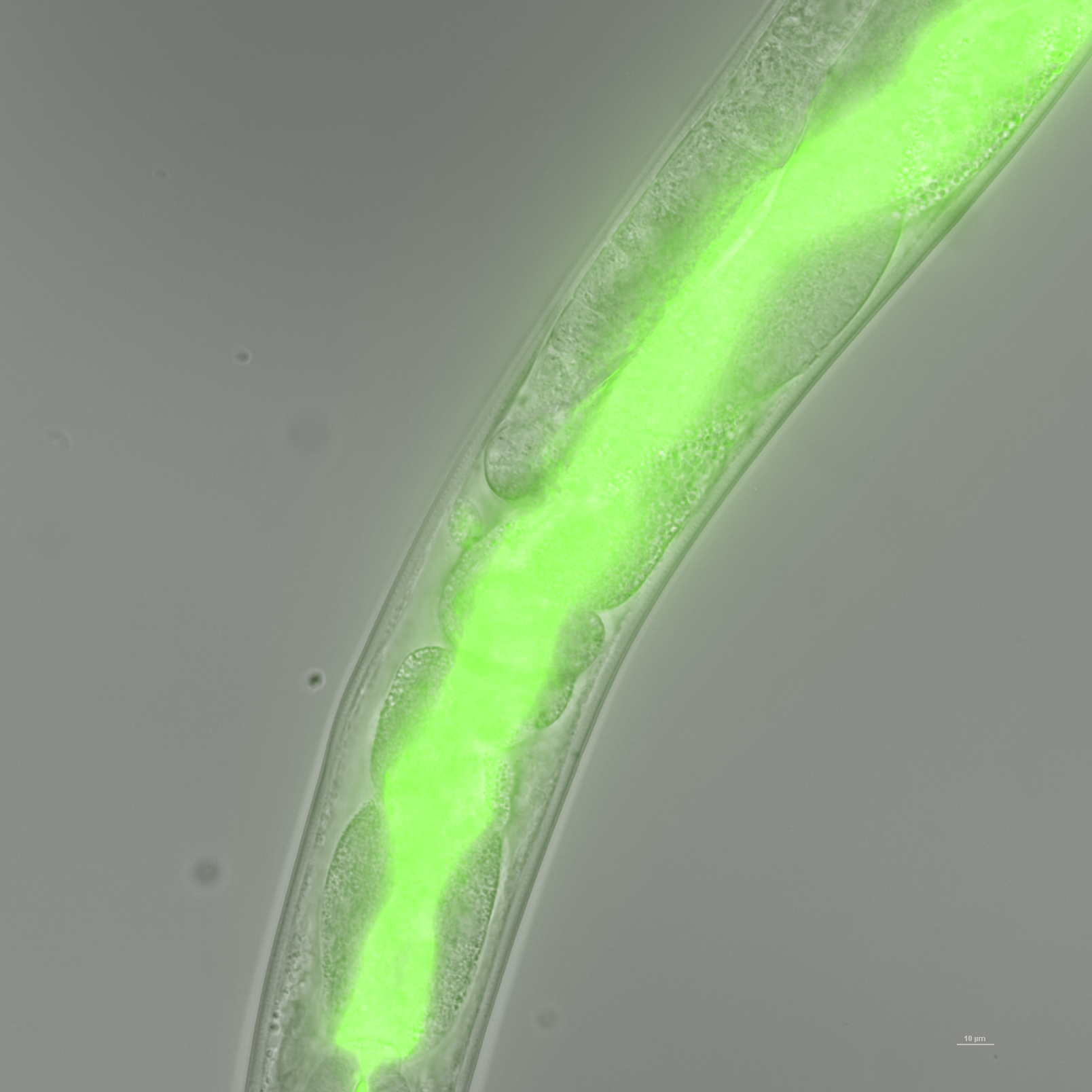

Supplement: Supplementary file 8 — Source data Fig. 5 [file 44318_2025_619_MOESM8_ESM.zip › Figure 5/5H/c.tif]

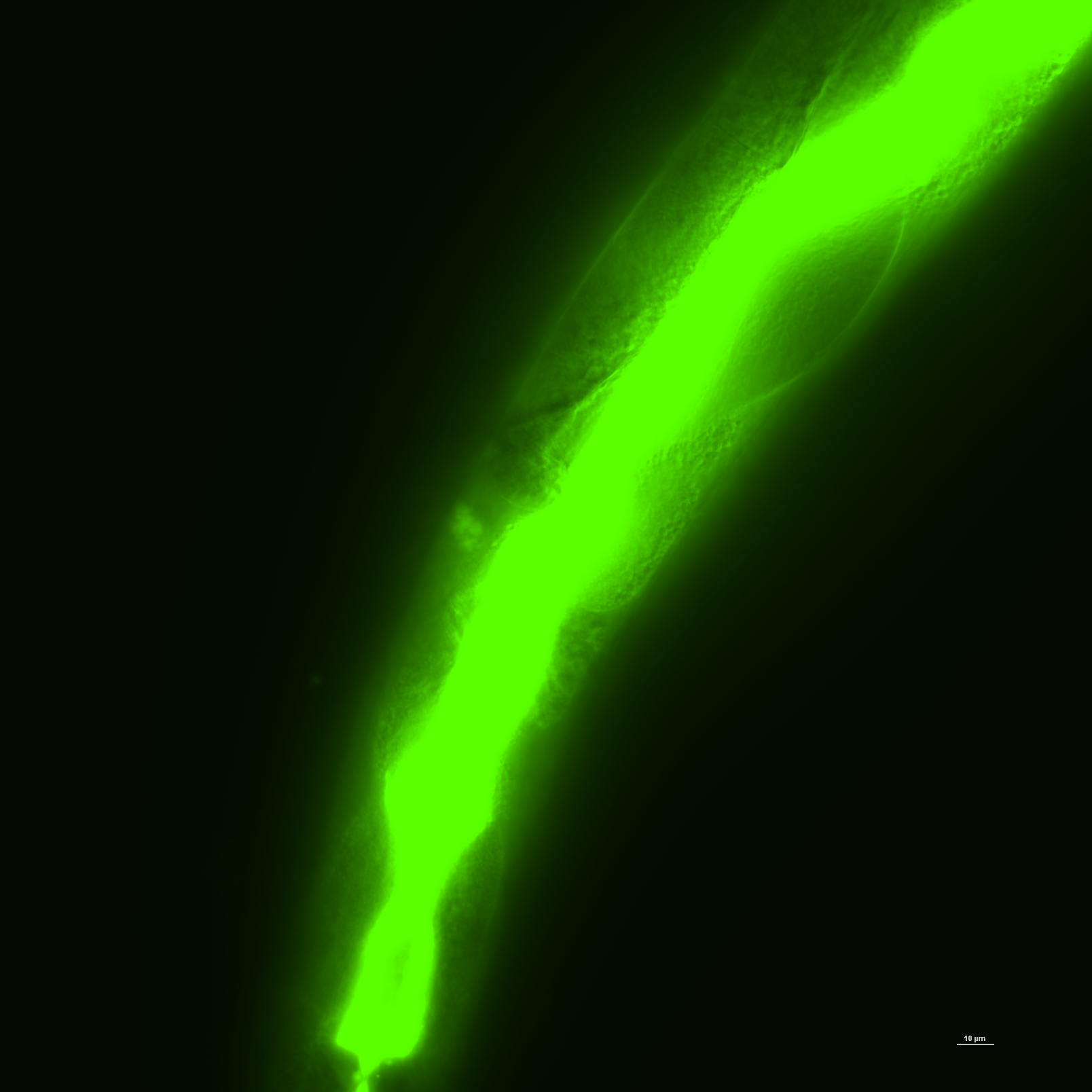

Supplement: Supplementary file 8 — Source data Fig. 5 [file 44318_2025_619_MOESM8_ESM.zip › Figure 5/5H/d.tif]

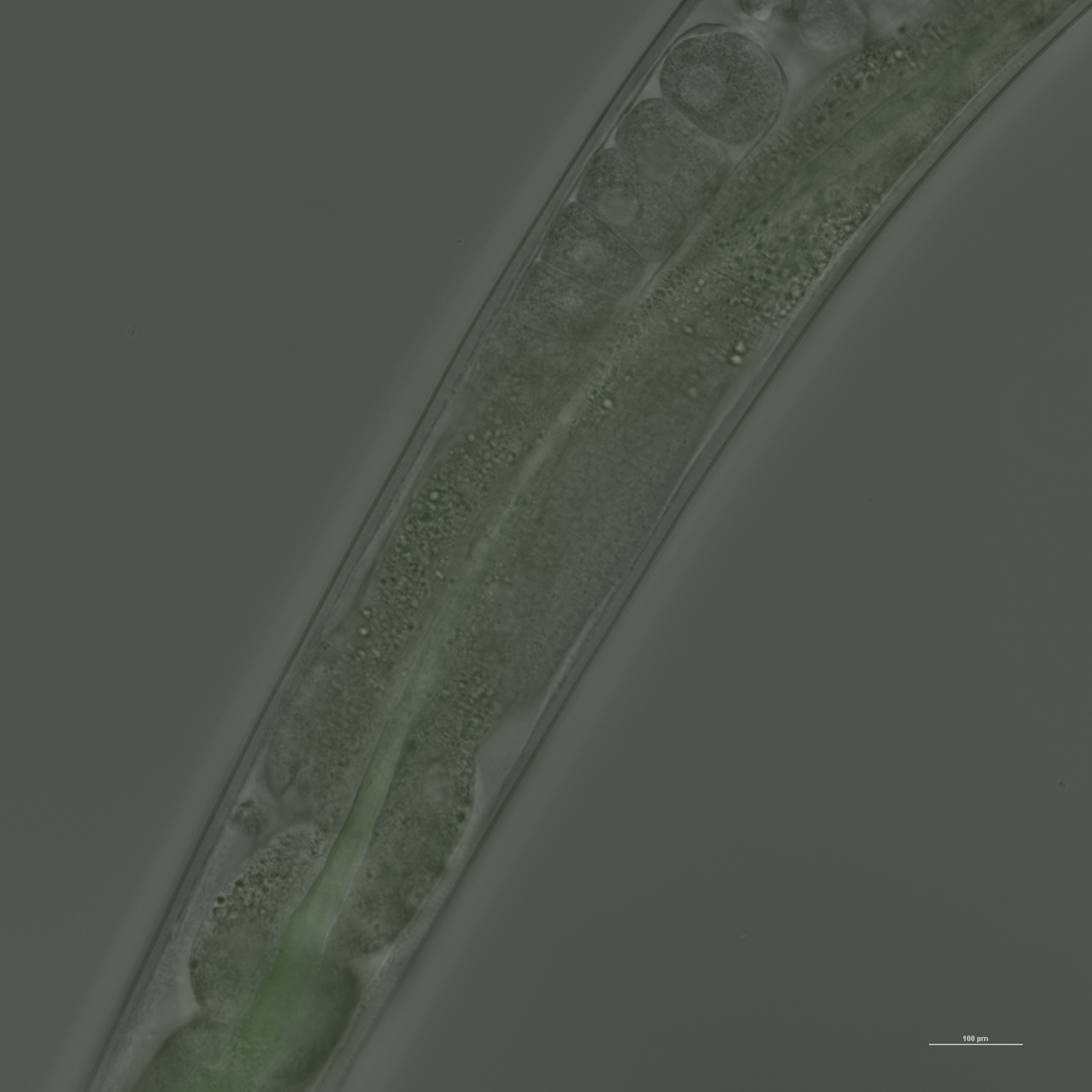

Supplement: Supplementary file 8 — Source data Fig. 5 [file 44318_2025_619_MOESM8_ESM.zip › Figure 5/5H/e.tif]

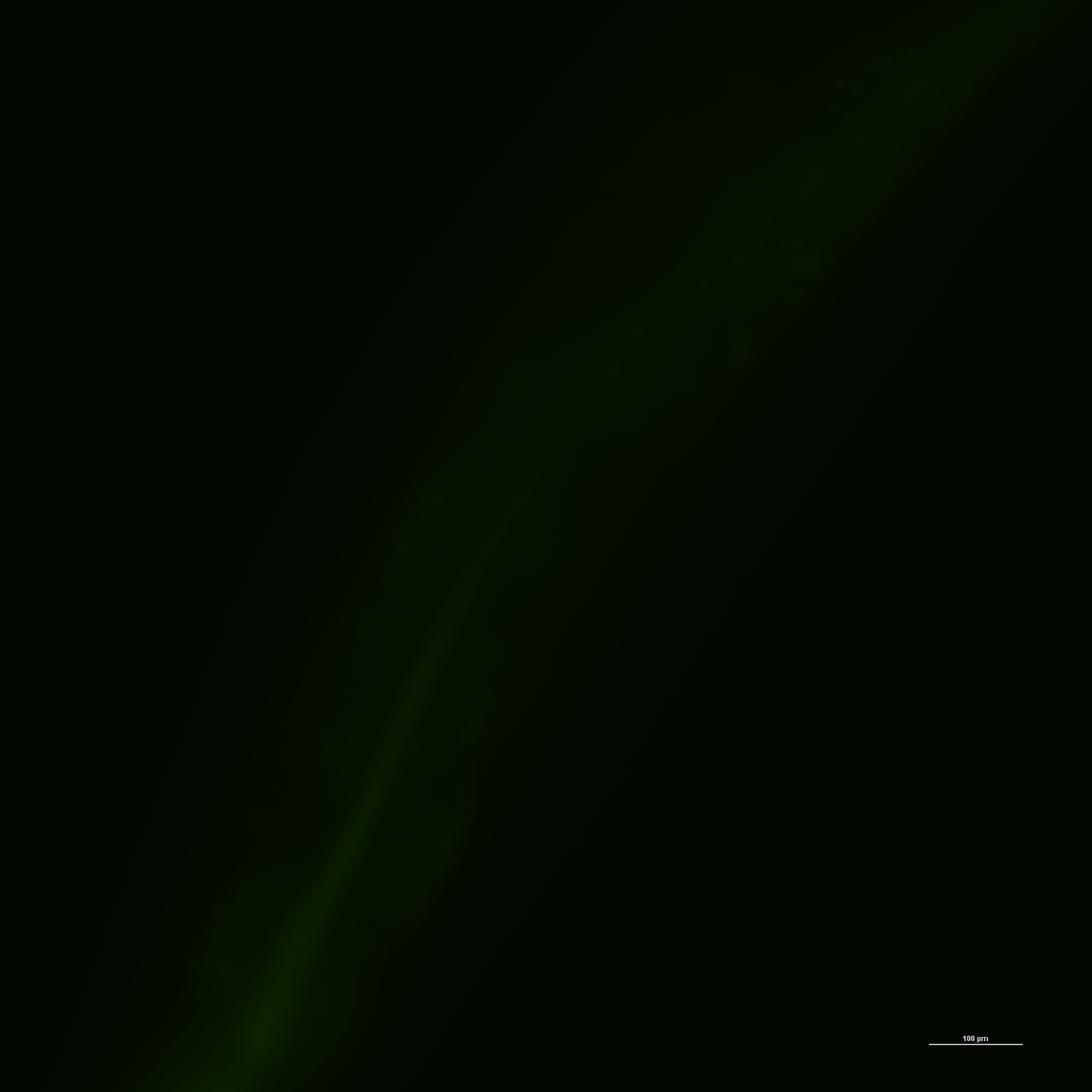

Supplement: Supplementary file 8 — Source data Fig. 5 [file 44318_2025_619_MOESM8_ESM.zip › Figure 5/5H/f.tif]

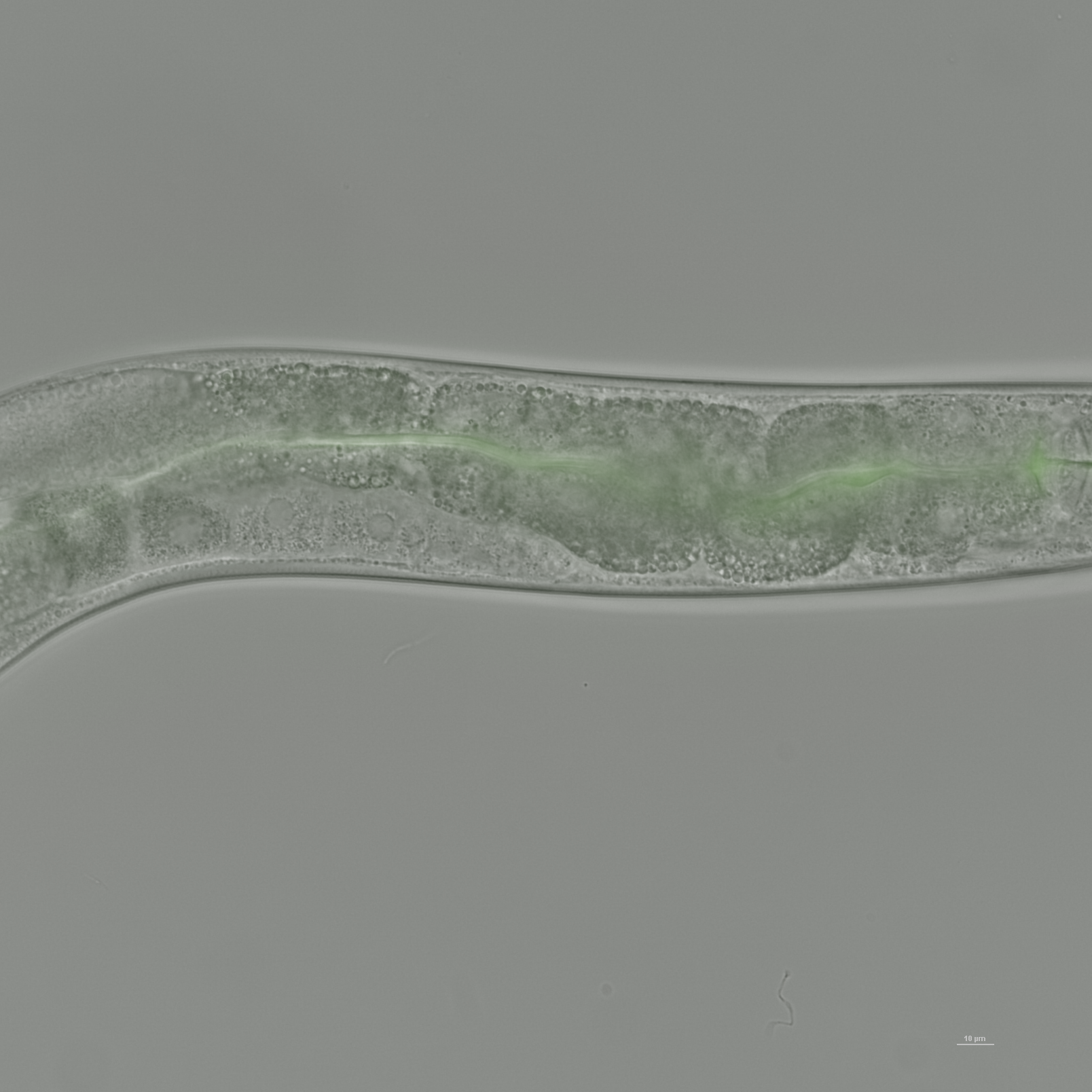

Supplement: Supplementary file 8 — Source data Fig. 5 [file 44318_2025_619_MOESM8_ESM.zip › Figure 5/5H/g.tif]

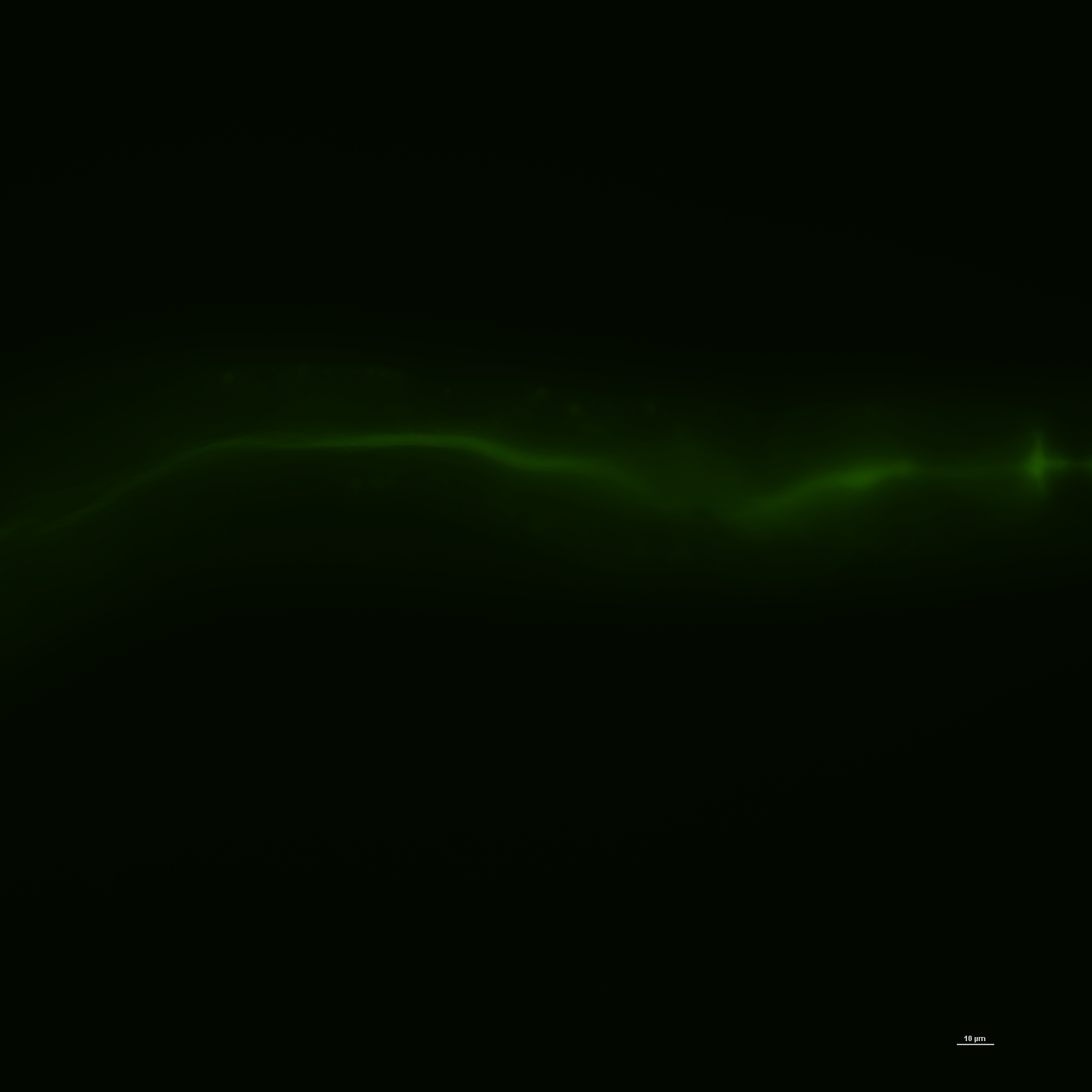

Supplement: Supplementary file 8 — Source data Fig. 5 [file 44318_2025_619_MOESM8_ESM.zip › Figure 5/5H/h.tif]

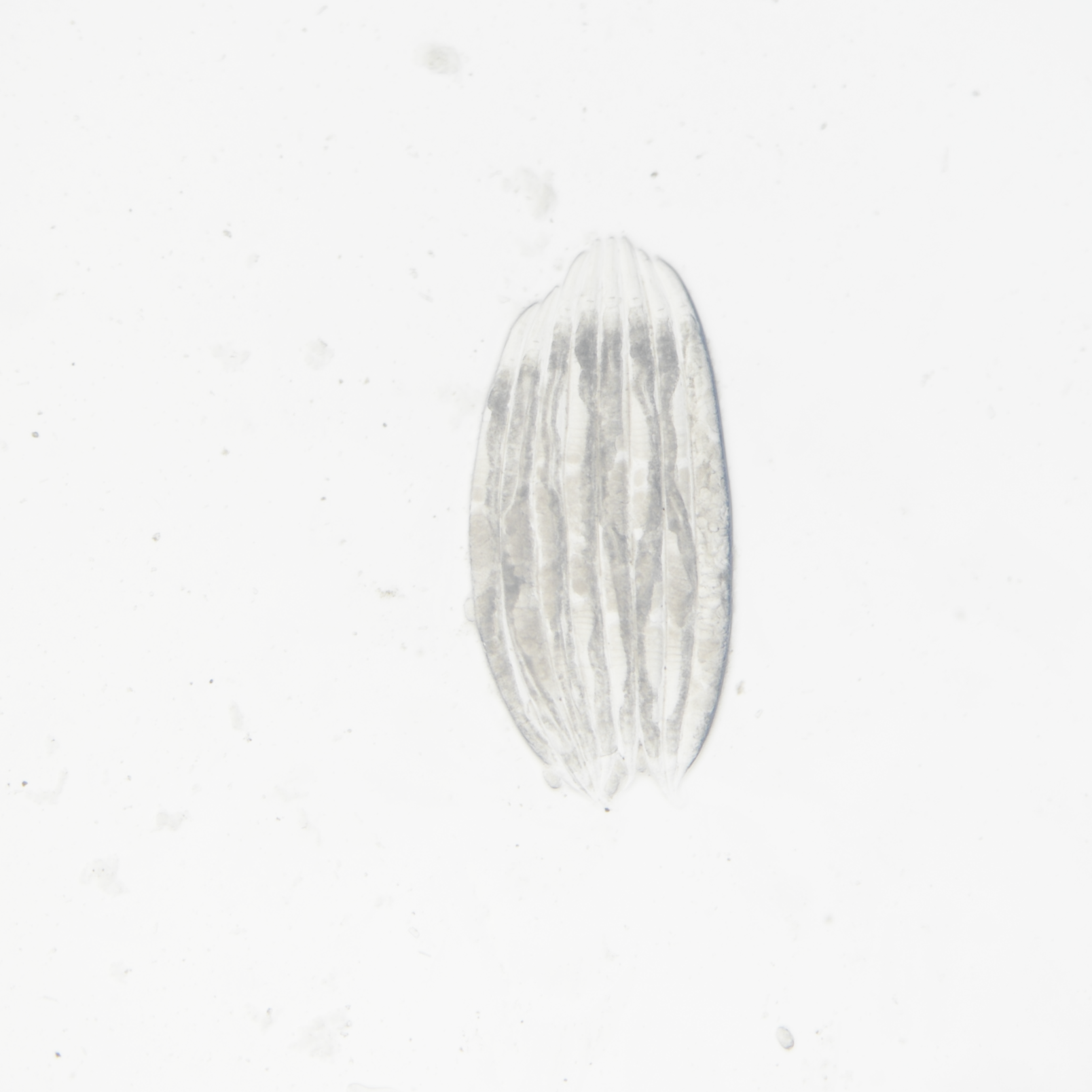

Supplement: Supplementary file 10 — Source data Fig. 7 [file 44318_2025_619_MOESM10_ESM.zip › Figure 7/7B/a.tif]

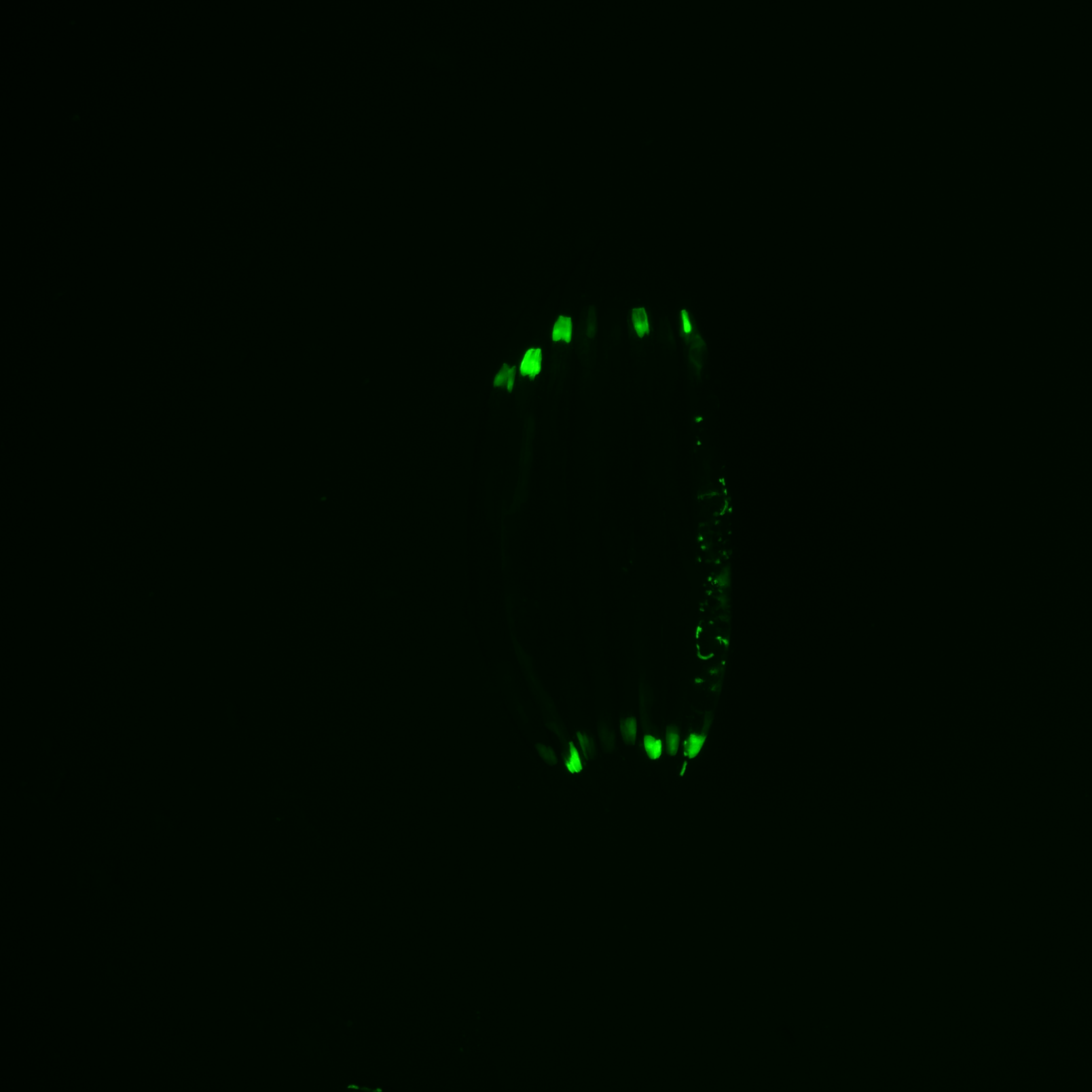

Supplement: Supplementary file 10 — Source data Fig. 7 [file 44318_2025_619_MOESM10_ESM.zip › Figure 7/7B/b.tif]

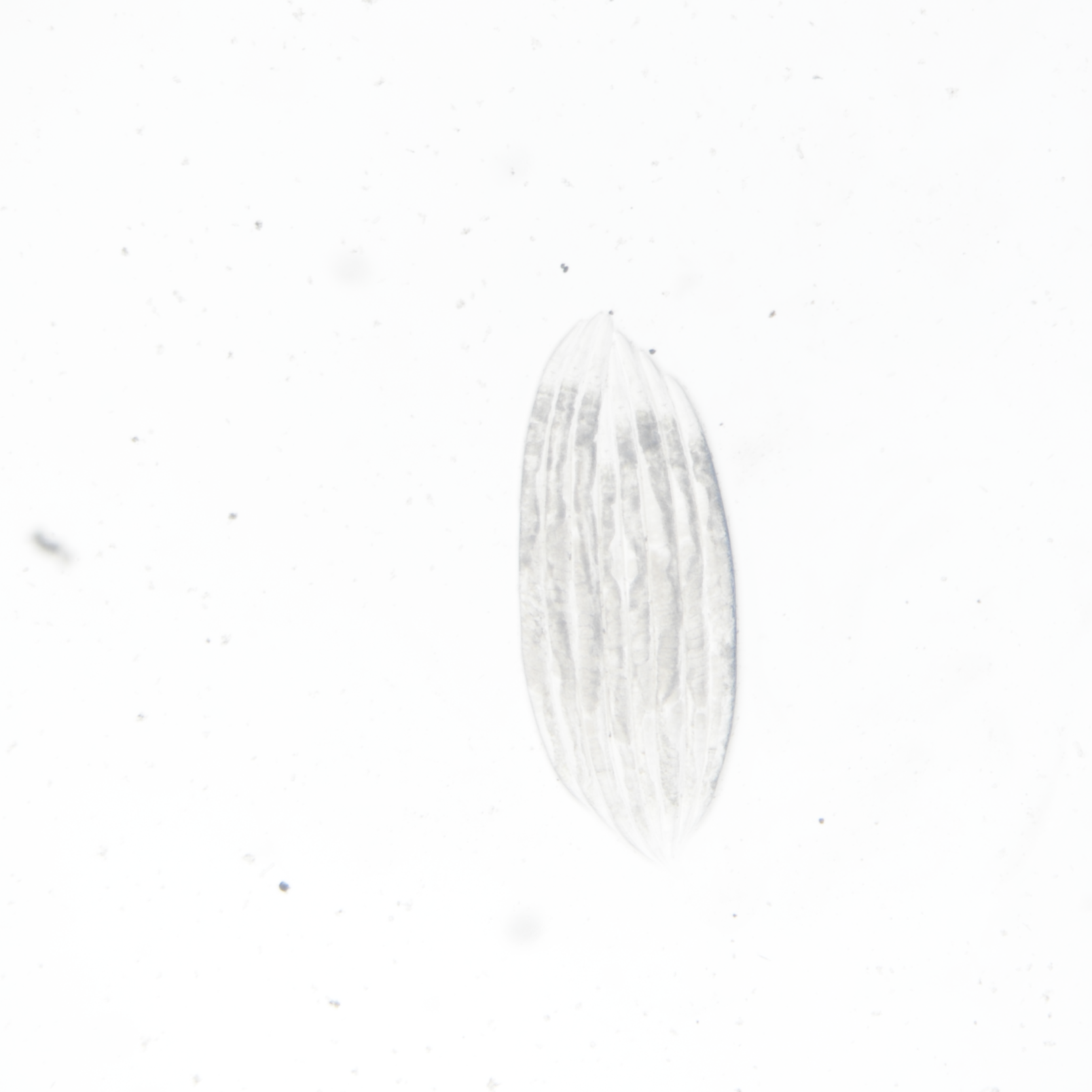

Supplement: Supplementary file 10 — Source data Fig. 7 [file 44318_2025_619_MOESM10_ESM.zip › Figure 7/7B/c.tif]

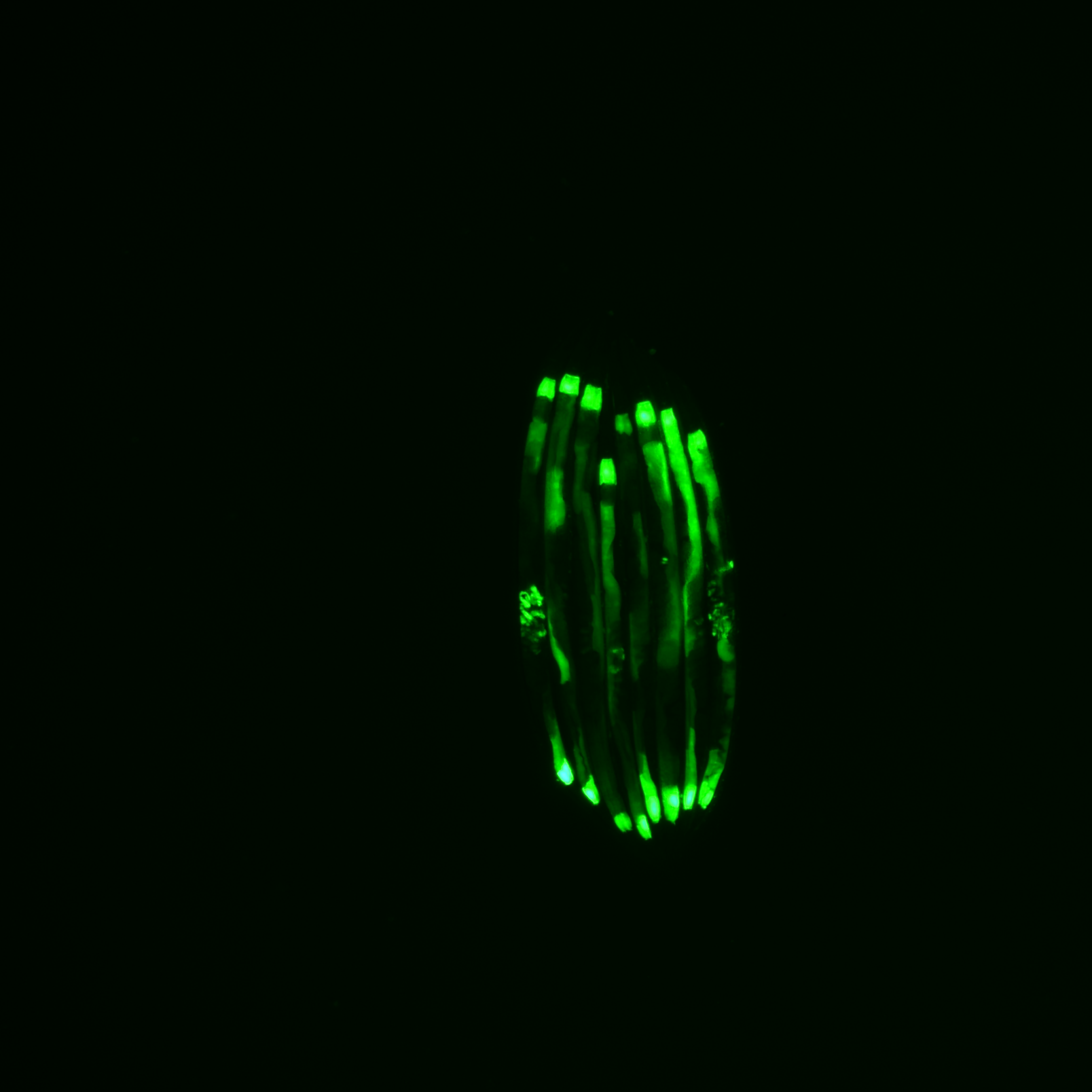

Supplement: Supplementary file 10 — Source data Fig. 7 [file 44318_2025_619_MOESM10_ESM.zip › Figure 7/7B/d.tif]

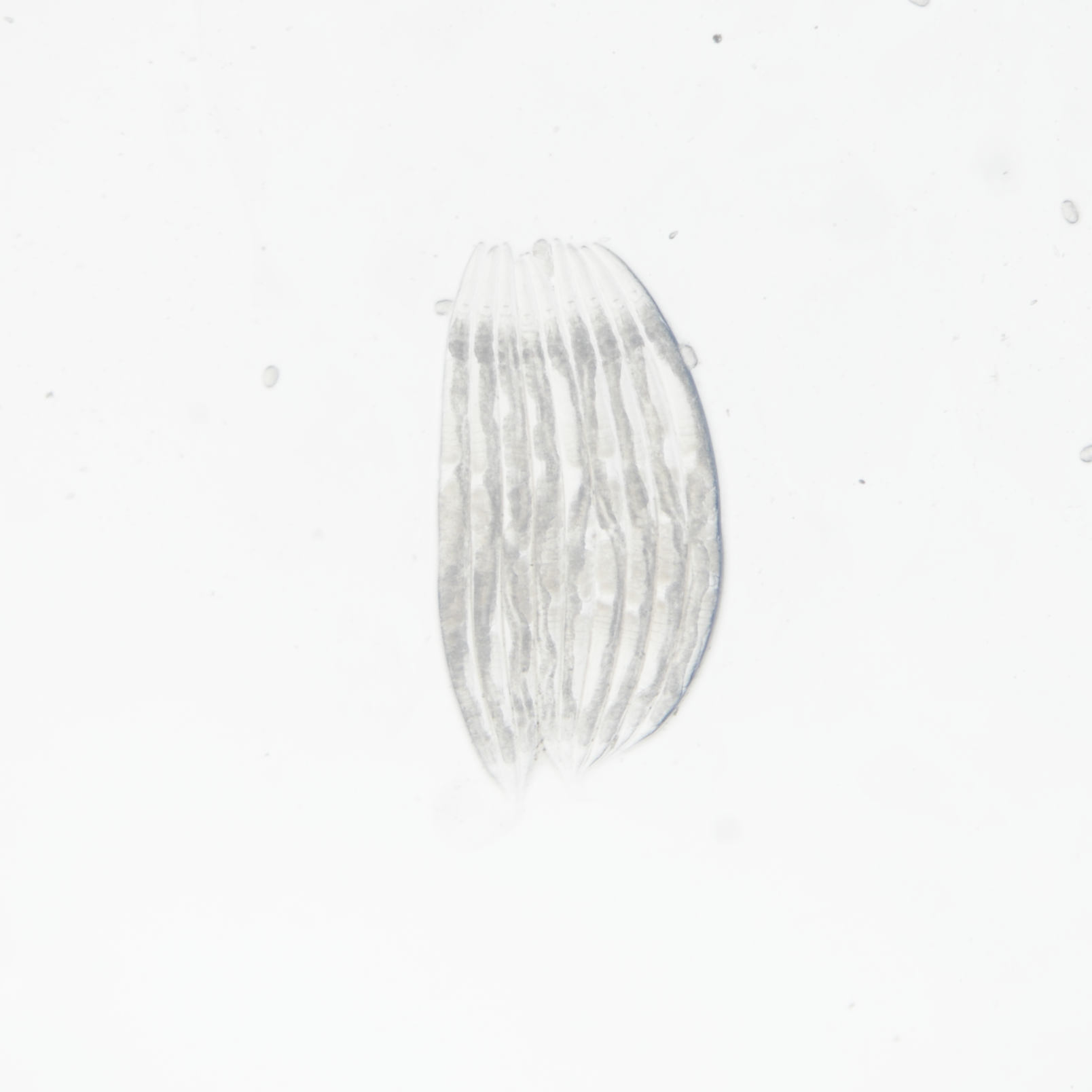

Supplement: Supplementary file 10 — Source data Fig. 7 [file 44318_2025_619_MOESM10_ESM.zip › Figure 7/7B/e.tif]

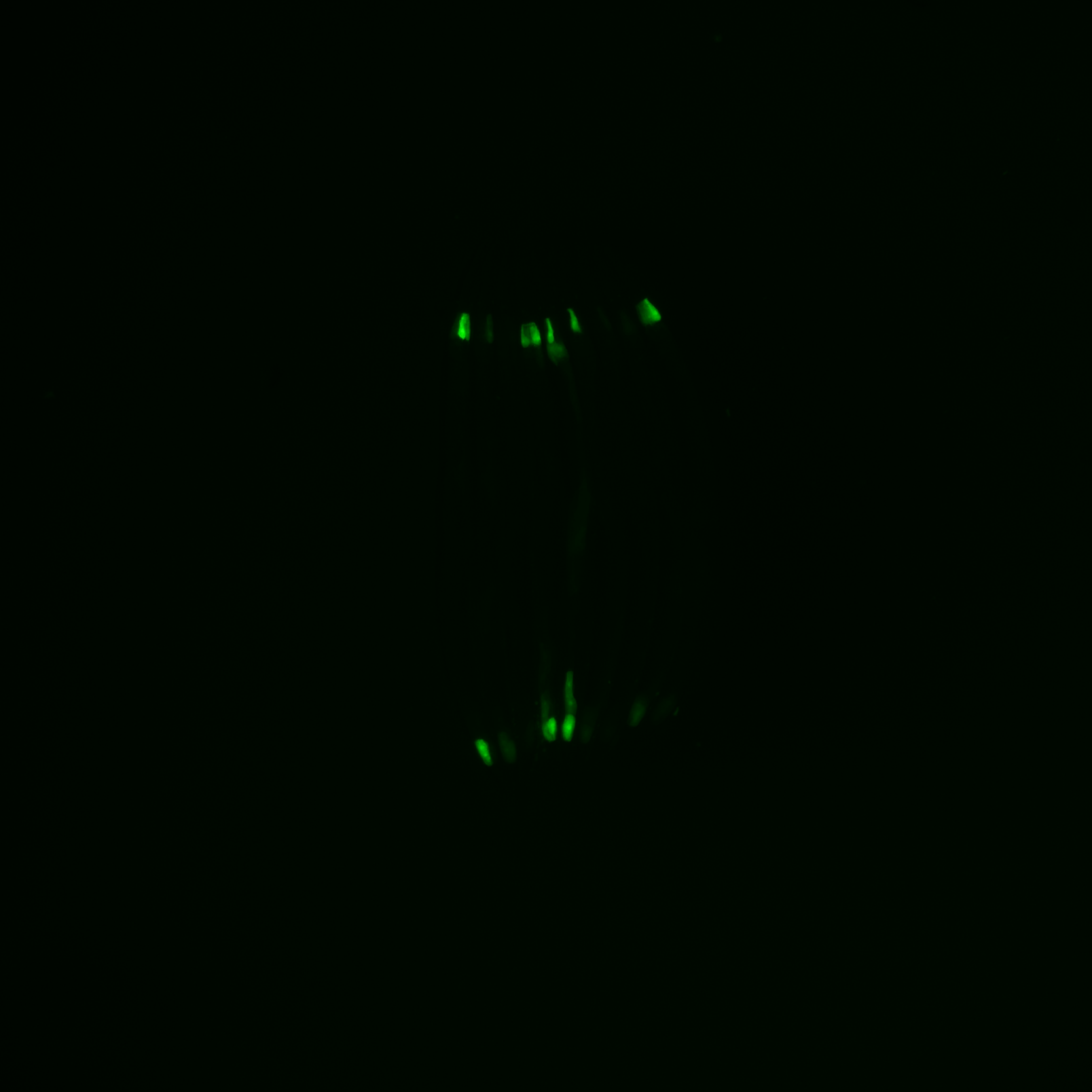

Supplement: Supplementary file 10 — Source data Fig. 7 [file 44318_2025_619_MOESM10_ESM.zip › Figure 7/7B/f.tif]

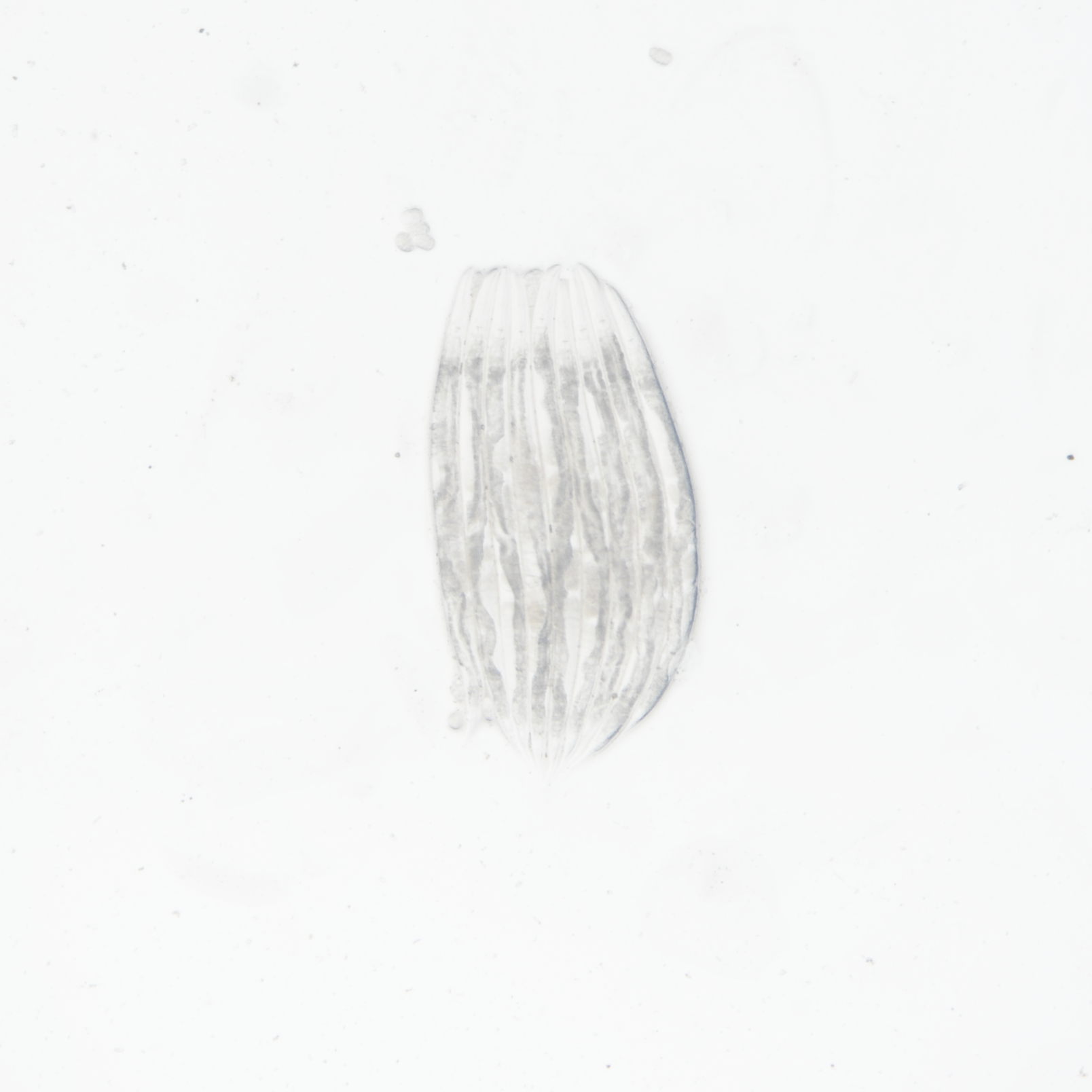

Supplement: Supplementary file 10 — Source data Fig. 7 [file 44318_2025_619_MOESM10_ESM.zip › Figure 7/7B/g.tif]

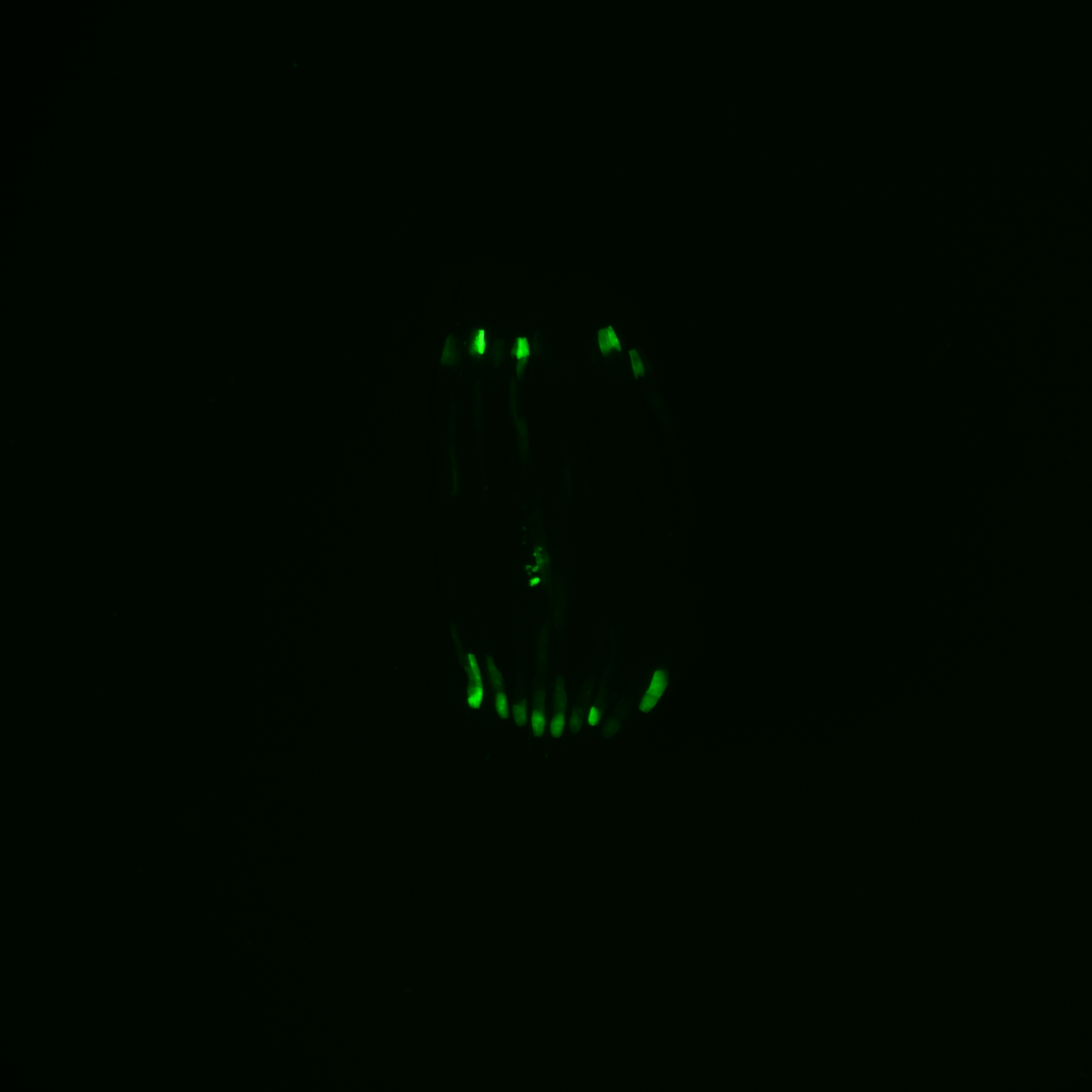

Supplement: Supplementary file 10 — Source data Fig. 7 [file 44318_2025_619_MOESM10_ESM.zip › Figure 7/7B/h.tif]
