# Supplementary material for: A gut-brain-gut axis orchestrates host responses counteracting microbiome-induced iron insufficiency
Source: EMBO J. 2025 Nov 3;44(24):7590–619. doi: 10.1038/s44318-025-00619-6 (PMC12705764; doi:10.1038/s44318-025-00619-6)
Supplement: Supplementary file 11 — Expanded View Figures [file 44318_2025_619_MOESM11_ESM.pdf]

Expanded View Figures

Figure EV1. Effects and categories of the bacterial genes that promote BAS-1 expression in *C. elegans* dopaminergic and serotonergic neurons following single deletion. ▶

(**A**) Graph showing the increase of BAS-1::GFP induced by the 29 *E. coli* mutants positive hits obtained from the screen described in Fig. 1B. % change in BAS-1::GFP fluorescence intensity were normalized to the expression levels in worms treated with wild-type BW25113 *E. coli*. Each dot indicates the effect of one mutant *E. coli* on the BAS-1::GFP level. $n >= 18$ each group. P values were determined by unpaired two-tailed t test. (**B–D**) Gene ontology analysis showing the enrichment of the screen hits in a variety of bacterial processes, including transportation and metabolism. GO analyses indicate the categories of the positive hits (**B**). Further GO analysis shows the detailed metabolic processes (**C**) and transportation processes (**D**) in which the bacterial genes, whose inactivation promotes BAS-1::GFP expression, are involved. (**E**) Bar graphs of qPCR analyses showing that *bas-1* mRNA levels are increased in the *Is[bas-1p::BAS-1::GFP]* worms treated with *ΔcyoB E. coli. Is[bas-1p::BAS-1::GFP]* animals treated with BW25113 were used as the control to calculate the fold change in mRNA levels. Data are the mean ± SEM. P values were determined by unpaired t test. $n > 200$ each group. (**F–K**) Representative chromatograms illustrate the acquisition time, ion pairs, and ion counts for serotonin (**F–H**) and dopamine (**I–K**) detection. (**F–H**) Present chromatograms of the serotonin commercial standard, serotonin in *C. elegans* fed BW25113, and serotonin in *ΔcyoB*-fed worms, respectively. (**I–K**) Depict the dopamine commercial standard, dopamine in BW25113-fed worms, and dopamine in *ΔcyoB*-fed worms, respectively. All panels exhibit retention time on the x axis versus ion counts on the y axis, with insets displaying the parent→product ion transitions (m/z) utilized for quantification.

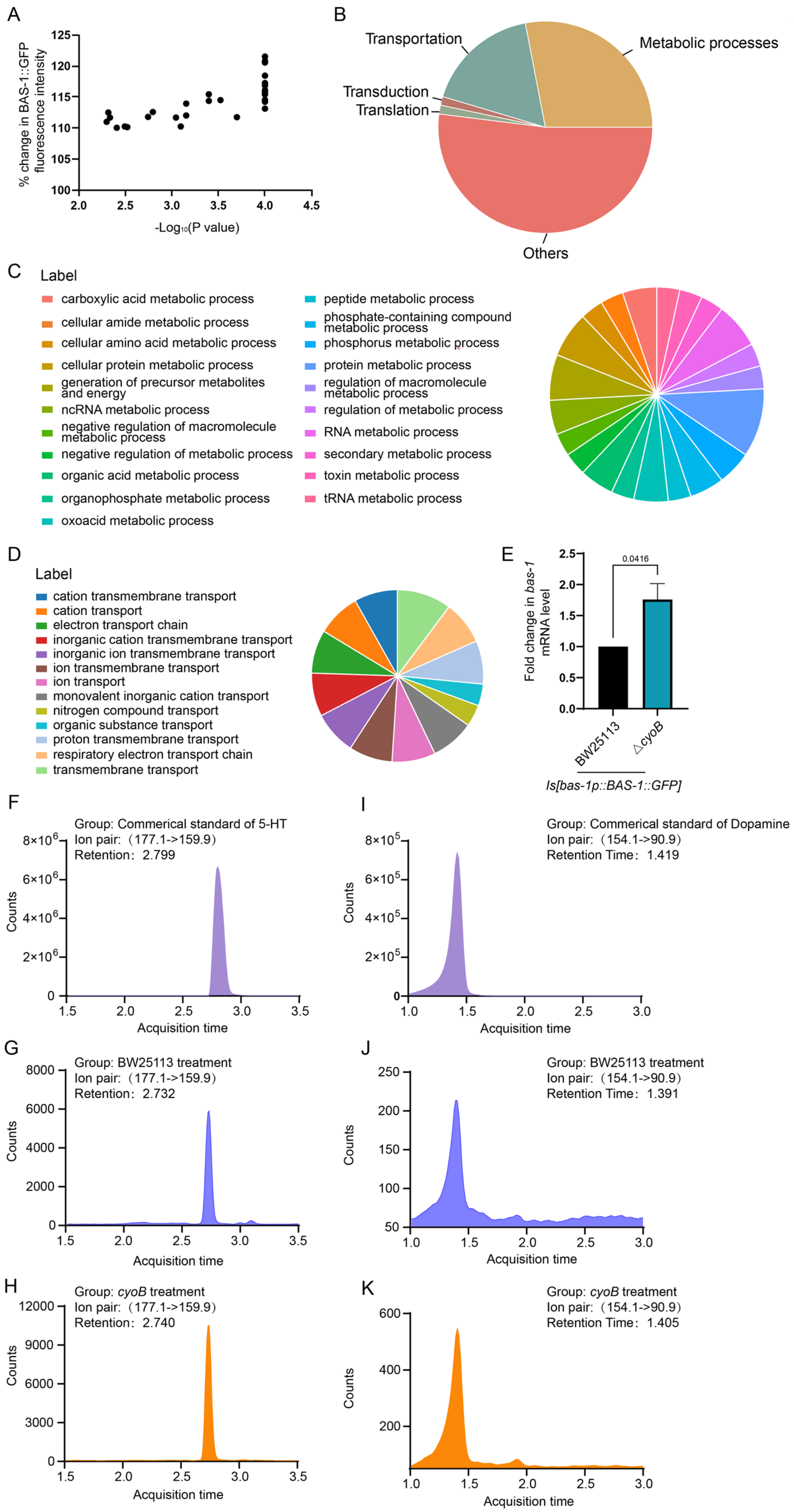
A
% change in BAS-1::GFP fluorescence intensity
125
120
115
110
105
100
2.0 2.5 3.0 3.5 4.0 4.5
-Log10(P value)
B
Transportation
Metabolic processes
Transduction
Translation
Others
C
Label
carboxylic acid metabolic process
cellular amide metabolic process
cellular amino acid metabolic process
cellular protein metabolic process
generation of precursor metabolites and energy
ncRNA metabolic process
negative regulation of macromolecule metabolic process
negative regulation of metabolic process
organic acid metabolic process
organophosphate metabolic process
oxoacid metabolic process
peptide metabolic process
phosphate-containing compound metabolic process
phosphorus metabolic process
protein metabolic process
regulation of macromolecule metabolic process
regulation of metabolic process
RNA metabolic process
secondary metabolic process
toxin metabolic process
tRNA metabolic process
D
Label
cation transmembrane transport
cation transport
electron transport chain
inorganic cation transmembrane transport
inorganic ion transmembrane transport
ion transmembrane transport
ion transport
monovalent inorganic cation transport
nitrogen compound transport
organic substance transport
proton transmembrane transport
respiratory electron transport chain
transmembrane transport
E
Fold change in bas-1 mRNA level
0.0416
2.5
2.0
1.5
1.0
0.5
0.0
BW25113
△cyoB
Is[bas-1p::BAS-1::GFP]
F
Group: Commerical standard of 5-HT
Ion pair: (177.1->159.9)
Retention: 2.799
Counts
8×10^6
6×10^6
4×10^6
2×10^6
0
1.5 2.0 2.5 3.0 3.5
Acquisition time
I
Group: Commerical standard of Dopamine
Ion pair: (154.1->90.9)
Retention Time: 1.419
Counts
8×10^5
6×10^5
4×10^5
2×10^5
0
1.0 1.5 2.0 2.5 3.0
Acquisition time
G
Group: BW25113 treatment
Ion pair: (177.1->159.9)
Retention: 2.732
Counts
8000
6000
4000
2000
0
1.5 2.0 2.5 3.0 3.5
Acquisition time
J
Group: BW25113 treatment
Ion pair: (154.1->90.9)
Retention Time: 1.391
Counts
250
200
150
100
50
1.0 1.5 2.0 2.5 3.0
Acquisition time
H
Group: cyoB treatment
Ion pair: (177.1->159.9)
Retention: 2.740
Counts
12000
9000
6000
3000
0
1.5 2.0 2.5 3.0 3.5
Acquisition time
K
Group: cyoB treatment
Ion pair: (154.1->90.9)
Retention Time: 1.405
Counts
600
400
200
1.0 1.5 2.0 2.5 3.0
Acquisition time

 © The Author(s)

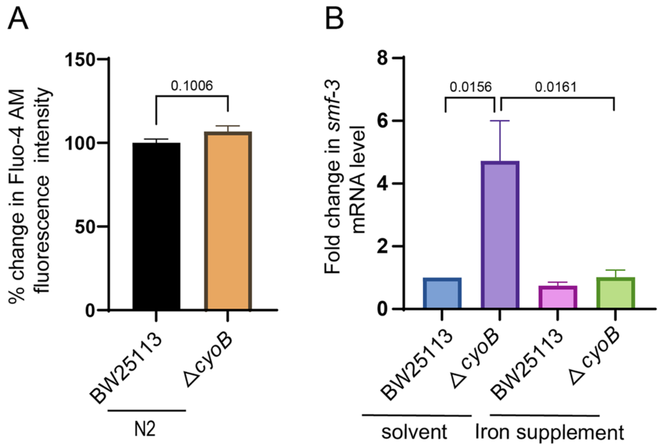

Figure EV2. The *ΔcyoB E. coli* treatment induces *smf-3* transcription, which is suppressed by iron supplementation.

(**A**) Bar graphs indicating no changes in Fluo-4 AM fluorescence in the host *C. elegans* treated with *ΔcyoB E. coli* compared to those treated with BW25113 *E. coli*. Data represent the mean ± SEM. ns > 0.05 by unpaired *t* test. *n* > 30 in each group. (**B**) qPCR results showing that transcription of *smf-3*, encoding transporters for iron absorbing in *C. elegans* (Romney et al, 2011), is increased in response to *ΔcyoB E. coli* treatment. 4 mM $FeCl_3$ was used for iron supplementation. Wild-type animals treated with BW25113 *E. coli* were used as the control to calculate the fold change in *smf-3* mRNA levels. Data are the mean ± SEM. *P* values were determined by one-way ANOVA; *n* > 200 each group.

© The Author(s)

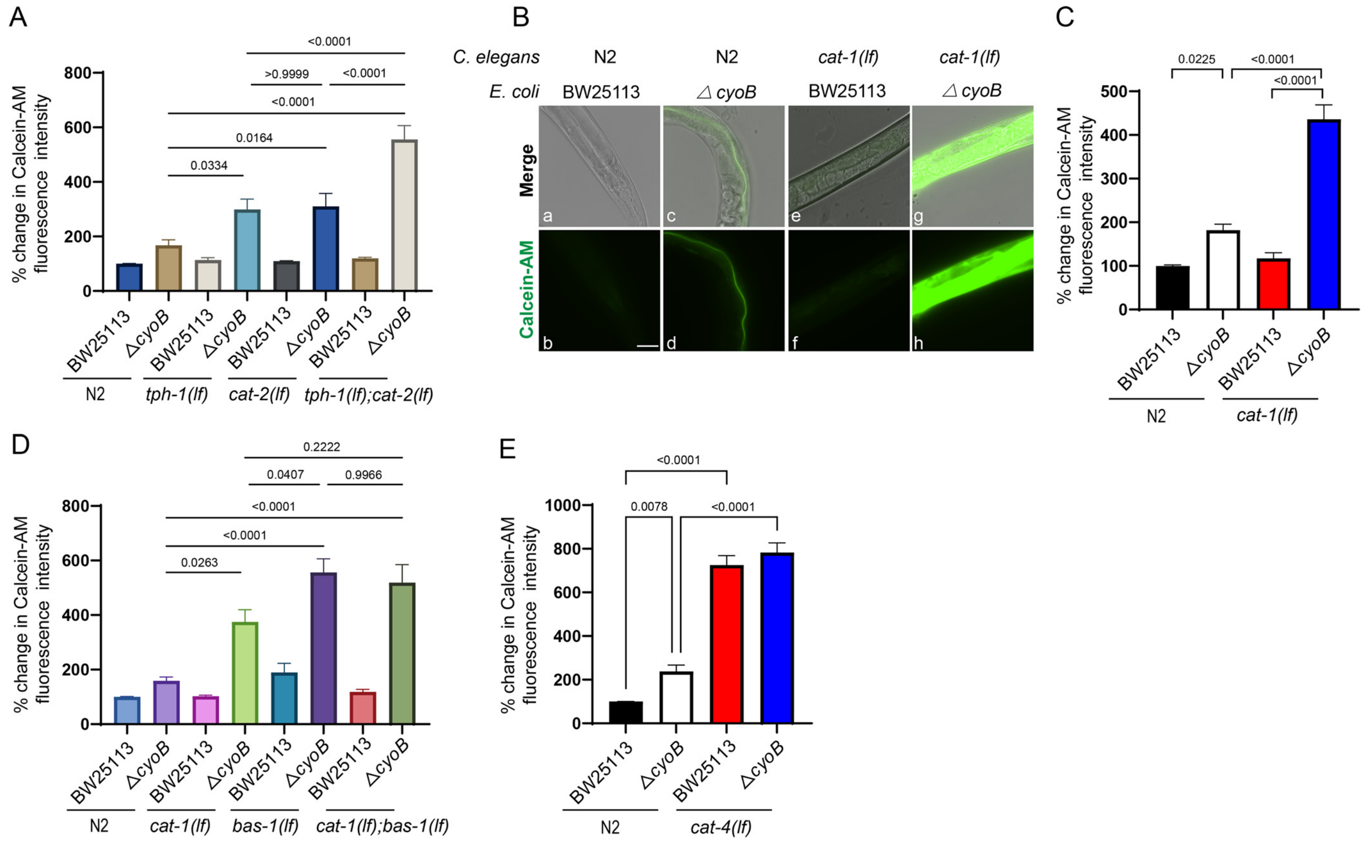

Figure EV3. Inhibiting dopamine and serotonin release by blocking their import into vesicles exacerbates the reduction in labile iron caused by *ΔcyoB E. coli.*

(**A**) Bar graphs indicating that both *tph-1(lf)* and *cat-2(lf)* mutations exacerbated iron reduction induced by *ΔcyoB E. coli*. *tph-1(lf);cat-2(lf)* displays a stronger reduction in labile iron compared to each single mutant. The *Y* axis represents the percentage change in Calcein fluorescence, normalized to the levels observed in wild-type N2 animals treated with BW25113 bacteria. (**B**, **C**) Images and bar graph indicating that the *cat-1(lf)* mutation caused a further reduction in the labile iron level in the presence of *ΔcyoB*. The % change in the Calcein fluorescence was calculated by normalization to the levels in wild-type N2 animals treated with BW25113 bacteria. CAT-1 is required for transporting monoamines, including dopamine and serotonin, into vesicles for release. Scale bar, 50 µm. (**D**, **E**) Bar graphs depicting the Calcein signal in worms with indicated genotypes and bacterial treatments. The percentage change in Calcein fluorescence was calculated by normalizing to the levels observed in wild-type N2 animals treated with BW25113 bacteria. For all panels, experiments were performed for at least three times. Data are the mean ± SEM. *P* values were determined by one-way ANOVA; *n* > 25 each group.

 © The Author(s)

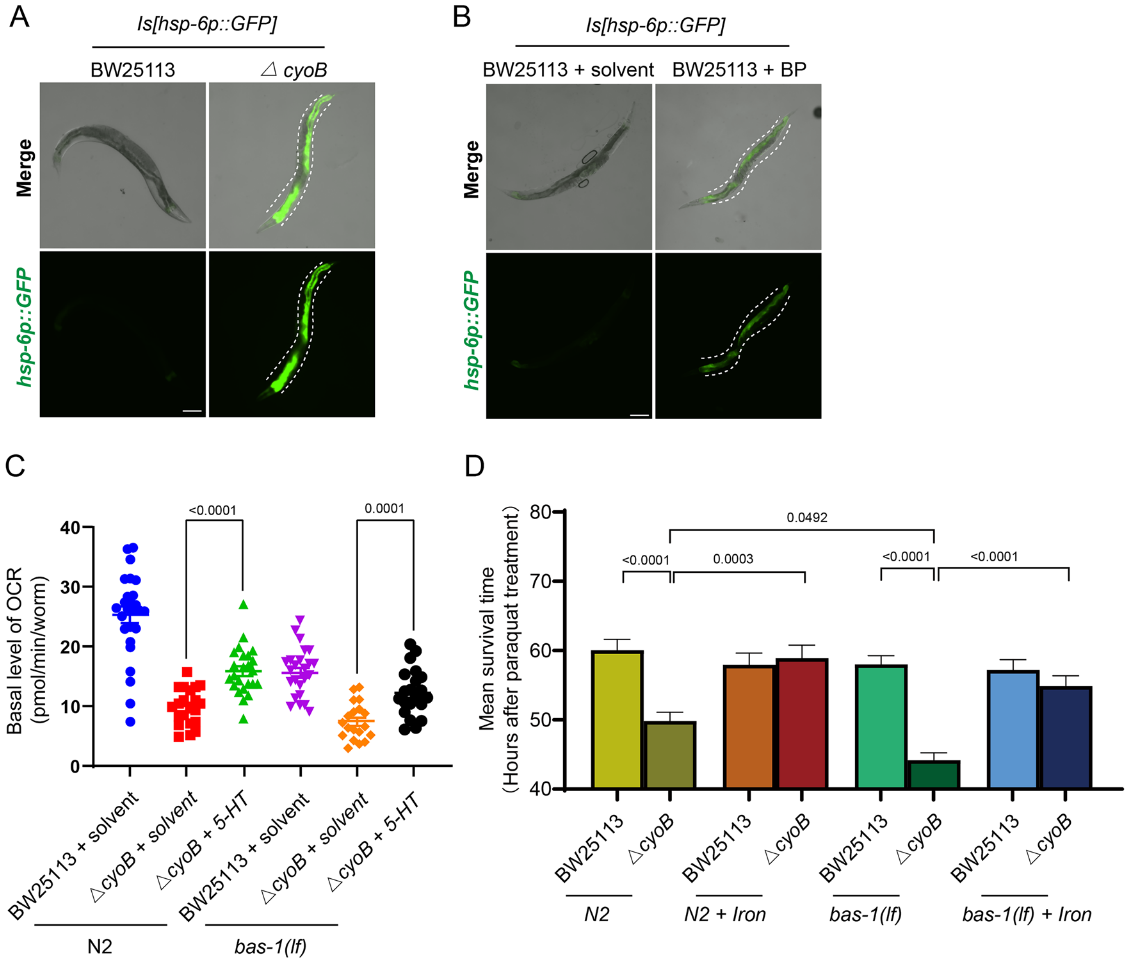

Figure EV4. The intestinal mitochondria impairment induced by the *ΔcyoB E. coli* and iron chelator treatment and the role of serotonergic and dopaminergic neurons response to the presence of *ΔcyoB E. coli* in preventing decline in mitochondrial function.

(**A**) Microscopic images indicating that *ΔcyoB* mutant *E. coli* mainly induces mitochondrial stress in the intestine of *C. elegans*. The induction of *hsp-6p::gfp* expression in the intestine (white lines) was detected in the presence of *ΔcyoB E. coli*. Scale bar, 100 μm. (**B**) Images showing that the iron chelator BP (25 μM) induces *hsp-6p::gfp* expression mainly in the intestine of *C. elegans*. White lines indicate the location of the intestine. Scale bar, 100 μm. (**C**) Bar graphs depicting the reduction in basal oxygen consumption rate (OCR) in wild-type N2 and *bas-1(lf)* worms treated with *ΔcyoB E. coli*, which was partially reversed by serotonin (5-HT) supplementation. Each dot represents the OCR normalized to each worm. P values were determined by unpaired *t* test; $n > 50$ per group. (**D**) Bar graph showing that the increase in *bas-1* expression in response to *ΔcyoB* bacteria is important for preventing a further decline in mitochondrial function. Paraquat was used to challenge mitochondrial function, and the mean survival time of *C. elegans* with the indicated genotypes and treatments was calculated as described in the Methods. Data are the mean ± SEM. *P* values were determined by one-way ANOVA; $n > 120$ each group.

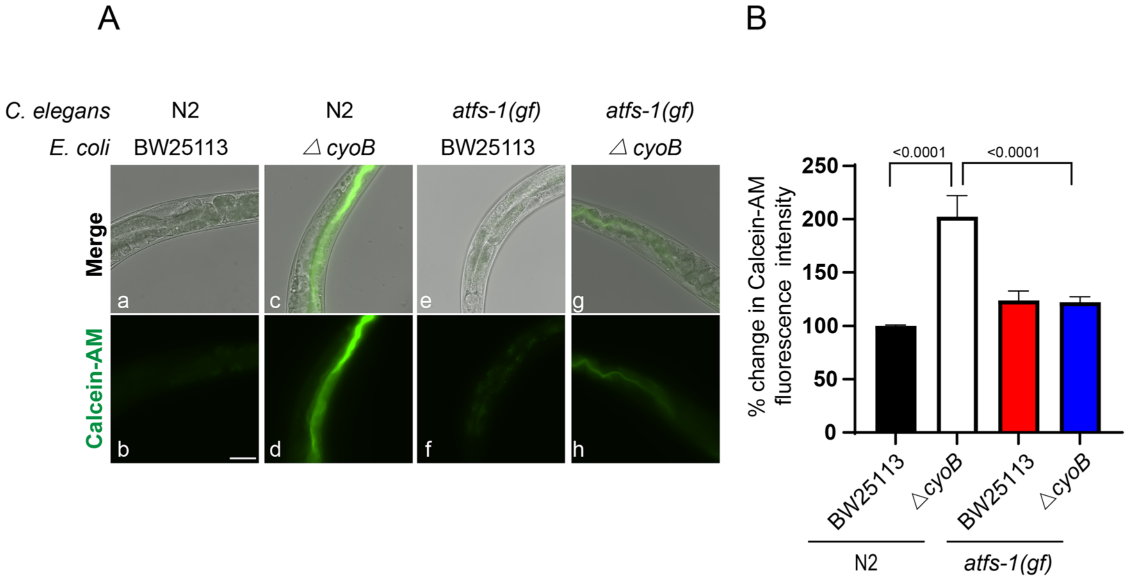

Figure EV5. Over-activation of ATFS-1 is able to alleviate the reduction in labile iron levels caused by *ΔcyoB E. coli*.

(**A**, **B**) Images and bar graph indicating that the dominant active form of ATFS-1 suppressed the reduction in labile iron level caused by the *ΔcyoB E. coli* treatment. Calcein-AM staining was performed in N2 and *atfs-1(gf)* worms with the indicated bacterial treatments to indicate the labile iron levels. The % change in Calcein fluorescence was calculated by normalization to the levels in N2 worms treated with BW25113. Data are the mean ± SEM. *P* values were determined by one-way ANOVA. *n* > 25 each group. Scale bar, 50 µm.

 © The Author(s)

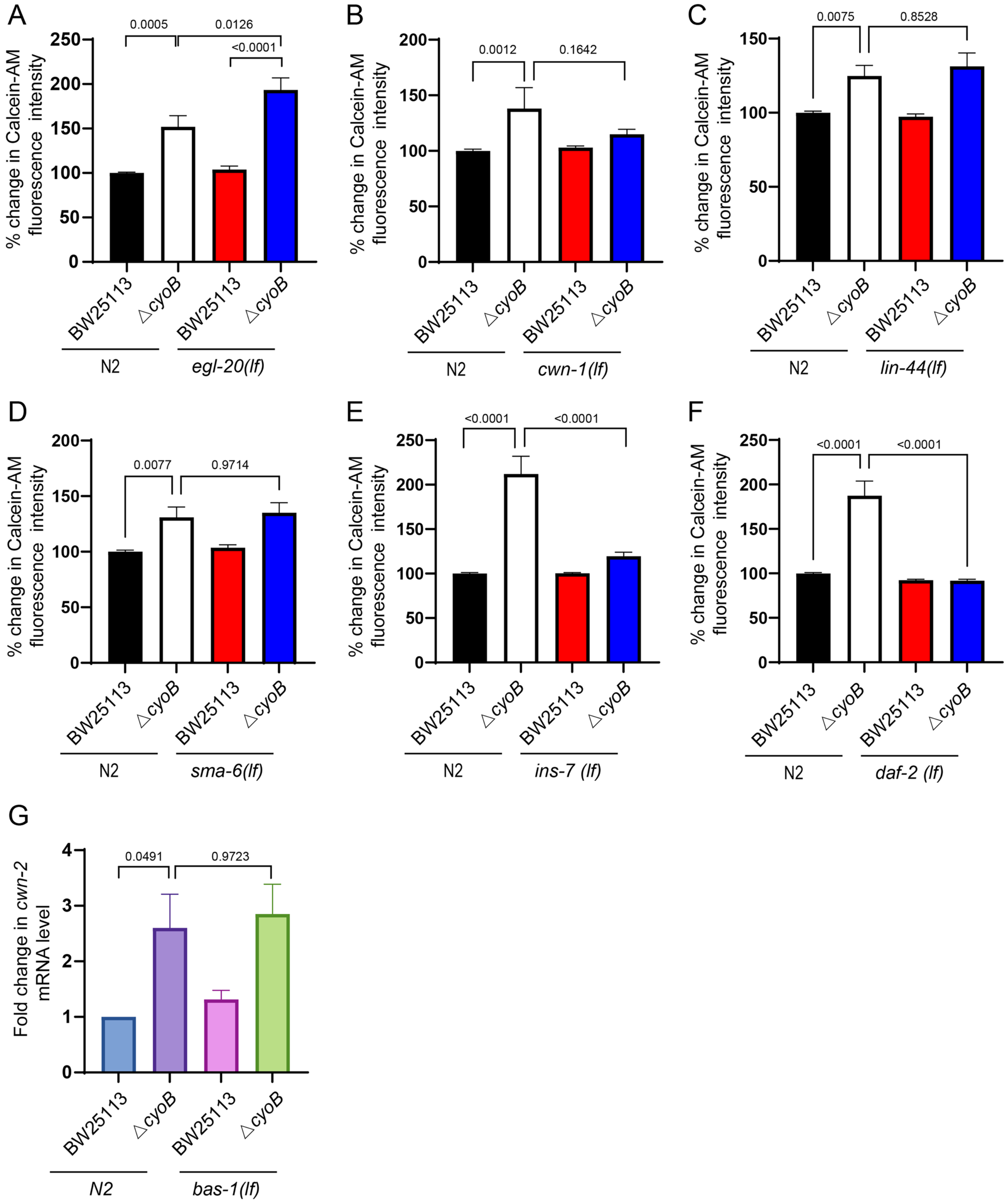
A
% change in Calcein-AM fluorescence intensity
0.0005
0.0126
<0.0001
BW25113
△cyoB
BW25113
△cyoB
N2
egl-20(lf)
B
0.0012
0.1642
cwn-1(lf)
C
0.0075
0.8528
lin-44(lf)
D
0.0077
0.9714
sma-6(lf)
E
<0.0001
<0.0001
ins-7 (lf)
F
<0.0001
<0.0001
daf-2 (lf)
G
Fold change in cwn-2 mRNA level
0.0491
0.9723
bas-1(lf)

Figure EV6. Wnt ligands are required for alleviating iron deficiency caused by the Δ*cyoB E. coli* mutant.

(**A–F**) Bar graphs depicting the change in Calcein signal in worms with the indicated genotypes and treatments. The fold change was calculated by normalizing to the levels in N2 worms treated with BW25113. *P* values were determined by one-way ANOVA. $n > 30$ per group. (**G**) qPCR analyses indicate that deletion of *bas-1* shows no effect on the transcription of *cwn-2*, supporting that *bas-1* functions downstream of *cwn-2*. The mRNA of *cwn-2* in worms with the indicated genotypes and treatments was analyzed by qPCR. In the presence of Δ*cyoB E. coli* mutant bacteria, the *bas-1(lf)* worms displayed similar levels of *cwn-2* mRNA to that in wild-type N2 *C. elegans*. N2 wild-type animals treated with BW25113 was used as the control to calculate the fold change in mRNA levels. *P* values were determined by one-way ANOVA. $n > 200$ each group.

© The Author(s)

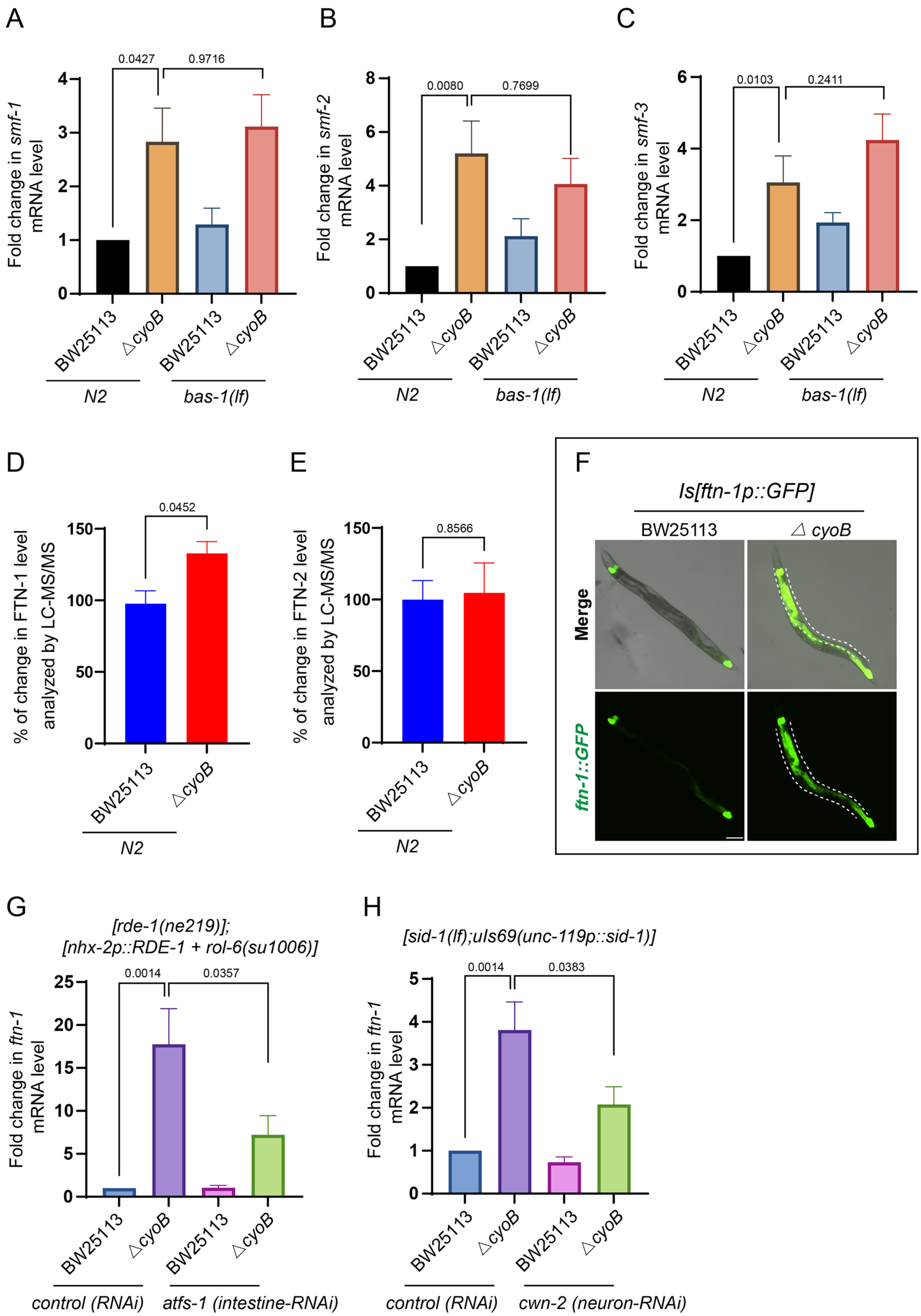
A
0.0427
0.9716
Fold change in smf-1 mRNA level
BW25113
△cyoB
BW25113
△cyoB
N2
bas-1(lf)
B
0.0080
0.7699
Fold change in smf-2 mRNA level
C
0.0103
0.2411
Fold change in smf-3 mRNA level
D
0.0452
% of change in FTN-1 level analyzed by LC-MS/MS
E
0.8566
% of change in FTN-2 level analyzed by LC-MS/MS
F
Is[ftn-1p::GFP]
BW25113
△ cyoB
Merge
ftn-1::GFP
G
[rde-1(ne219)];
[nhx-2p::RDE-1 + rol-6(su1006)]
0.0014
0.0357
Fold change in ftn-1 mRNA level
control (RNAi)
atfs-1 (intestine-RNAi)
H
[sid-1(lf);uIs69(unc-119p::sid-1)]
0.0014
0.0383
Fold change in ftn-1 mRNA level
control (RNAi)
cwn-2 (neuron-RNAi)

Figure EV7. In response to the *ΔcyoB E. coli* mutant, increased transcription of ferritin but not iron absorption genes is dependent on the host neuronal response.

(**A–C**) qPCR results show that *bas-1* is not required for the induction of *smf-1/-2/-3* mRNA expression caused by the *ΔcyoB E. coli*. The transcription of *smf-1/-2/-3*, genes encoding transporters for absorbing iron in *C. elegans* (Romney et al, 2011), are increased in response to *ΔcyoB E. coli*. In the presence of *ΔcyoB E. coli*, the *bas-1(lf)* worms showed no significant difference in the level of *smf-1/-2/-3* mRNA compared with N2 wild-type *C. elegans*. Wild-type animals treated with BW25113 was used as the control to calculate the fold change in mRNA levels. $n > 200$ each group. (**D**, **E**) Bar graph showing LC-MS/MS analyses of FTN-1 and FTN-2 protein levels in *C. elegans* treated with BW25113 or *ΔcyoB E. coli*. The percent change of FTN-1 (**D**) or FTN-2 (**E**) was calculated by normalizing to the levels from the *C. elegans* treated with BW25113. $n > 500$ each group. *P* values were determined by unpaired t test. (**F**) Microscopic images showing the intestine-specific expression of *ftn-1* and its induction by the presence of *ΔcyoB*. The induction of the Is[*ftn-1p::GFP*] was observed with *ΔcyoB* treatment when compared to the BW25113 group. The intestine was outlined. Scale bar, 100 µm. (**G**) qPCR results show that intestine *atfs-1* is at least partially required for the increase in *ftn-1* transcription in response to *ΔcyoB E. coli* mutant bacteria. The VP303 worm strain was used to perform intestine-specific knockdown of *atfs-1* in the presence of the indicated bacteria. Fold change was determined by normalization to the animals fed with both control RNAi and BW25113 bacteria. $n > 200$ each group. (**H**) qPCR analyses show that neuronal *cwn-2* is at least partially required for the induction of *ftn-1* mRNA expression in the presence of *ΔcyoB E. coli* mutant bacteria. TU3401[*sid-1(lf); uIs69(unc-119p::sid-1)*] was used to knock down *cwn-2* specifically in neurons. *ΔcyoB E. coli*-induced *ftn-1* transcription is partially suppressed by *cwn-2(neuron-RNAi)*. $n > 200$ each group. For (**A–C**, **G**, **H**), data are the mean ± SEM. *P* values were determined by one-way ANOVA.

 © The Author(s)
